# Azetidine synthesis by La(OTf)_3_-catalyzed intramolecular regioselective aminolysis of *cis*-3,4-epoxy amines

**DOI:** 10.3389/fchem.2023.1251299

**Published:** 2023-09-19

**Authors:** Yuse Kuriyama, Yusuke Sasano, Yoshiharu Iwabuchi

**Affiliations:** Department of Organic Chemistry, Graduate School of Pharmaceutical Sciences, Tohoku University, Sendai, Japan

**Keywords:** azetidine, epoxide, catalytic reaction, regioselectivity, cyclization, Lewis acid, synthetic methods

## Abstract

Azetidine is a prevalent structural motif found in various biologically active compounds. In this research paper, we report La(OTf)_3_-catalyzed intramolecular regioselective aminolysis of *cis*-3,4-epoxy amines to afford azetidines. This reaction proceeded in high yields even in the presence of acid-sensitive and Lewis basic functional groups.

## 1 Introduction

Arranging specific polar functional groups in three-dimensional space is a basic strategy for imparting a specific bioactive function to organic molecules and is universally found in nature and used in medicinal chemistry. The regioselective nucleophilic ring opening of epoxides is an efficient strategy for constructing contiguous chiral centers, and many methods have been developed to achieve this reaction (Caron and Sharpless, 1985; Faiz and Zahoor, 2016; Wang, 2017; Rodríguez-Berríos et al., 2023). Aminolysis of epoxides is a useful reaction for the synthesis of sterically complex nitrogen-containing compounds such as alkaloids. However, the regioselective aminolysis of epoxides poses a significant challenge, especially when Lewis and Brønsted acid promoters are used as catalysts. This is because the acid added to activate the epoxide is usually quenched by the high basicity of amine nucleophiles.

**SCHEME 1:**
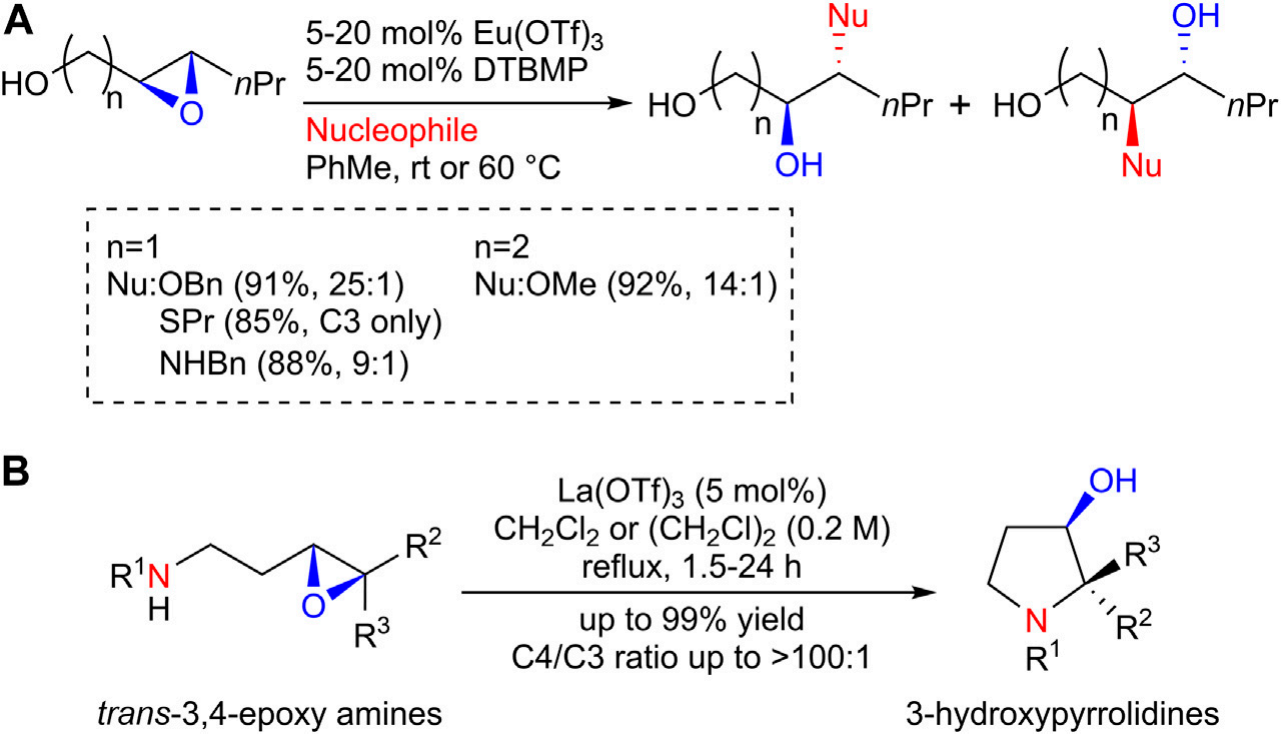
**(A)** Eu(OTf)_3_-catalyzed regioselective ring-opening reaction of epoxy alcohols; **(B)** La(OTf)_3_-catalyzed regioselective ring-opening reaction of *trans*-3,4-epoxy amines.

In the course of our study on the total synthesis of biologically active natural products (Uesugi et al., 2015), our research group discovered that lanthanoid (III) trifluoromethanesulfonate (Ln(OTf)_3_) functions as an excellent catalyst for the regioselective nucleophilic ring opening of epoxides, which led us to exploit the synthetic use of Ln(OTf)_3_ as a catalyst for epoxide ring-opening reactions. Thus, we have demonstrated that a catalytic amount of europium (III) trifluoromethanesulfonate (Eu(OTf)_3_) enables the introduction of alcohols and thiols, as well as aryl and aliphatic amines, onto the C3 position of 2,3-epoxy alcohols with high regioselectivity (Scheme 1A) (Uesugi et al., 2014). Eu(OTf)_3_ also efficiently catalyzed the C4-selective aminolysis of a 3,4-epoxy alcohol, the synthetic use of which was demonstrated by the efficient synthesis of the antipsychotic agent (+)-nemonapride (Uesugi et al., 2017). Furthermore, the lanthanum (III) trifluoromethanesulfonate (La(OTf)_3_) catalyst induced anti-Baldwin 5-*endo*-*tet* cyclization of *trans*-3,4-epoxy amines via C4-selective intramolecular epoxide aminolysis to give 3-hydroxypyrrolidines in high yields (Scheme 1B) (Kuriyama et al., 2021). Interestingly, the La(OTf)_3_ catalyst was found to promote the C3-selective intramolecular aminolysis of a *cis*-3,4-epoxy amine to give an azetidine in high yield, which led to the development of a novel synthetic route for azetidines, as reported herein.

**SCHEME 2:**
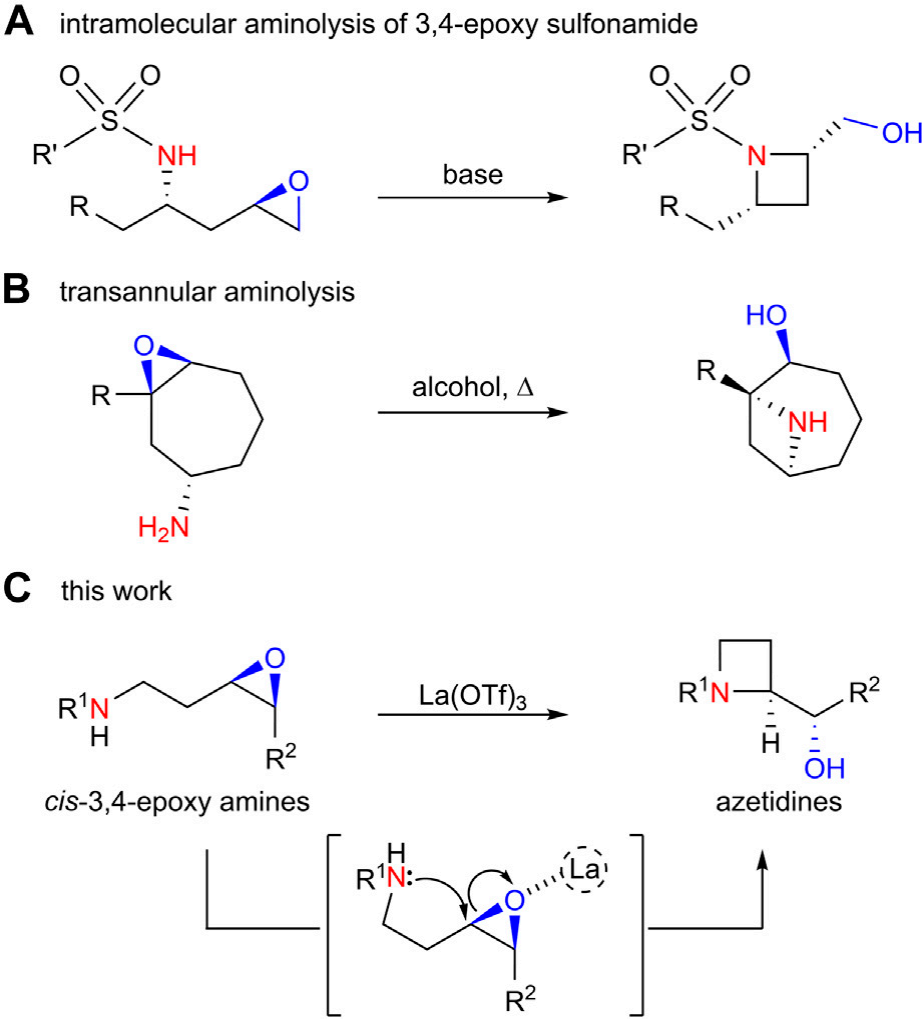
Azetidine syntheses by aminolysis reaction of epoxides; **(A)** intramolecular aminolysis of 3,4-epoxy sulfonamide; **(B)** transannular aminolysis; **(C)** this work.

Azetidine is a structural motif found in various natural products and pharmaceuticals. This strained structure has encouraged synthetic chemists to develop strategies for the synthesis of azetidines (Hameed et al., 2017; Parmar et al., 2021; Yoda et al., 2011). Intramolecular S_N_2 reactions are often used to form azetidine rings in which a nitrogen atom attacks a carbon atom connected to a leaving group [halogen (Betz et al., 2019; Rowe et al., 2019; Dherange et al., 2022), mesylate (Bose et al., 1994), *etc.*]. The intramolecular aminolysis of 3,4-epoxy amines could be an alternative method for constructing an azetidine ring with a carbonyl group adjacent to the azetidine ring, which could be a useful scaffold for further functionalization. However, such reactions have rarely been reported, except the intramolecular aminolysis of 3,4-epoxy sulfonamide (Scheme 2A) (Moulines et al., 1993; Breternitz and Schaumann, 1999; Medjahdi et al., 2009; Faigl et al., 2012) and transannular aminolysis of 3,4-epoxy amine (Scheme 2B) (Shimokawa et al., 2011; Shing and So, 2011; Wang et al., 2018; Hocine et al., 2023). To the best of our knowledge, the intramolecular aminolysis of an unprotected linear 3,4-epoxy amine (rather than amide) has not been reported before. Herein, we describe further investigations to clarify the optimum conditions and substrate scope for the La(OTf)_3_-catalyzed highly regioselective 4-*exo*-*tet* cyclization of linear 3,4-epoxy amines to afford azetidines (Scheme 2C).

## 2 Materials and methods

### 2.1 General information

All reactions were carried out in an argon atmosphere with dehydrated solvents under anhydrous conditions unless otherwise noted. Dehydrated THF and CH_2_Cl_2_ were purchased from Kanto Chemical Co., Inc., and the other solvents were dehydrated and distilled according to standard protocols. Reagents were obtained from commercial suppliers and used without further purification unless otherwise noted.

Reactions were monitored by thin-layer chromatography (TLC) on 0.25 mm Merck silica gel plates (60F-254). Column chromatography was performed using Silica Gel 60N (Kanto Chemical Co., Inc., spherical, neutral, particle size 63–210 mm) and NH-DM1020 (Fuji Silysia Chemical Ltd., spherical, particle size 100 μm); flash column chromatography was performed using Silica Gel 60N (Kanto Chemical Co., Inc., spherical, neutral, particle size 40–50 mm), unless otherwise noted.

Melting points were measured using a Yazawa BY-2 and Buchi M-565 and were uncorrected. Infrared (IR) spectra were obtained using a JASCO FT-IR-4600 instrument and are reported as wavenumbers. Proton nuclear magnetic resonance (^1^H-NMR) spectra were recorded using a JEOL JMN-AL400 (400 MHz) and a JEOL ECA-600 (600 MHz) spectrometer. Chemical shift (*δ*) is reported in parts per million (ppm) downfield relative to tetramethyl silane (TMS; 0.0 ppm) in CDCl_3_ and benzene (7.16 ppm) in C_6_D_6_. The coupling constants (*J*) are reported in Hz. Carbon-13 nuclear magnetic resonance (^13^C-NMR) spectra were recorded on a JEOL JMN-AL400 (100 MHz) spectrometer. Chemical shifts are reported in ppm relative to the centerline of the triplet of ^13^CDCl_3_ (77.0 ppm) and ^13^C_6_D_6_ (128.0 ppm). Low-resolution mass spectra (MS) were recorded on JEOL JMS-DX303, JMS-T100GC, and JEOL JMS-700 instruments. High-resolution mass spectra (HRMS) were recorded on JEOL JMS-T100GC and JEOL JMS-700 mass spectrometers using electron impact (EI) and on a Thermo Scientific Exactive Mass Spectrometer using electrospray ionization (ESI).

### 2.2 General procedure

#### 2.2.1 Synthesis of *cis*-3,4-epoxy amines (1aa–1ka, 1ab and 1ac)

Et_3_N (2.5 eq) and MsCl (1.5 eq) were added to a solution of epoxy alcohol (1 eq) in CH_2_Cl_2_ (0.5 M) at 0°C, and the mixture was stirred for 10 min at room temperature. Then, saturated aqueous NaHCO_3_ was added to the mixture at 0°C, and the mixture was extracted thrice with CH_2_Cl_2_. The combined organic layers were dried over anhydrous MgSO_4_, filtered, and concentrated under reduced pressure. The resulting crude product was used immediately in the subsequent reaction without further purification.

Alkyl amine (3.0 eq) and NaI (10 mol%) were added to a solution of the crude product in DMSO (0.5 M) at room temperature (ca. 25°C), and the mixture was stirred for 2 days at ambient temperature. The mixture was diluted with H_2_O and extracted with Et_2_O. The combined organic layers were washed thrice with brine, dried over anhydrous Na_2_SO_4_, filtered, and concentrated under reduced pressure. The resulting residue was purified using column chromatography to yield the corresponding epoxy amines.

#### 2.2.2 Synthesis of *cis*-3,4-epoxy anilines (1la–1na)

A pre-mixed solution of NaOCl·5H_2_O (1.5 eq) in saturated aqueous NaHCO was added dropwise to a cooled and well-stirred mixture of epoxy alcohol (1.0 eq) and TEMPO (1 mol%) in CH_2_Cl_2_ (0.2 M) and saturated aqueous NaHCO_3_ containing KBr (10 mol%), and the resulting mixture was stirred for 10 min at 0°C. Then, saturated aqueous Na_2_S_2_O_3_ was added at 0°C, and the mixture was extracted with CH_2_Cl_2_. The combined organic layers were washed with brine, dried over MgSO_4_, filtered, and concentrated under reduced pressure. The resulting crude product was used immediately in the subsequent reaction without purification (Lucio Anelli et al., 1987).

ArNH_2_ (1.0 eq) was added to a solution of the abovementioned crude product in CH_2_Cl_2_. After the mixture was stirred for 10 min at 0°C, NaBH(OAc)_3_ (1.2 eq) was added at 0°C and stirred at room temperature. Then saturated aqueous NaHCO_3_ was added, and the resulting mixture was extracted thrice with CH_2_Cl_2_. The combined organic layers were dried over anhydrous Na_2_SO_4_, filtered, and concentrated under reduced pressure. The resulting residue was purified using column chromatography to yield the corresponding epoxy anilines.

#### 2.2.3 Optimized conditions of intramolecular aminolysis of *cis*-3,4-epoxy amines

La(OTf)_3_ (5 mol%) was added to a solution of *cis*-3,4-epoxy amine (1 eq) in (CH_2_Cl)_2_ (0.2 M) at room temperature, and the mixture was stirred under reflux. Upon completion of the reaction, the mixture was cooled to 0°C, and saturated aqueous NaHCO_3_ was added. The mixture was extracted thrice with CH_2_Cl_2_. The combined organic layers were dried over Na_2_SO_4_, filtered, and then concentrated under reduced pressure. The resulting residue was purified using column chromatography to yield the corresponding azetidine.

## 3 Results

The feasibility of azetidine synthesis via Ln(OTf)_3_-catalyzed intramolecular aminolysis of *cis*-3,4-epoxy amines was investigated using *cis*-3,4-epoxy amine **1aa**, prepared from *cis*-3-hexen-1-ol in three steps, as a model substrate (Table 1). Preliminary experiments revealed the optimum conditions for the intramolecular aminolysis of *trans*-3,4-epoxy amines to yield pyrrolidine; catalytic La(OTf)_3_ in refluxing CH_2_Cl_2_ did not complete the reaction (Kuriyama et al., 2021). Therefore, 1,2-dichloroethane (DCE) was used for refluxing instead of CH_2_Cl_2_ for 2.5 h to afford azetidine **2aa** in 81% yield, along with a trace amount of pyrrolidine **3aa** (**2aa**/**3aa** = >20:1) (Table 1, entry 1). Solvents with almost the same boiling points similar to that of DCE were screened. The selectivity for benzene (PhH) was lower than that for DCE (Table 1, entry 2). Coordinative solvents such as MeCN and THF exhibited good selectivity, but some of the substrate **1aa** remained (Table 1, entries 3 and 4). Subsequently, the acids were screened using DCE as the solvent. Using Sc(OTf)_3_ instead of La(OTf)_3_ required a longer reaction time and afforded **2aa** in moderate yield (Table 1, entry 5). LiOTf afforded a complex mixture of products. Ni(ClO_4_)_2_·6H_2_O, the catalyst reported by Yamamoto for the intermolecular aminolysis of 3,4-epoxy alcohols (Wang and Yamamoto, 2015), and TfOH, a Brønsted acid, gave low yields of **2aa** because some substrate **1aa** remained; the reaction gave a complex mixture (Table 1, entries 7 and 8). In the absence of acids in refluxing DCE, no reaction occurred after 2.5 h (Table 1, entry 9). In contrast, although **1aa** was completely consumed after 24 h, **2aa** was not detected, and a complex mixture was obtained (Table 1, entry 10). Based on the aforementioned examination, the optimum conditions were identified as 5 mol% La(OTf)_3_ in refluxing DCE.

**TABLE 1:** Optimization of reaction conditions.

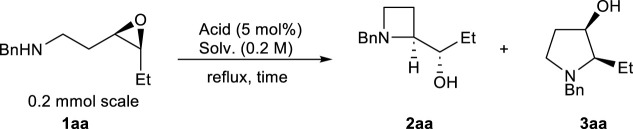				
Entry	Acid	Solv	NMR Yield of **2aa** ^a^ ^)^	**2aa**/**3aa**
1	La(OTf)_3_	(CH_2_Cl)_2_	81% (2.5 h)	>20:1
2	La(OTf)_3_	PhH	81% (2.5 h)	16:1
3	La(OTf)_3_	EtOAc	77% (12 h)	>20:1
4	La(OTf)_3_	MeCN	58% (12 h)	>20:1
5	Sc(OTf)_3_	(CH_2_Cl)_2_	62% (4.5 h)	>20:1
6	LiOTf	(CH_2_Cl)_2_	N.D. (6 h)	–
7	Ni(ClO_4_)_2_·6H_2_O	(CH_2_Cl)_2_	13% (6 h)	–
8	TfOH^b^	(CH_2_Cl)_2_	10% (6 h)	–
9	–	(CH_2_Cl)_2_	N.R. (2.5 h)	–
10	–	(CH_2_Cl)_2_	N.D. (24 h)	–

a NMR yield was determined using mesitylene.

b 15 mol%.

**FIGURE 1:**
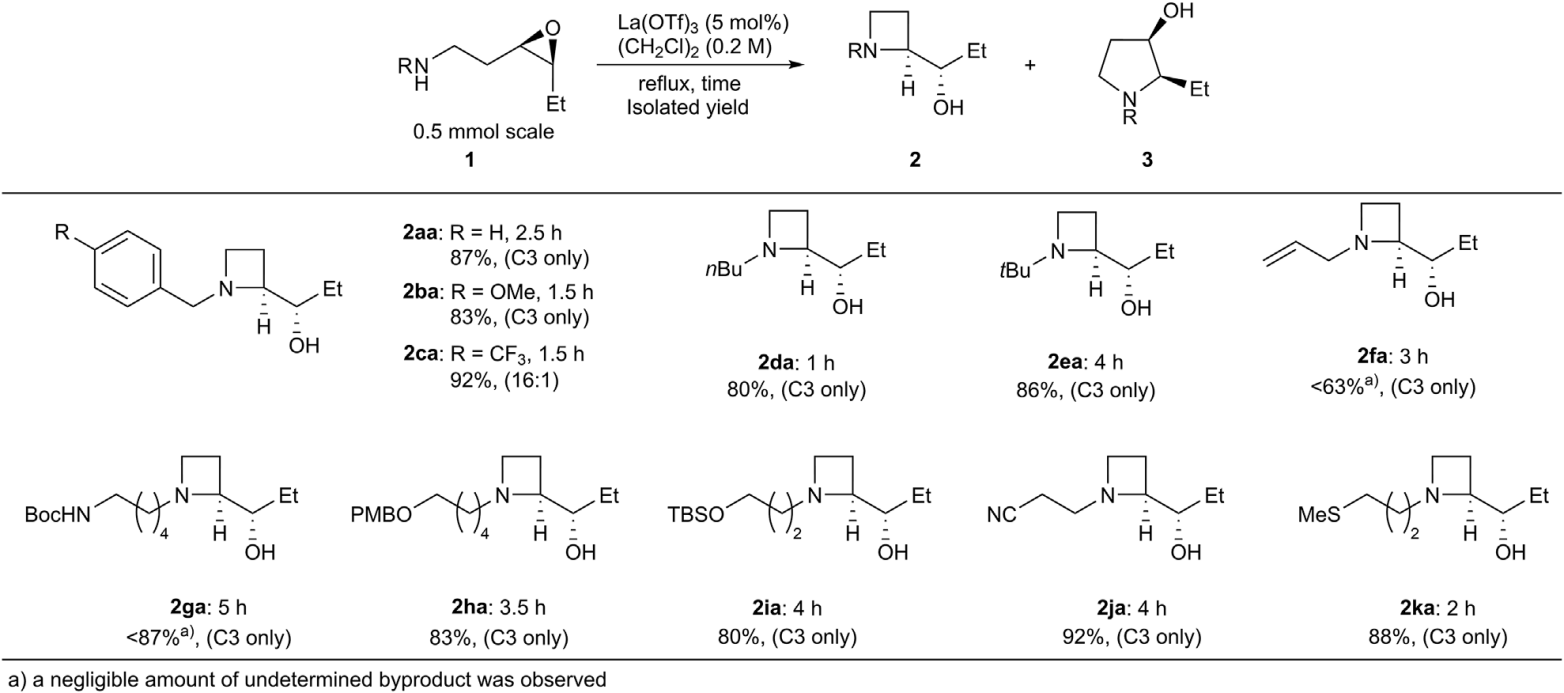
Substrate scope of the regioselective aminolysis.

**SCHEME 3:**
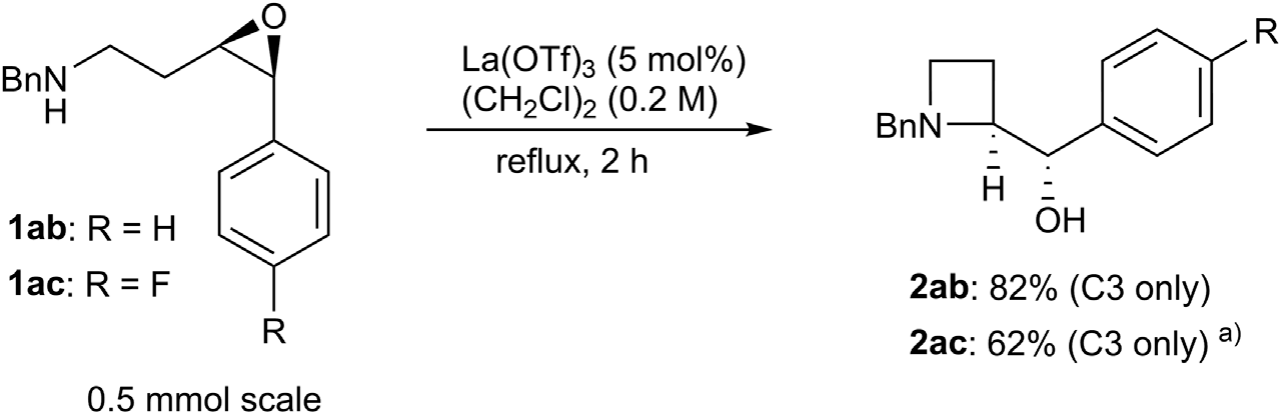
Aryl-substituted epoxy amine; a) A tiny amount of pyrrolidine was observed in the crude product (C3/C4 = >20:1).

With the optimum conditions established, the effects of the substituents on the amino groups were evaluated (Figure 1). Azetidine formation proceeded smoothly in the presence of electron-rich and electron-deficient benzyl groups (**2ba**, **2ca**). Substrates with an *n*-butyl amine moiety afforded azetidine **2da** in high yield with high regioselectivity. A substrate with a bulky *tert*-butyl amine afforded azetidine **2ea** in high yield. Substrates with π-basic allyl group also afforded the corresponding azetidine **2fa** in moderate yield. Acid-prone functional groups, such as Boc, PMB, and TBS groups, were tolerated to afford azetidines (**2ga**–**2ia**) in high yields. Nitrile and sulfide functionalities hardly affected the yields of azetidines (**2ja** and **2ka**). Interestingly, epoxy aniline **1la** gave azetidine **2la** in only 39% yield because of the competing formation of tetrahydroquinoline **4** via electrophilic aromatic substitution, whereas the corresponding *trans*-epoxy aniline efficiently underwent C4-selective intramolecular aminolysis to give pyrrolidine in high yield (Table 2) (Barvainiene et al., 2007; Wipf and Maciejewski, 2008; Mühlhaus et al., 2019). While epoxy aniline **1ma** bearing an electron-donating methoxy group underwent aminolysis as did **1la**, epoxy aniline **1na** bearing an electron-withdrawing nitro group did not undergo the reaction. The effect of the functional groups adjacent to the epoxide was evaluated. Apart from the cation stabilization at the benzylic position, phenyl-substituted epoxide **1ab** underwent aminolysis at the homobenzylic position, rather than the benzylic position, to afford azetidine **2ab** in high yields (Scheme 3). Azetidine **2ac** was also given in moderate yield from 4-fluorophenyl-substituted epoxide **1ac**.

**TABLE 2:** Substrate scope of epoxy anilines.

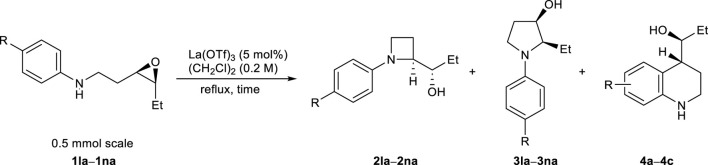				
R	Time (h)	Isolated yield		
		2	3	4
H (**1la**)	5	39%	19%	21%
MeO (**1ma**)	5	38%	<12%^a^	<29%^b^
NO_2_ ^c^ (**1na**)	7	N.R.		

a A negligible amount of undetermined byproduct was contaminated.

b Isomers of tetrahydroquinoline were obtained as an inseparable mixture.

c 0.25 mmol scale.

## 4 Discussion

**FIGURE 2:**
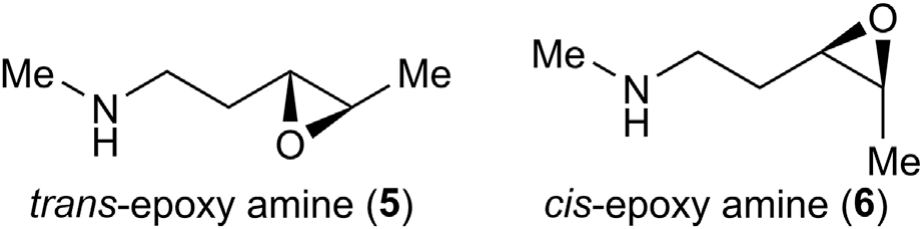
Substrates studied computationally.

**FIGURE 3:**
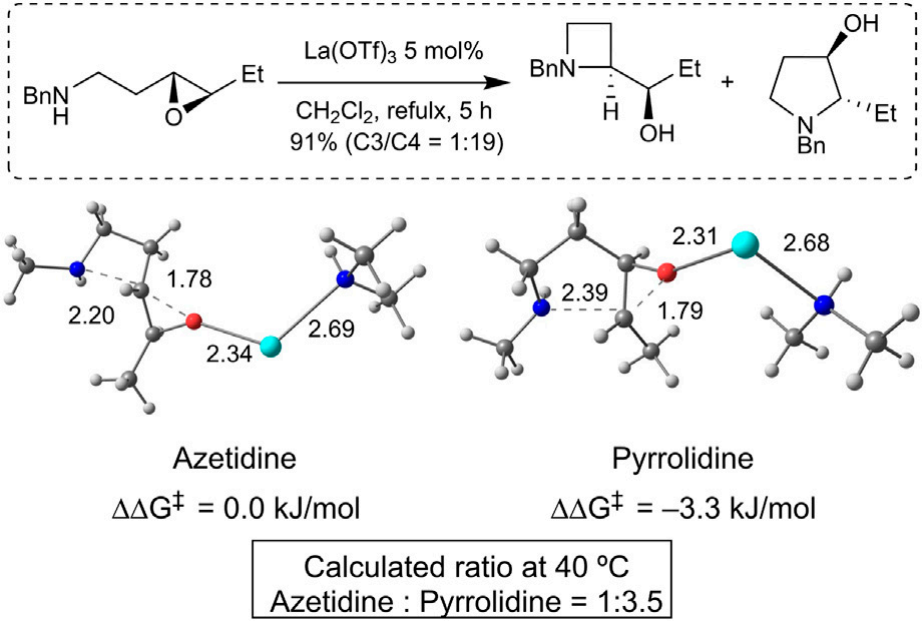
DFT studies of the selectivity for *trans*-epoxy amine (PCM (dichloromethane)/ωB97XD/6-311++G**, SDD (La)//PCM (dichloromethane)/ωB97XD/6-31G**, LanL2DZ (La)); lanthanum (light blue), oxygen (red), nitrogen (blue), carbon (gray), and hydrogen (white).

**FIGURE 4:**
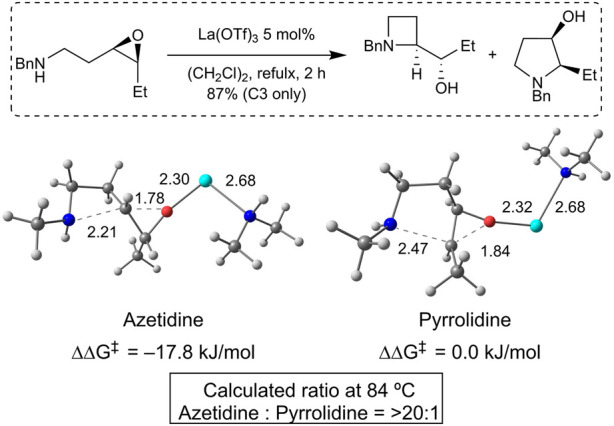
DFT studies of the selectivity for *cis*-epoxy amine (PCM (dichloroethane)/ωB97XD/6-311++G**, SDD (La)//PCM (dichloroethane)/ωB97XD/6-31G**, LanL2DZ (La)); lanthanum (light blue), oxygen (red), nitrogen (blue), carbon (gray), and hydrogen (white).

Density functional theory (DFT) calculations were performed to gain insight into the opposite regioselectivity between *trans*- and *cis*-epoxy amines. Simplified *trans*- and *cis*-epoxy amines **5** and **6** were used as substrates to reduce computational costs (Figure 2). When naked lanthanum (III) is coordinated to *trans*-epoxy amine **5**, the energy of the transition state that yields azetidine by C3-selective aminolysis is lower than that produced by C4-selective aminolysis, which indicates selectivity opposite to that of the experimental results (see Supplementary Material S1). Dimethylamine-coordinated lanthanum (III) was used as the activator. The calculations showed that the energy of the pyrrolidine transition state was lower than that of the azetidine transition state, which was consistent with the experimental results (Figure 3). Calculations of the transition states of *cis*-epoxy amines complexed with dimethylamine-coordinated lanthanum (III) showed that the transition state of azetidine was much smaller than that of pyrrolidine, which was consistent with the experimental results (Figure 4). These computational results suggest that lanthanum complexes coordinated by substrates and/or products are likely to contribute to inverse regioselectivity.

## 5 Conclusion

We have developed the La(OTf)_3_-catalyzed regioselective intramolecular aminolysis of *cis*-3,4-epoxy amines to afford azetidines. This reaction tolerated various functional groups, including coordinative and acid-prone functional groups. C3-selective aminolysis also proceeded with styrene oxide-type 3,4-epoxy amine, in which the C4 position was the benzylic position. Computational studies suggest that the difference in the regioselectivity of aminolysis between the *cis*- and *trans*-isomers was likely caused by lanthanum (III) coordinated with the substrate and/or product. Further investigations of the Lewis acid-promoted ring-opening reaction of strained heterocycles and its application to successive ring-opening reactions are currently underway. The reactions developed herein are expected to be applied to the synthesis of various highly functionalized bioactive compounds.

## Data Availability

The original contributions presented in the study are included in the article/Supplementary Materials, further inquiries can be directed to the corresponding author.
