# Supplementary material for: Azetidine synthesis by La(OTf)3-catalyzed intramolecular regioselective aminolysis of cis-3,4-epoxy amines
Source: Front Chem. 2023 Sep 19;11:1251299. doi: 10.3389/fchem.2023.1251299 (PMC10546187; doi:10.3389/fchem.2023.1251299)

N-benzyl-2-((2*R**, 3*S**)-3-ethyloxiran-2-yl) ethan-1-amine

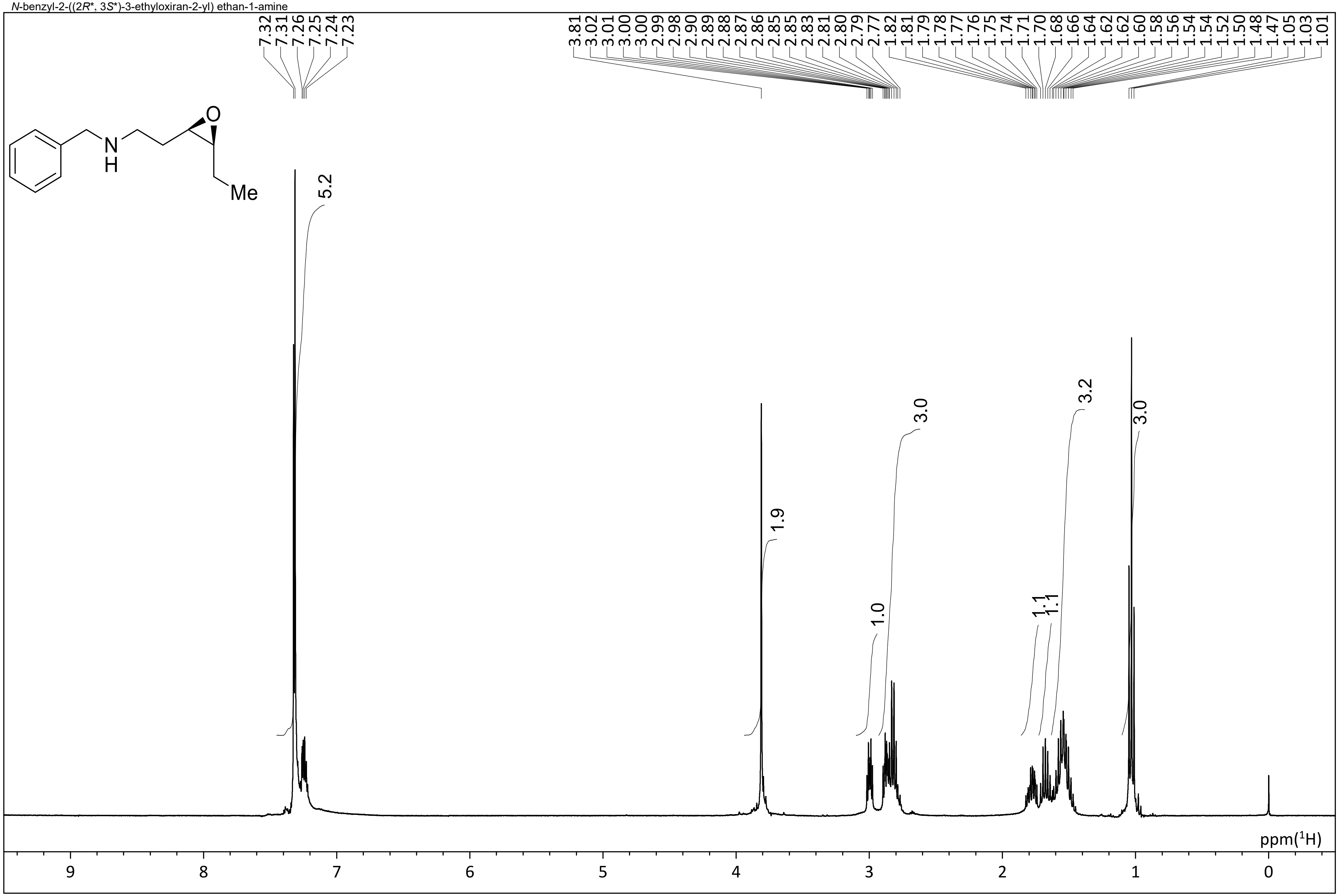

N-benzyl-2-((2*R**, 3*S**)-3-ethyloxiran-2-yl) ethan-1-amine

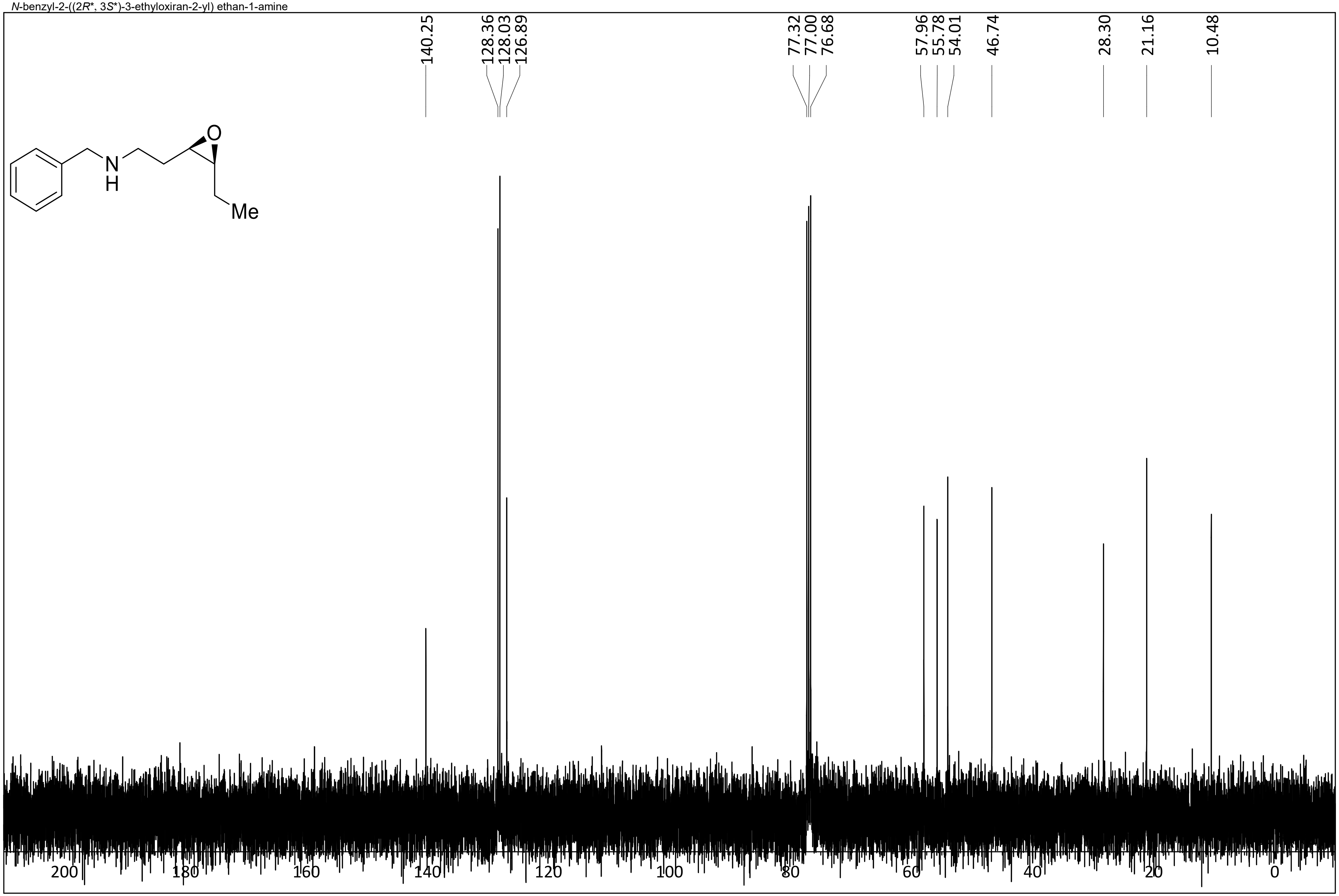

2-((2*R**, 3*S**)-3-ethyloxiran-2-yl)-*N*-(4-methoxybenzyl)ethan-1-amine

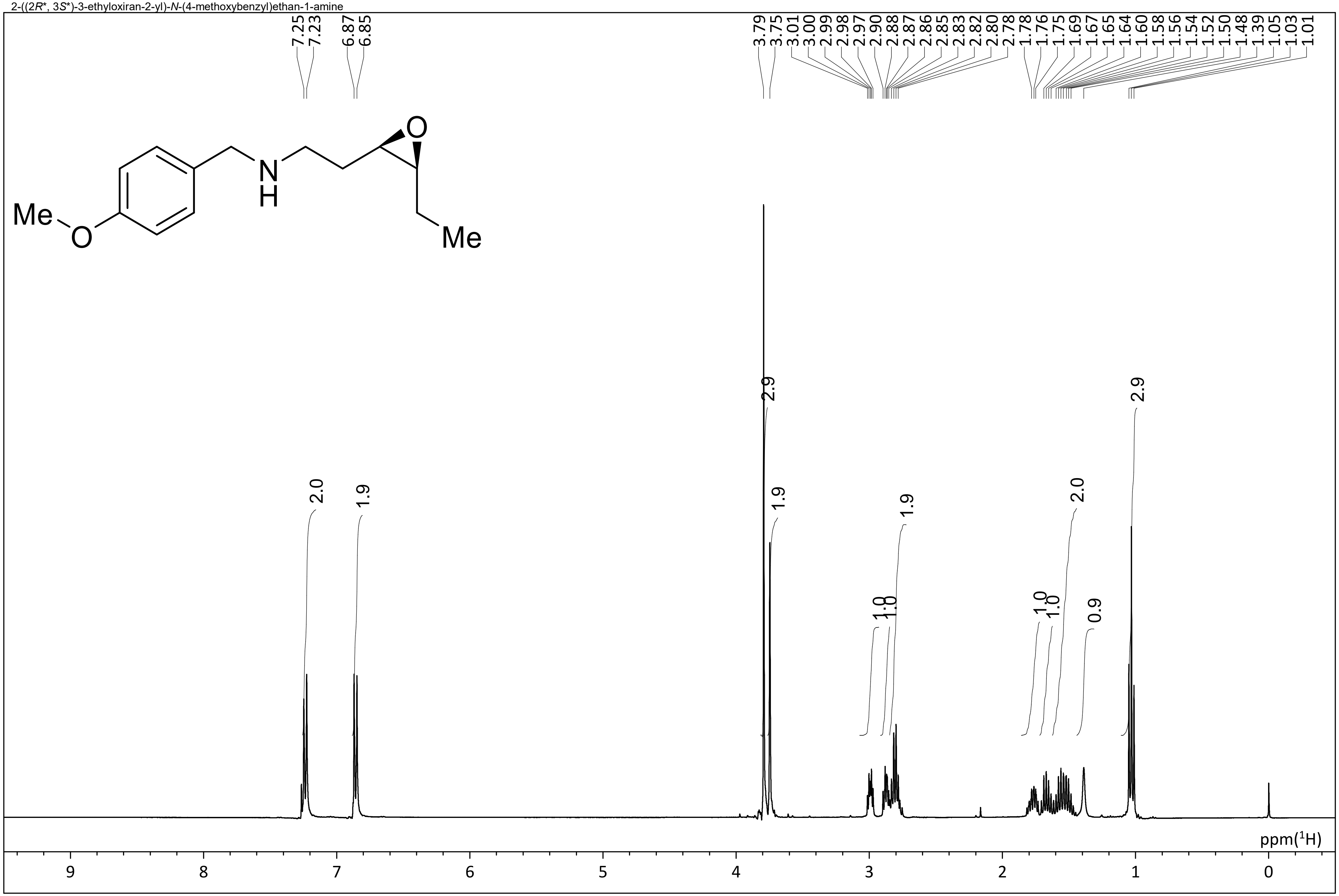

2-((2*R**, 3*S**)-3-ethyloxiran-2-yl)-*N*-(4-methoxybenzyl) ethan-1-amine

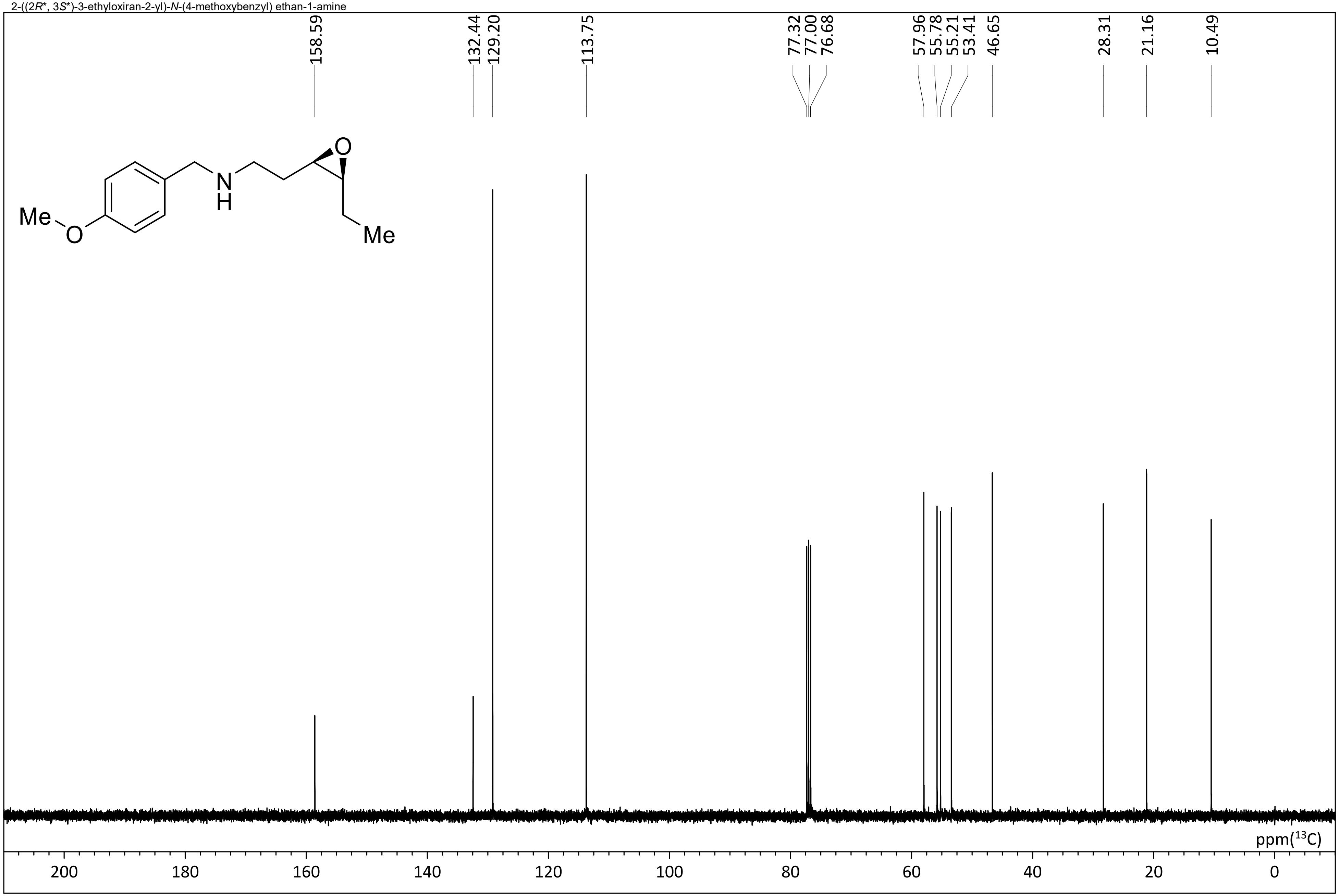

2-((2*R**, 3*S**)-3-ethyloxiran-2-yl)-*N*-(4-(trifluoromethyl) benzyl) ethan-1-amine

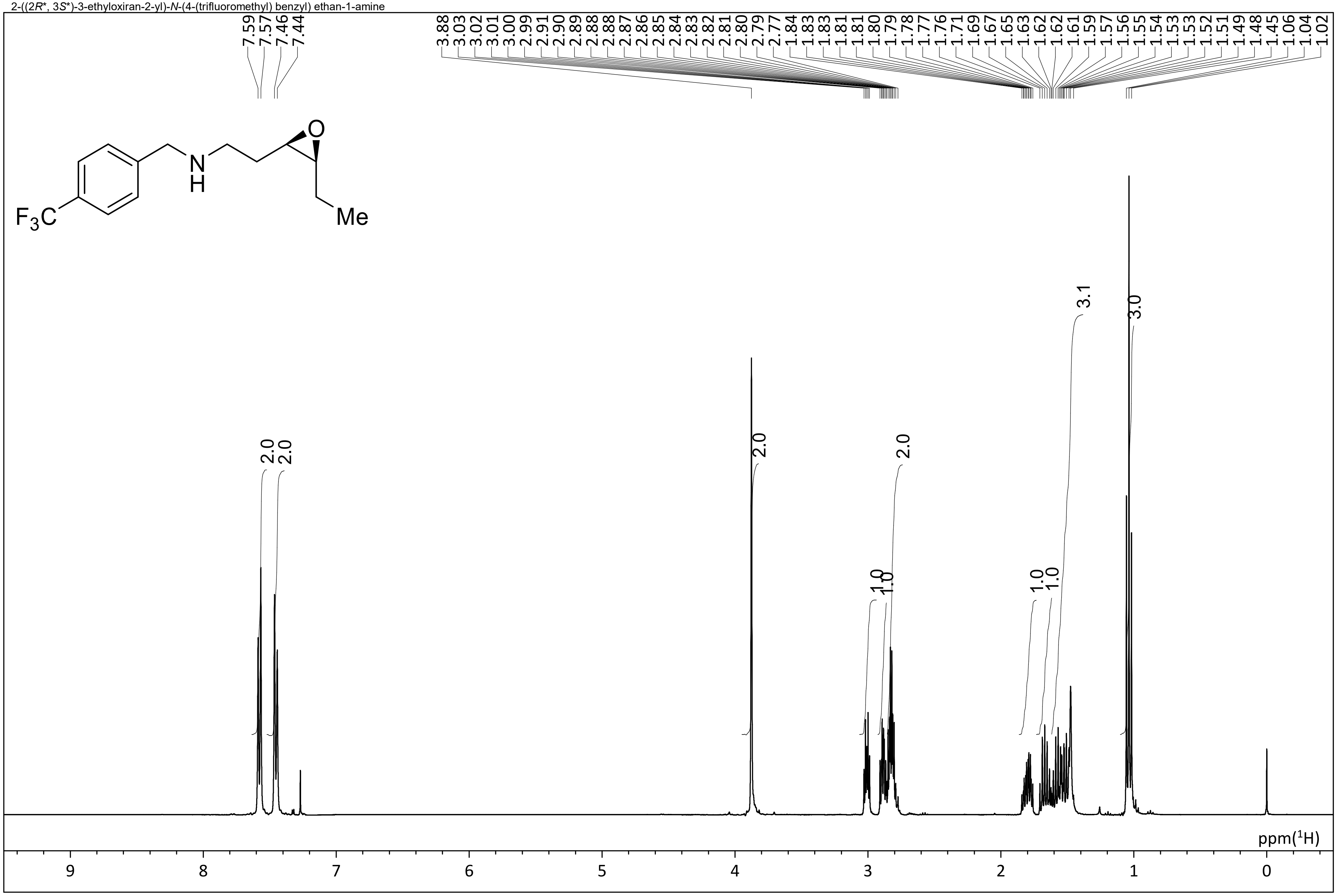

2-((2*R**, 3*S**)-3-ethyloxiran-2-yl)-*N*-(4-(trifluoromethyl) benzyl) ethan-1-amine

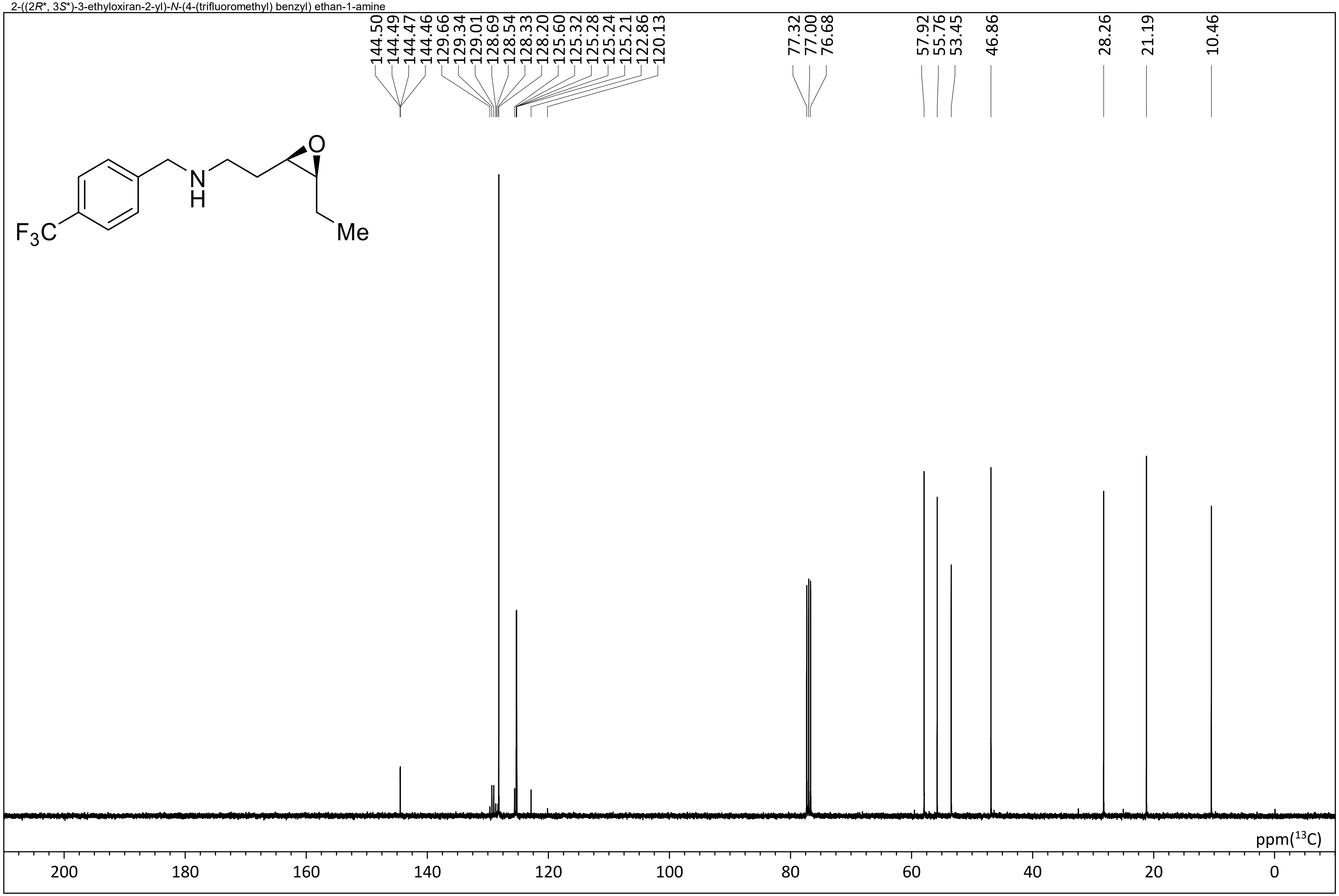

N-(2-((2*R**, 3*S**)-3-ethyloxiran-2-yl) ethyl) butan-1-amine

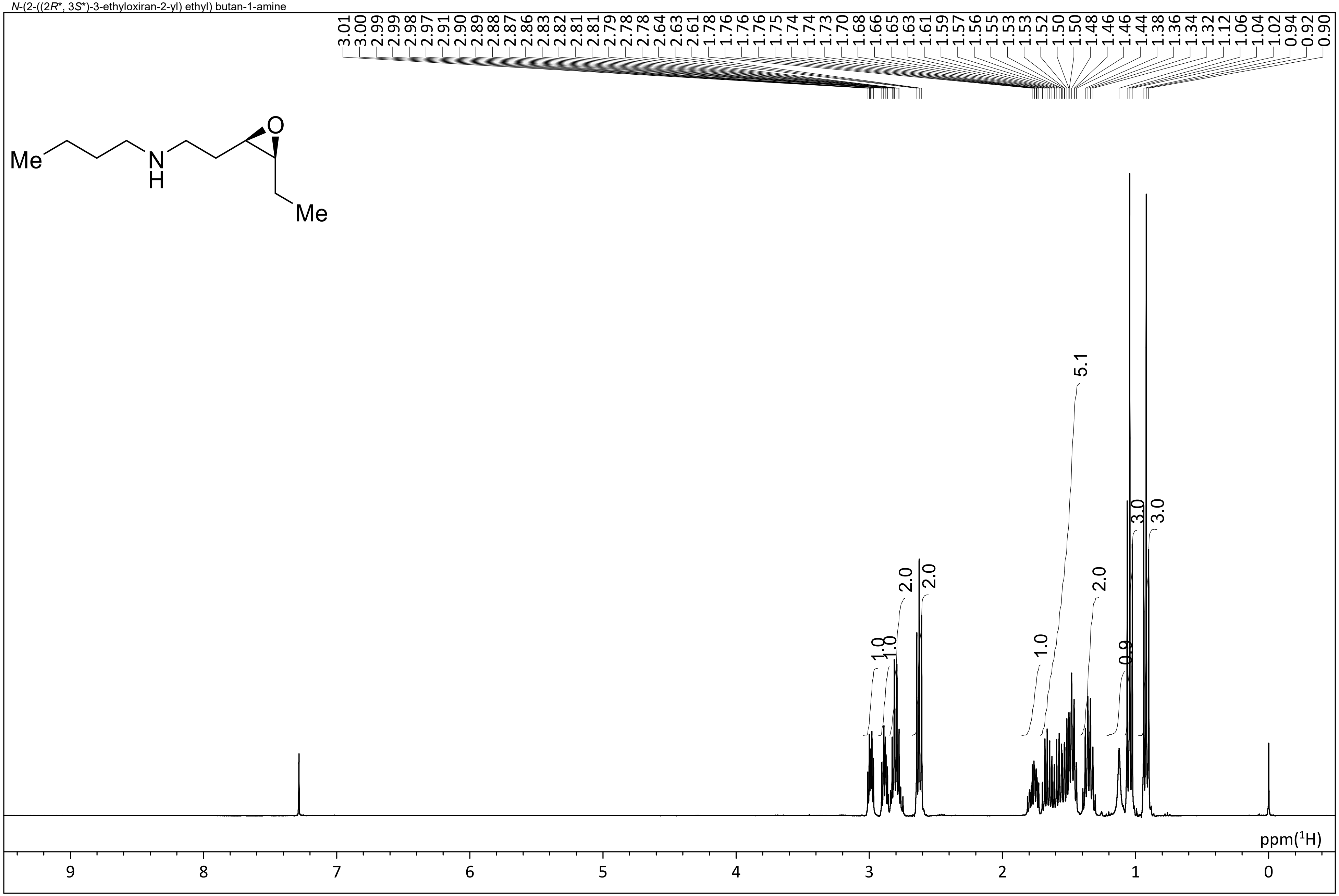

N-(2-((2*R**, 3*S**)-3-ethyloxiran-2-yl) ethyl) butan-1-amine

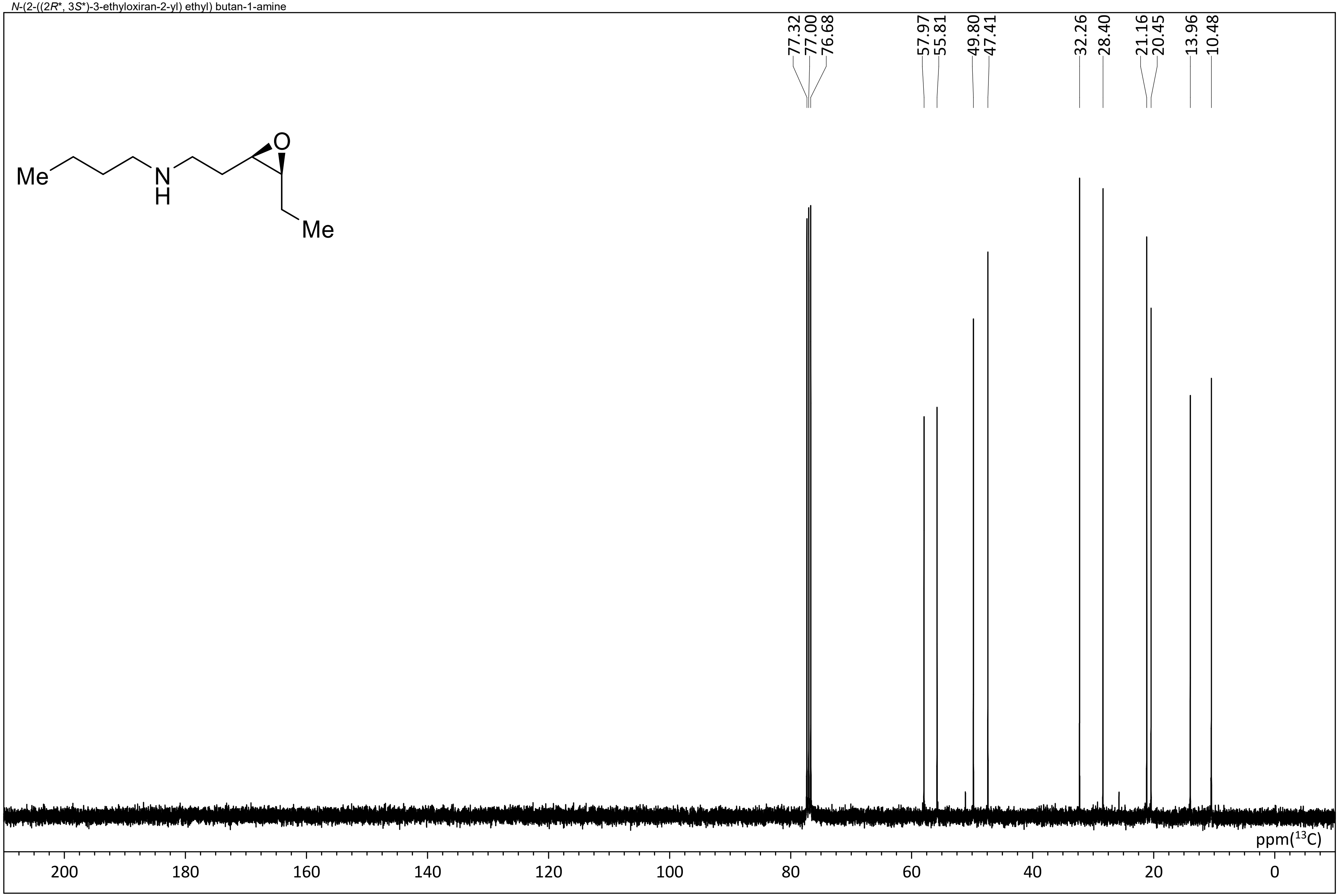

N-(2-((2*R**, 3*S**)-3-ethyloxiran-2-yl) ethyl)-2-methylpropan-2-amine

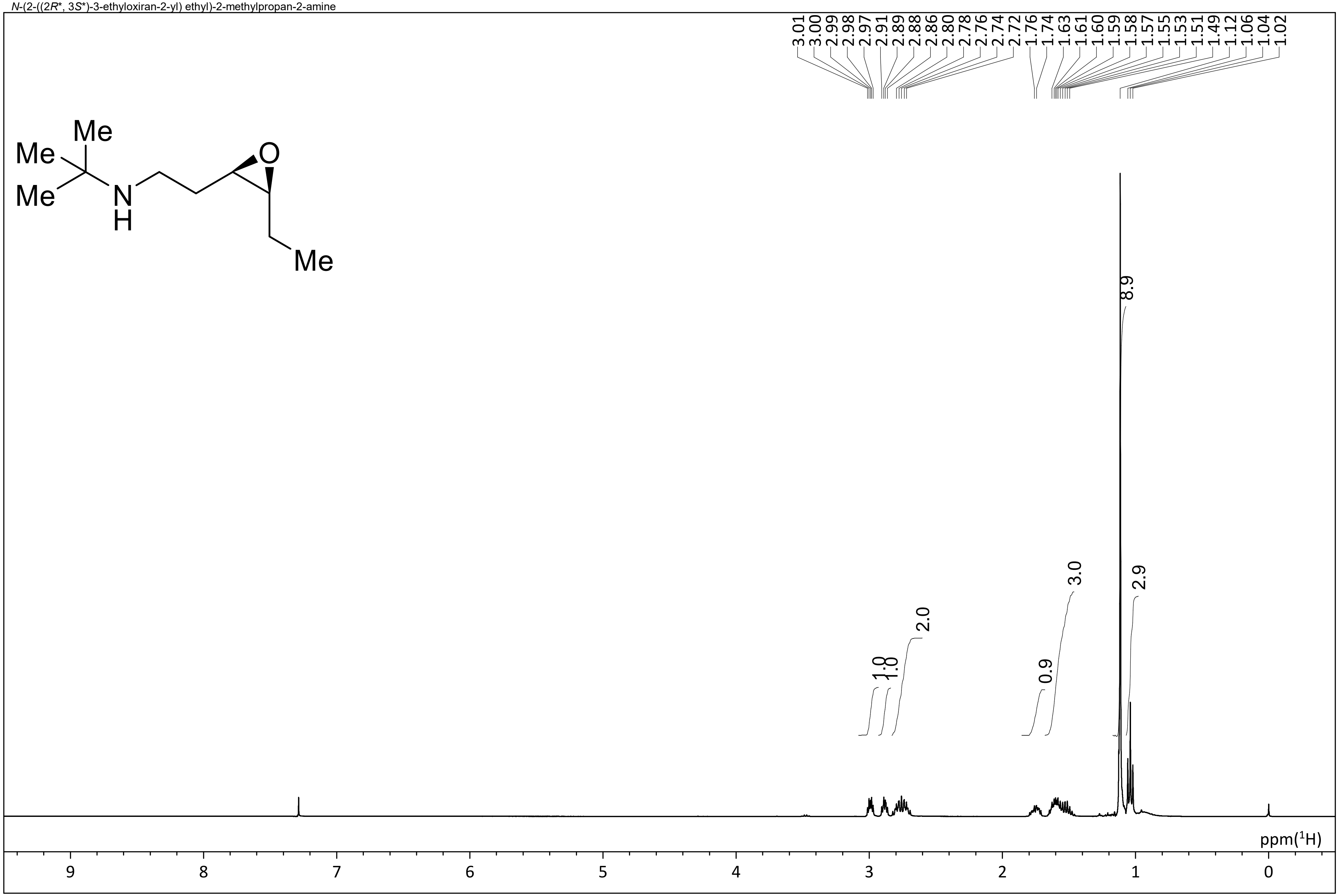

N-(2-((2*R**, 3*S**)-3-ethyloxiran-2-yl) ethyl)-2-methylpropan-2-amine

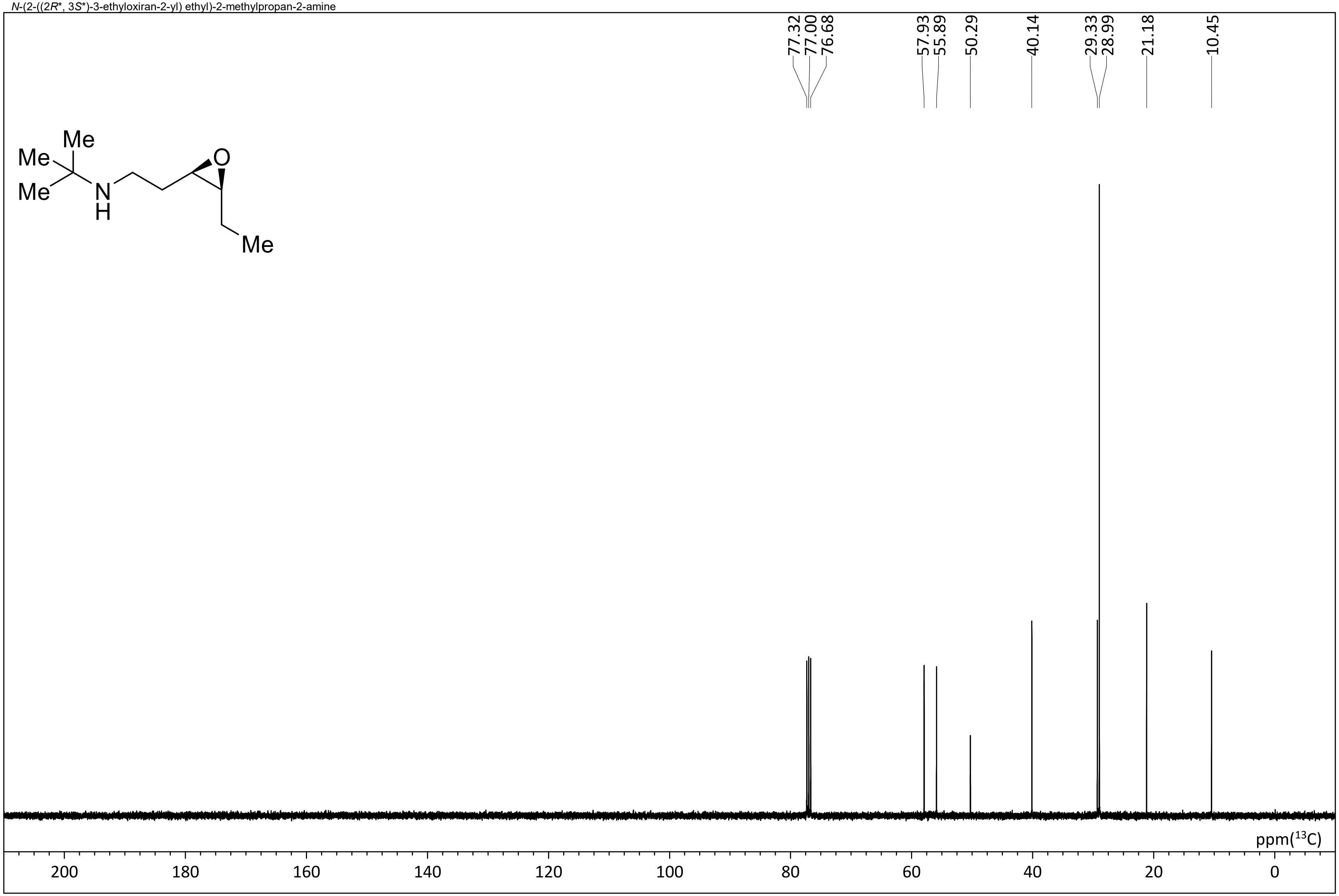

N-(2-((2*R**, 3*S**)-3-ethyloxiran-2-yl) ethyl) prop-2-en-1-amine

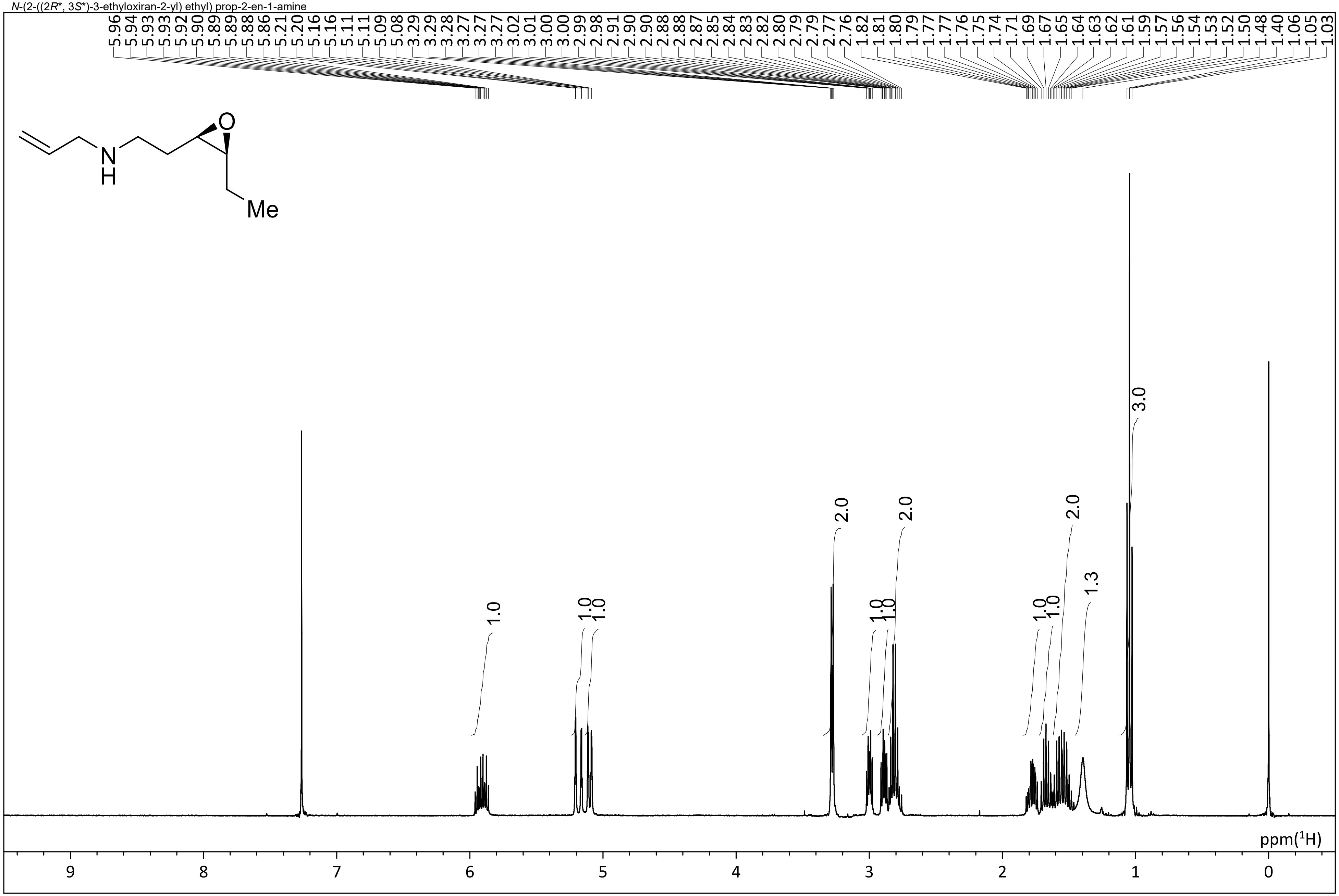

N-(2-((2*R**, 3*S**)-3-ethyloxiran-2-yl) ethyl) prop-2-en-1-amine

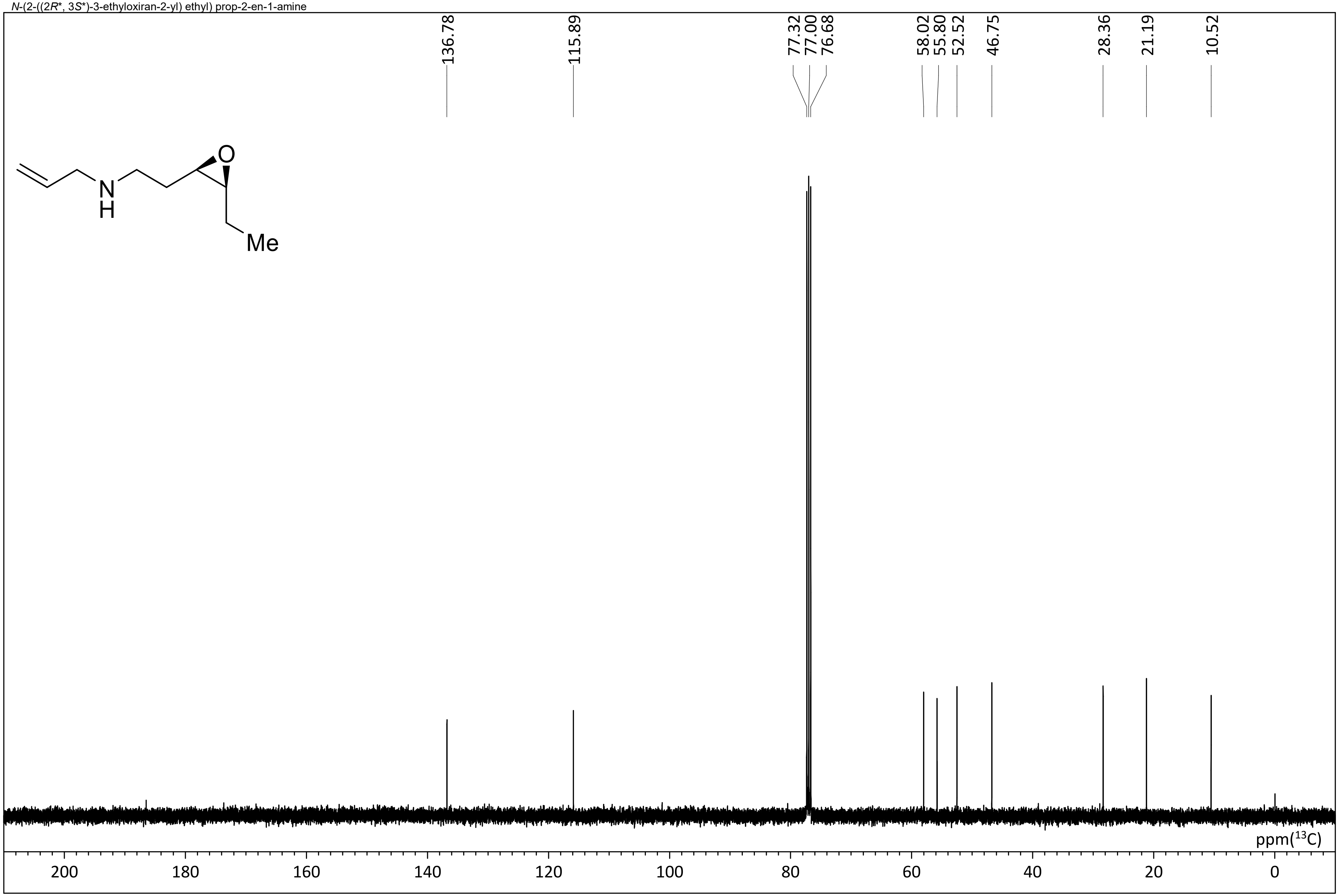

tert-butyl (5-((2-((2*R**, 3*S**)-3-ethyloxiran-2-yl)ethyl)amino)pentyl)carbamate

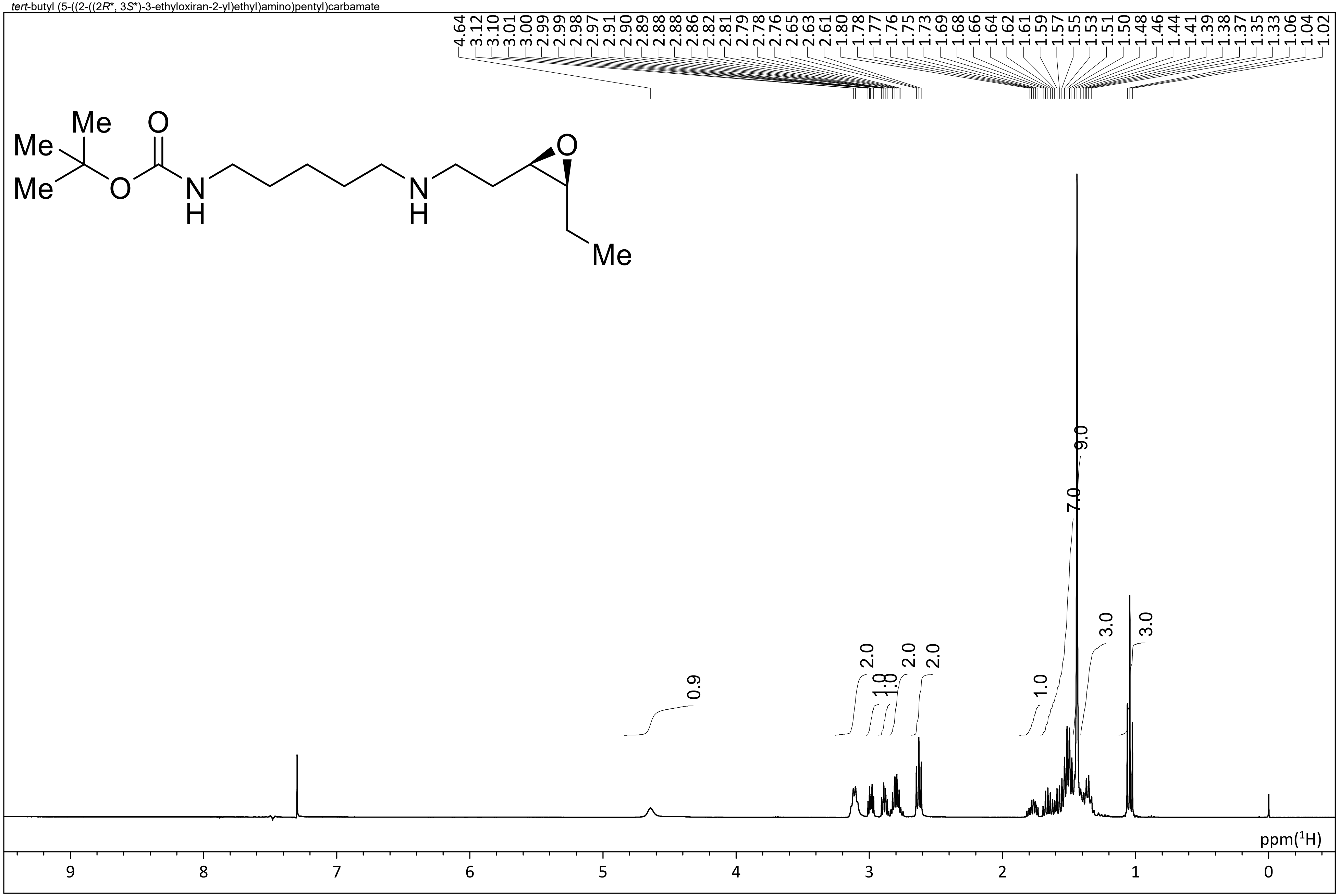

tert-butyl (5-((2-((2*R**, 3*S**)-3-ethyloxiran-2-yl)ethyl)amino)pentyl)carbamate

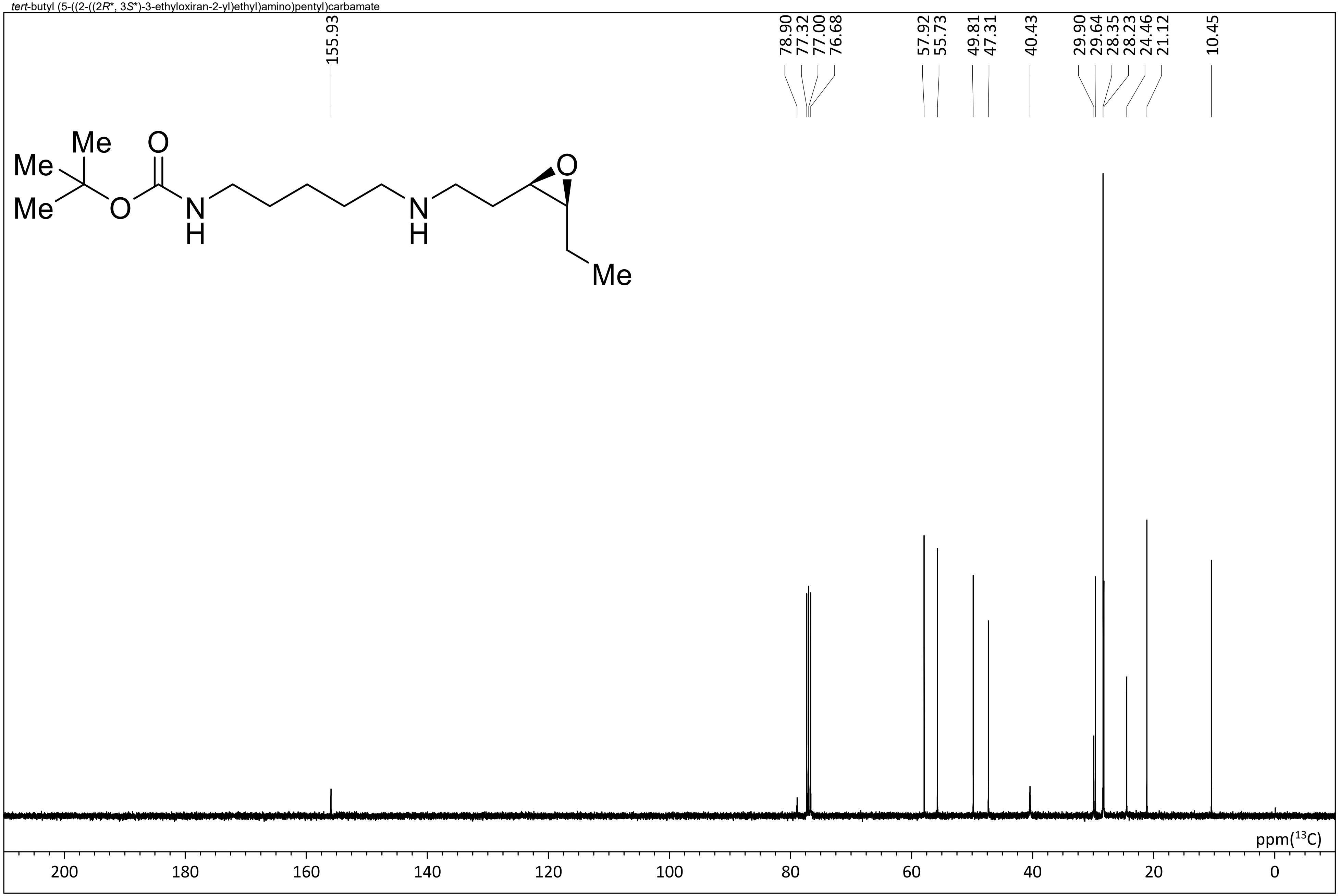

N-(2-((2*R**, 3*S**)-3-ethyloxiran-2-yl)ethyl)-5-((4-methoxybenzyl)oxy)pentan-1-amine

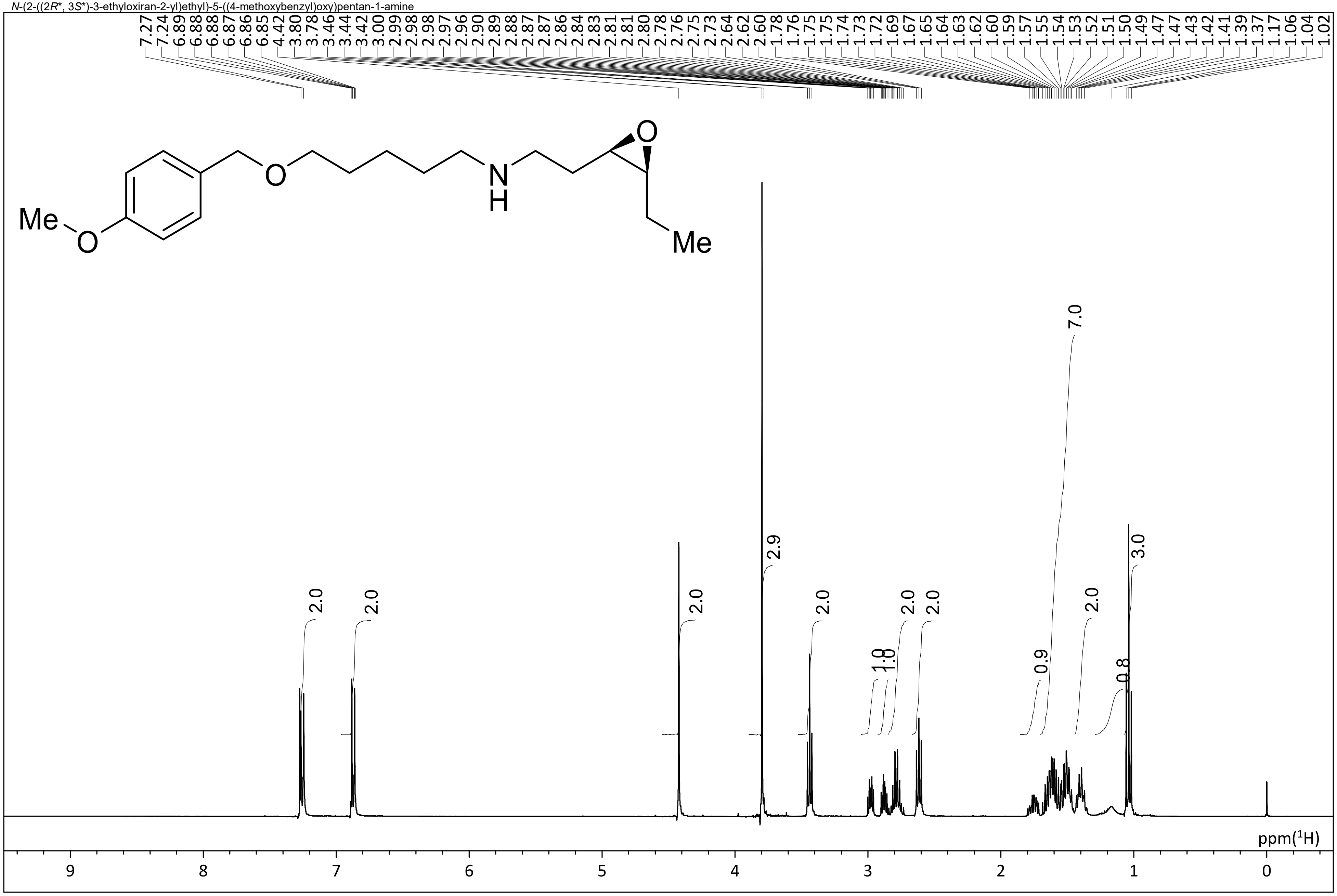

N-(2-((2*R**, 3*S**)-3-ethyloxiran-2-yl)ethyl)-5-((4-methoxybenzyl)oxy)pentan-1-amine

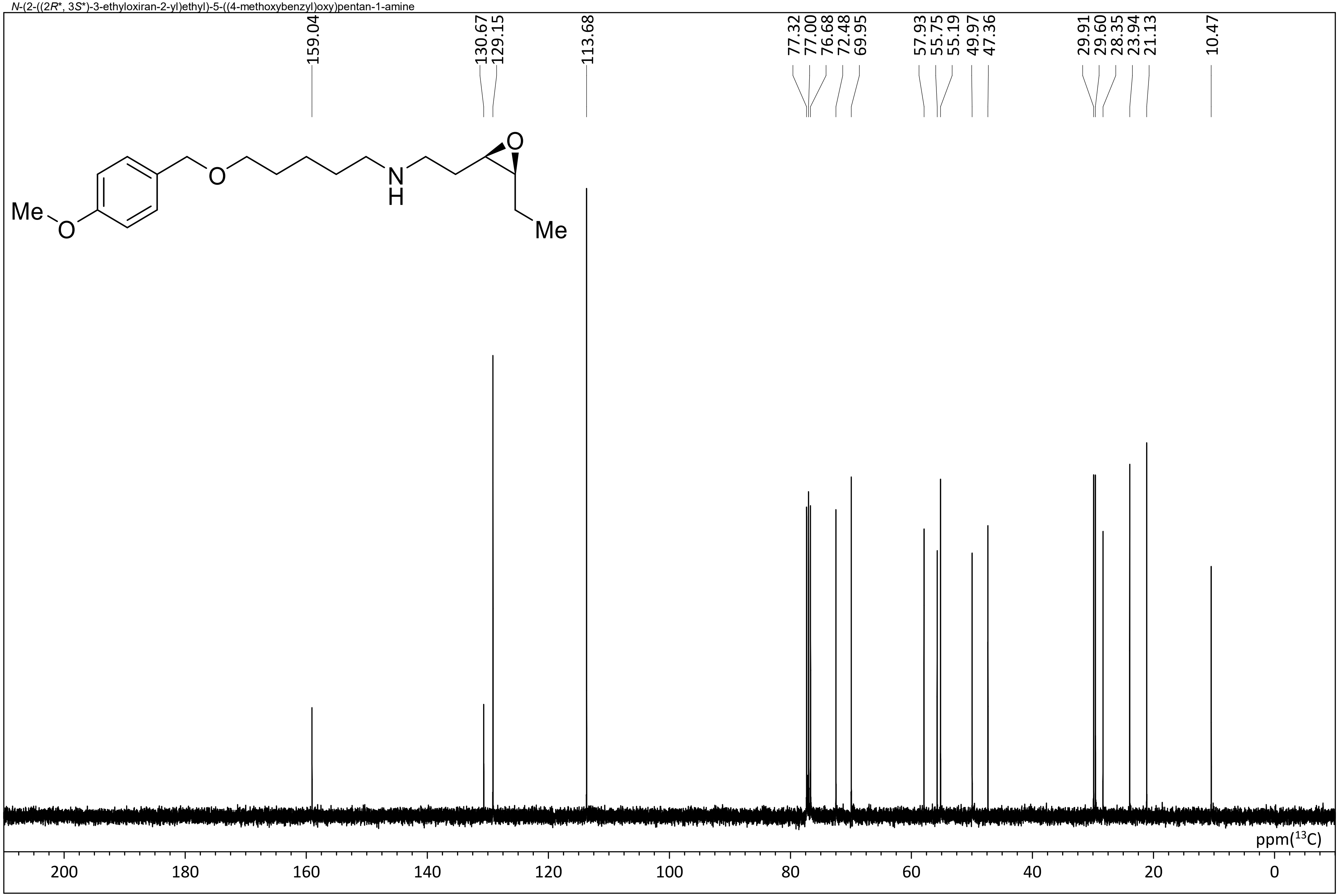

3-((*tert*-butyldimethylsilyl)oxy)-*N*-(2-((2*R**, 3*S**)-3-ethyloxiran-2-yl)ethyl)propan-1-amine

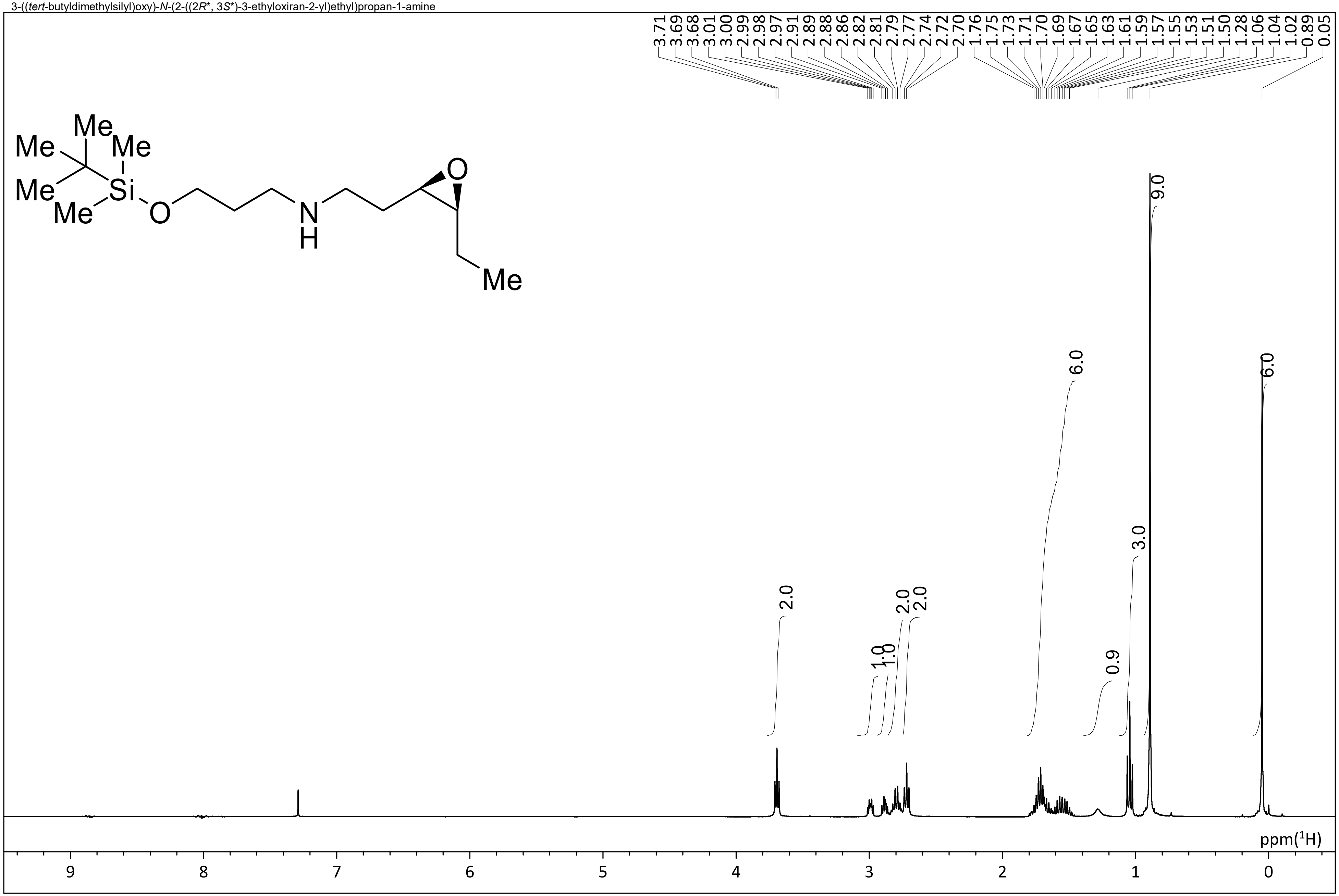

3-((*tert*-butyldimethylsilyl)oxy)-*N*-(2-((2*R**, 3*S**)-3-ethyloxiran-2-yl)ethyl)propan-1-amine

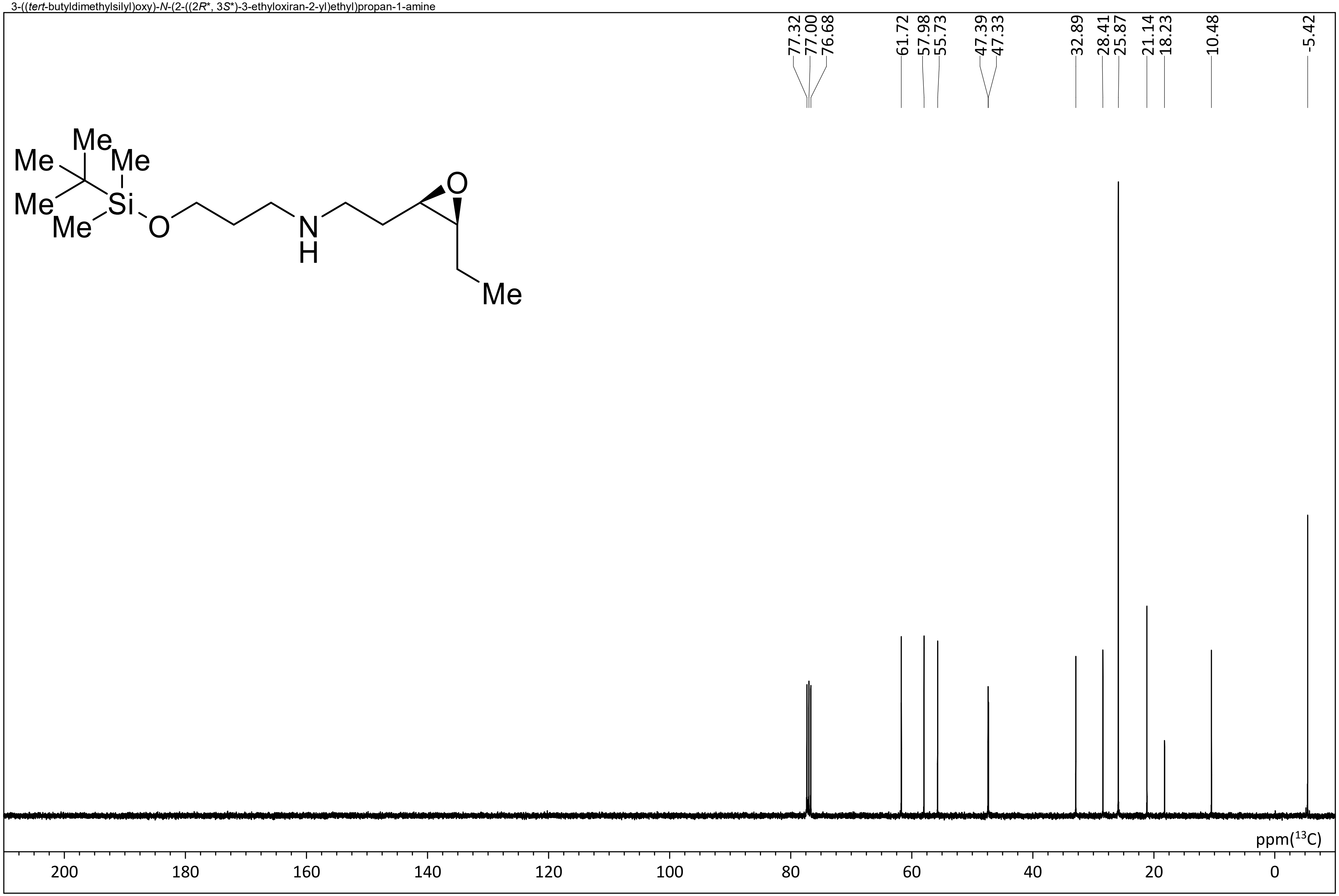

3-((2-((2*R**, 3*S**)-3-ethyloxiran-2-yl) ethyl) amino) propanenitrile

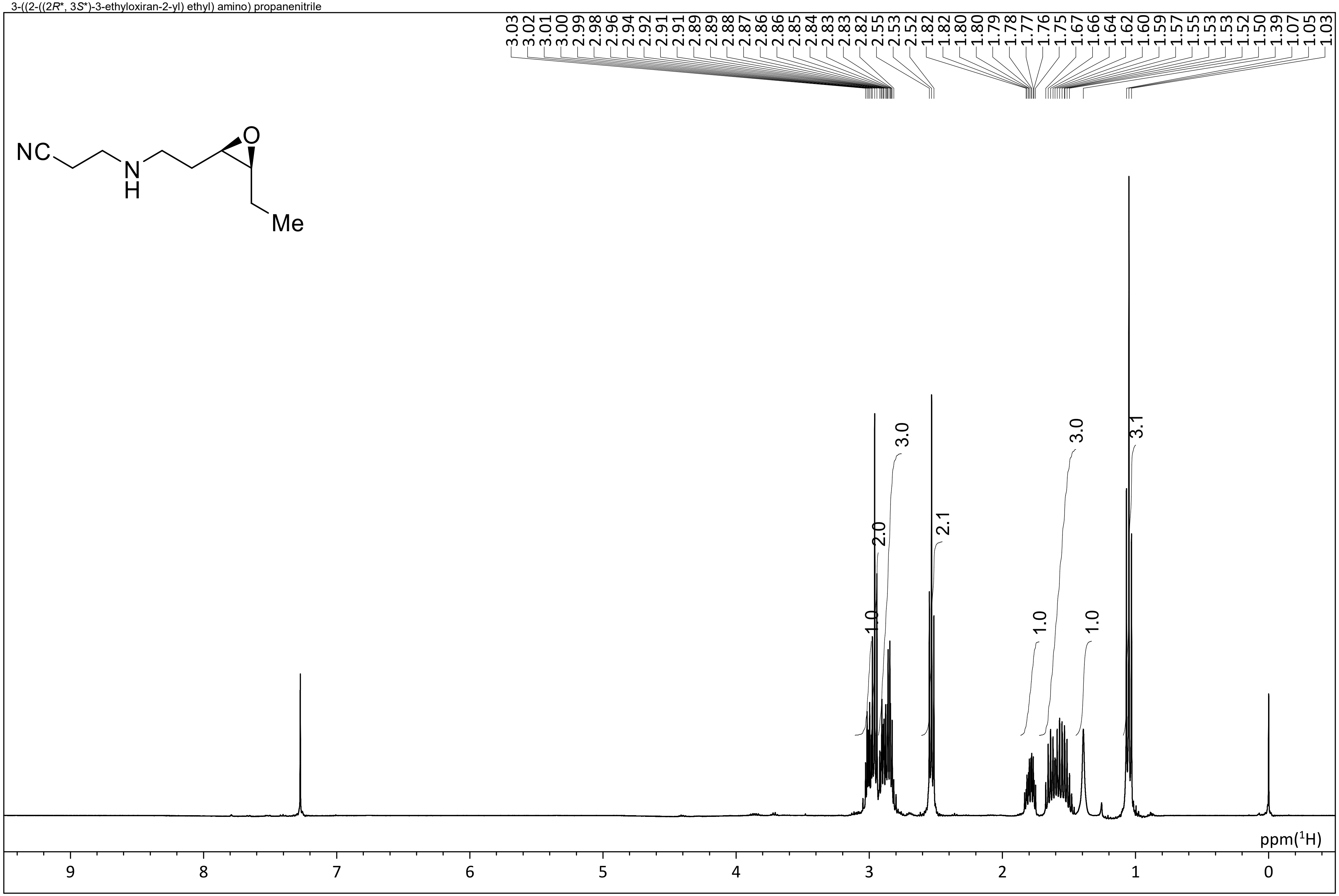

3-((2-((2*R**, 3*S**)-3-ethyloxiran-2-yl) ethyl) amino) propanenitrile

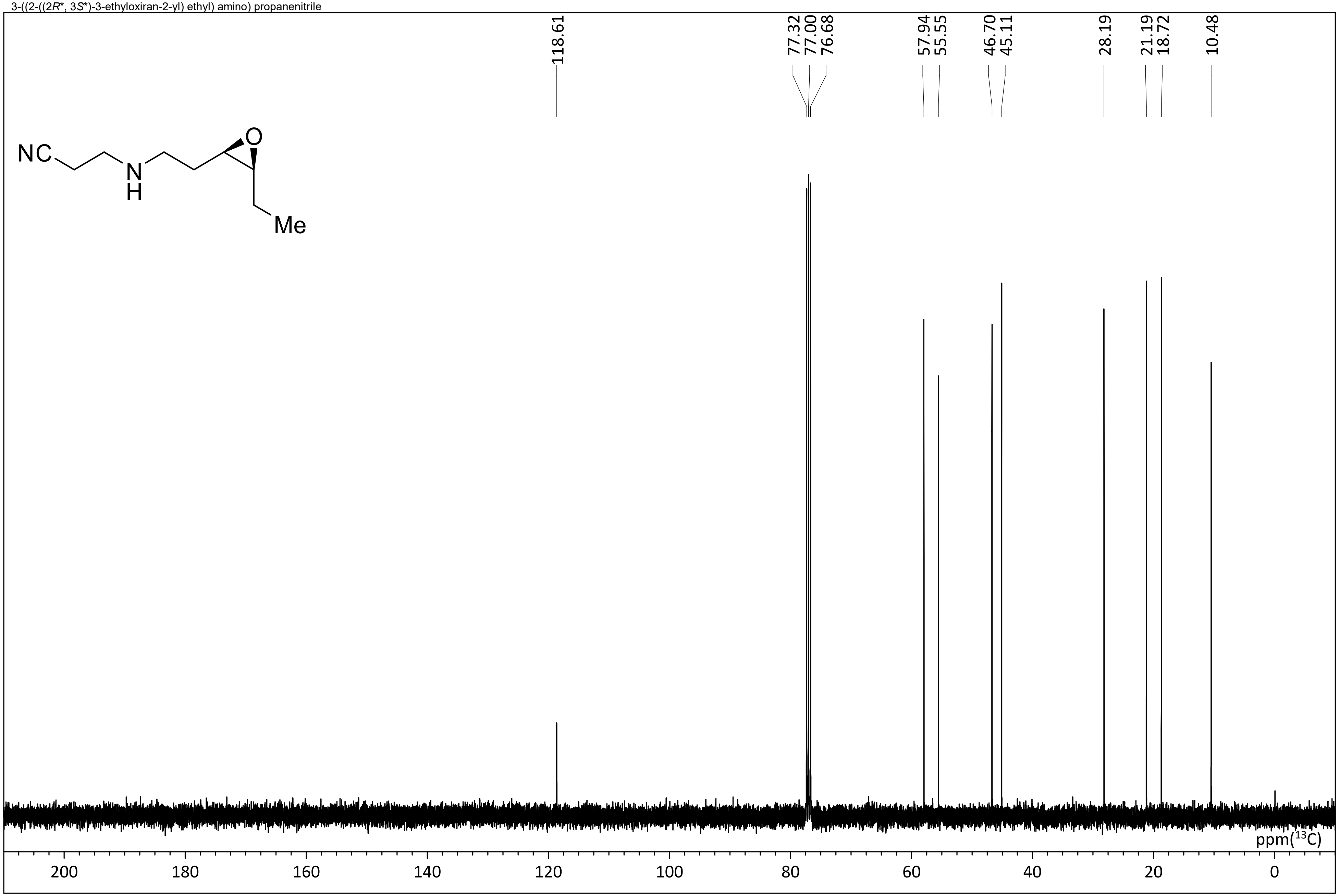

N-(2-((2*R**, 3*S**)-3-ethyloxiran-2-yl) ethyl)-3-(methylthio) propan-1-amine

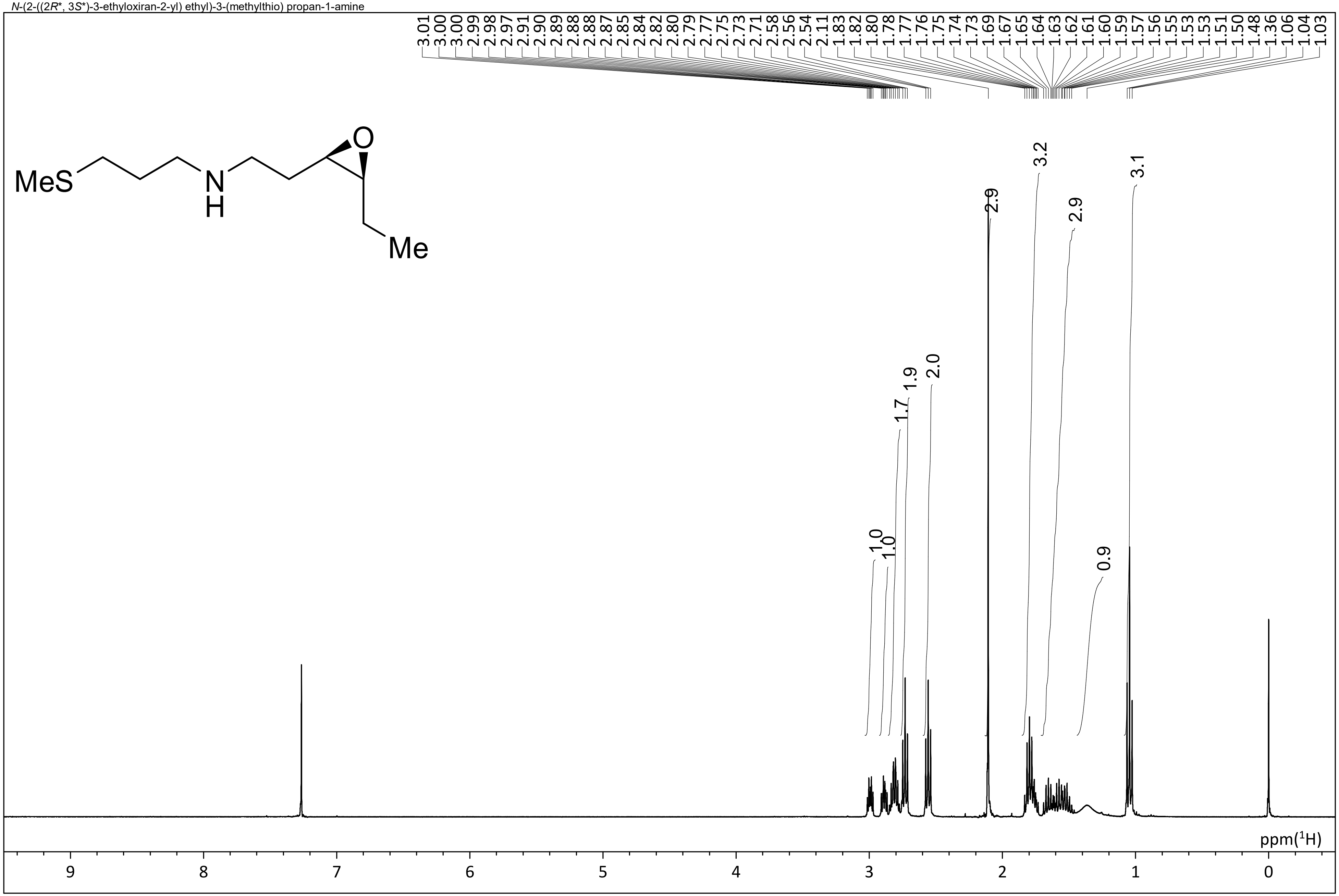

N-(2-((2*R**, 3*S**)-3-ethyloxiran-2-yl) ethyl)-3-(methylthio) propan-1-amine

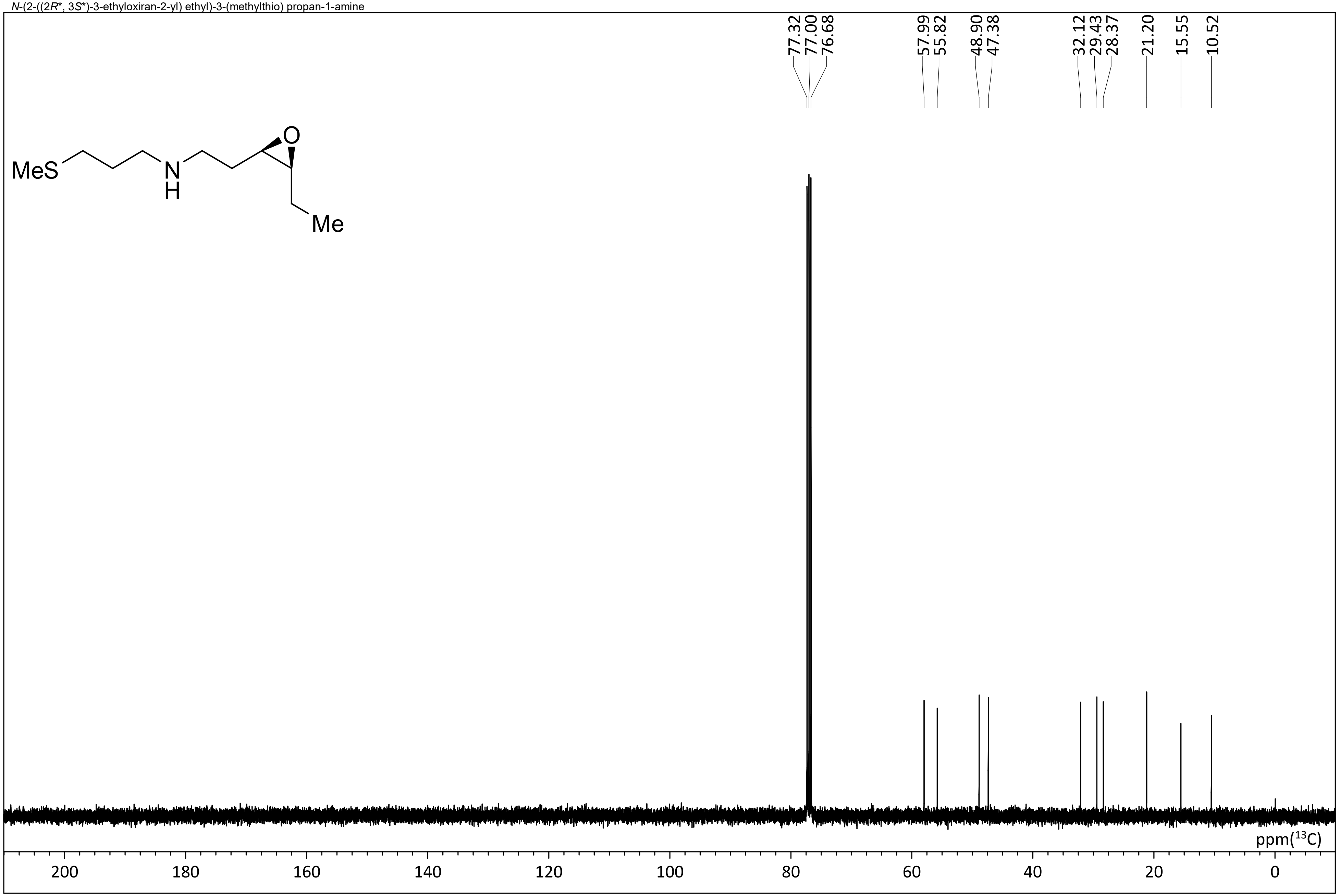

N-(2-((2*R**, 3*S**)-3-ethyloxiran-2-yl)ethyl)aniline

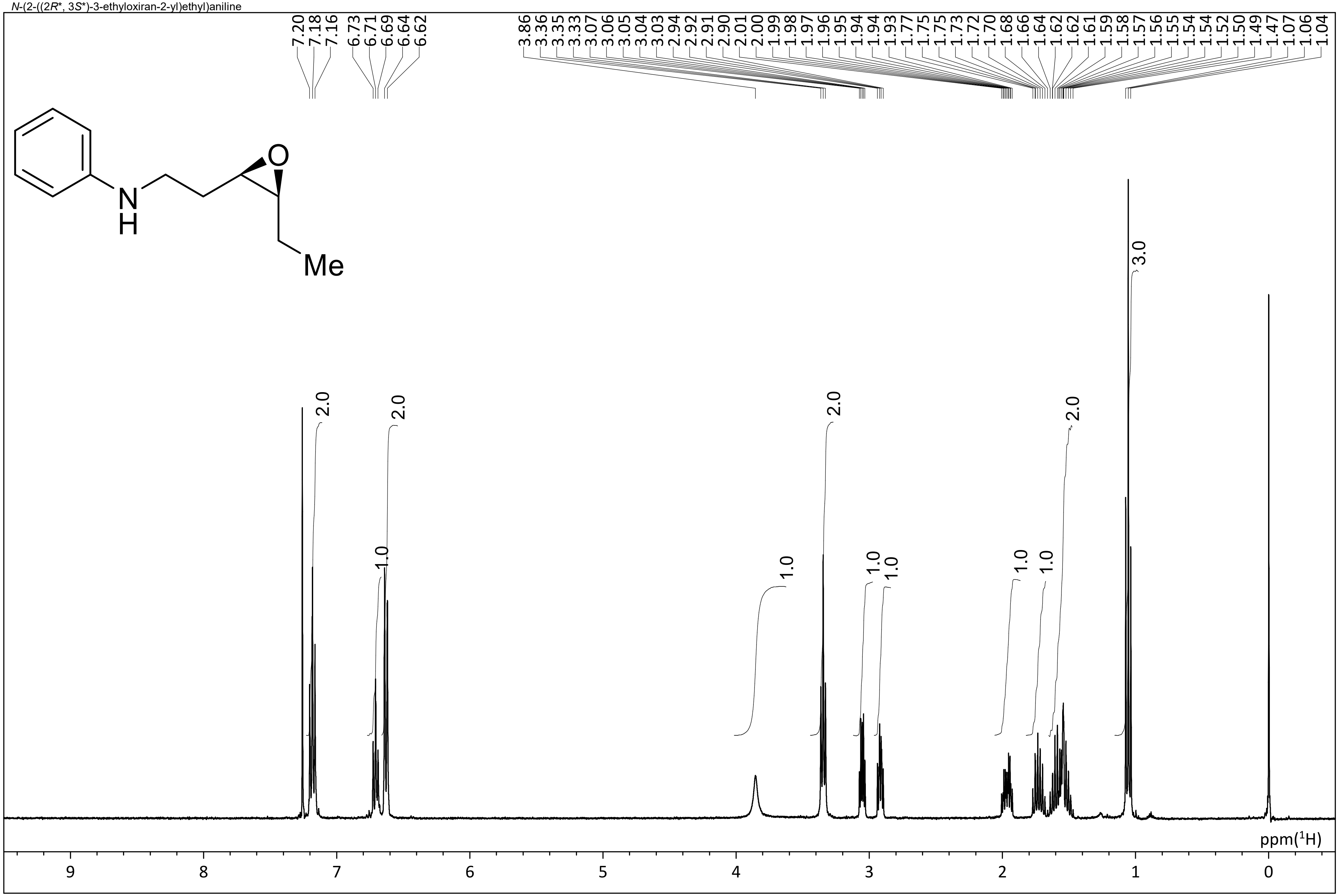

N-(2-((2*R**, 3*S**)-3-ethyloxiran-2-yl)ethyl)aniline

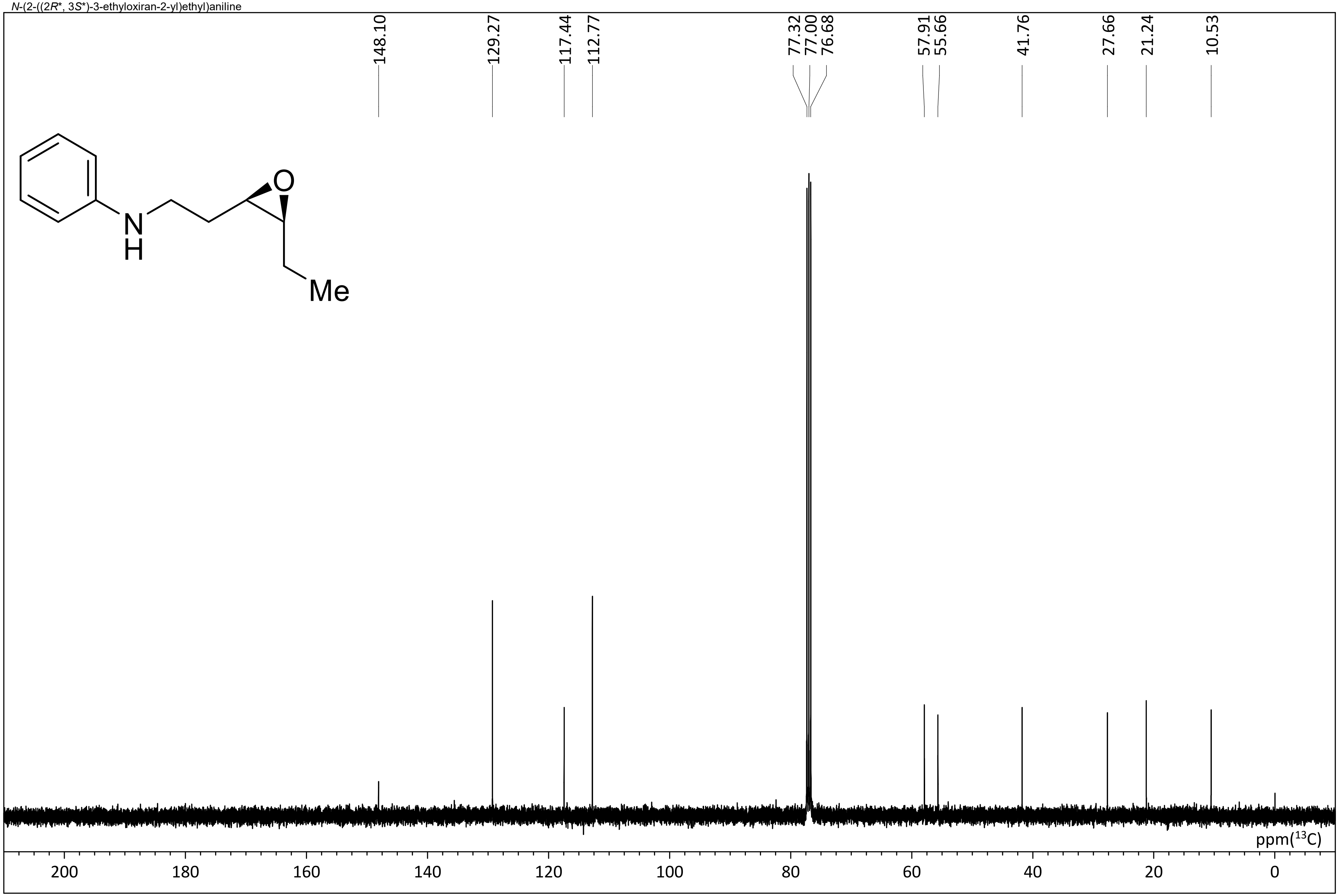

N-(2-((2*R**, 3*S**)-3-ethyloxiran-2-yl)ethyl)-4-methoxyaniline

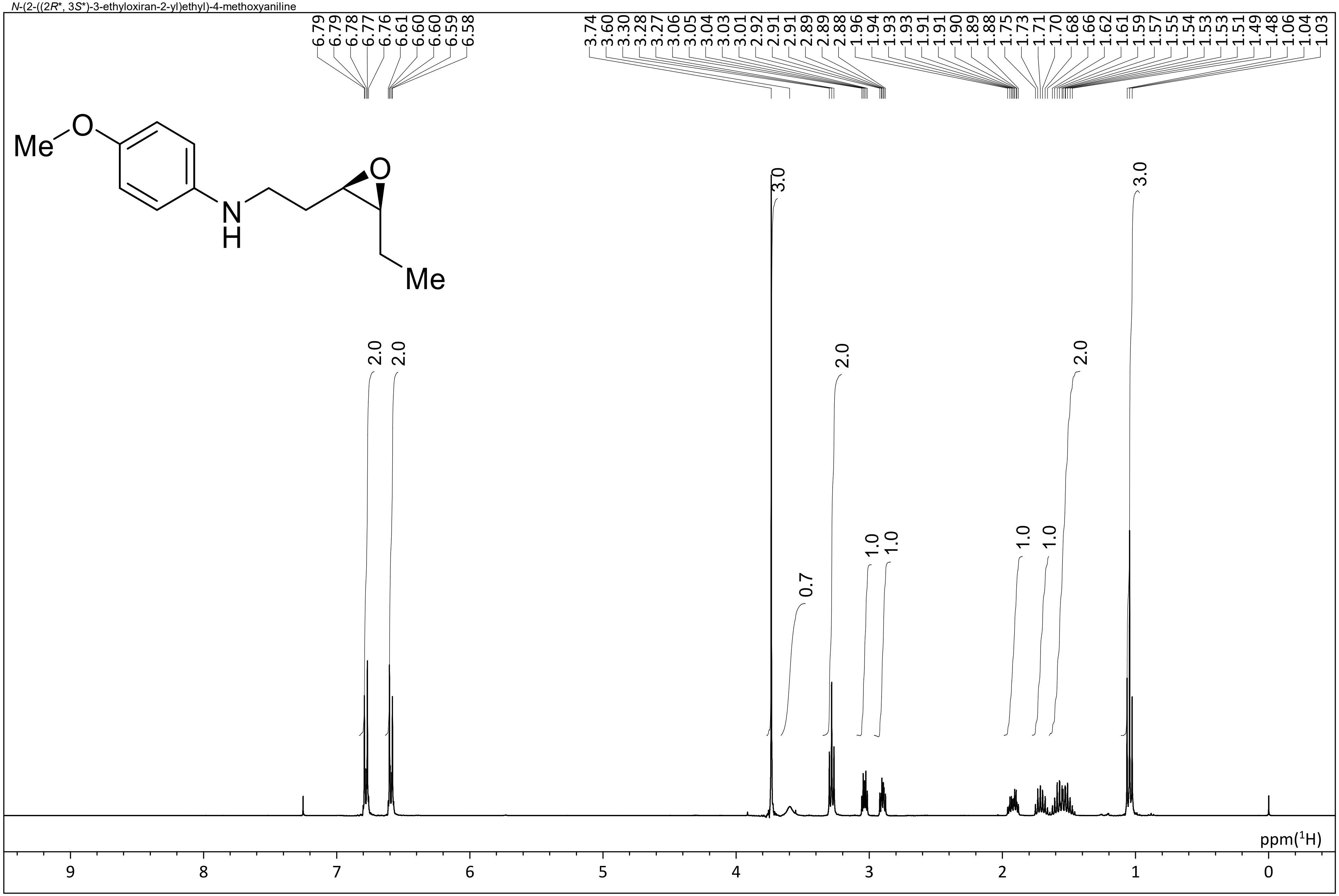

N-(2-((2*R** ,3*S**)-3-ethyloxiran-2-yl)ethyl)-4-methoxyaniline

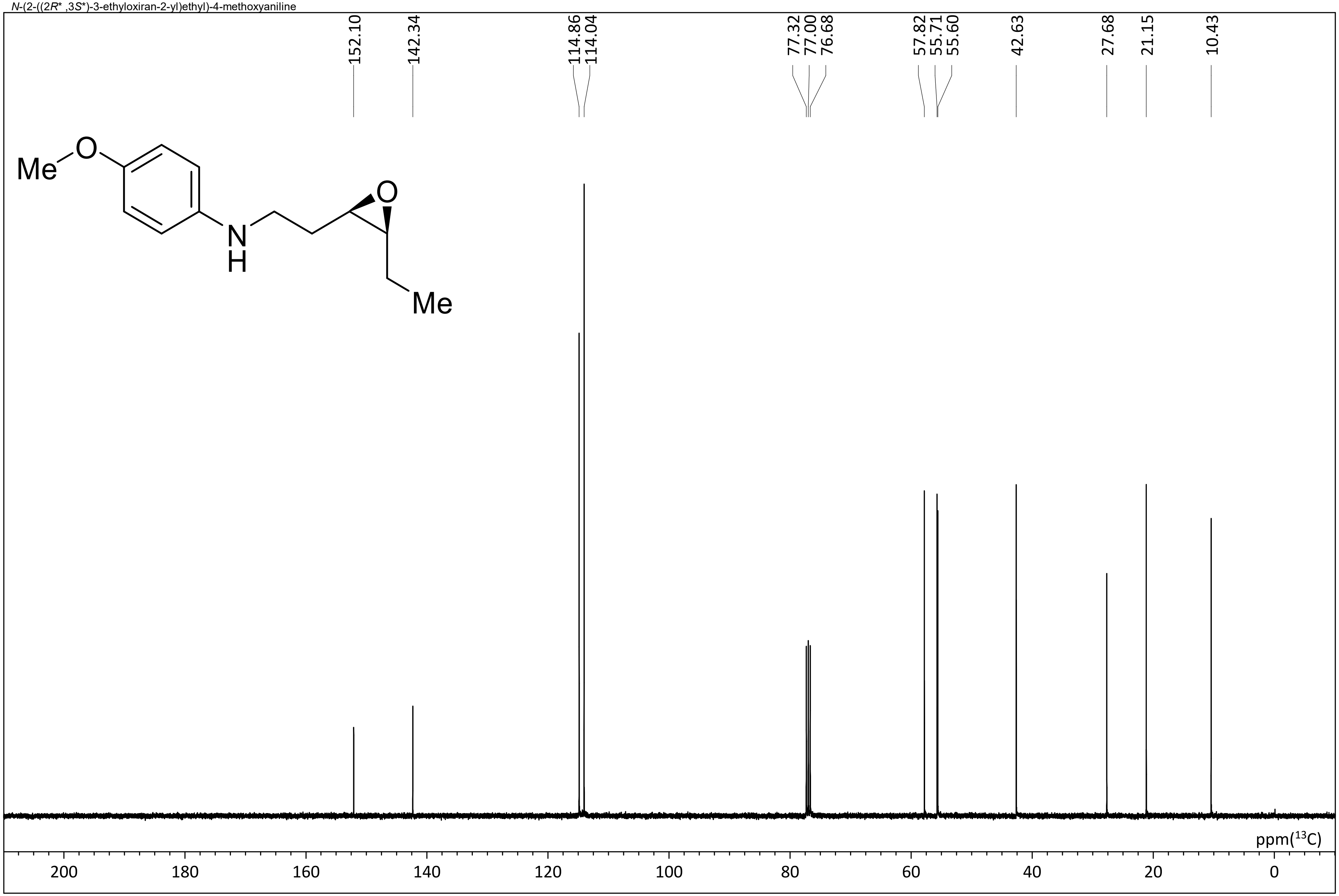

N-(2-((2*R**, 3*S**)-3-ethyloxiran-2-yl)ethyl)-4-nitroaniline

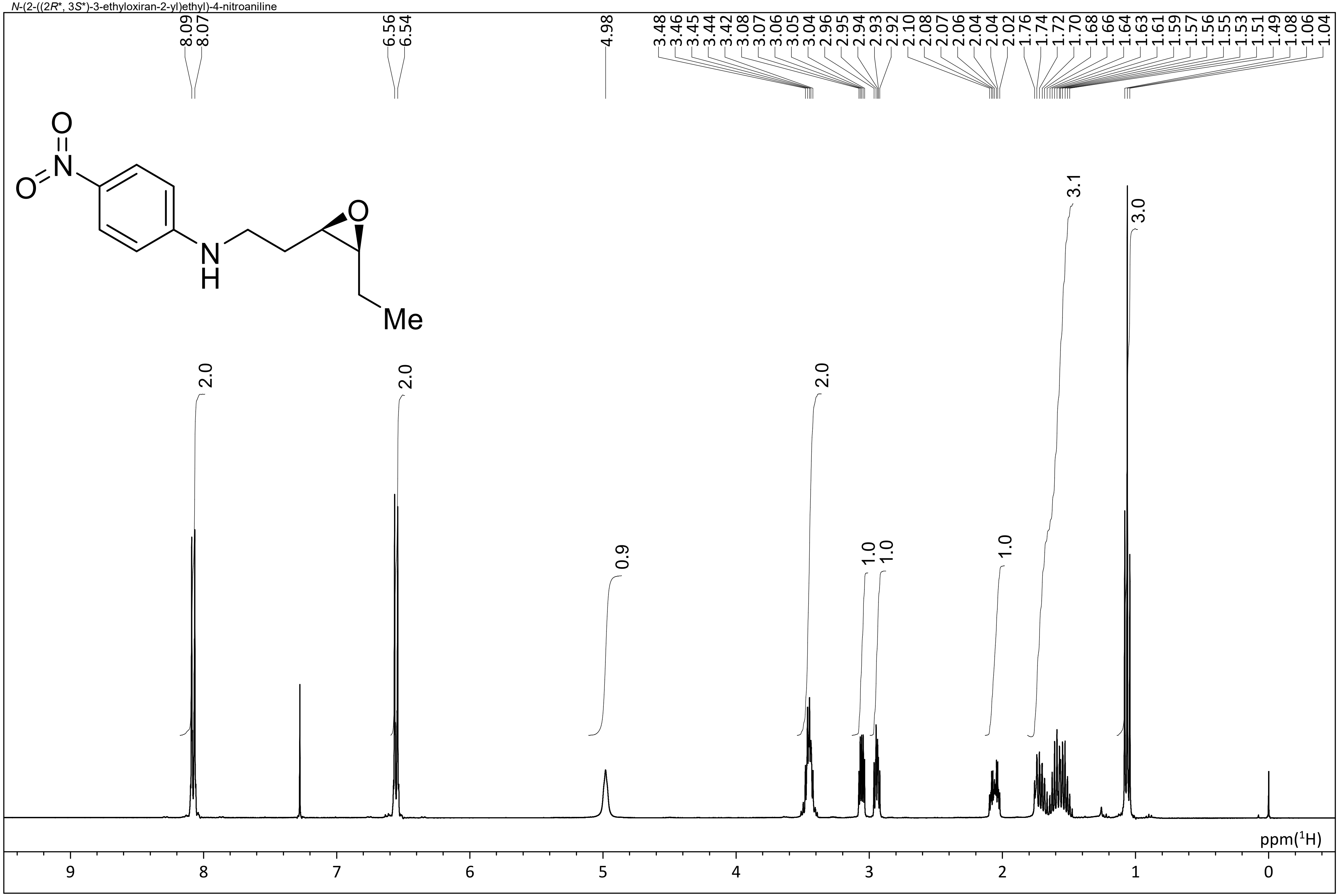

N-(2-((2*R**, 3*S**)-3-ethyloxiran-2-yl)ethyl)-4-nitroaniline

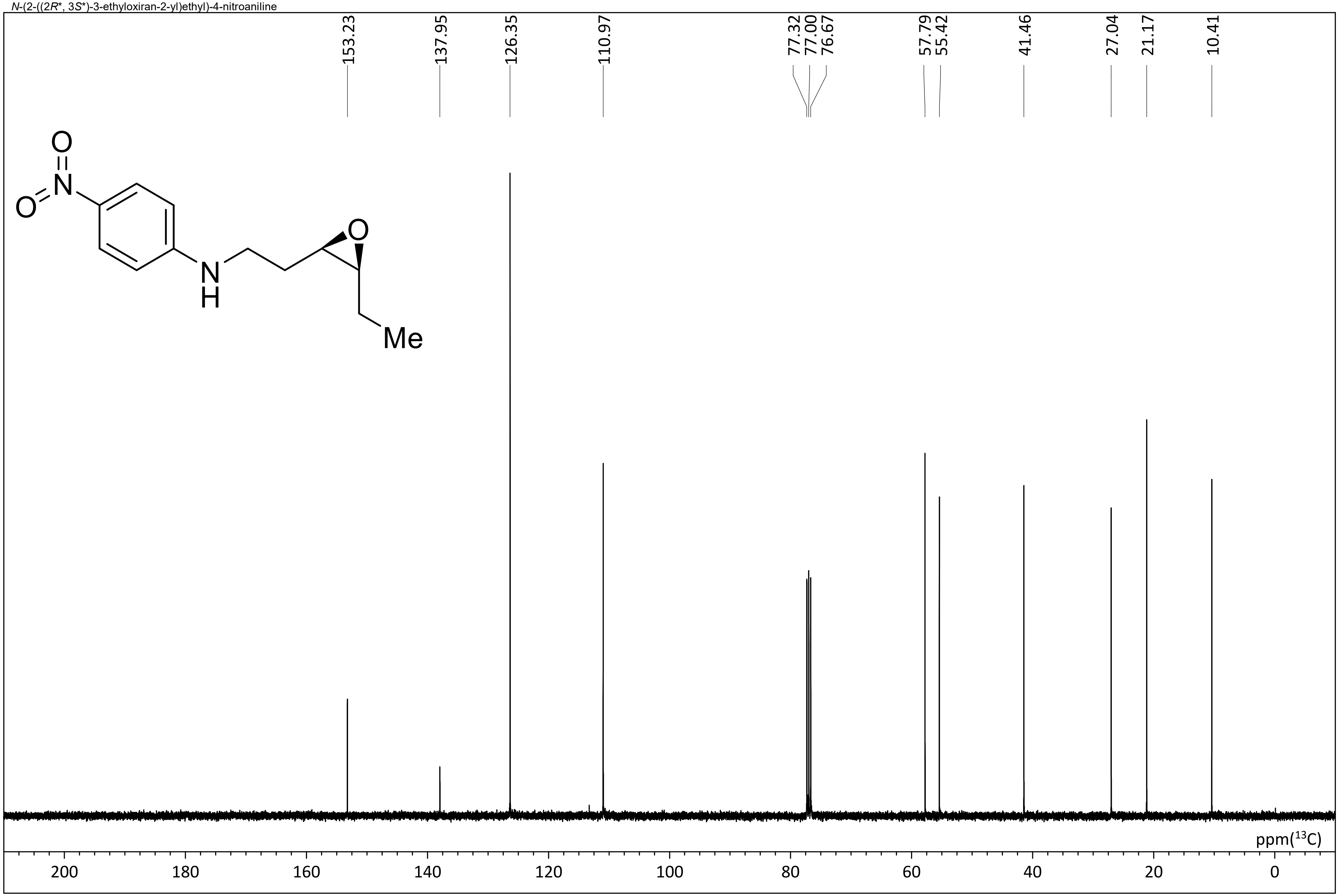

4-phenylbut-3-yn-1-ol)

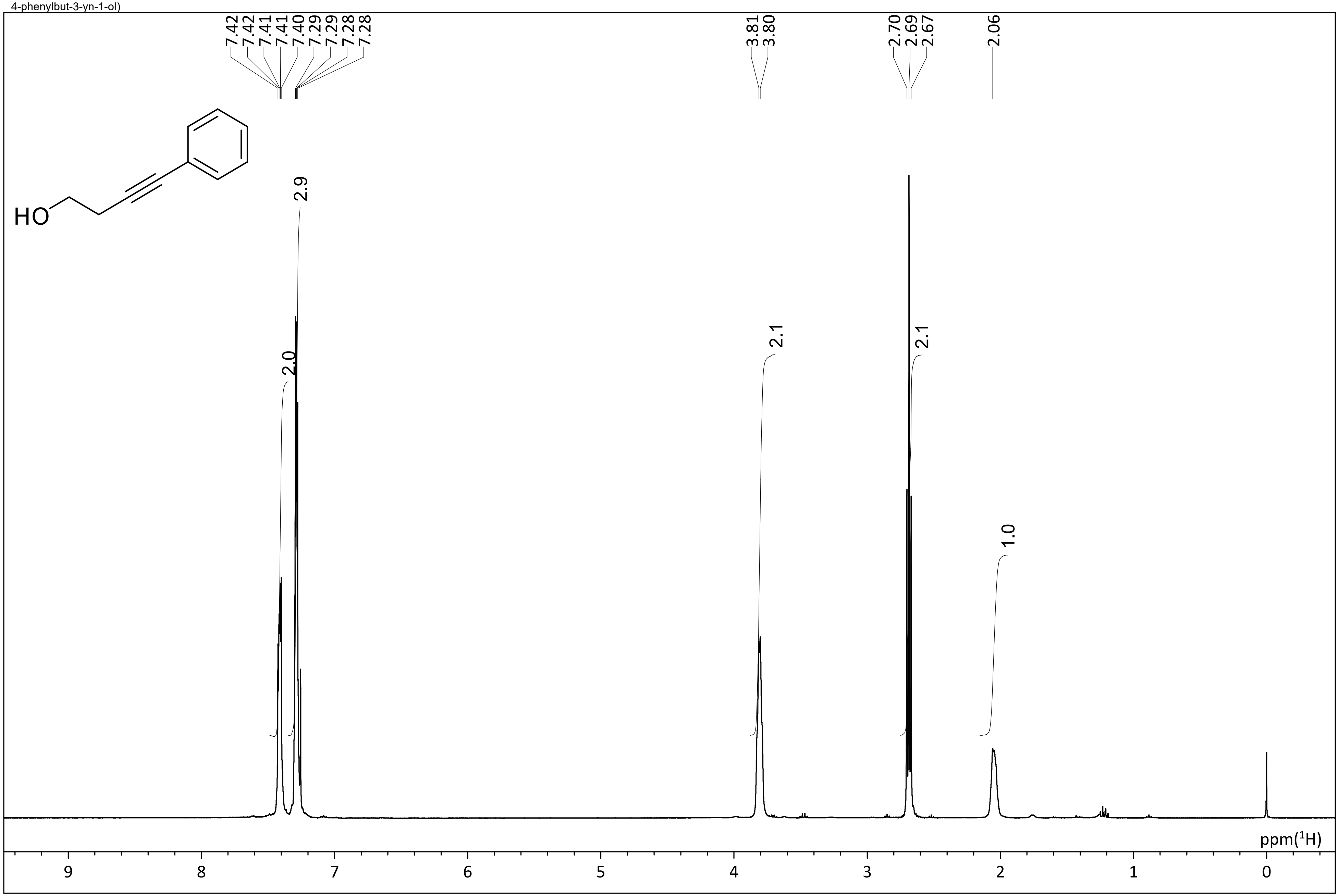

4-phenylbut-3-yn-1-ol

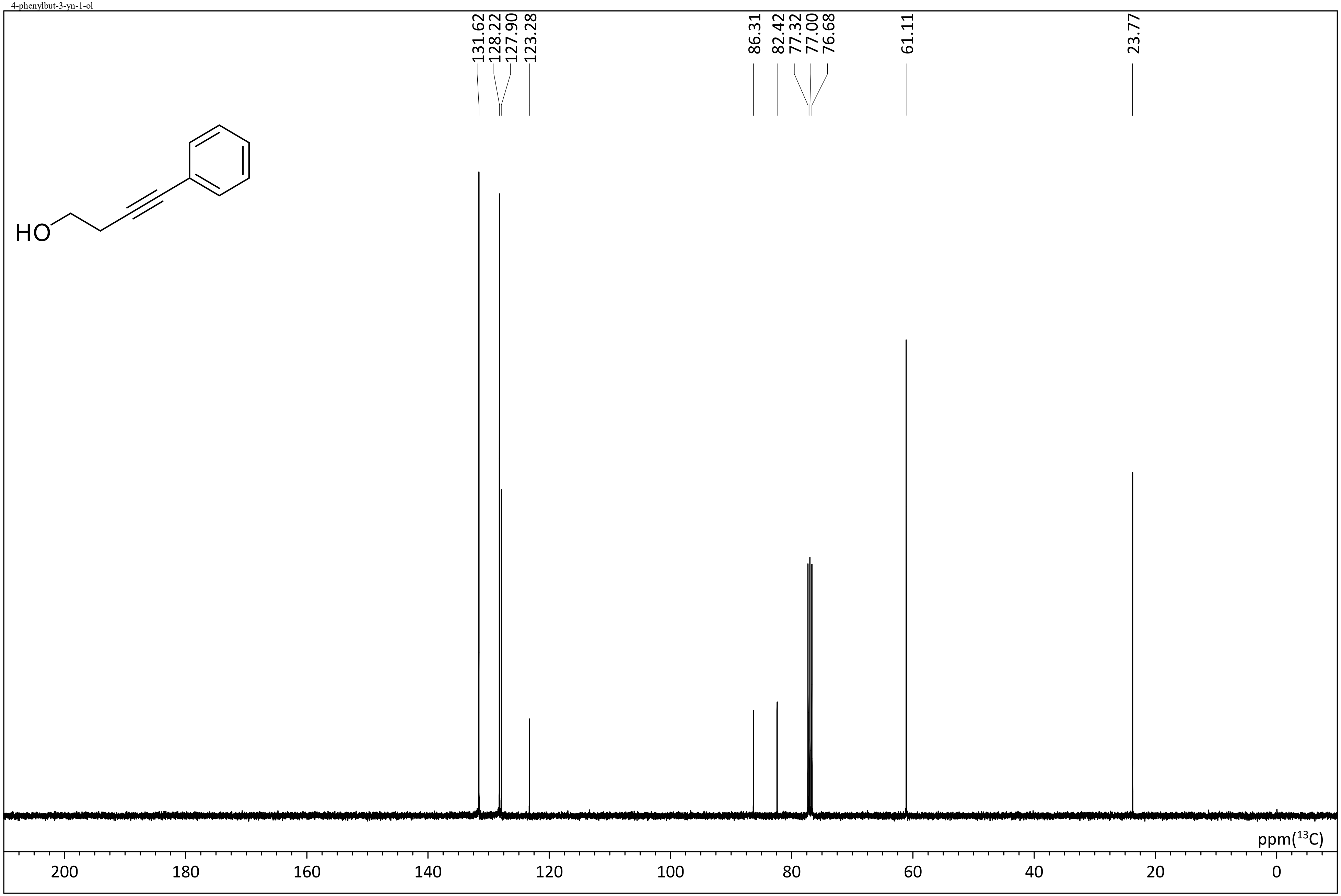

4-(4-fluorophenyl) but-3-yn-1-ol

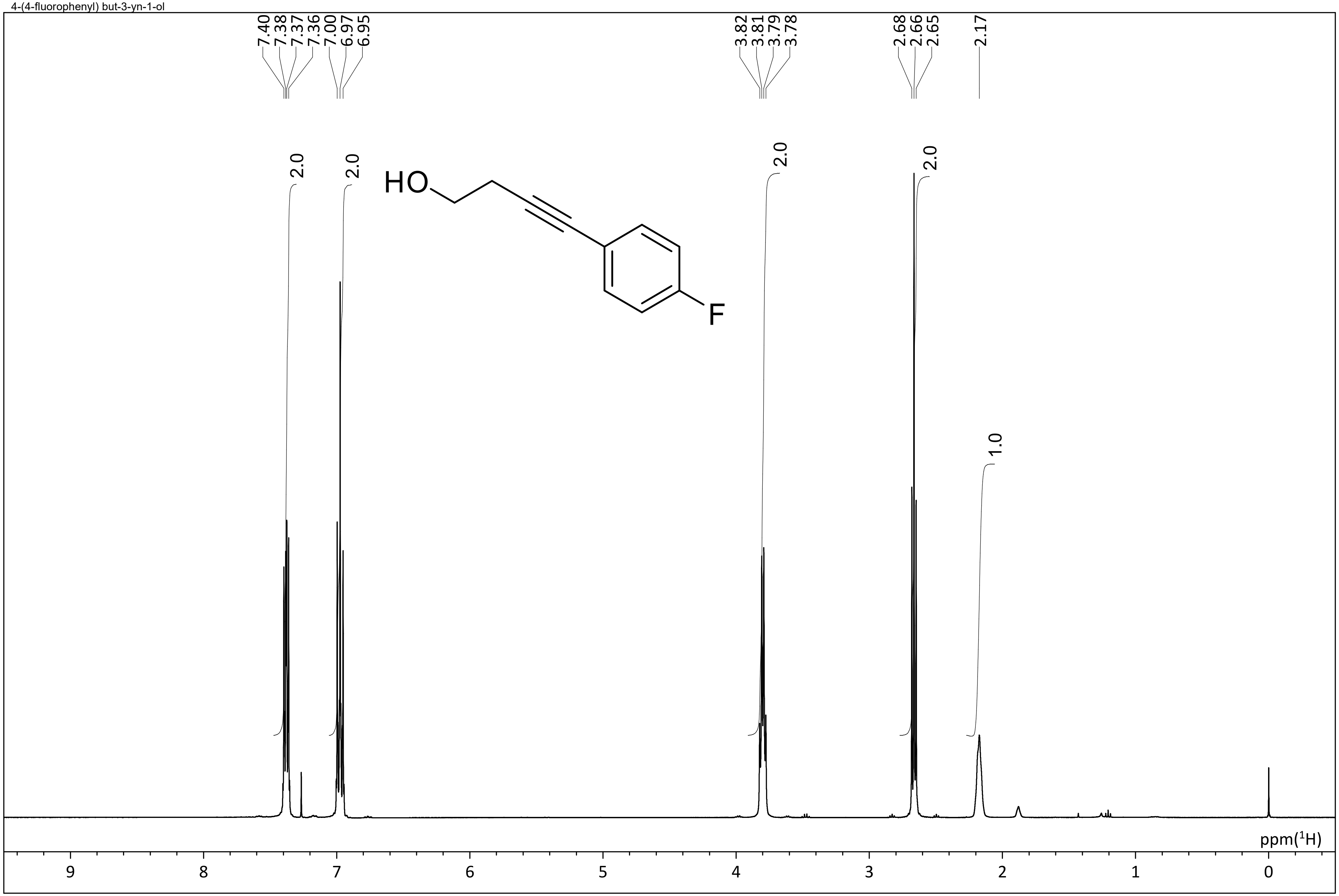

4-(4-fluorophenyl) but-3-yn-1-ol

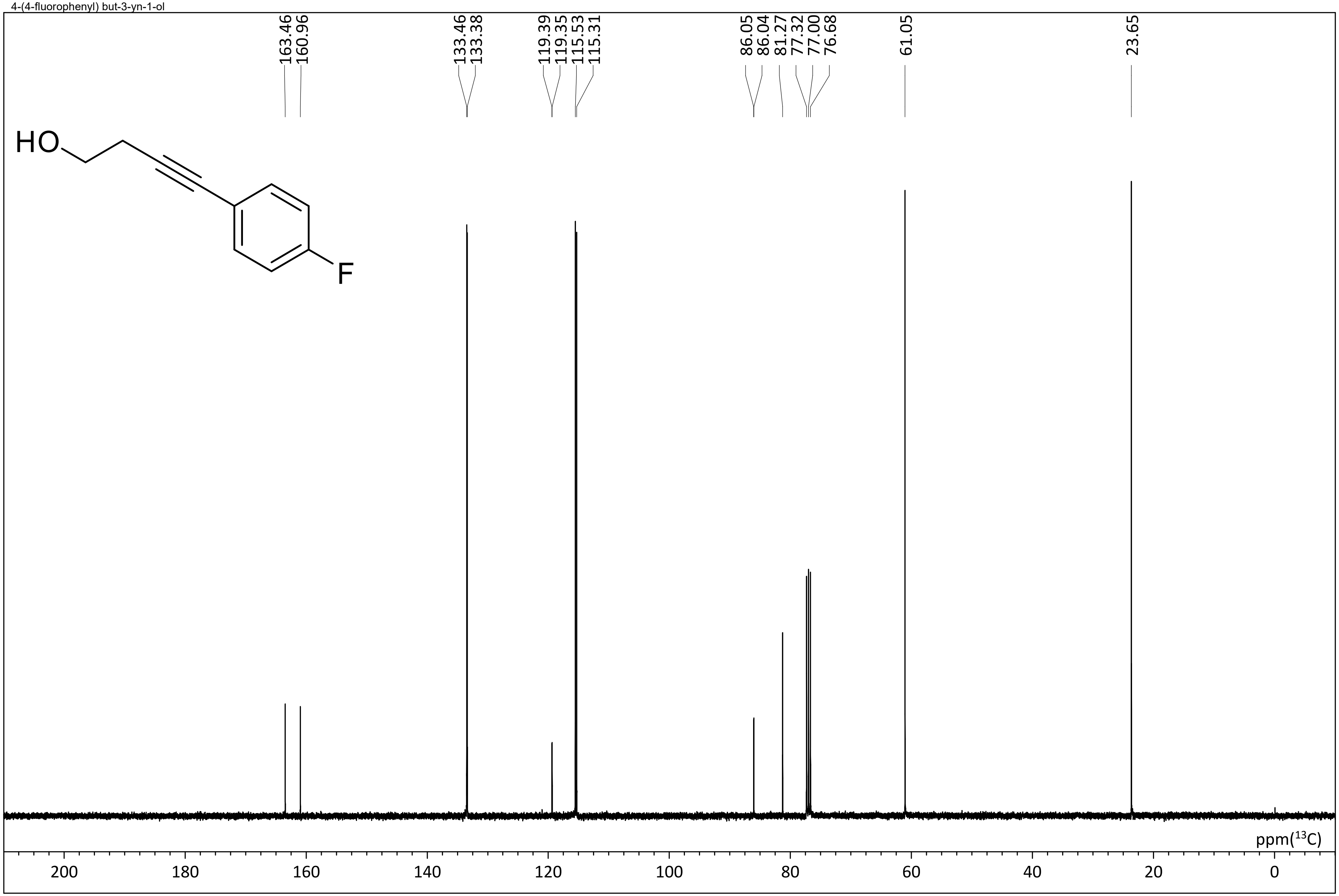

(*Z*)-4-phenylbut-3-en-1-ol

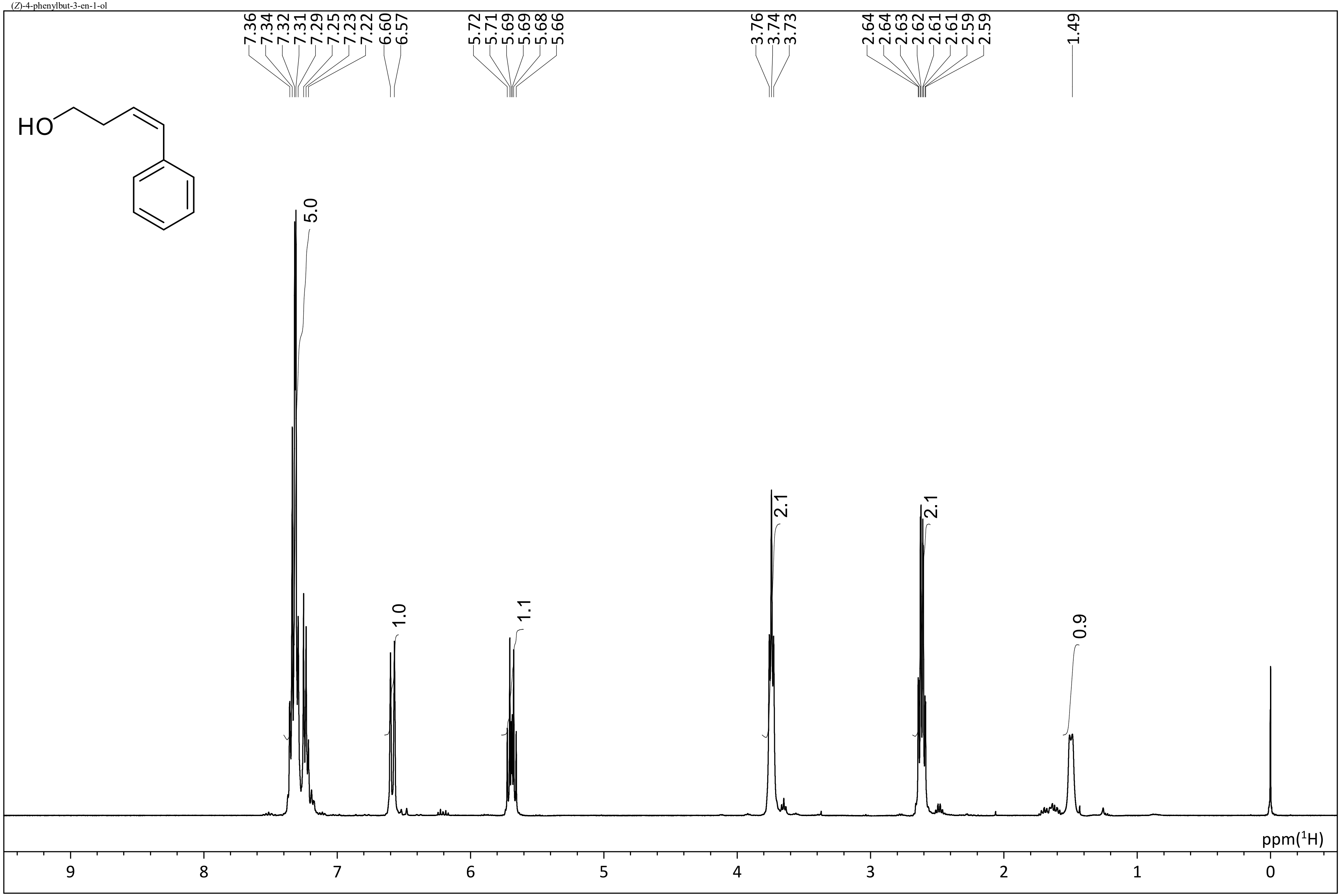

(*Z*)-4-phenylbut-3-en-1-ol

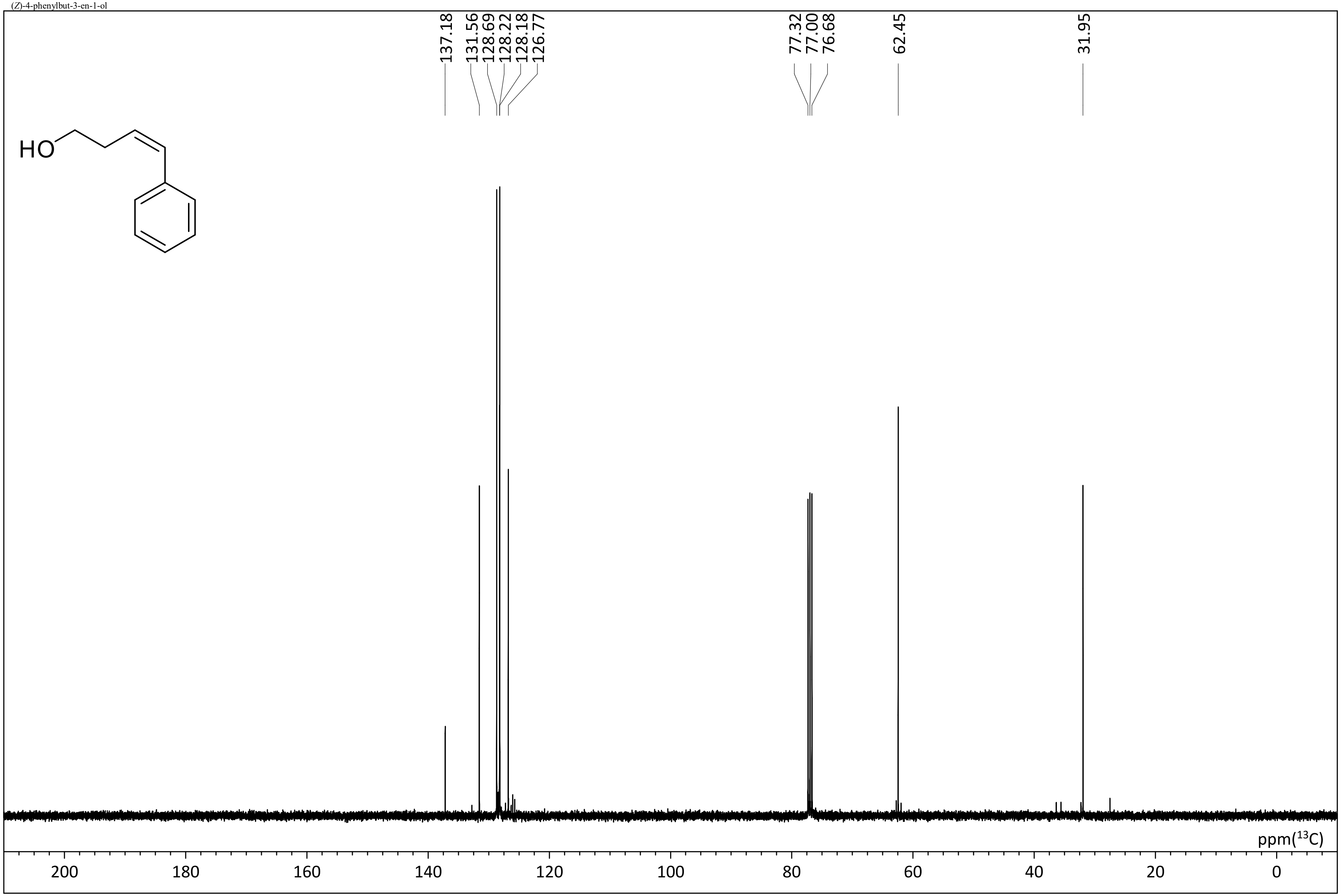

(*Z*)-4-(4-fluorophenyl)but-3-en-1-ol

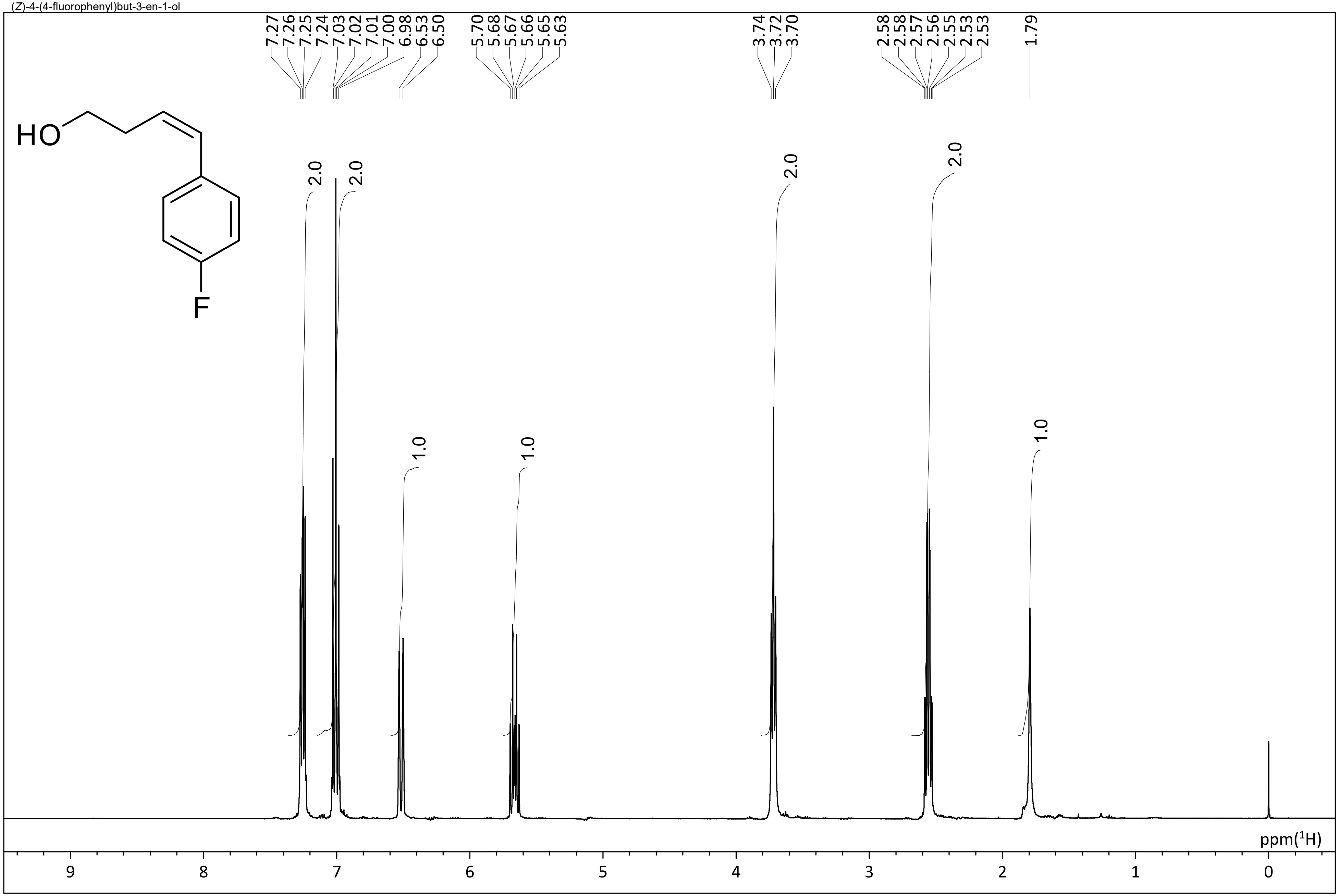

(*Z*)-4-(4-fluorophenyl)but-3-en-1-ol

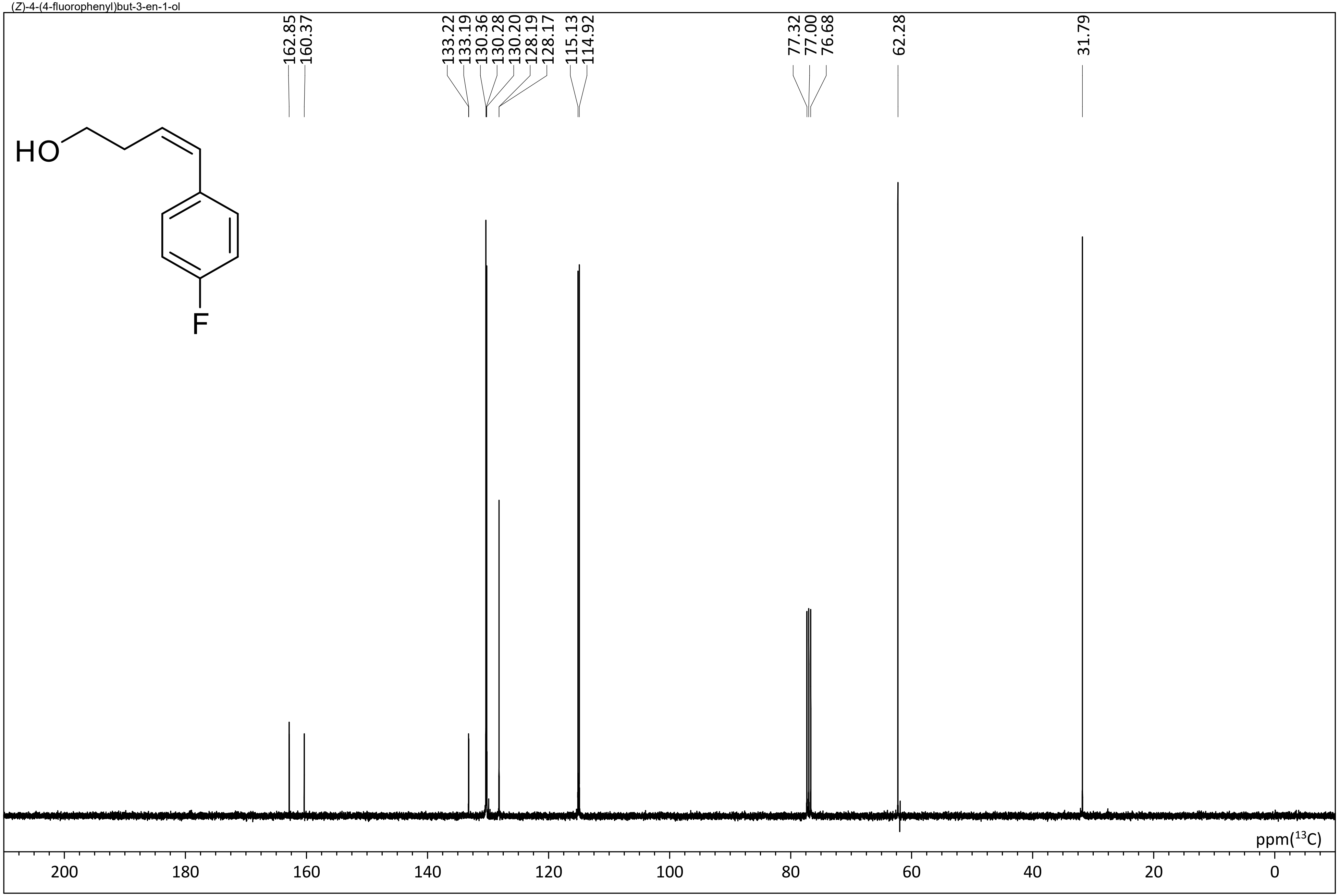

2-((2*R**,3*S**)-3-phenyloxiran-2-yl)ethan-1-ol

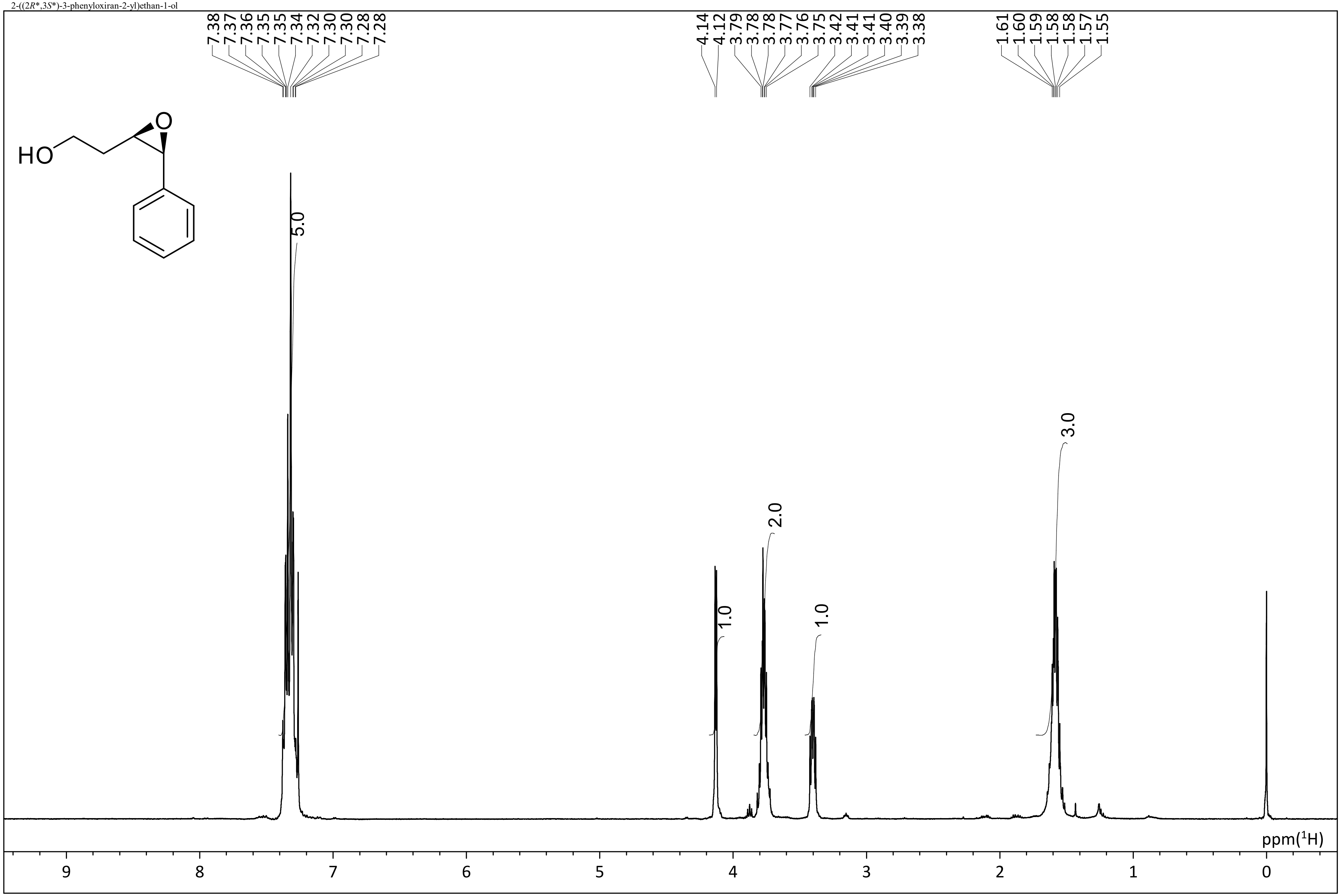

2-((2*R**,3*S**)-3-phenyloxiran-2-yl)ethan-1-ol

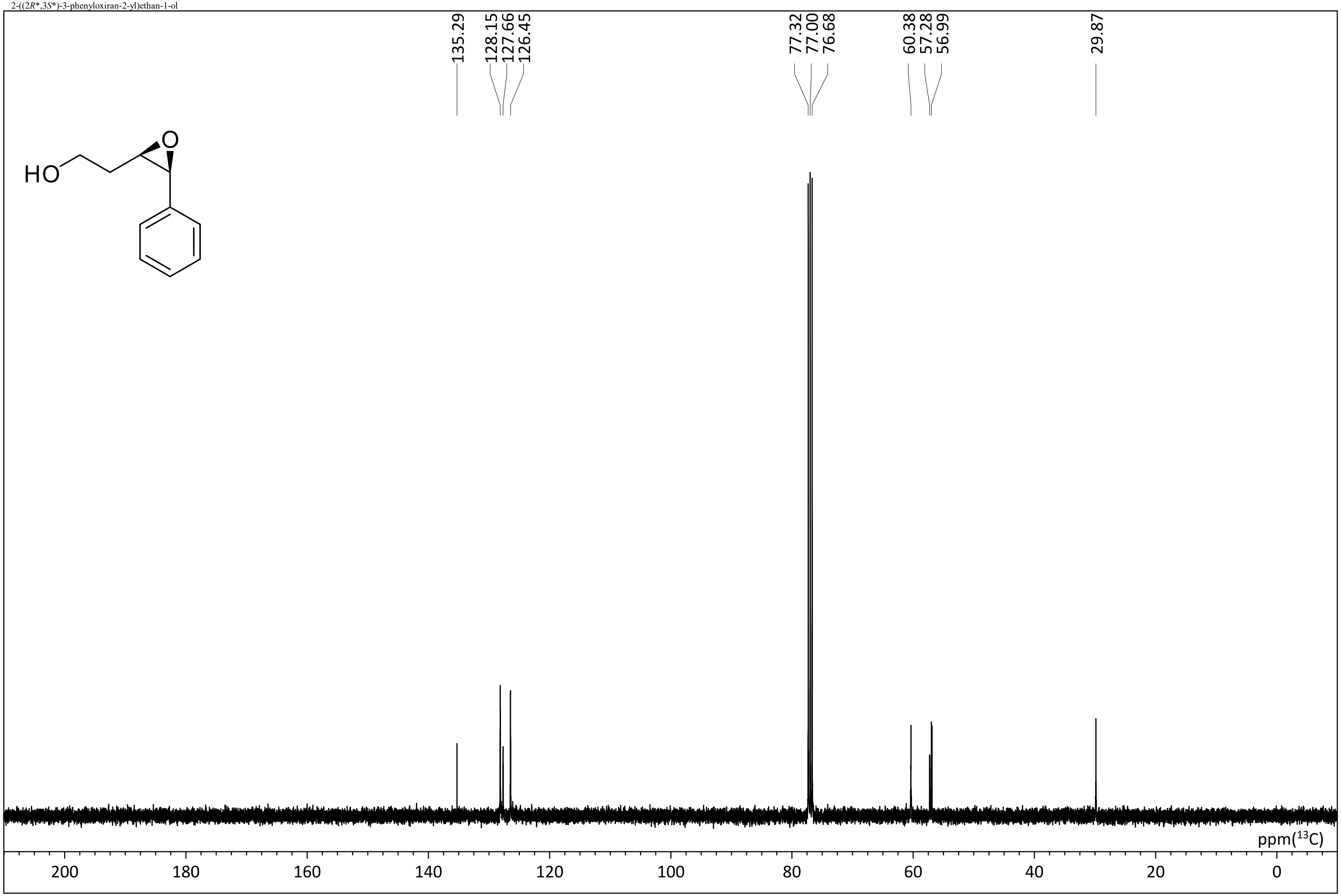

2-((2*R**, 3*S**)-3-(4-fluorophenyl)oxiran-2-yl)ethan-1-ol

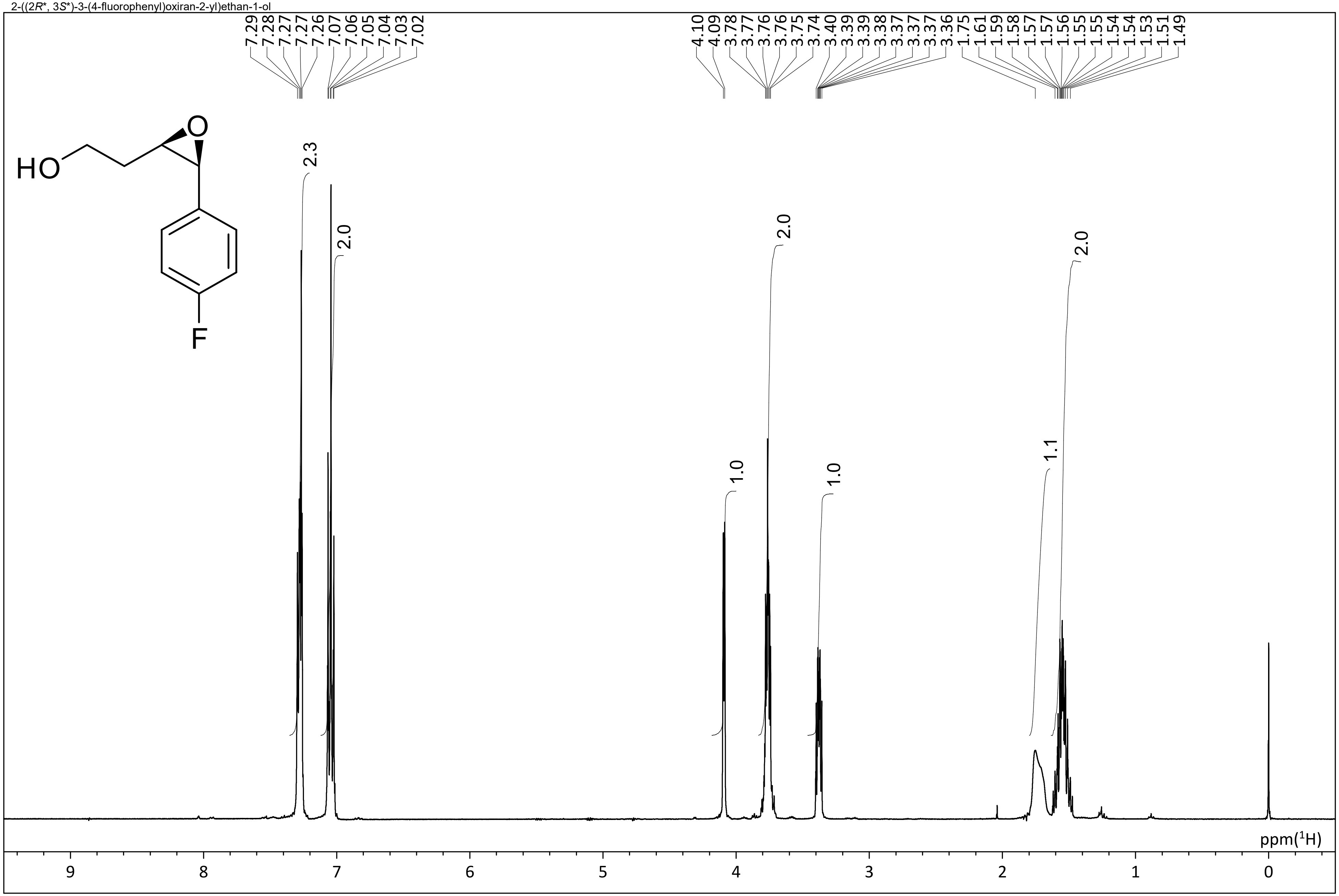

2-((2*R**, 3*S**)-3-(4-fluorophenyl)oxiran-2-yl)ethan-1-ol

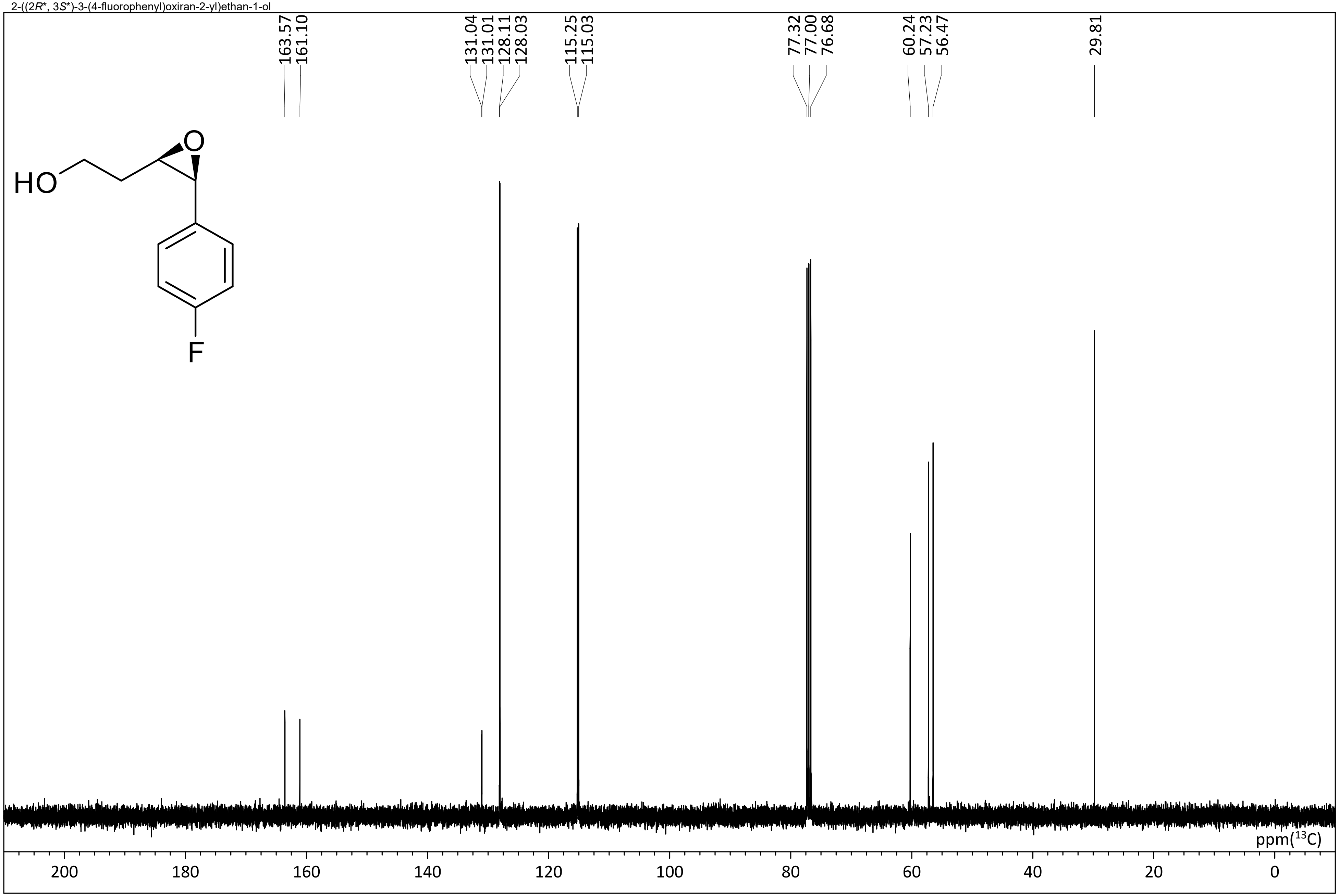

N-benzyl-2-((2*R**,3*S**)-3-phenyloxiran-2-yl)ethan-1-amine

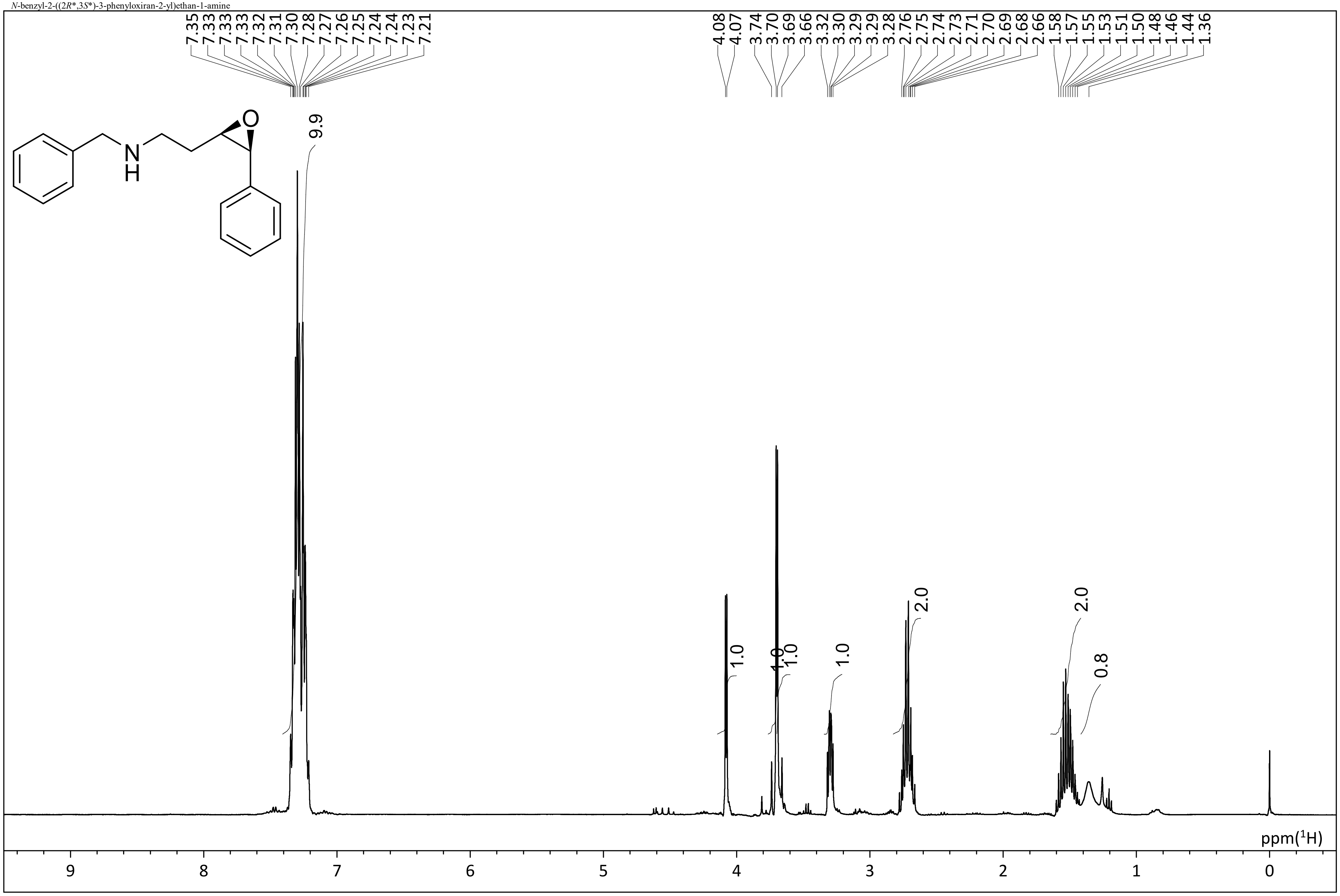

N-benzyl-2-((2*R**,3*S**)-3-phenyloxiran-2-yl)ethan-1-amine

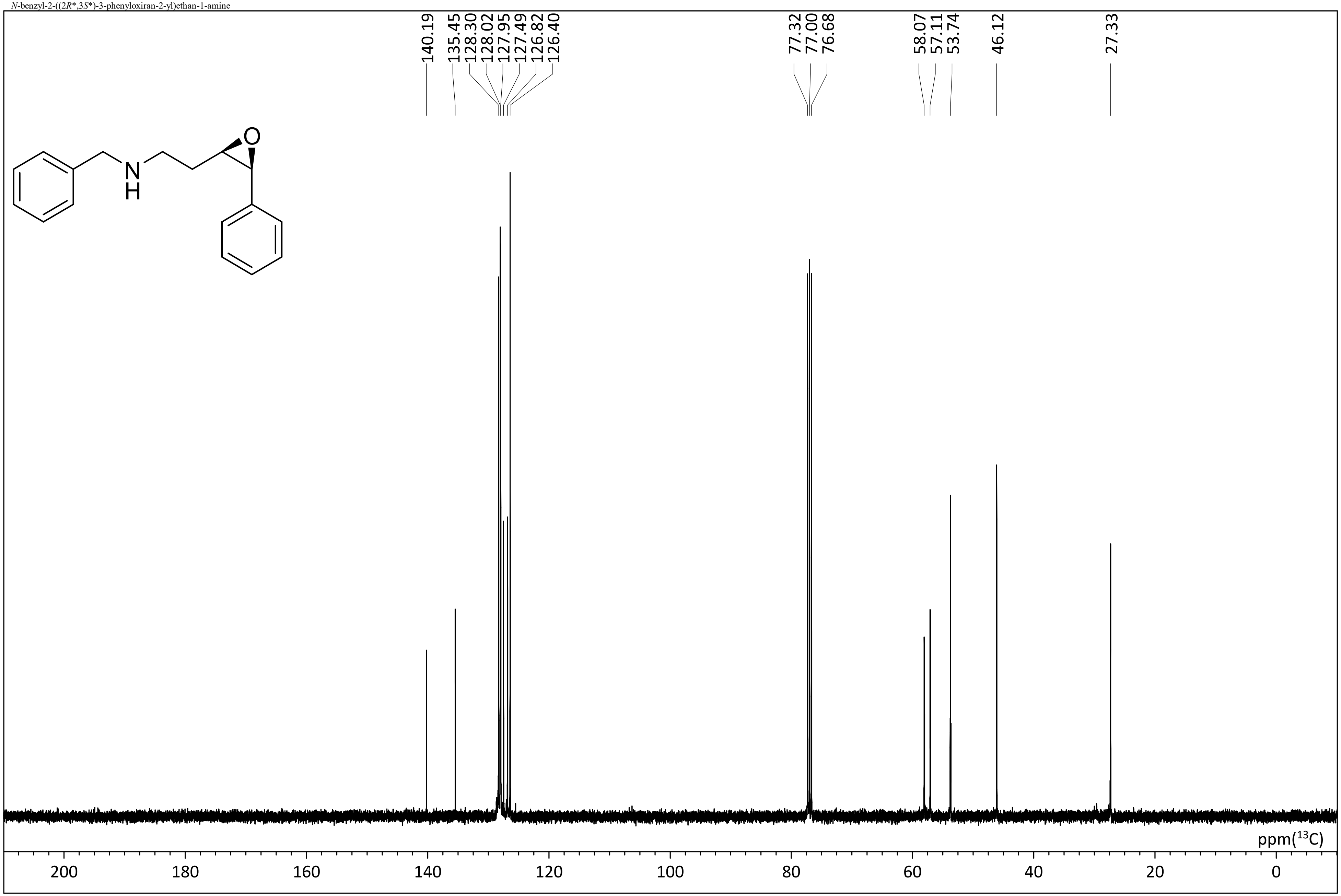

N-benzyl-2-((2*R**, 3*S**)-3-(4-fluorophenyl)oxiran-2-yl)ethan-1-amine

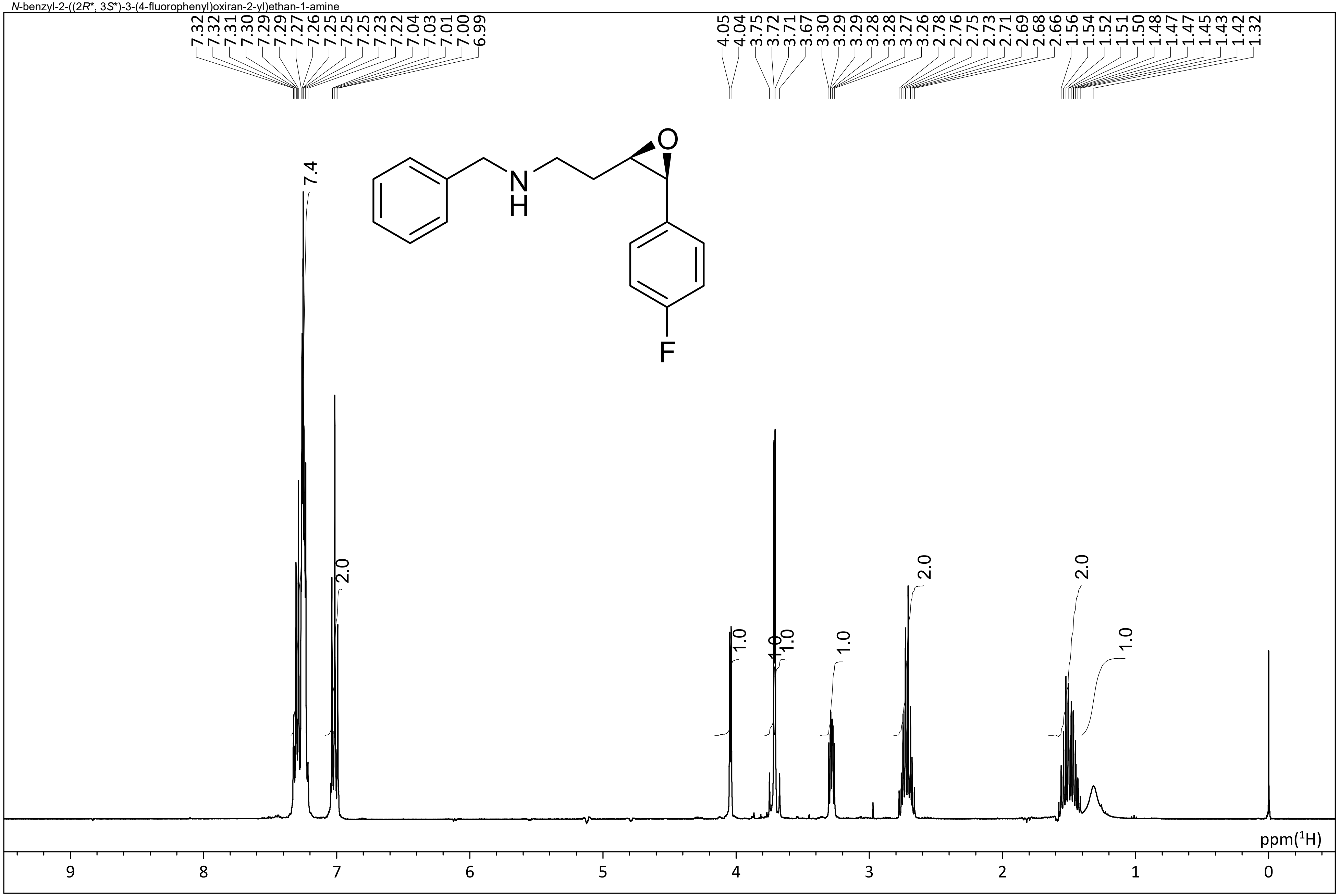

N-benzyl-2-((2*R**, 3*S**)-3-(4-fluorophenyl)oxiran-2-yl)ethan-1-amine

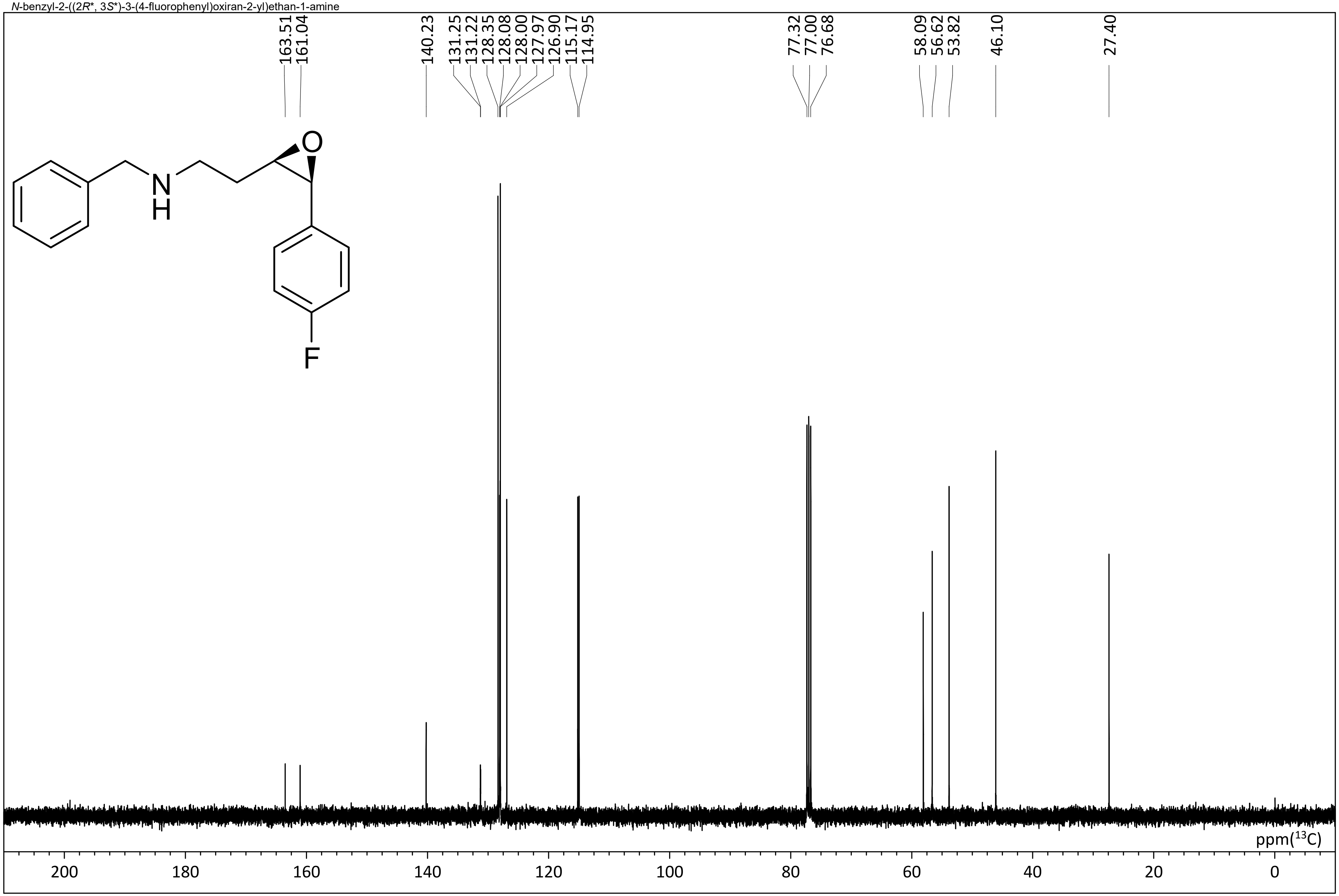

(S*)-1-((S*)-1-benzylazetidin-2-yl)propan-1-ol

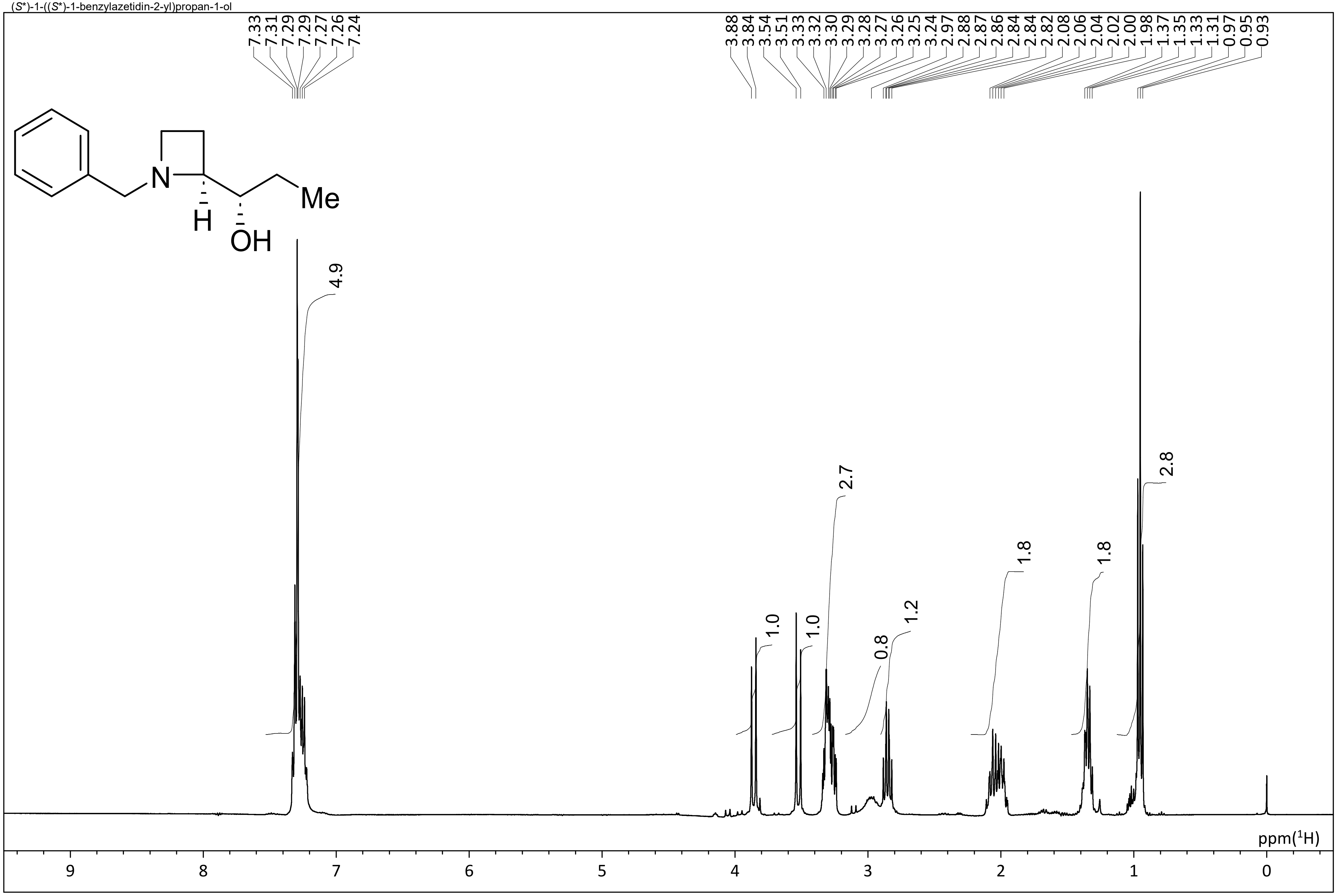

(*S**)-1-((*S**)-1-benzylazetidin-2-yl)propan-1-ol

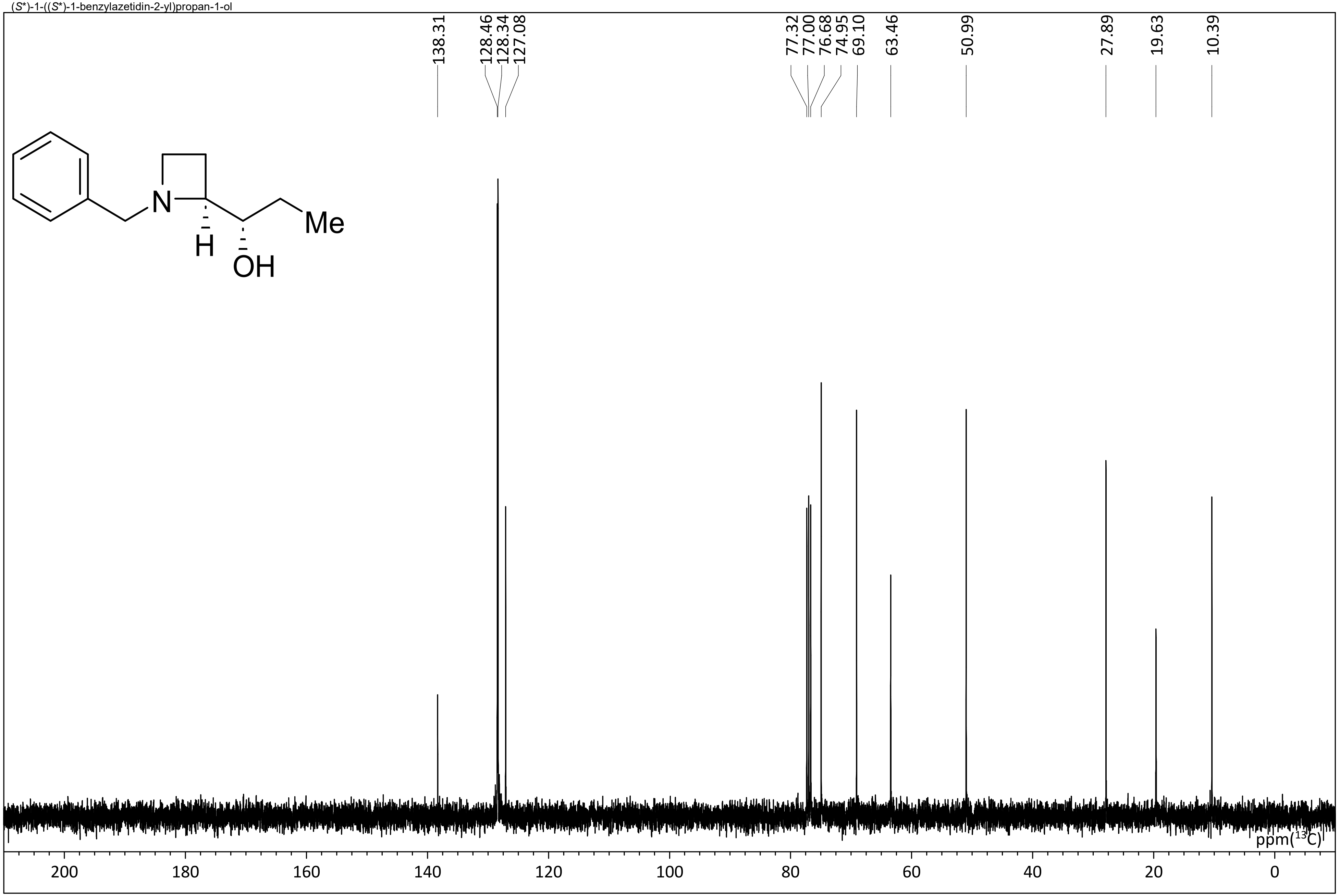

(*S**)-1-((*S**)-1-benzylazetidin-2-yl) propyl acetate

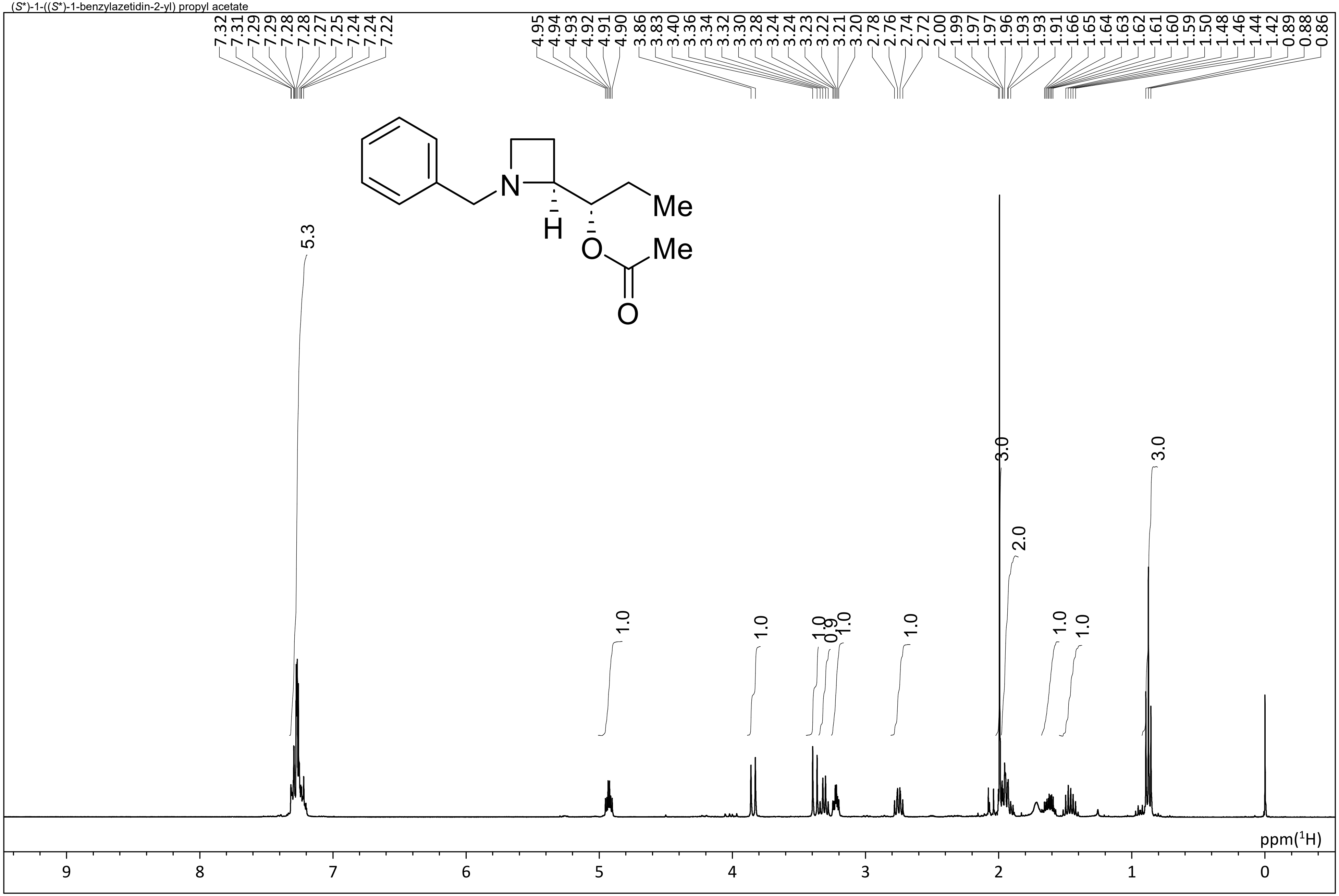

COSY

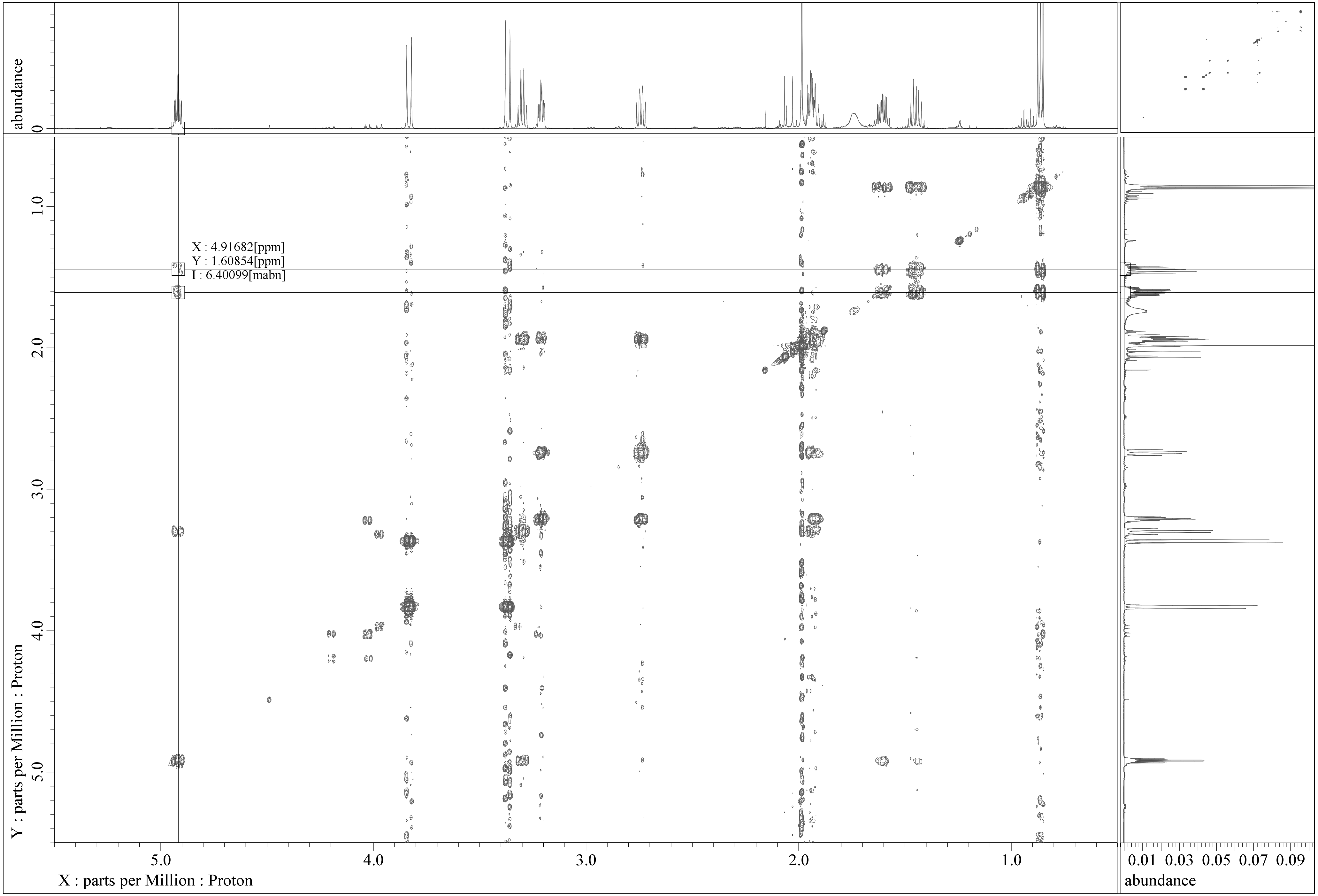
abundance
0
X : 4.91682[ppm]
Y : 1.60854[ppm]
I : 6.40099[mabn]
Y : parts per Million : Proton
1.0
2.0
3.0
4.0
5.0
X : parts per Million : Proton
0.01 0.03 0.05 0.07 0.09
abundance

(*S**)-1-((*S**)-1-benzylazetidin-2-yl) propyl acetate

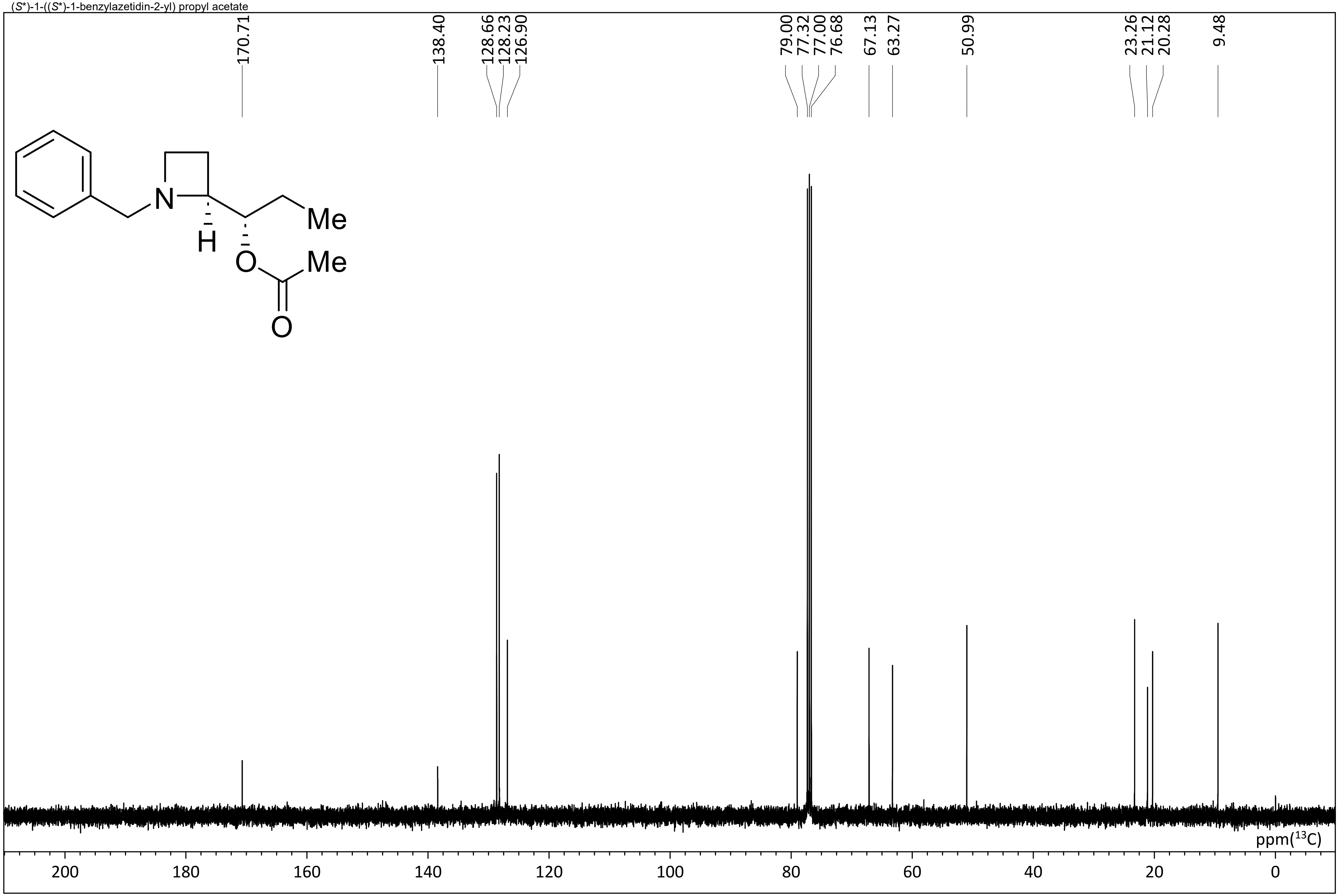

(*S**)-1-((*S**)-1-(4-methoxybenzyl)azetidin-2-yl)propan-1-ol

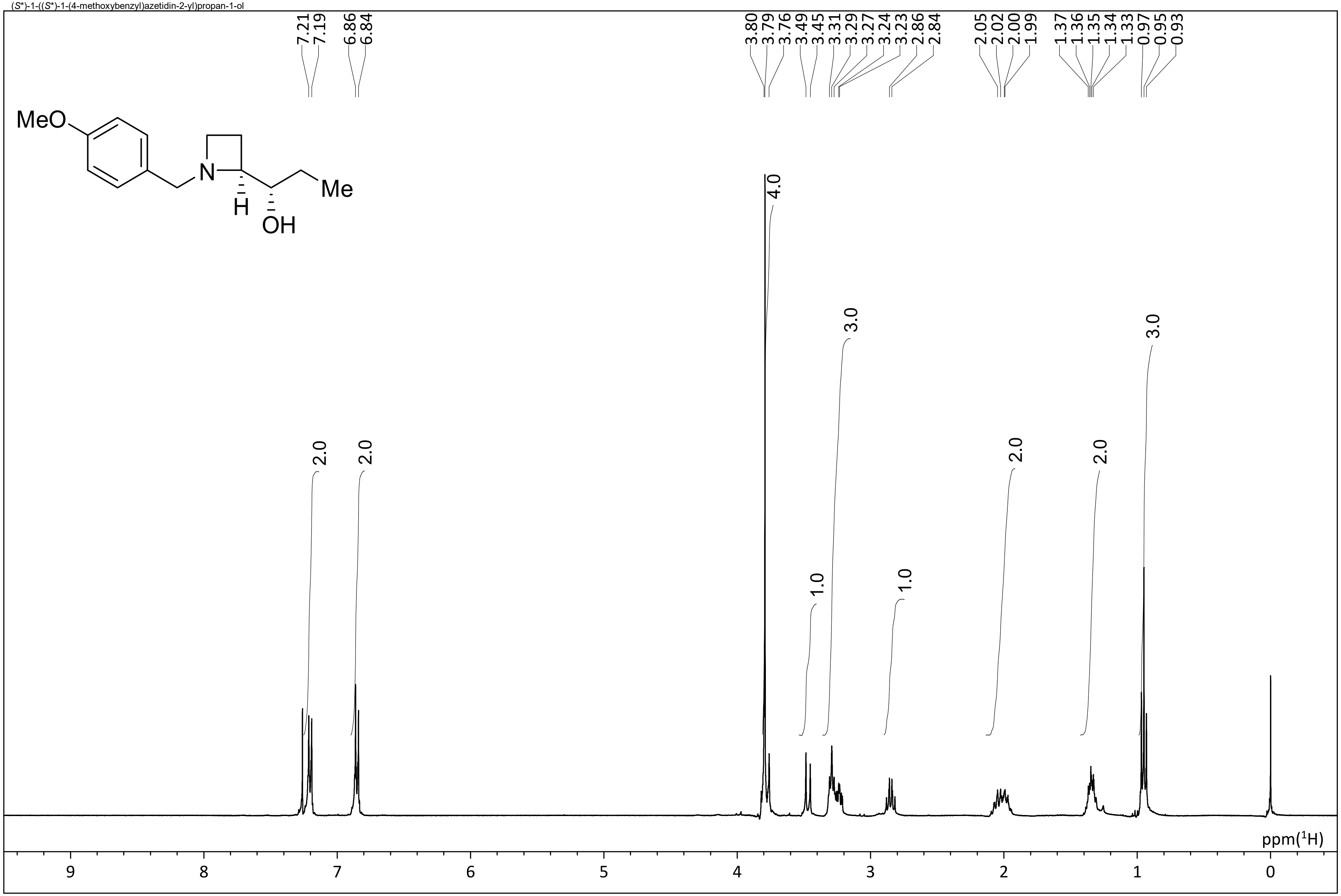

(S*)-1-((S*)-1-(4-methoxybenzyl)azetidin-2-yl)propan-1-ol

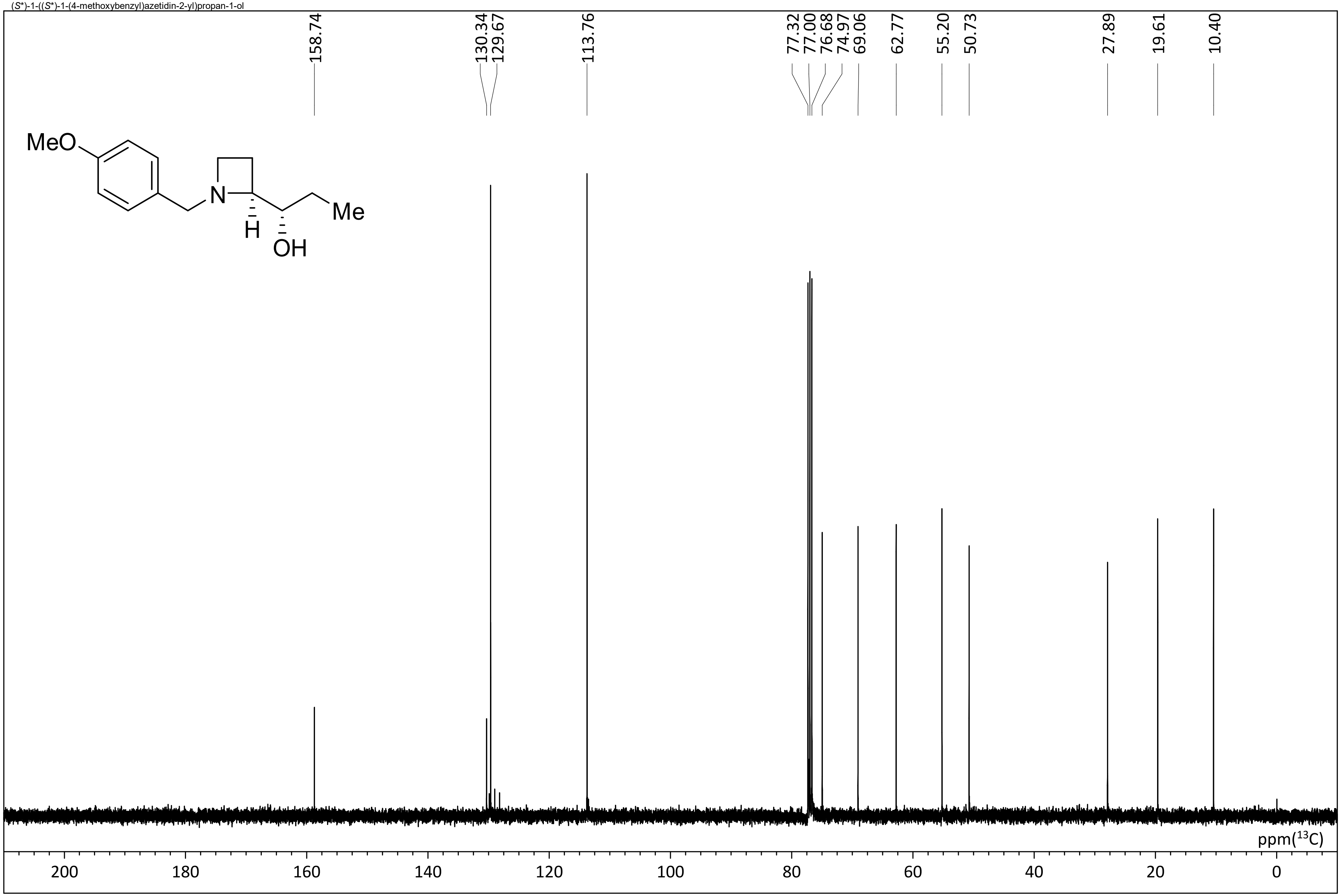

(*S**)-1-((*S**)-1-(4-(trifluoromethyl)benzyl)azetidin-2-yl)propan-1-ol

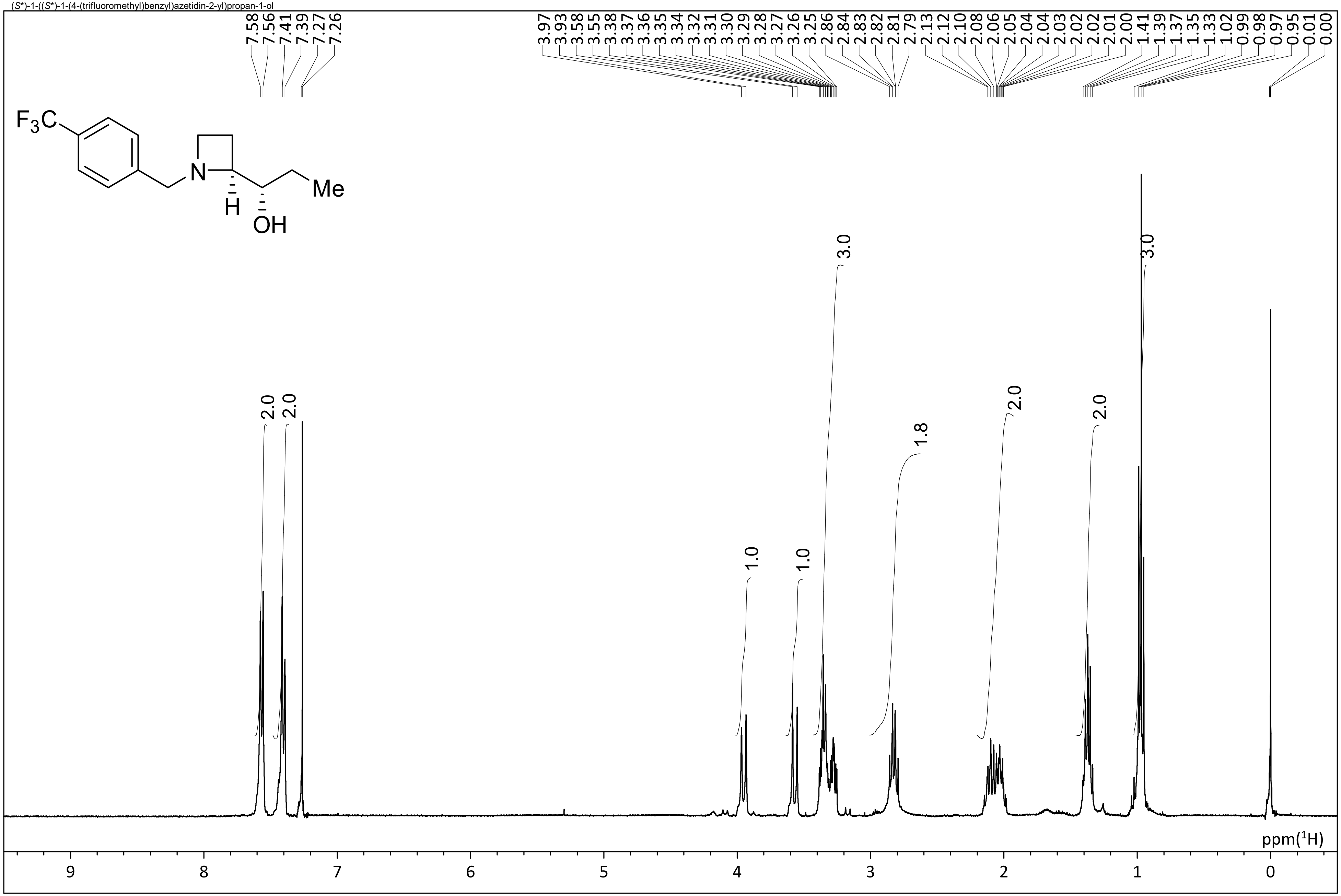

(*S**)-1-((*S**)-1-(4-(trifluoromethyl)benzyl)azetidin-2-yl)propan-1-ol

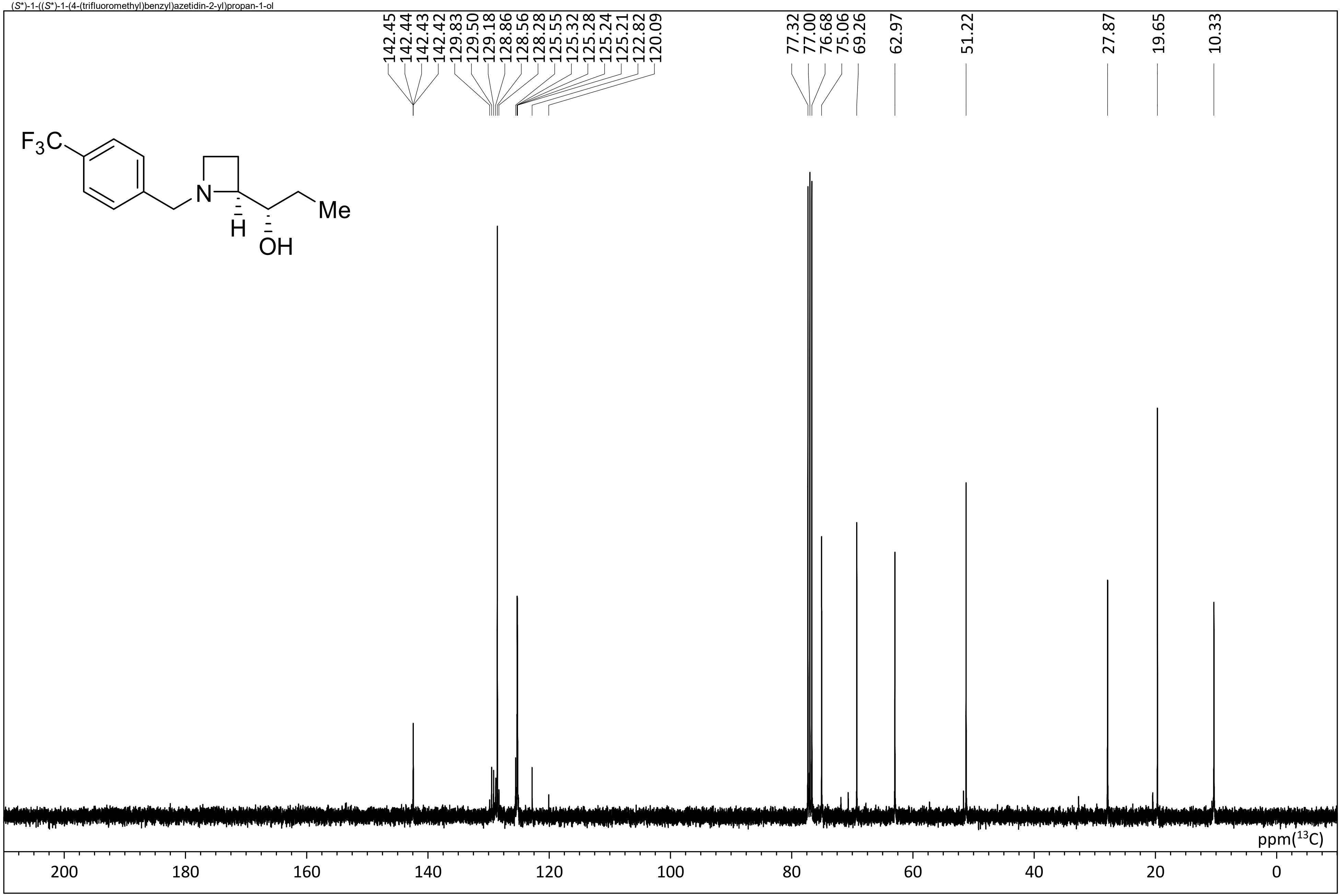

(*S**)-1-((*S**)-1-butylazetidin-2-yl)propan-1-ol

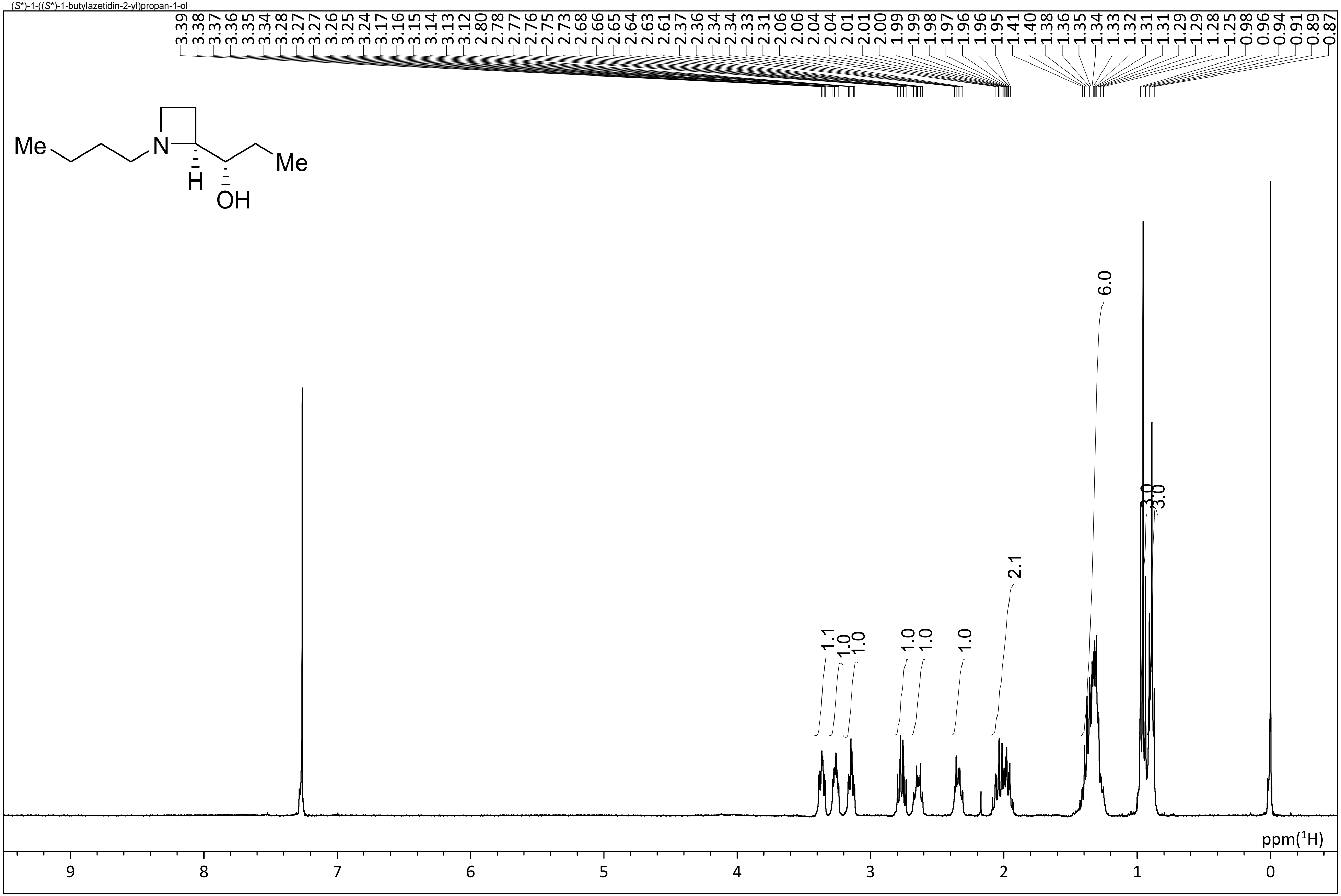

(*S**)-1-((*S**)-1-butylazetidin-2-yl)propan-1-ol

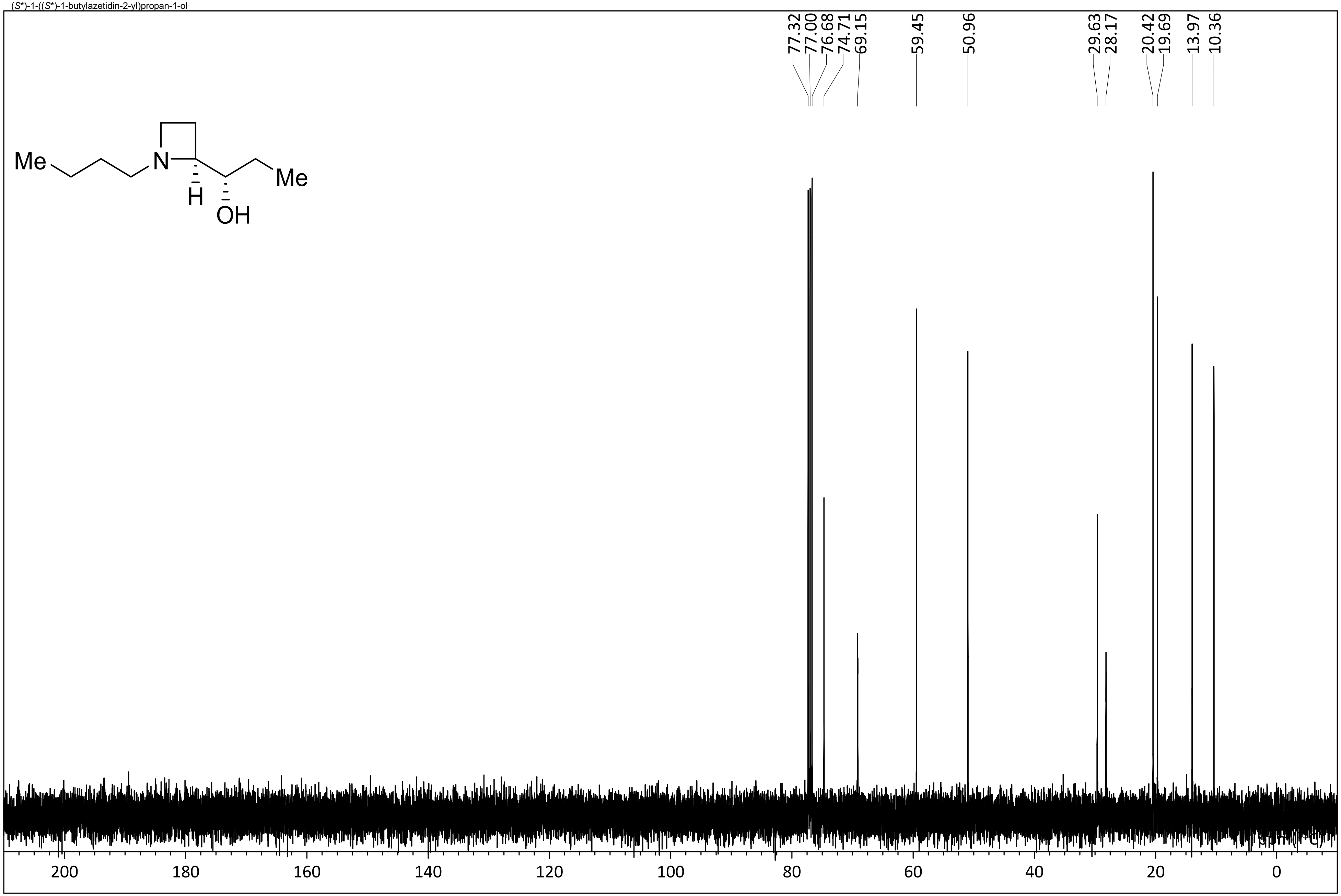

(*S**)-1-((*S**)-1-(*tert*-butyl)azetidin-2-yl)propan-1-ol

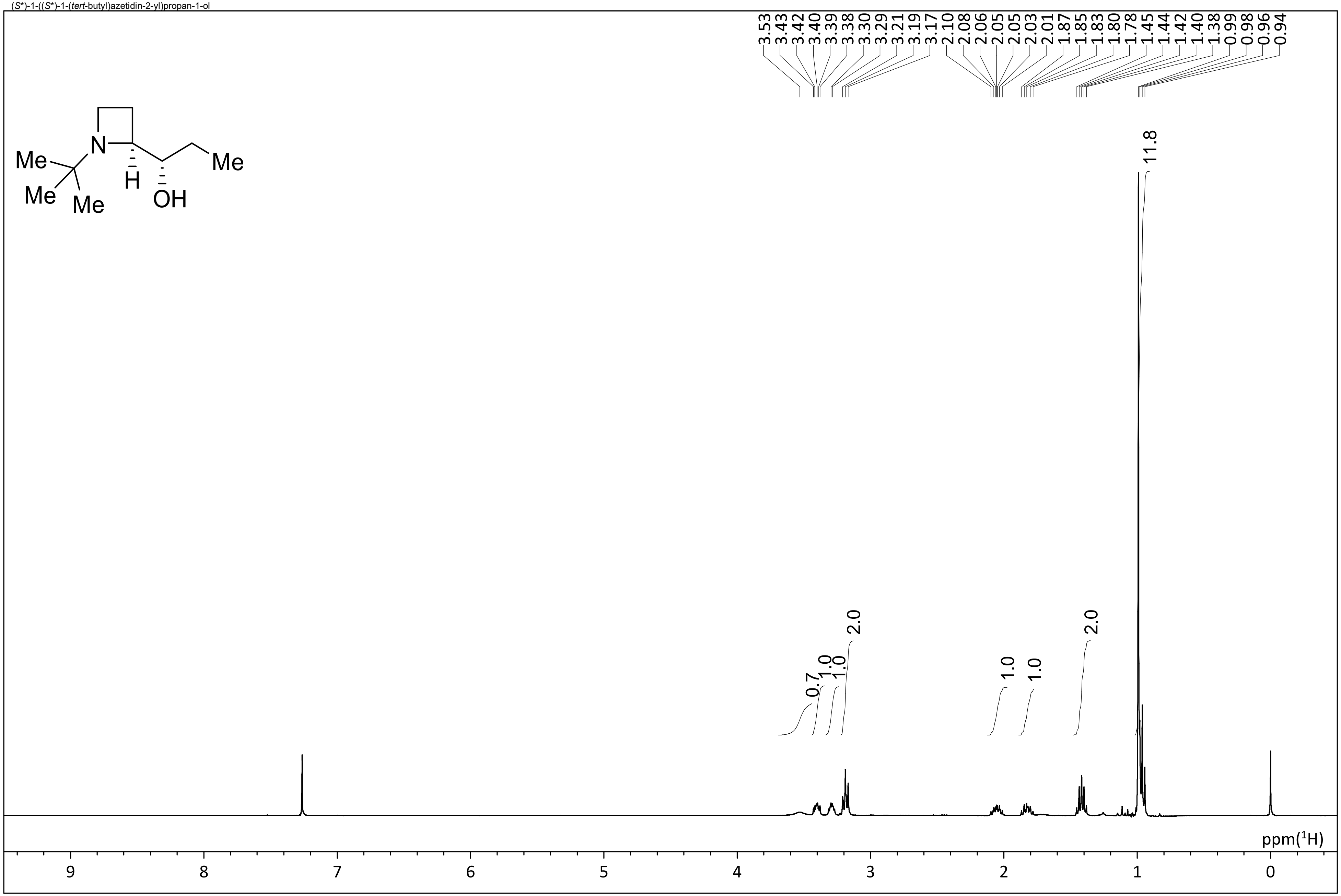

(*S**)-1-((*S**)-1-(*tert*-butyl)azetidin-2-yl)propan-1-ol

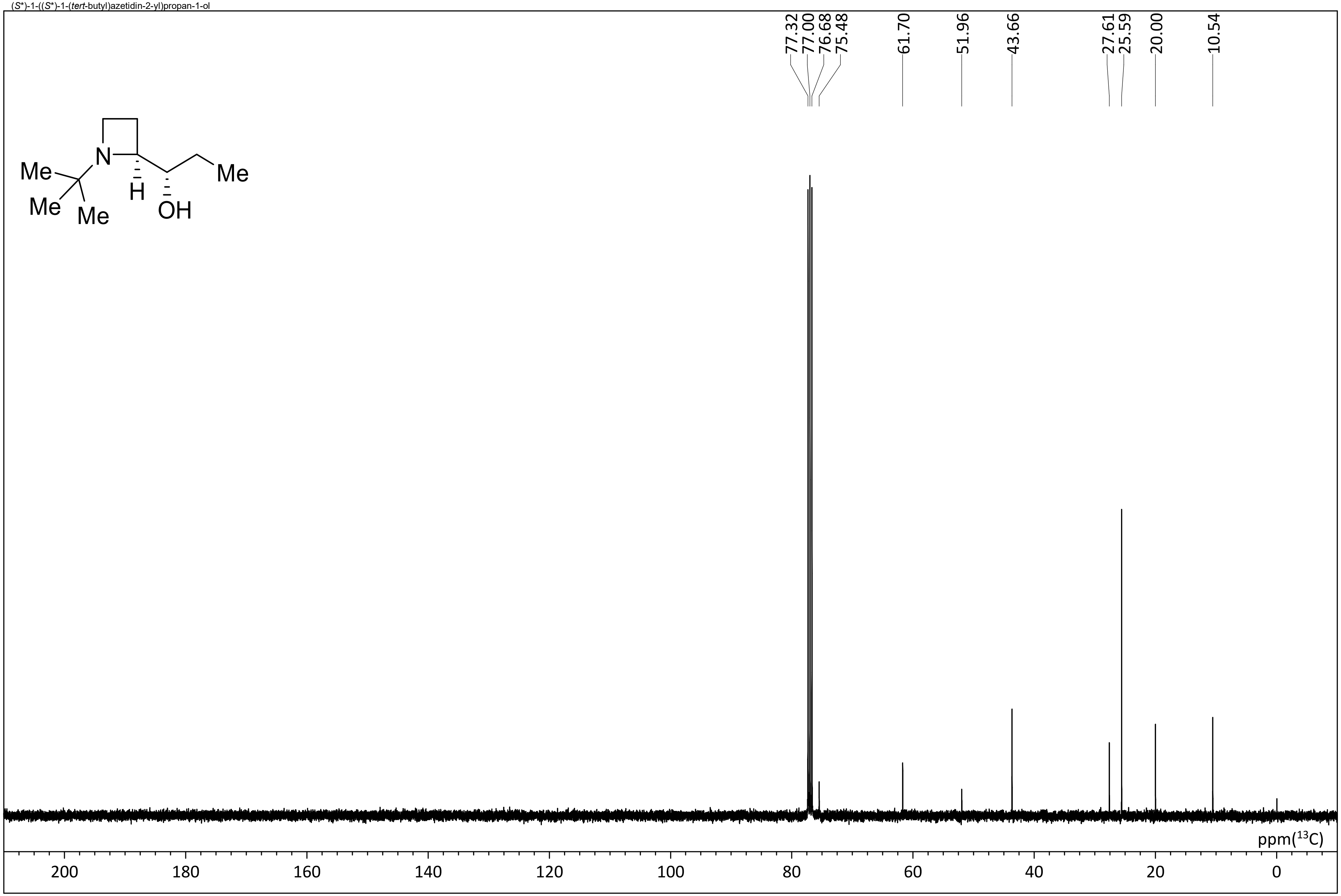

(*S**)-1-((*S**)-1-allylazetidin-2-yl) propan-1-ol

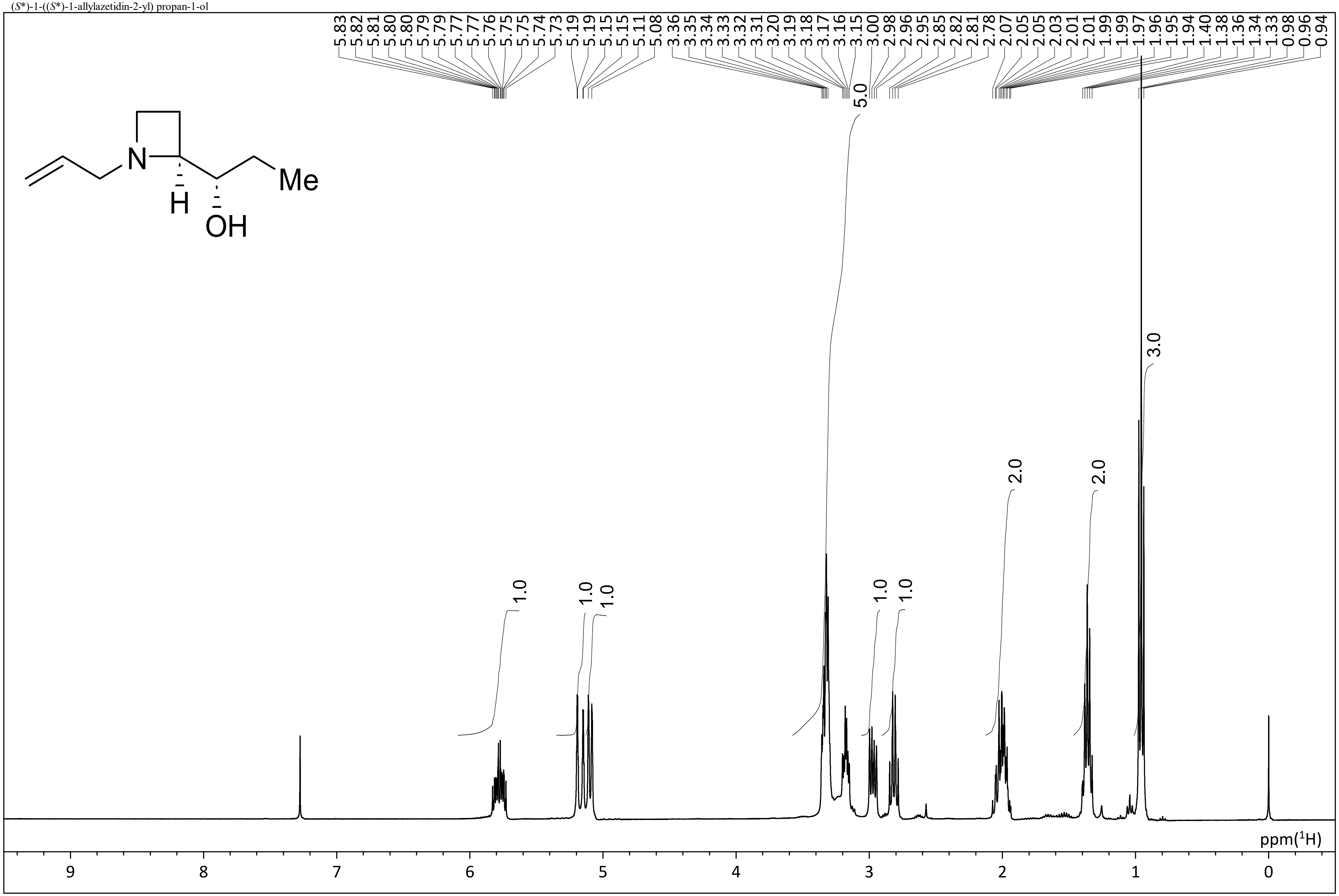

(*S**)-1-((*S**)-1-allylazetidin-2-yl)propan-1-ol

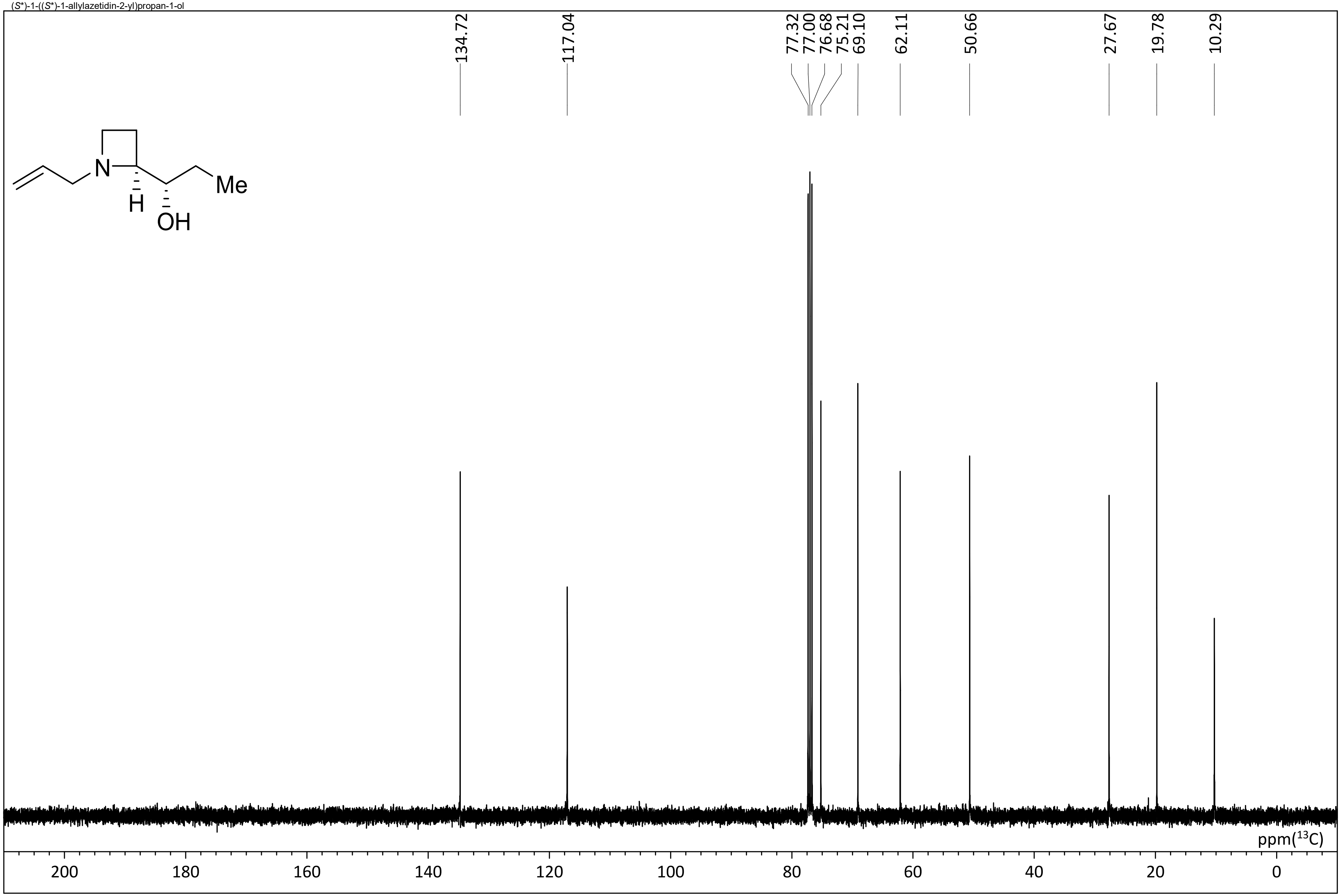

tert-butyl (5-((*S**)-2-((*S**)-1-hydroxypropyl)azetidin-1-yl)pentyl)carbamate

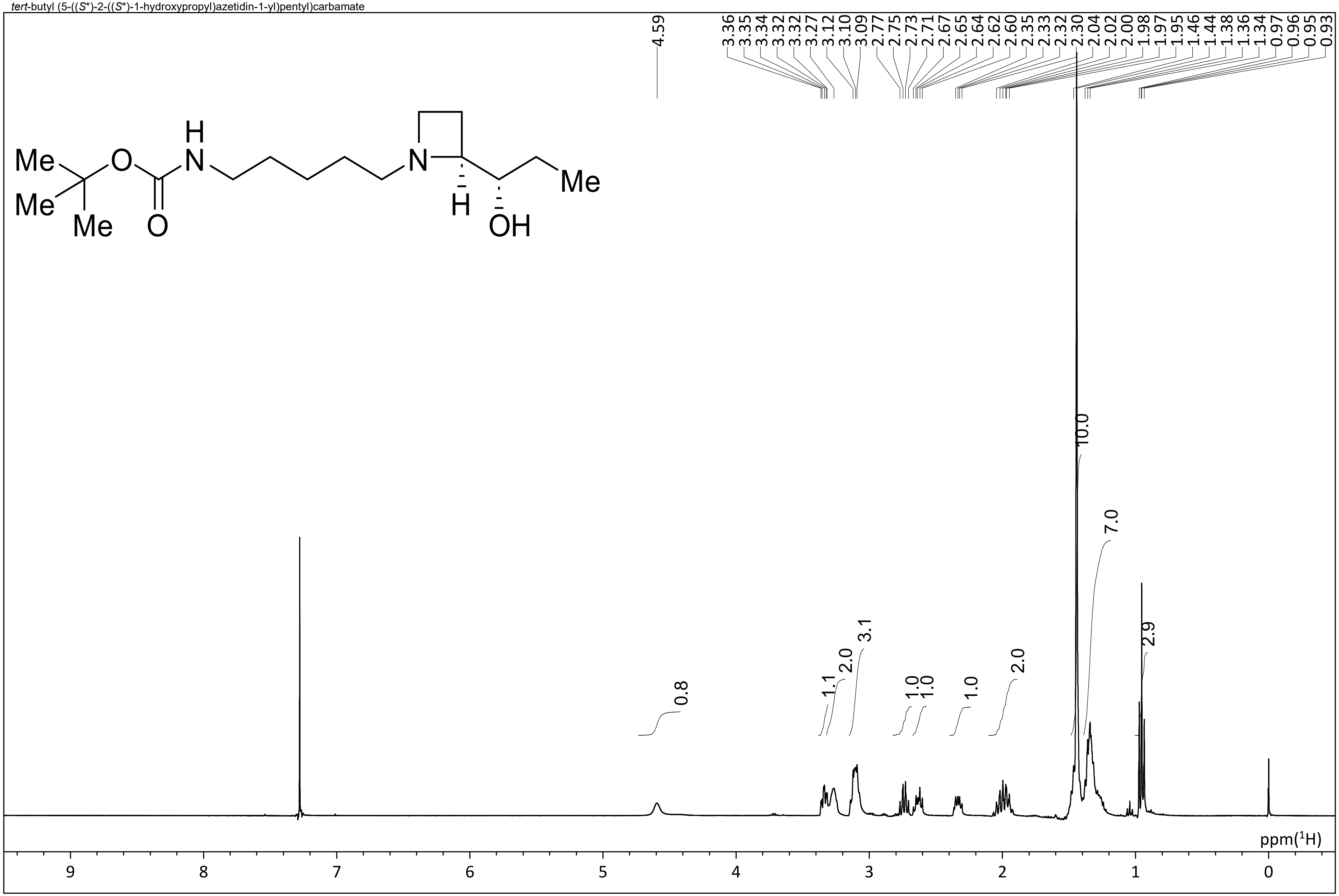

tert-butyl (5-((*S**)-2-((*S**)-1-hydroxypropyl)azetidin-1-yl)pentyl)carbamate

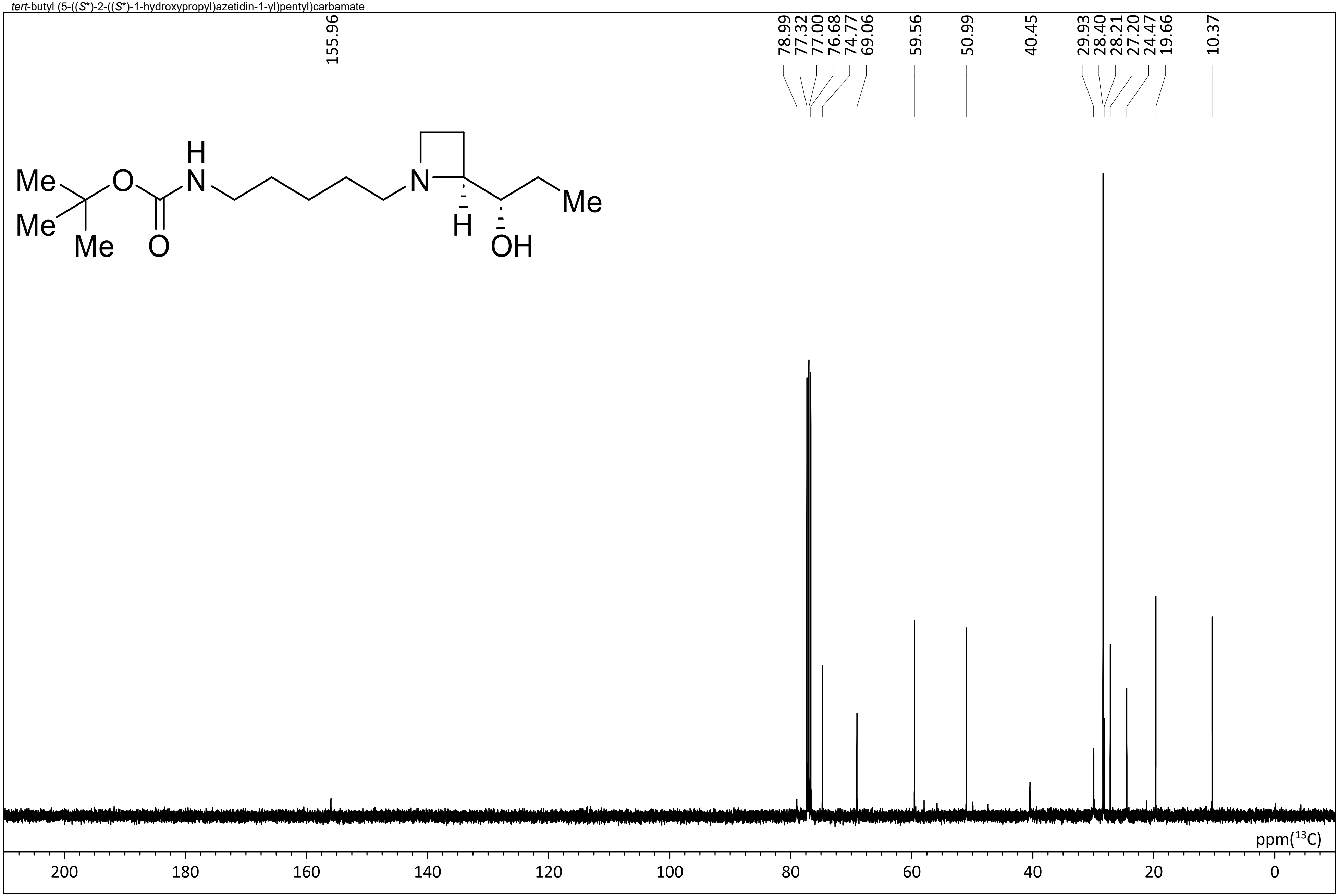

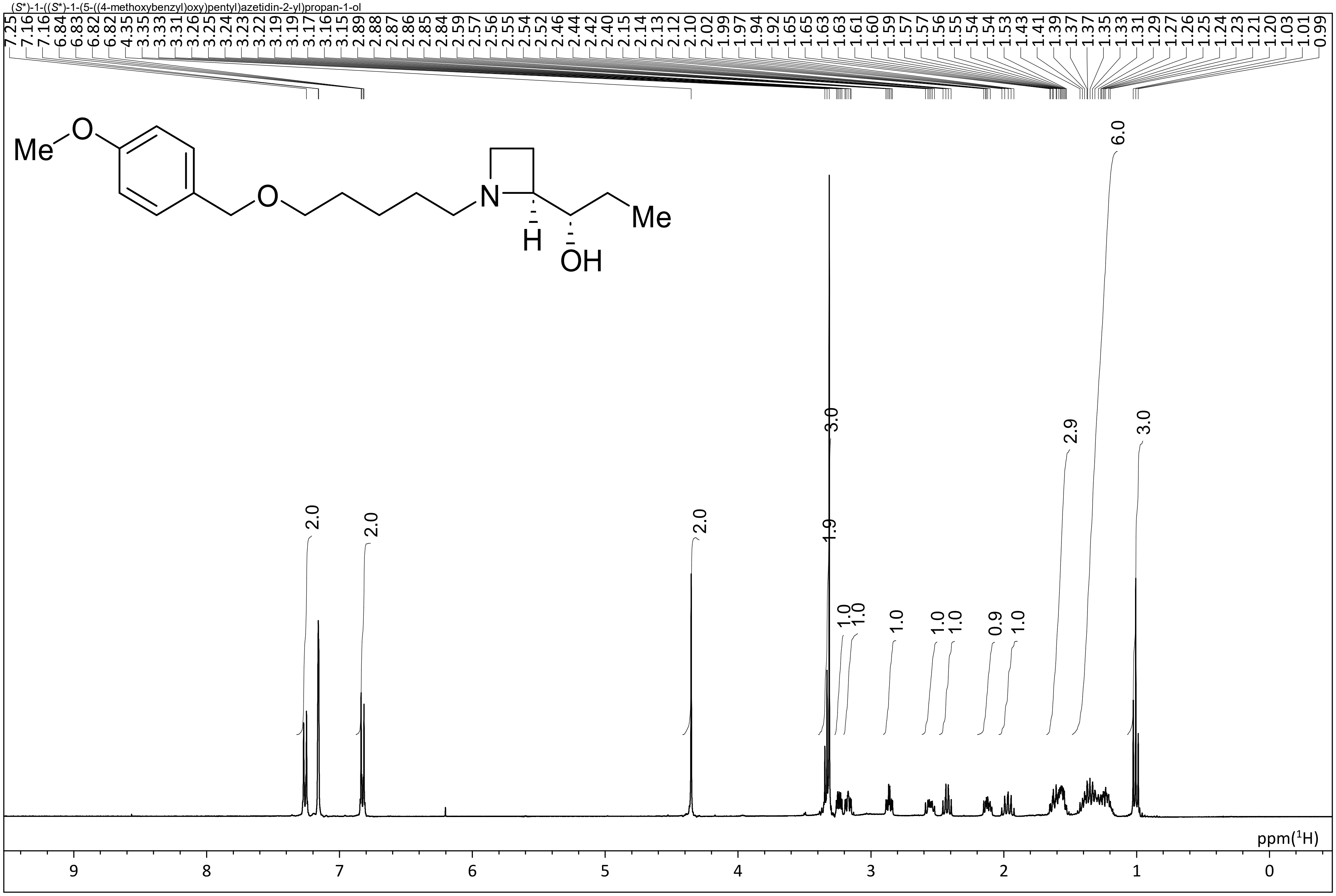
(S*)-1-((S*)-1-(5-((4-methoxybenzyl)oxy)pentyl)azetidin-2-yl)propan-1-ol
7.25 7.16 7.16 6.84 6.83 6.82 6.82 4.35 3.35 3.33 3.31 3.26 3.25 3.24 3.23 3.22 3.19 3.19 3.17 3.16 3.15 2.89 2.88 2.87 2.86 2.85 2.84 2.59 2.57 2.56 2.55 2.54 2.52 2.46 2.44 2.42 2.40 2.15 2.14 2.13 2.12 2.10 2.02 1.99 1.97 1.94 1.92 1.65 1.65 1.63 1.63 1.61 1.60 1.59 1.57 1.57 1.56 1.55 1.54 1.54 1.53 1.43 1.41 1.39 1.37 1.37 1.35 1.33 1.31 1.29 1.27 1.26 1.25 1.24 1.23 1.21 1.20 1.03 1.01 0.99
Me
O
O
N
H
OH
Me
2.0
2.0
2.0
3.0
1.9
1.0
1.0
1.0
1.0
1.0
0.9
1.0
2.9
6.0
3.0
ppm(1H)
9
8
7
6
5
4
3
2
1
0

(*S**)-1-((*S**)-1-(5-((4-methoxybenzyl)oxy)pentyl)azetidin-2-yl)propan-1-ol

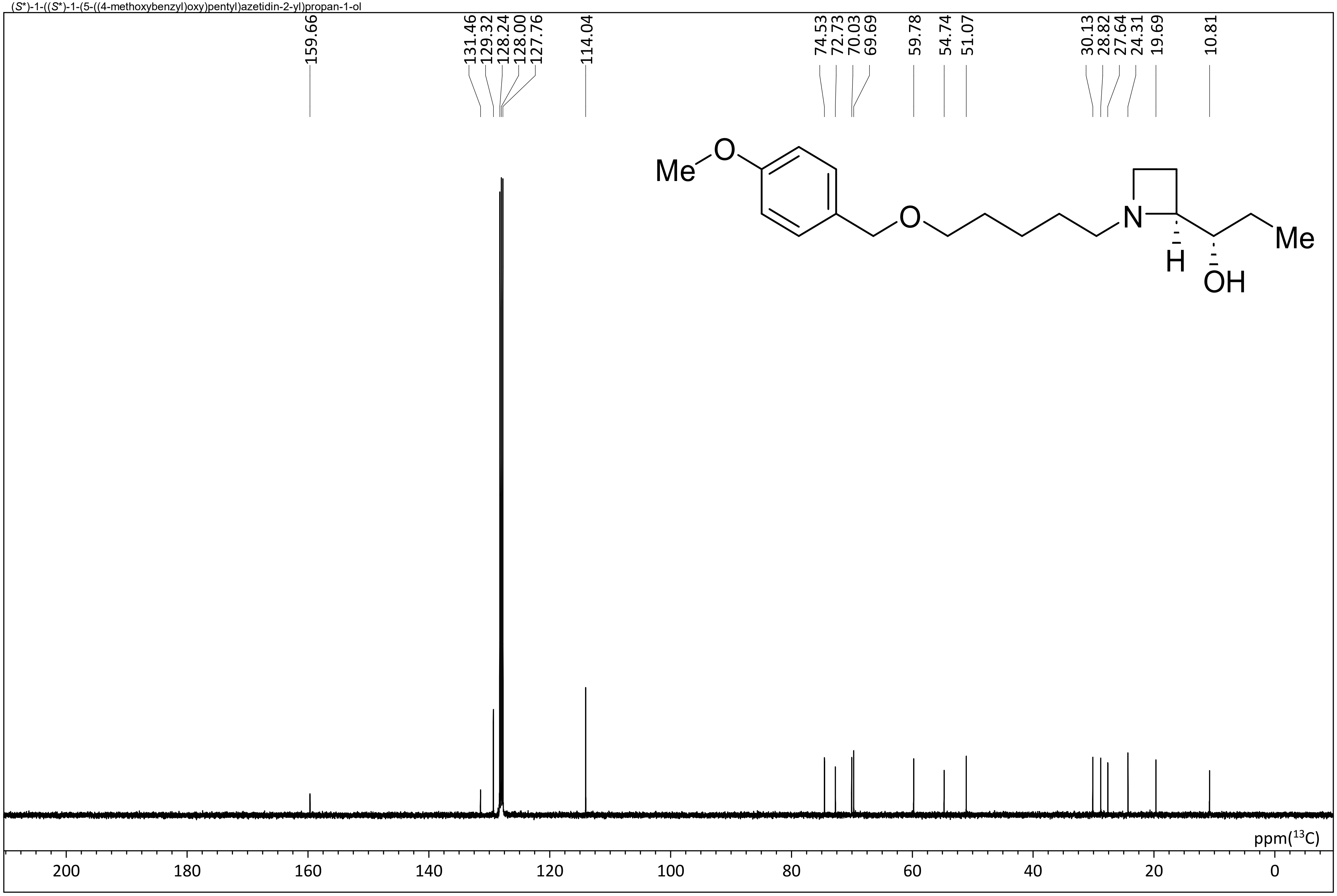

(*S**)-1-((*S**)-1-(3-((*tert*-butyldimethylsilyl)oxy)propyl)azetidin-2-yl)propan-1-ol

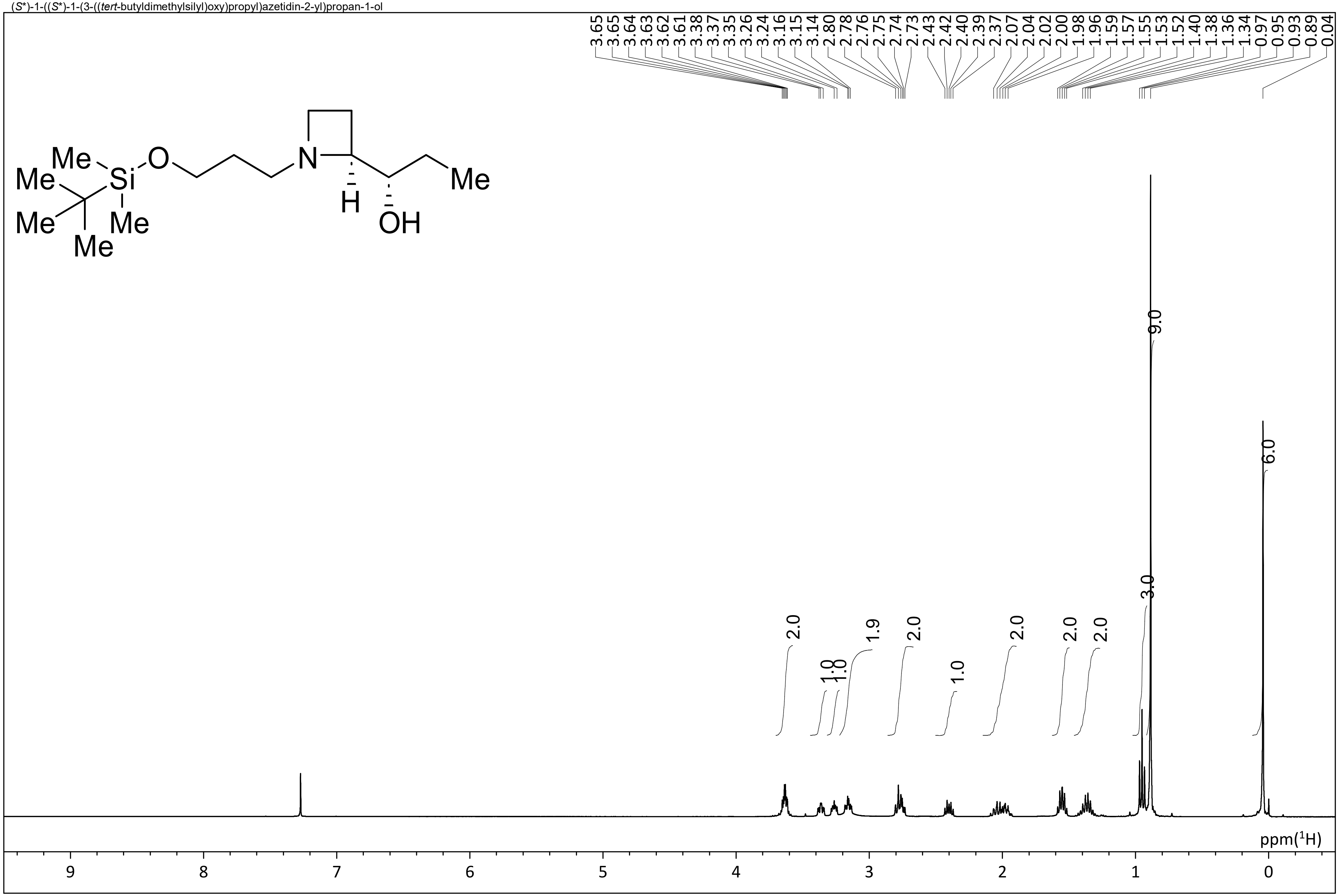

(*S**)-1-((*S**)-1-(3-((*tert*-butyldimethylsilyl)oxy)propyl)azetidin-2-yl)propan-1-ol

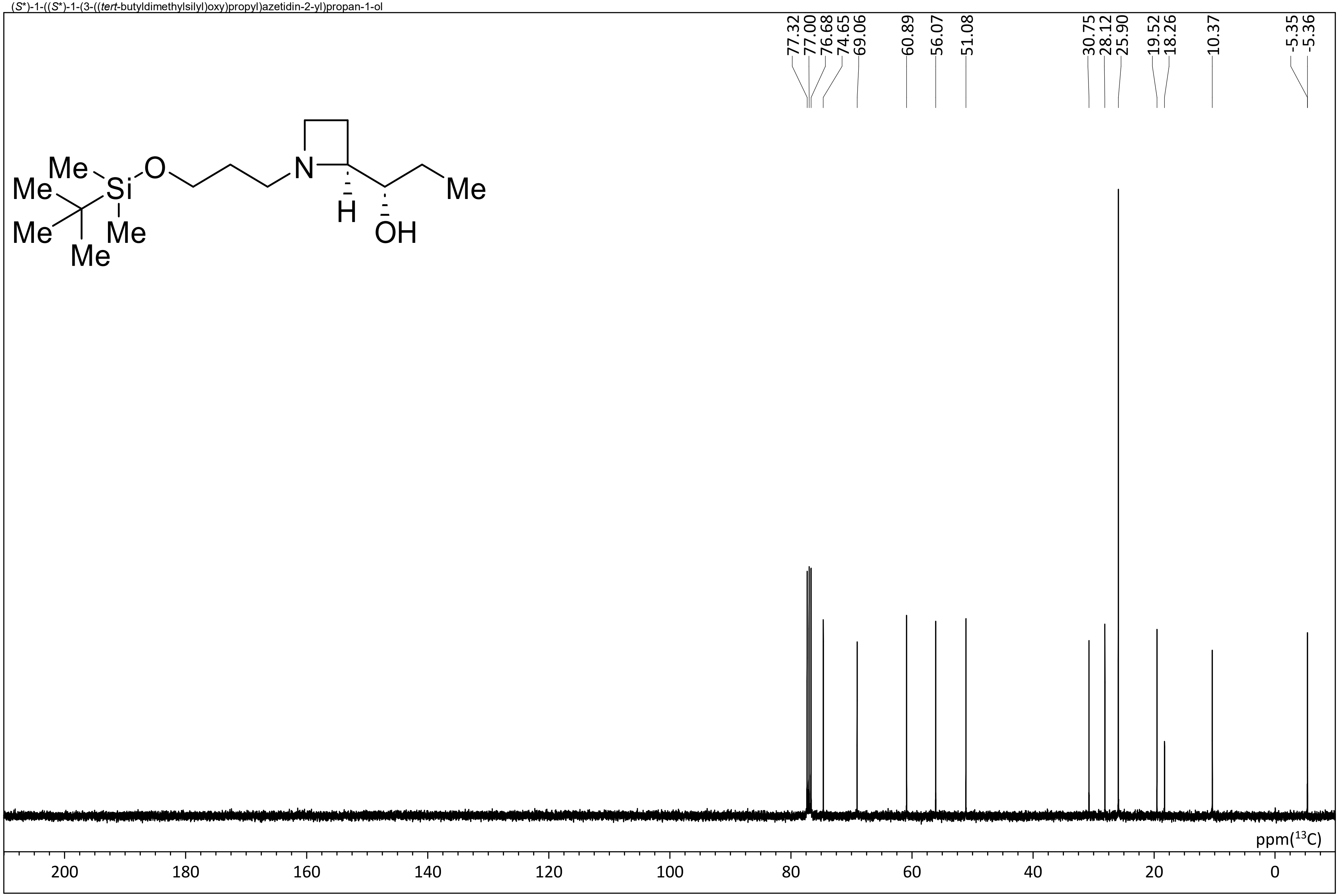

3-((*S**)-2-((*S**)-1-hydroxypropyl)azetidin-1-yl)propanenitrile

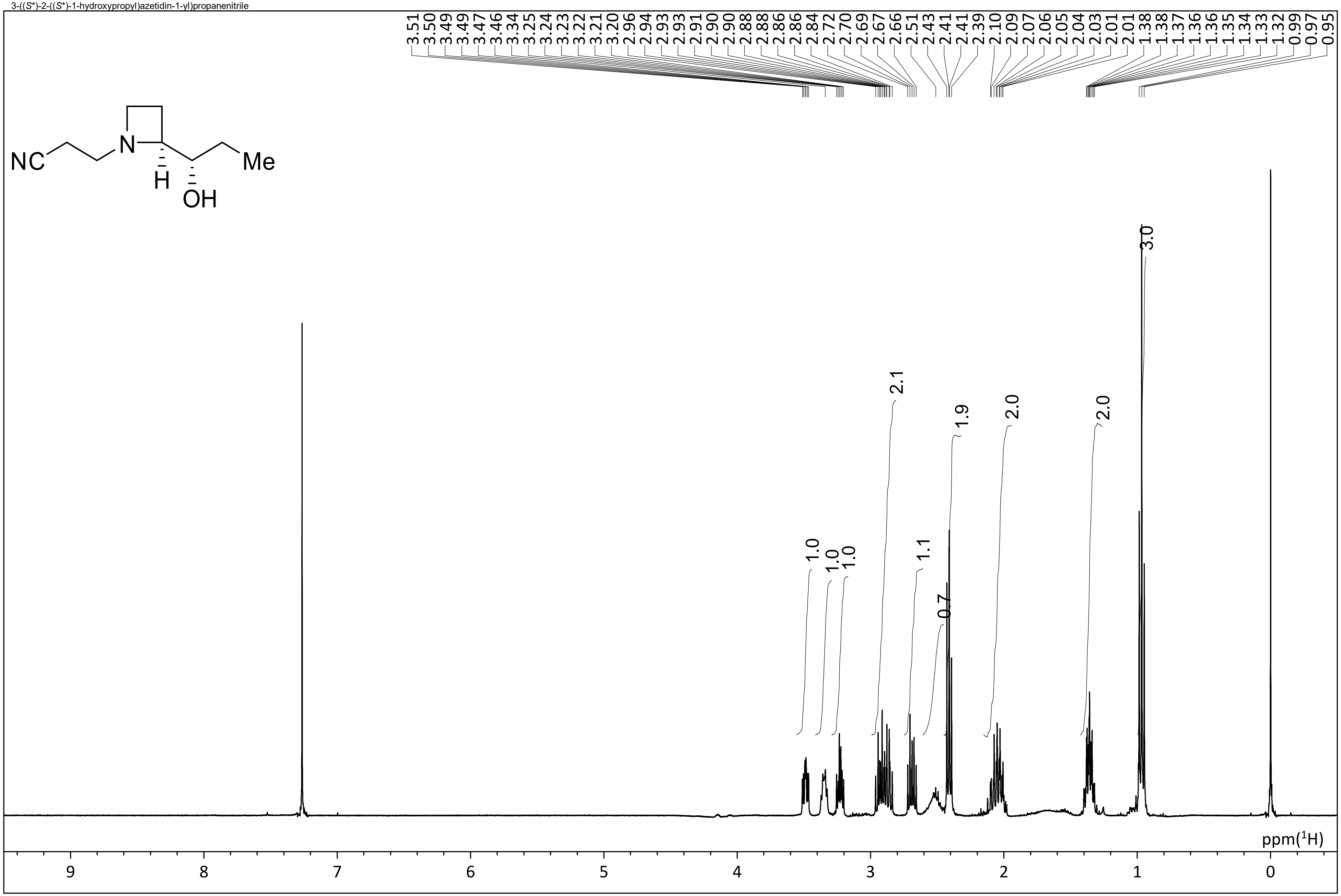

3-((*S**)-2-((*S**)-1-hydroxypropyl)azetidin-1-yl)propanenitrile

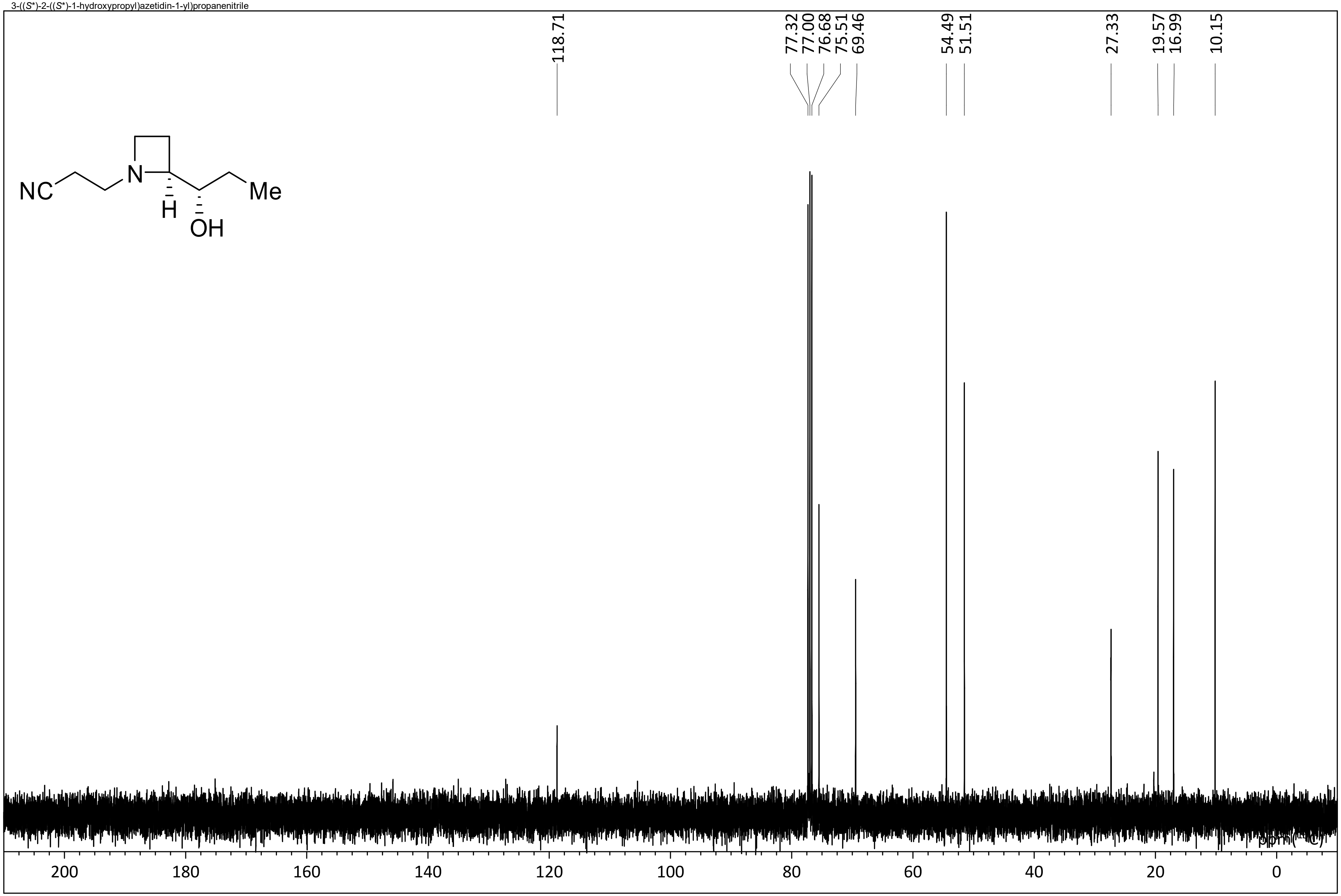

(S*)-1-((S*)-1-(3-(methylthio)propyl)azetidin-2-yl)propan-1-ol

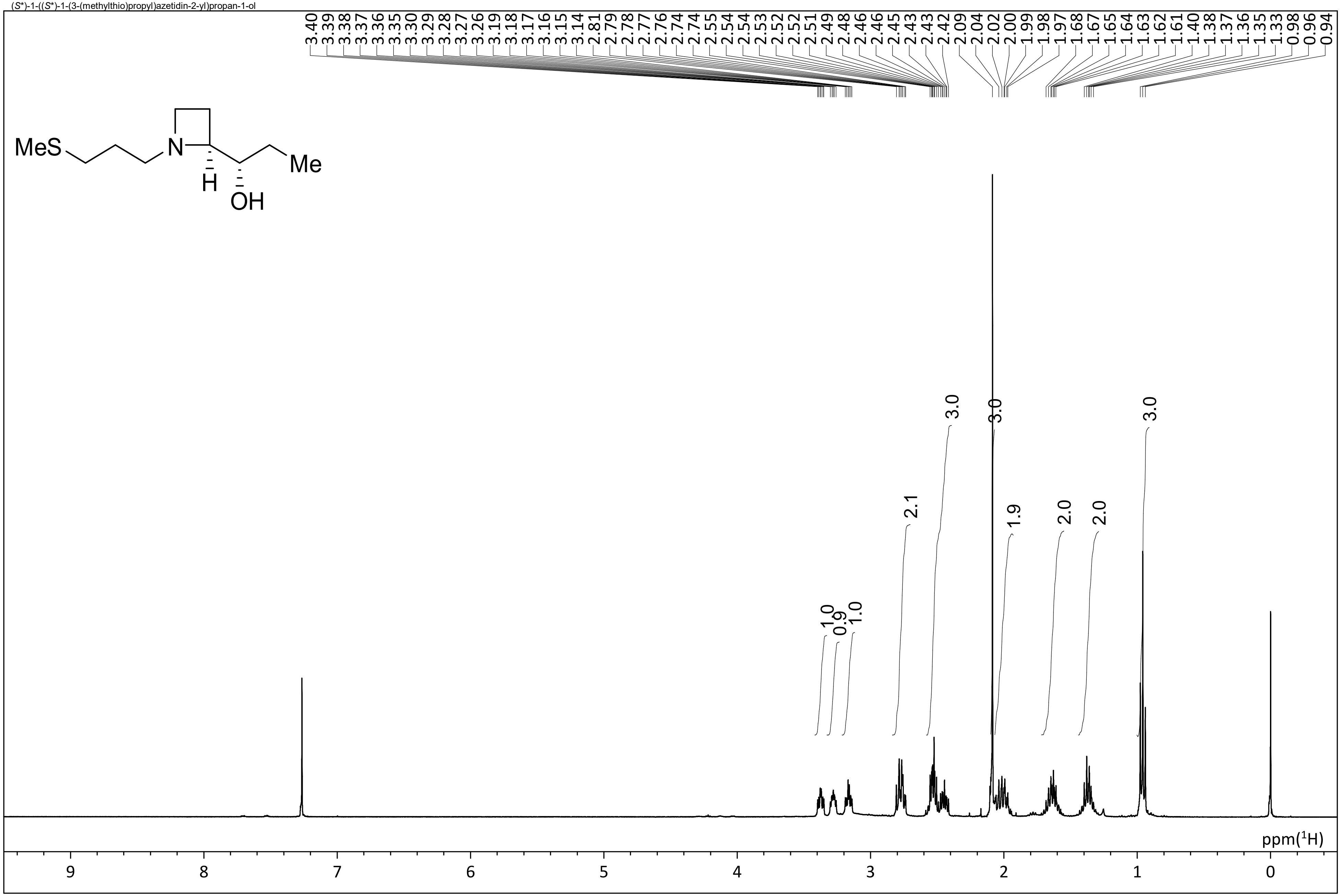

(*S**)-1-((*S**)-1-(3-(methylthio)propyl)azetidin-2-yl)propan-1-ol

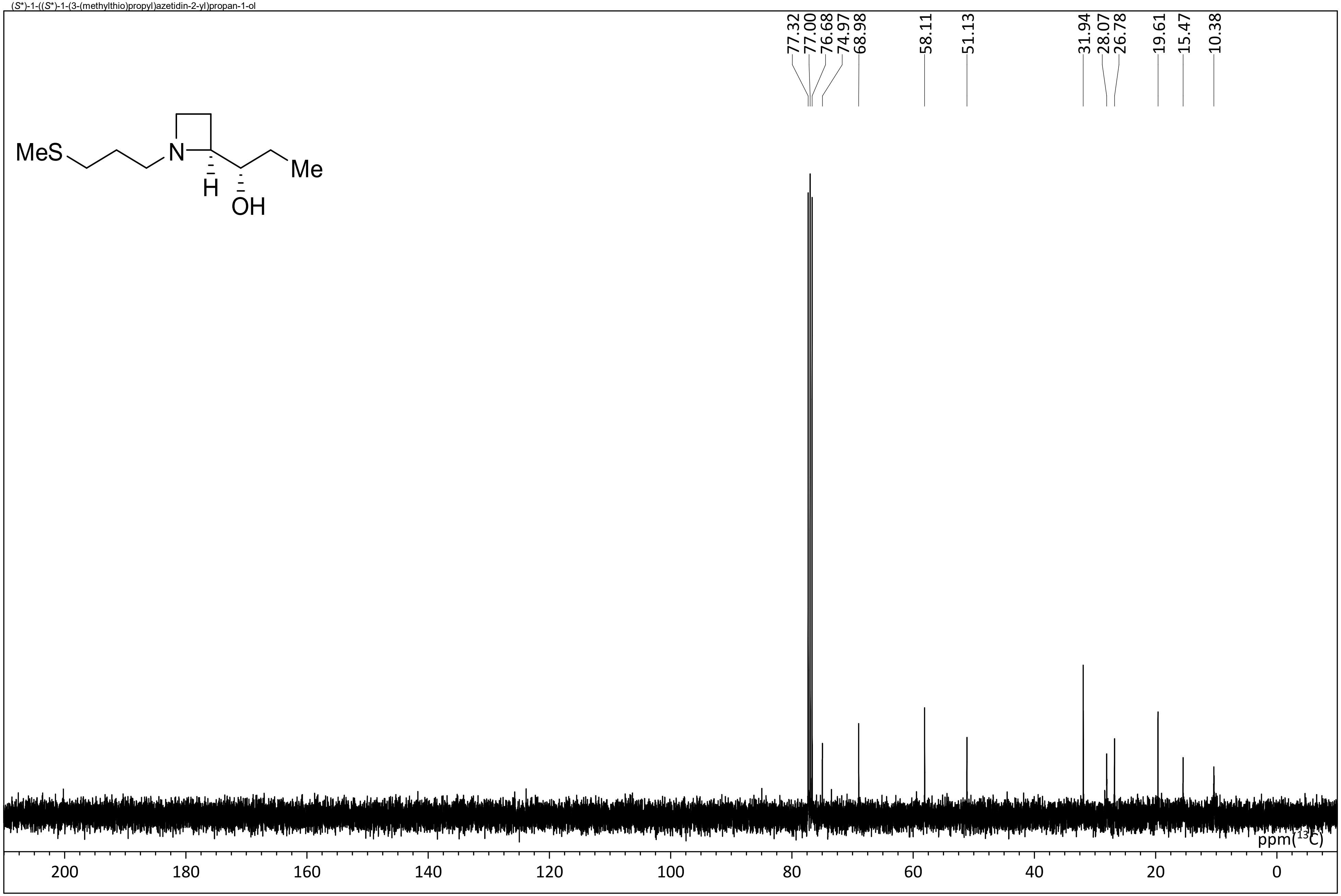

(*S**)-1-((*S**)-1-phenylazetidin-2-yl)propan-1-ol

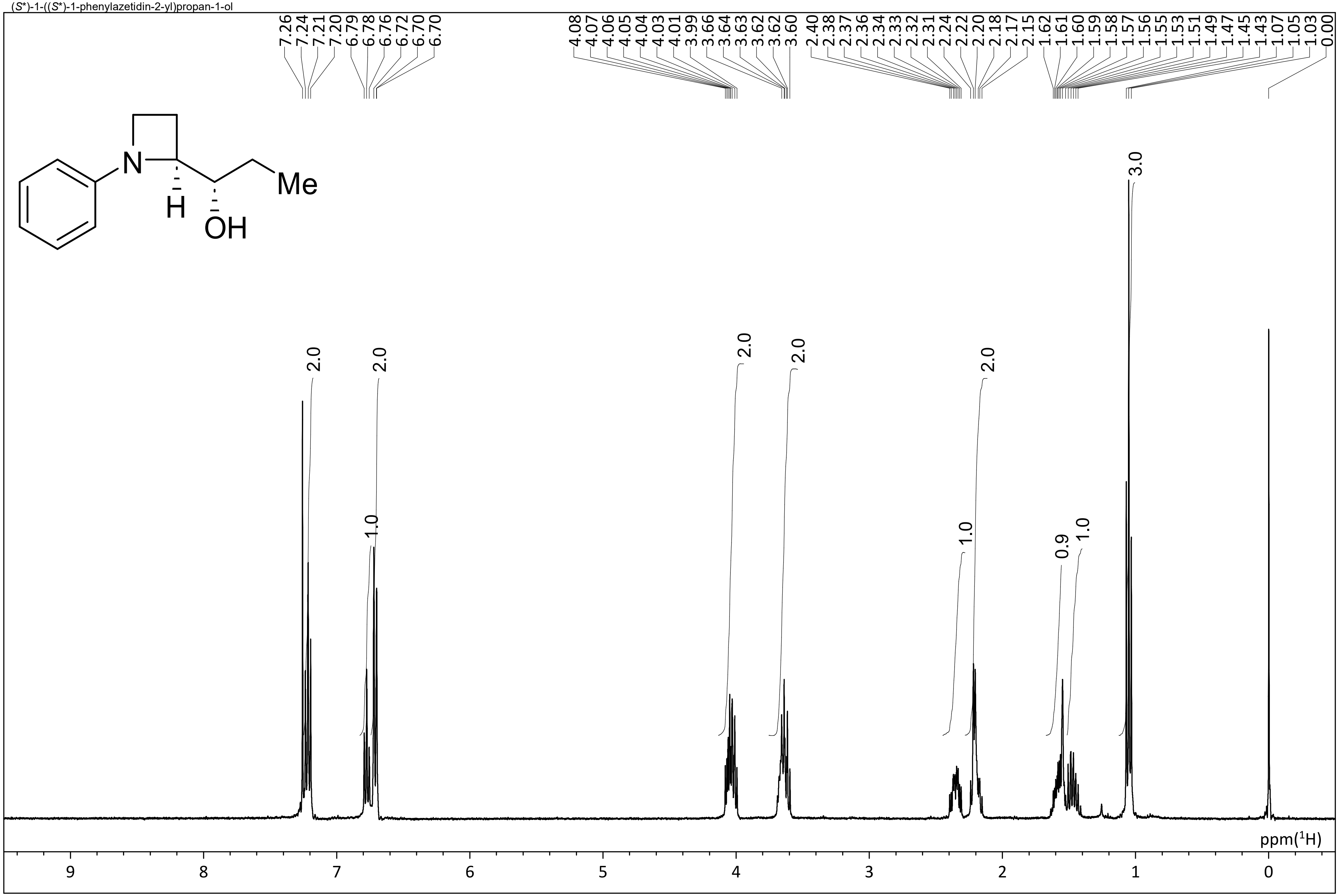

(*S**)-1-((*S**)-1-phenylazetidin-2-yl)propan-1-ol

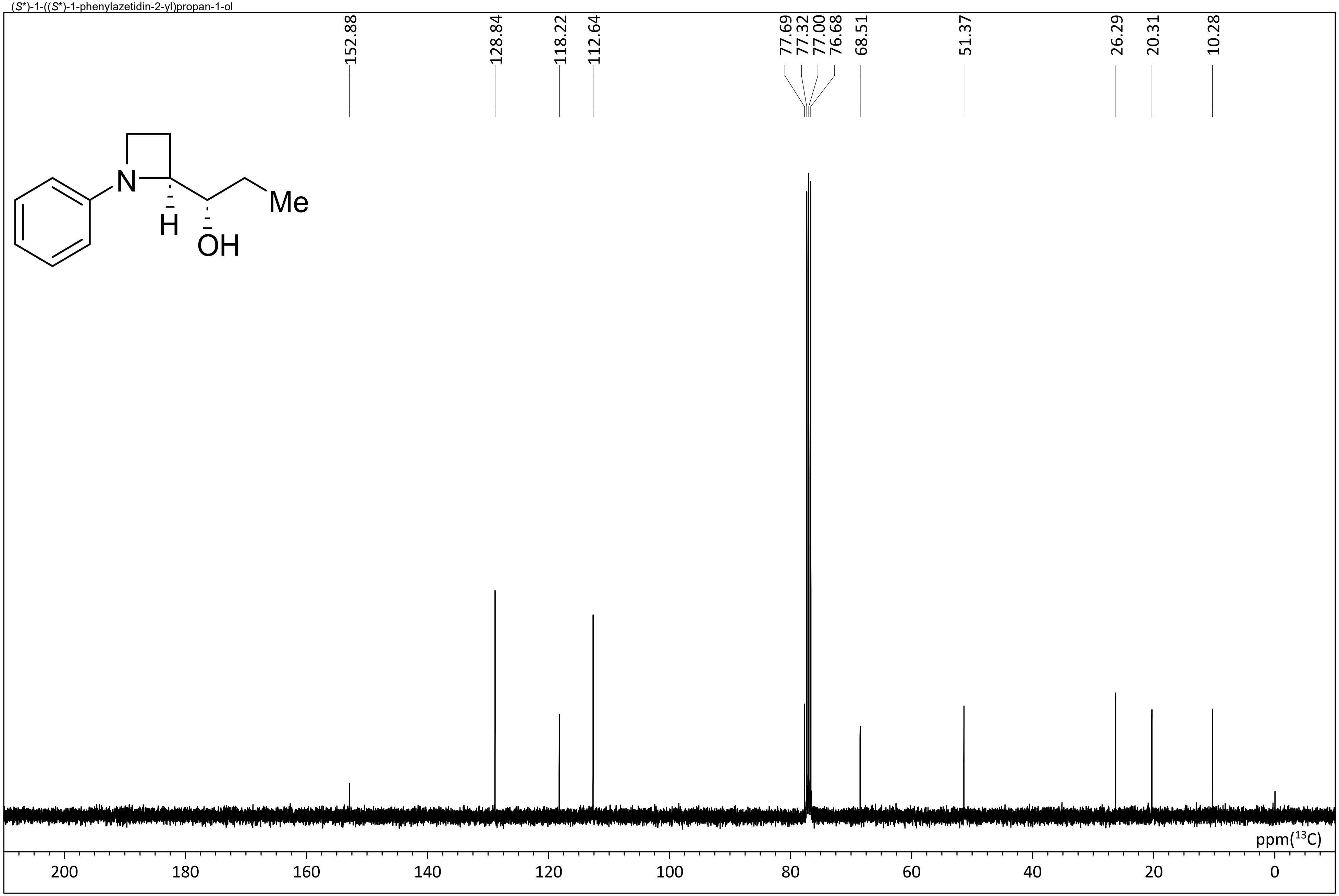

(2*R**, 3*R**)-2-ethyl-1-phenylpyrrolidin-3-ol

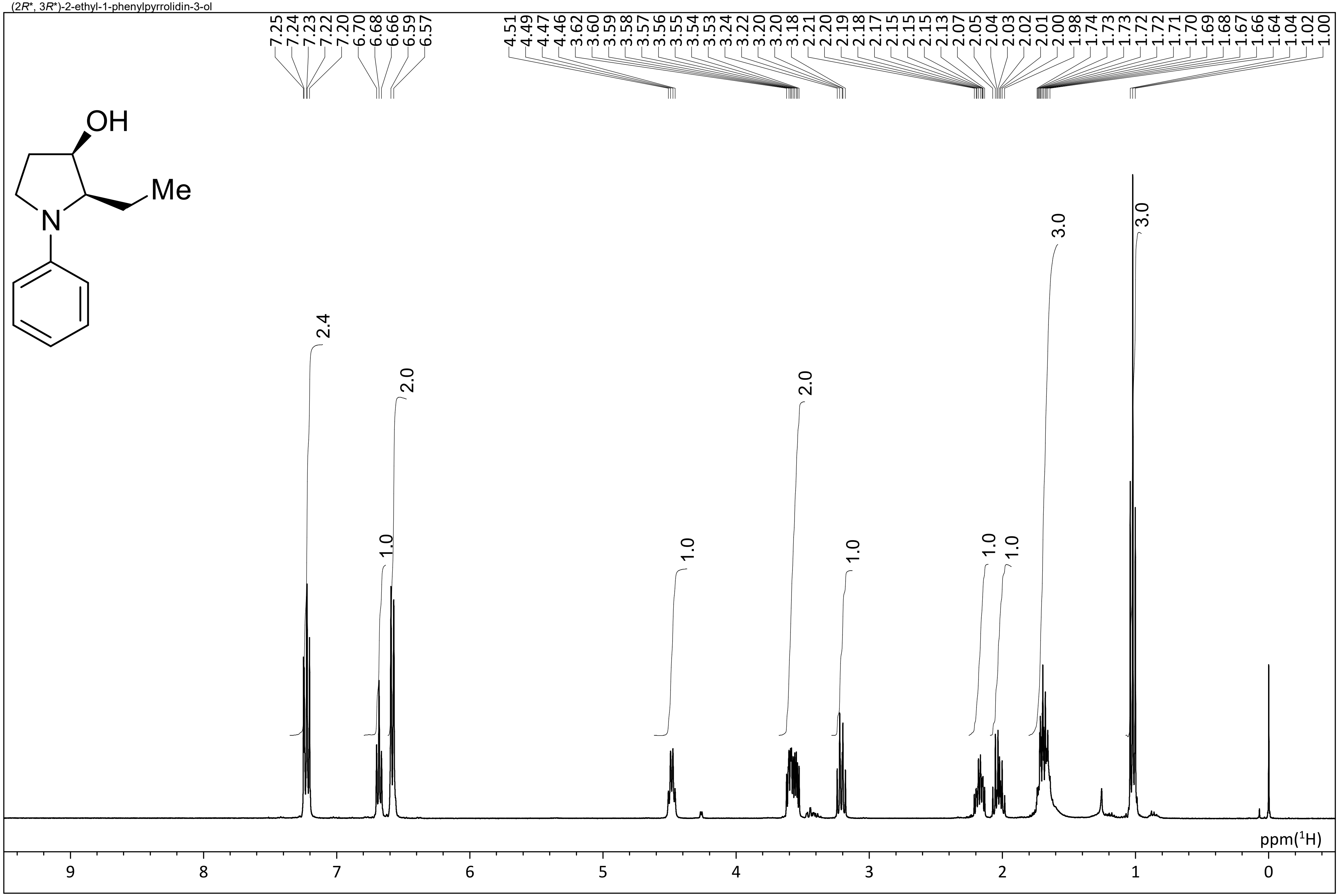

(2*R**, 3*R**)-2-ethyl-1-phenylpyrrolidin-3-ol

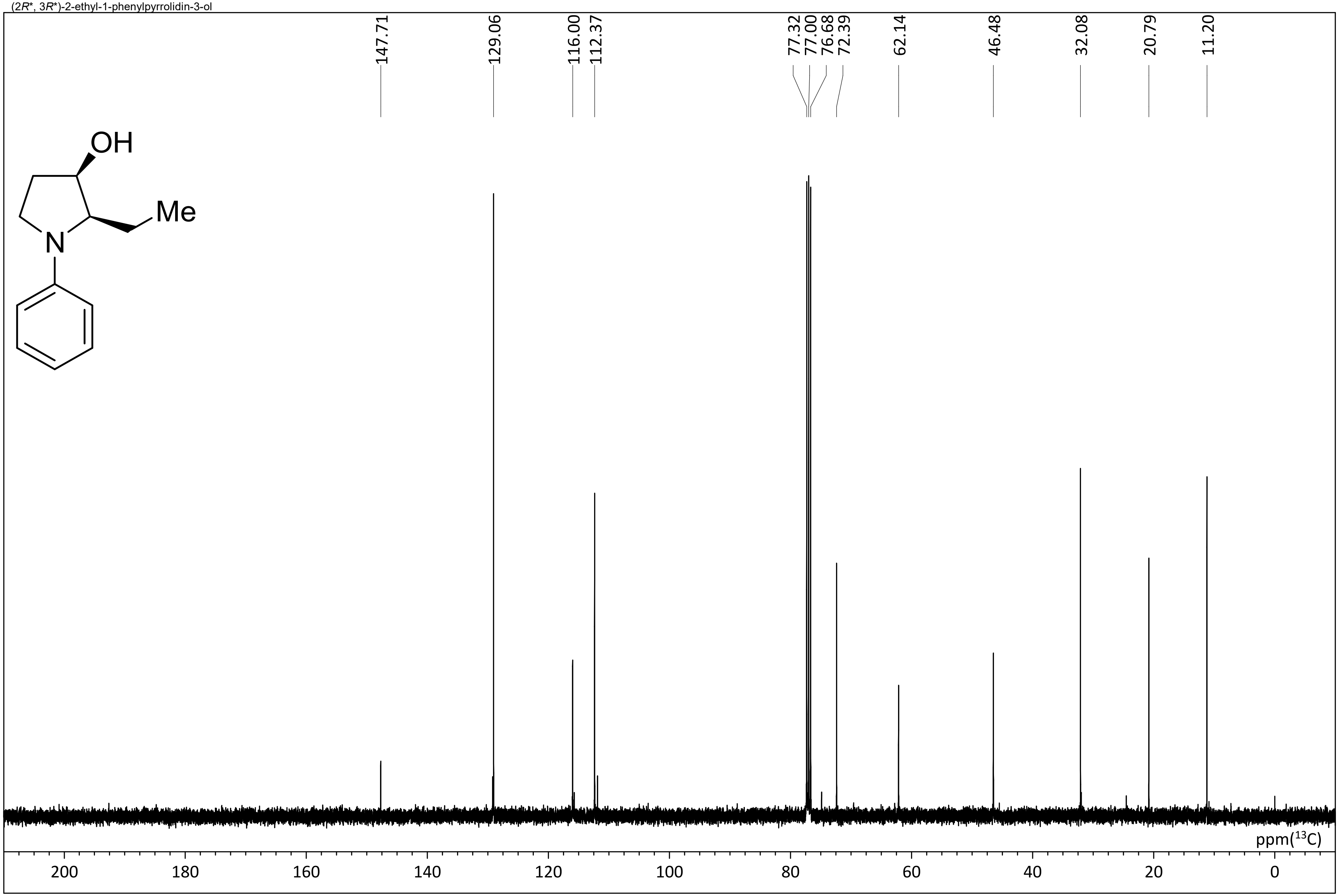

(*S**)-1-((*R**)-1,2,3,4-tetrahydroquinolin-4-yl)propan-1-ol

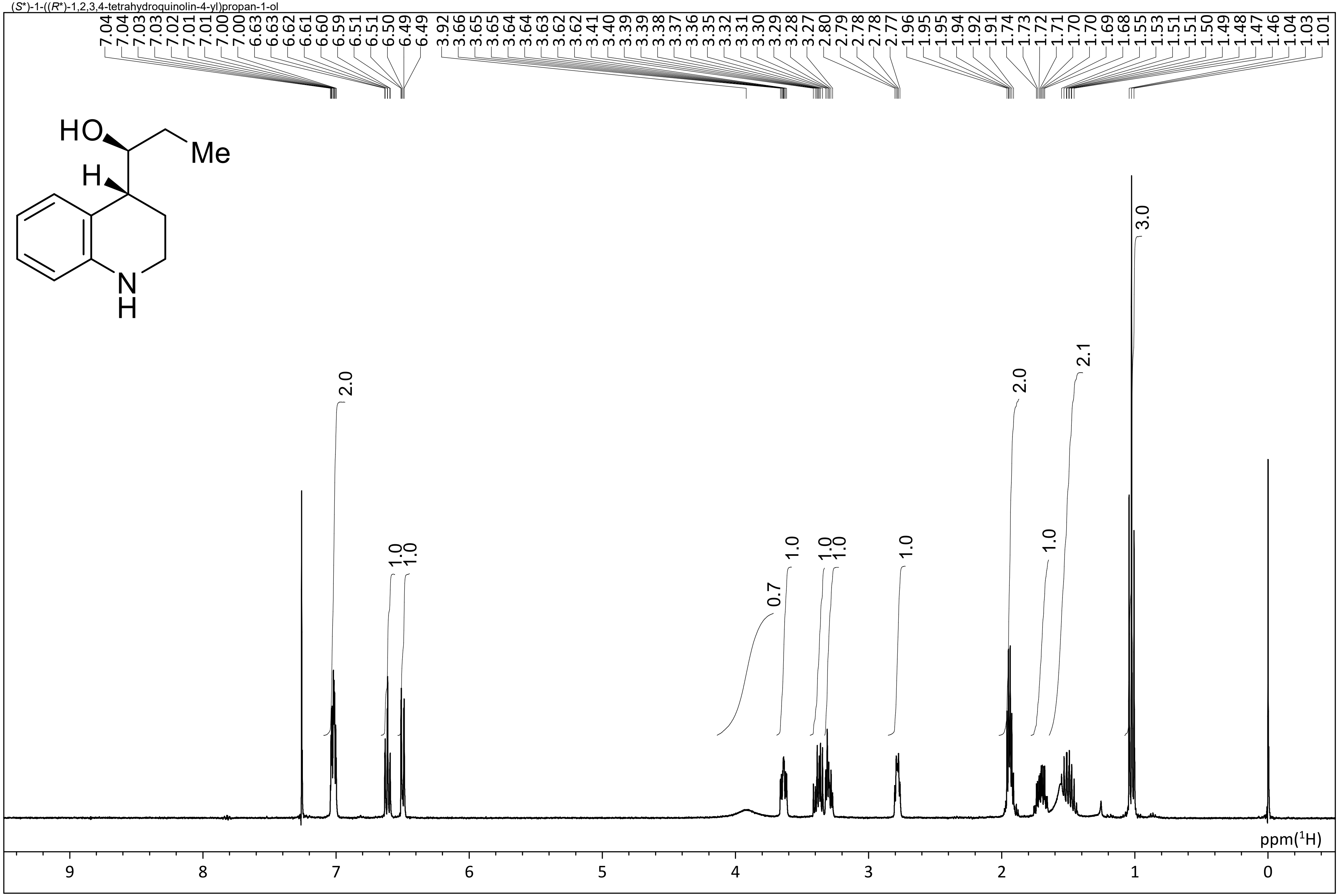

(*S**)-1-((*R**)-1,2,3,4-tetrahydroquinolin-4-yl)propan-1-ol

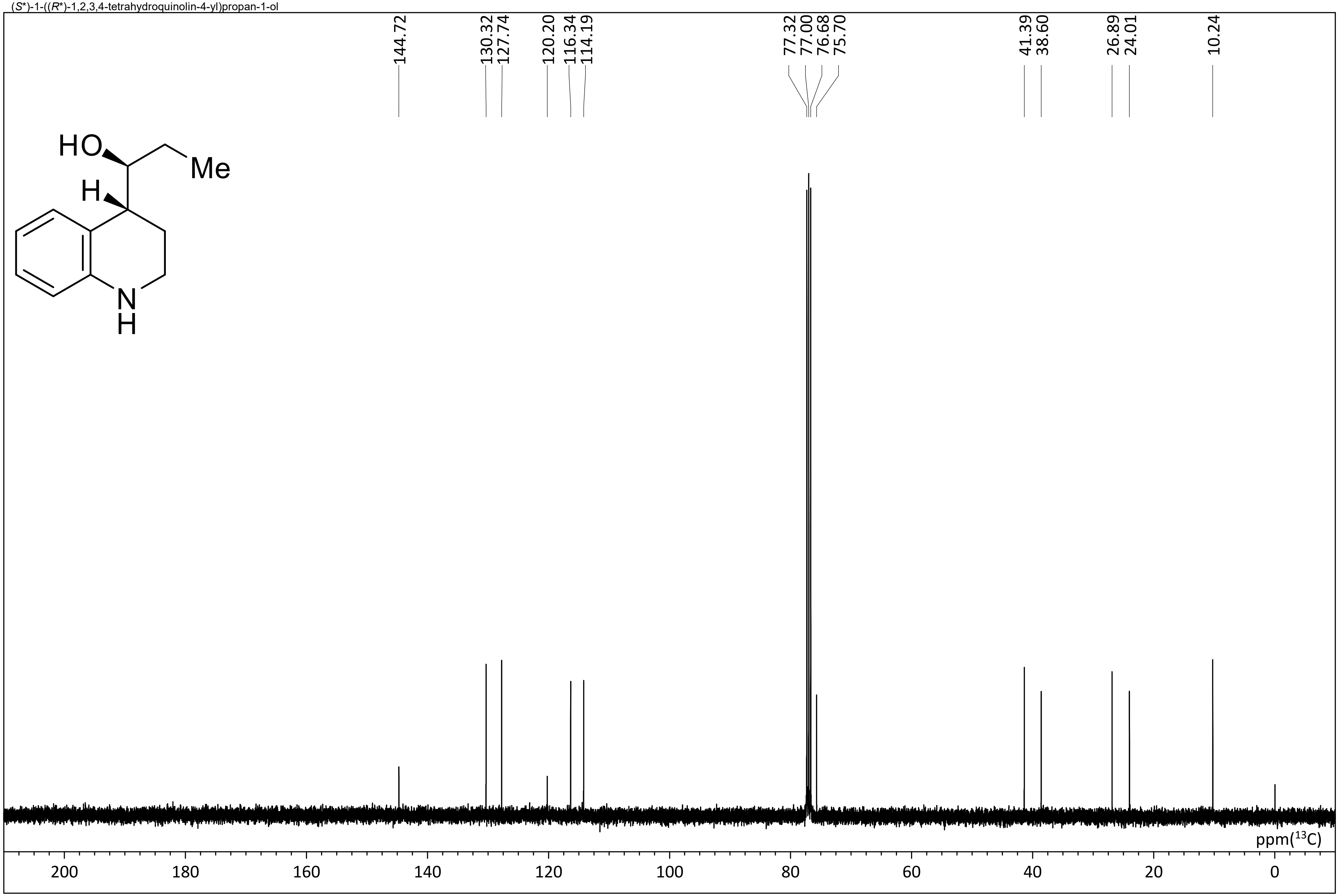

(S*)-1-((S*)-1-(4-methoxyphenyl)azetidin-2-yl)propan-1-ol

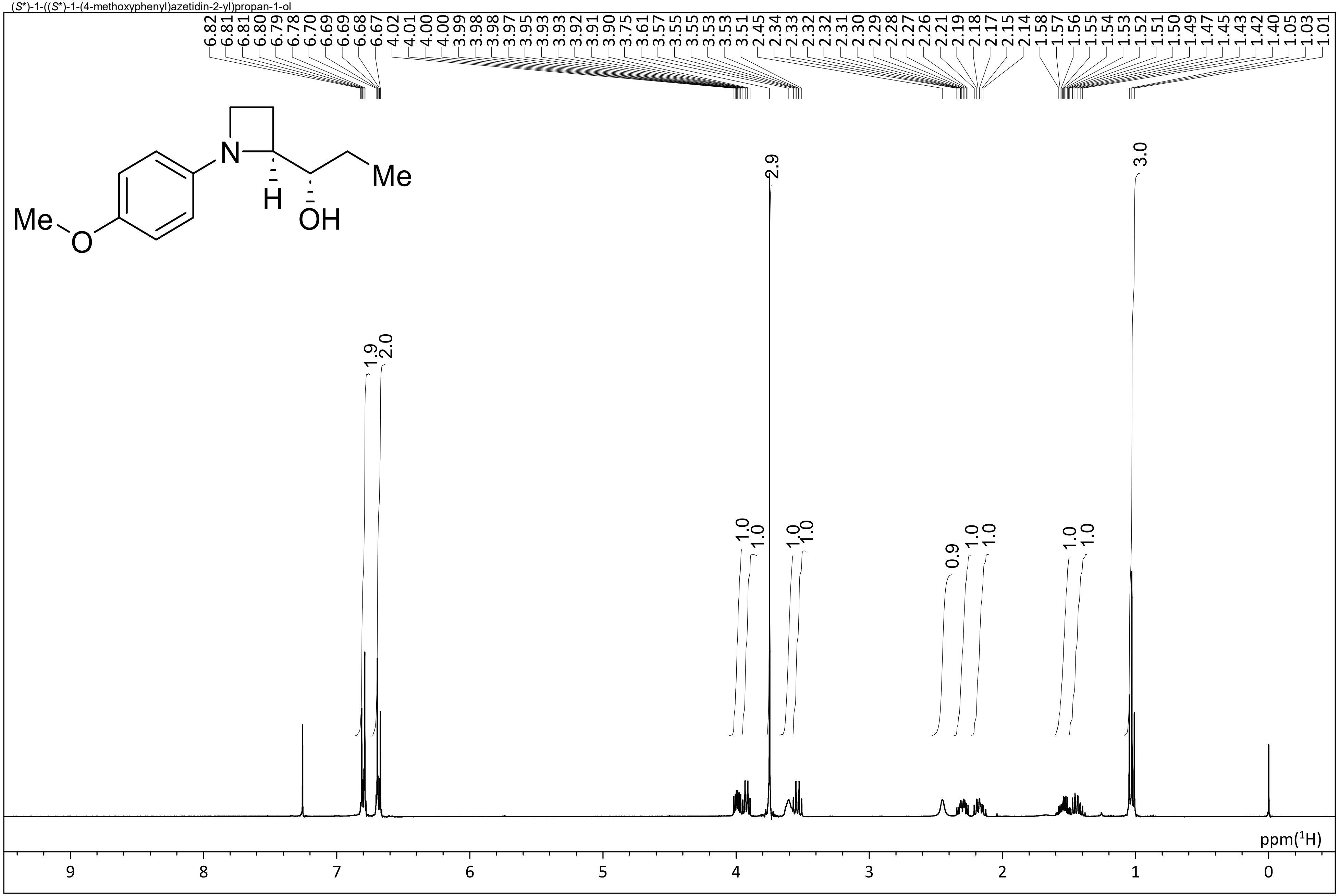

(*S**)-1-((*S**)-1-(4-methoxyphenyl)azetidin-2-yl)propan-1-ol

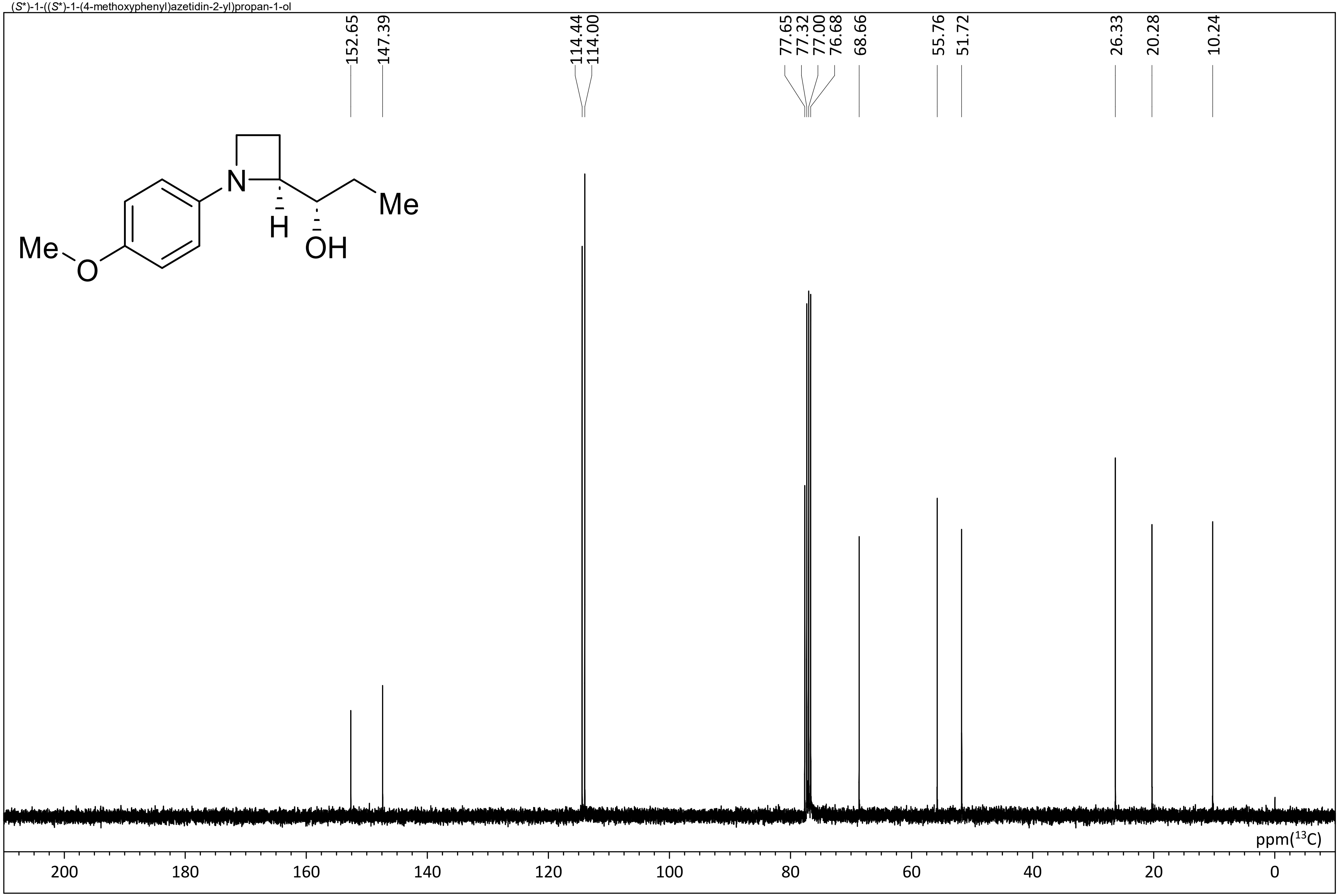

(2*R**, 3*R**)-2-ethyl-1-(4-methoxyphenyl)pyrrolidin-3-ol

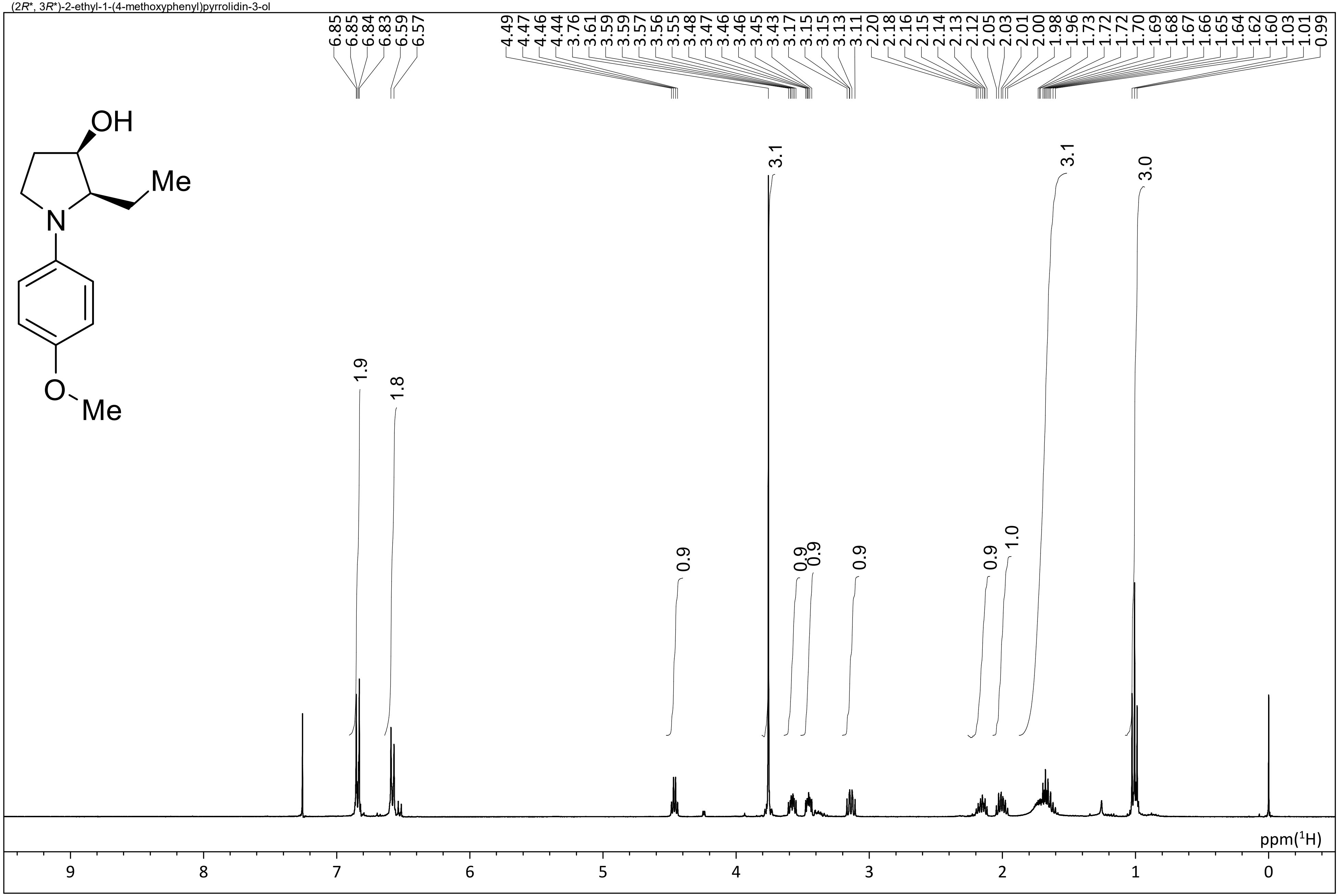

(2*R**, 3*R**)-2-ethyl-1-(4-methoxyphenyl)pyrrolidin-3-ol

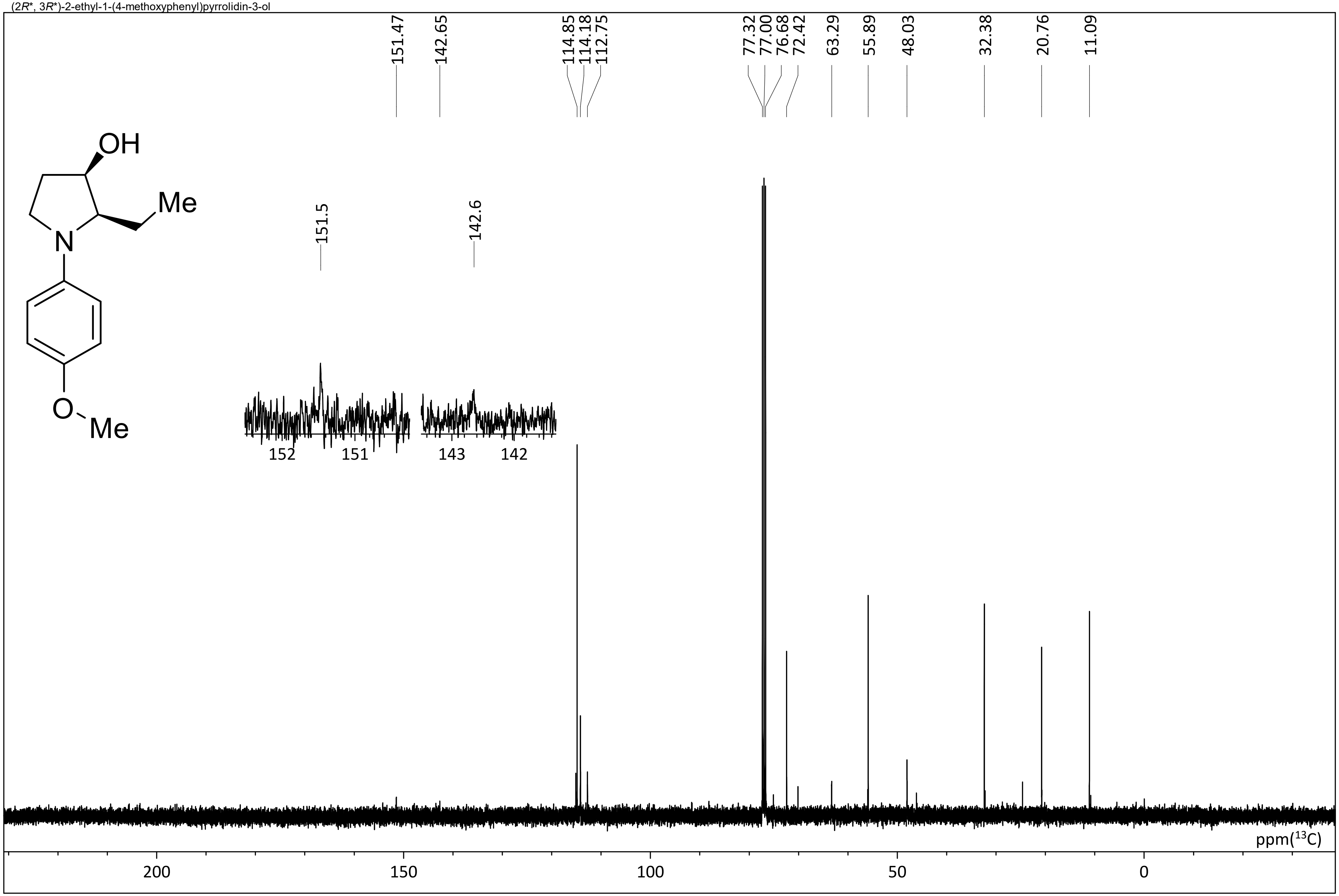

(*S**)-((*S**)-1-benzylazetidin-2-yl)(phenyl)methanol

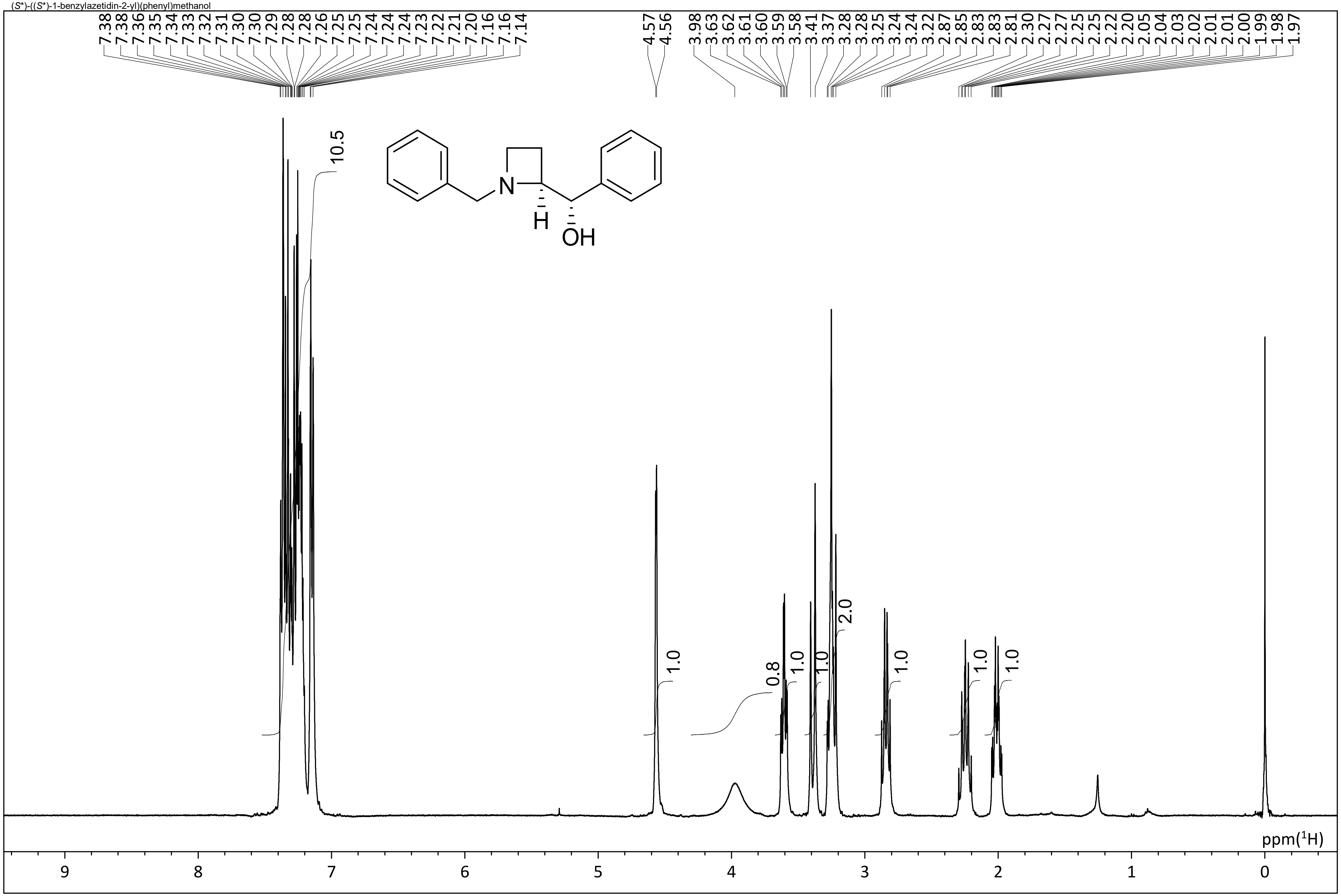

(*S**)-((*S**)-1-benzylazetidin-2-yl)(phenyl)methanol

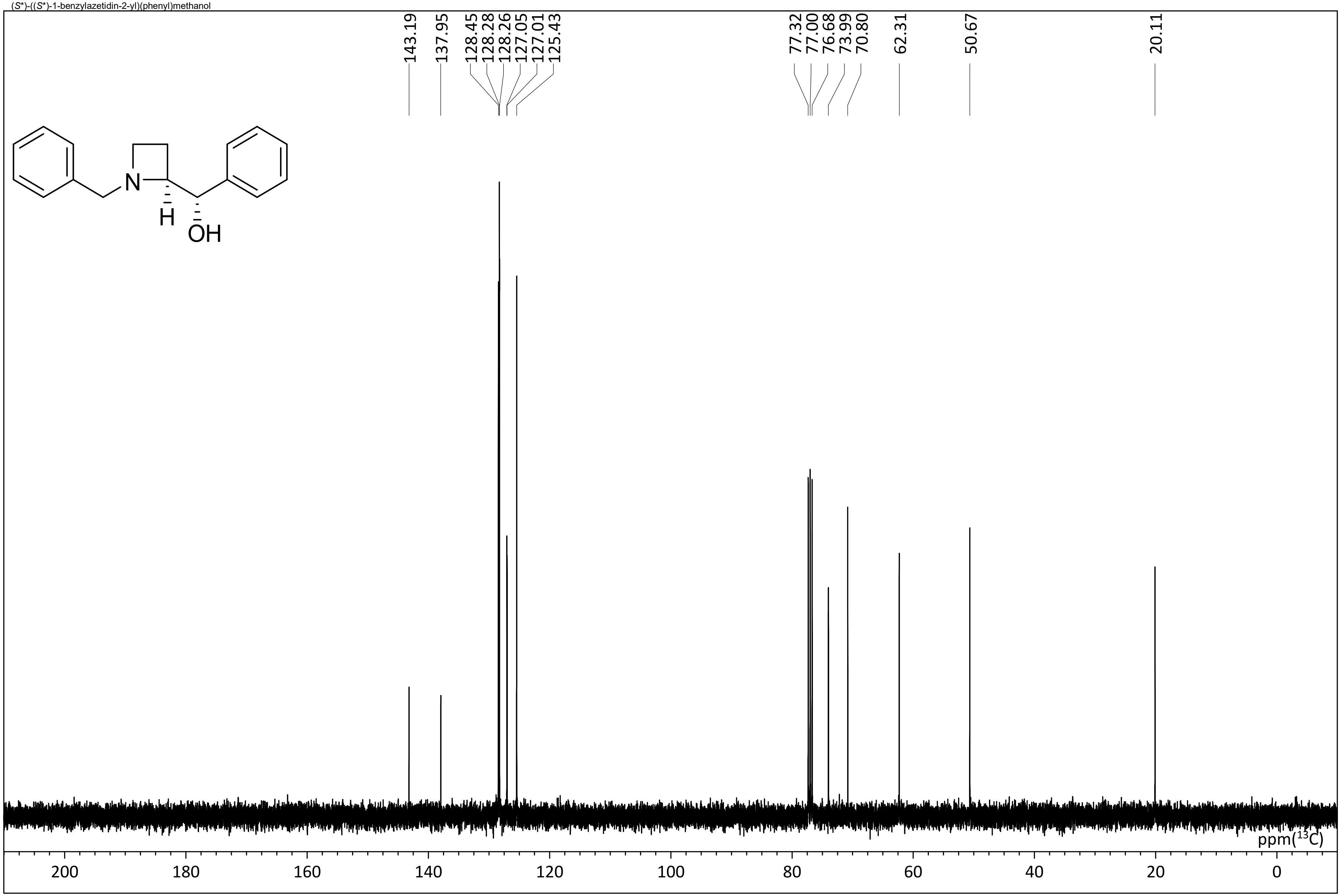

(*S**)-((*S**)-1-benzylazetidin-2-yl)(phenyl)methyl acetate

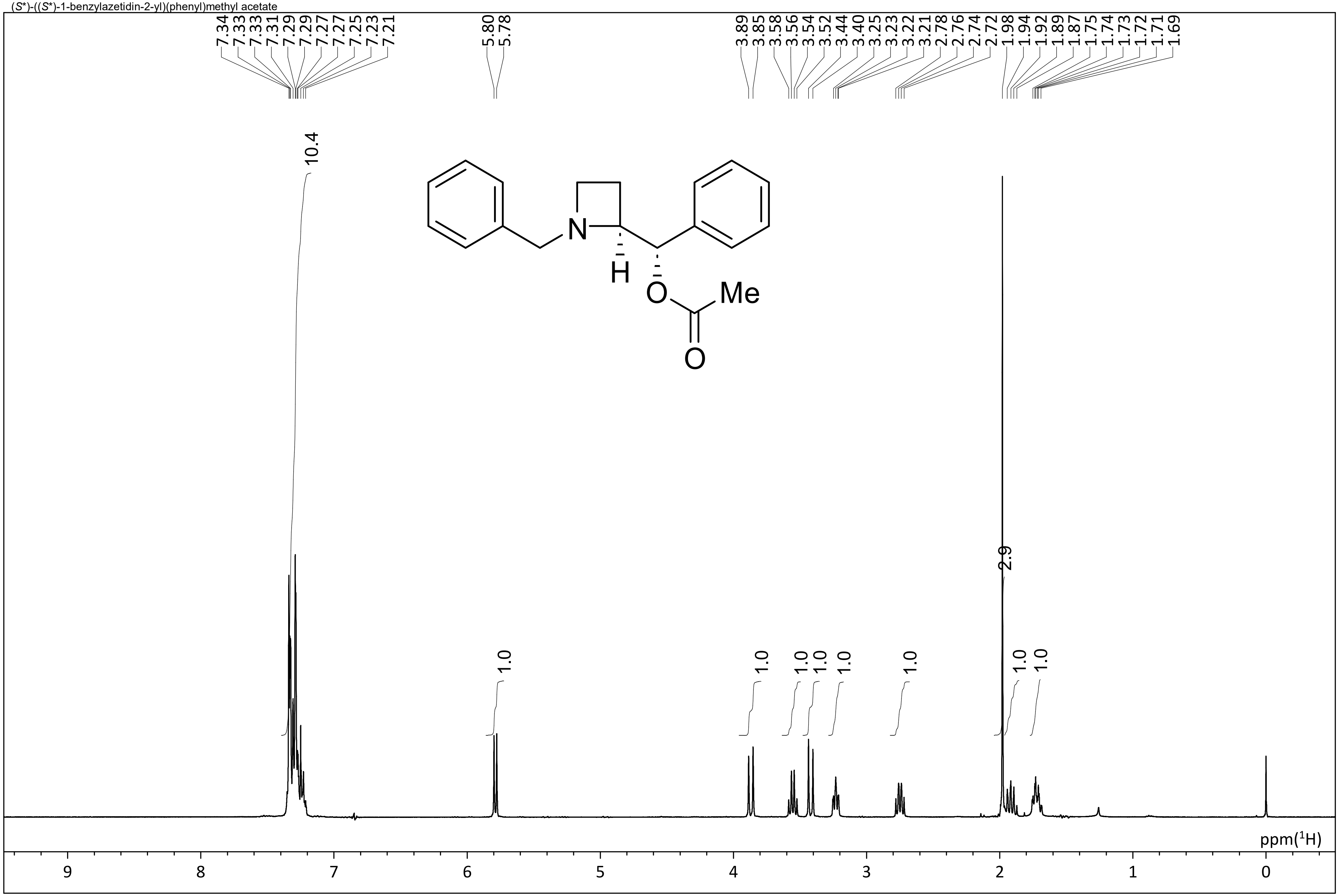

(*S**)-((*S**)-1-benzylazetidin-2-yl)(phenyl)methyl acetate

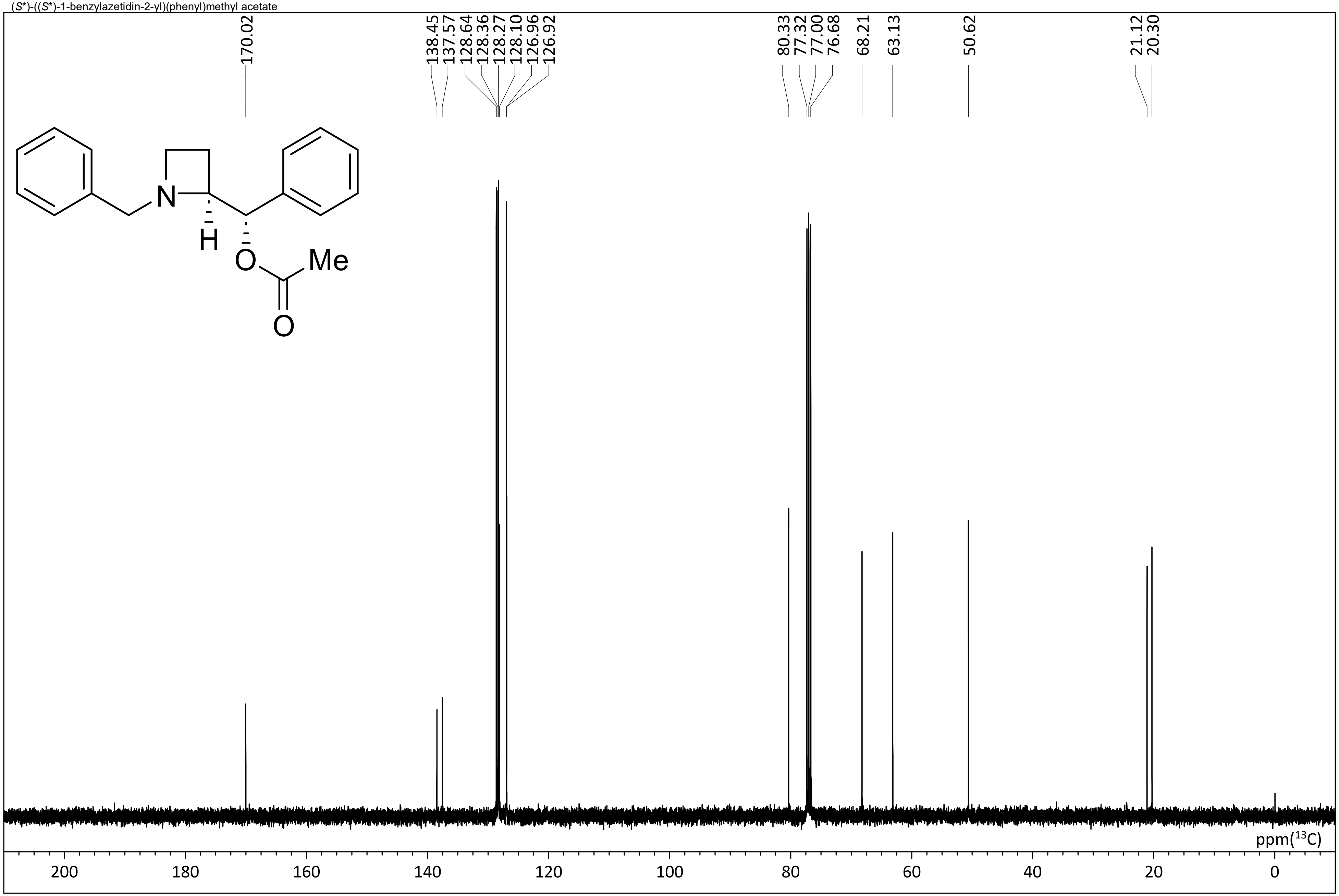

(S*)-((S*)-1-benzylazetidin-2-yl)(4-fluorophenyl)methanol

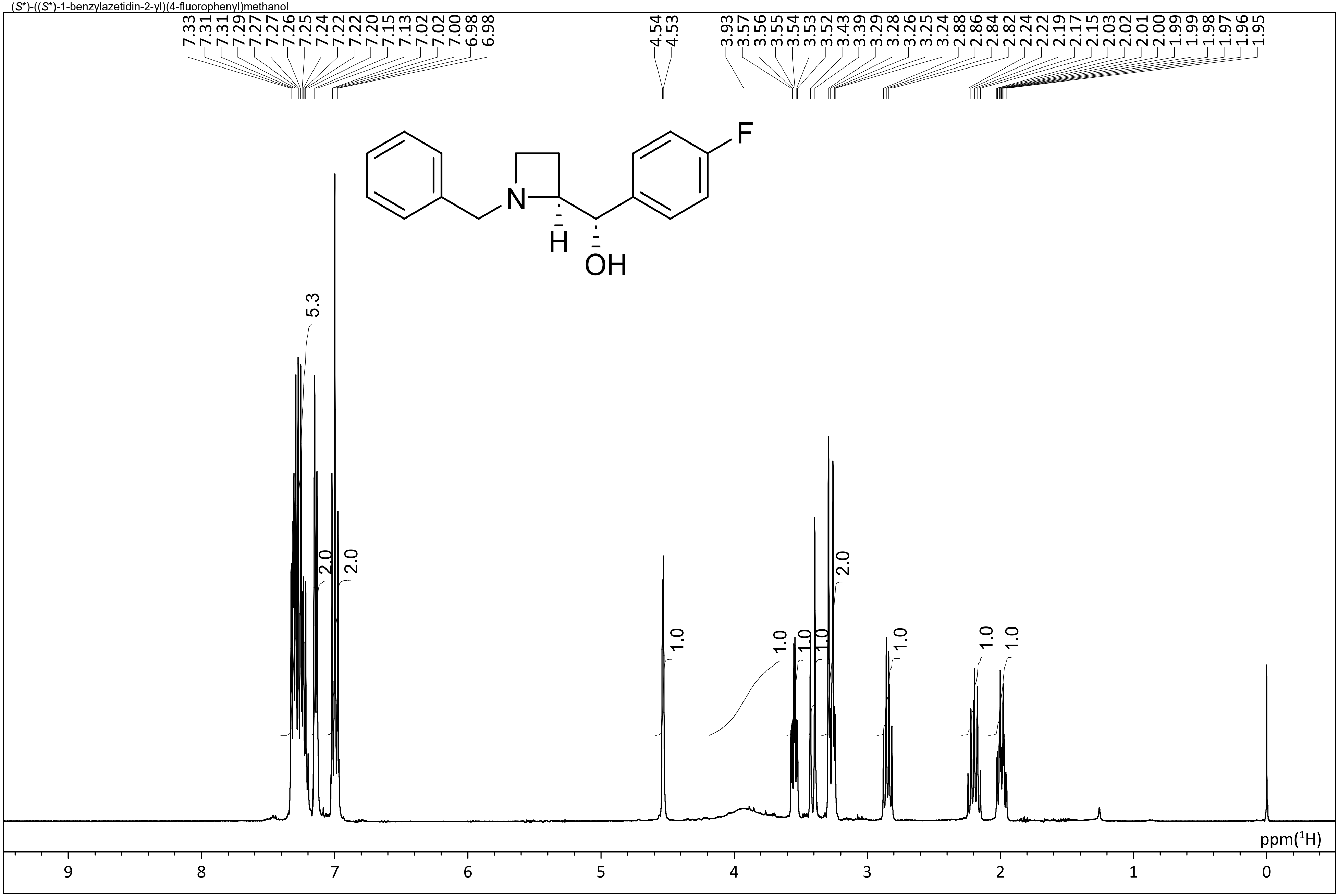

(*S**)-((*S**)-1-benzylazetidin-2-yl)(4-fluorophenyl)methanol

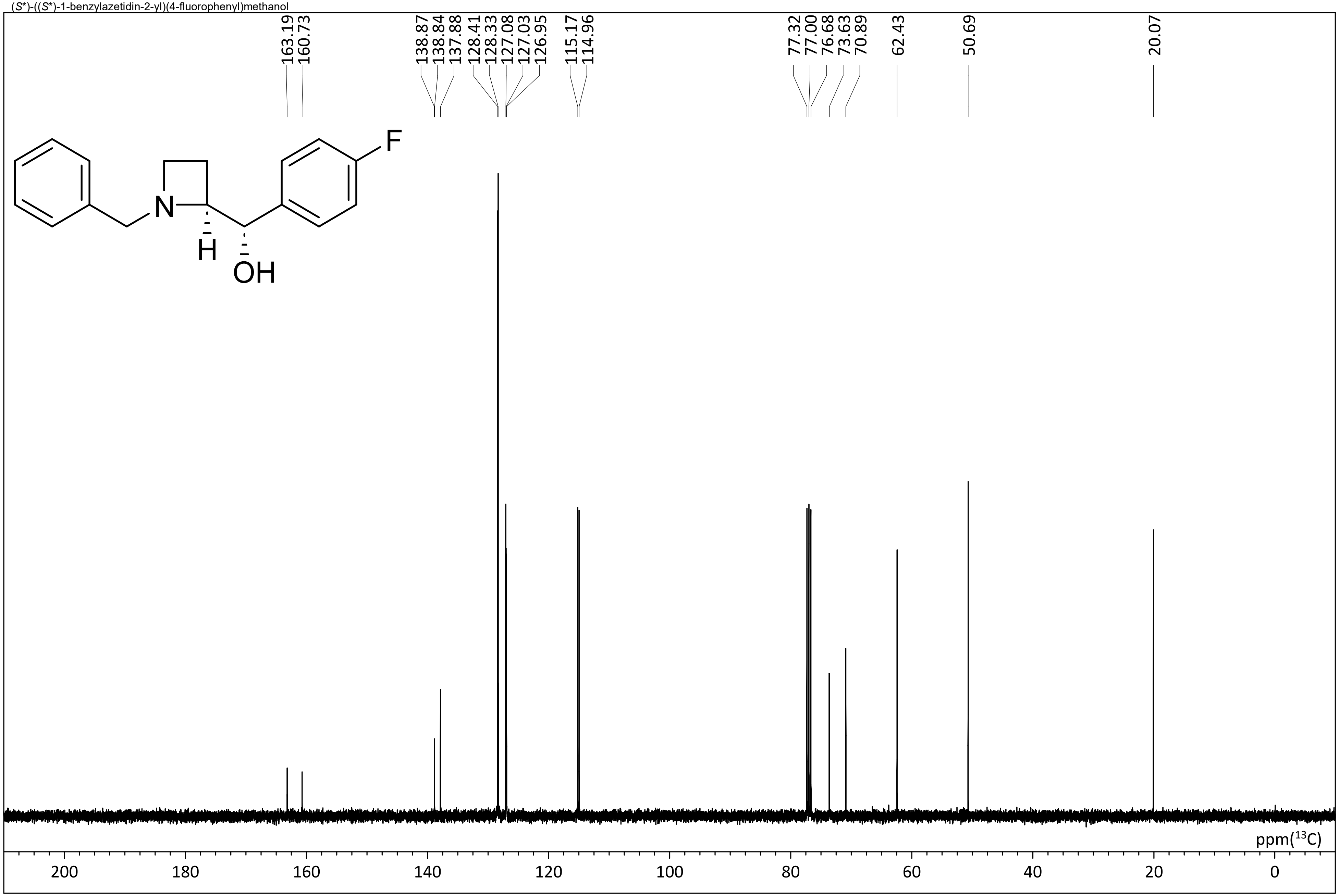

Supplement: Supplementary file 1 [file DataSheet2.pdf]
