# Supplementary material for: Azetidine synthesis by La(OTf)3-catalyzed intramolecular regioselective aminolysis of cis-3,4-epoxy amines
Source: Front Chem. 2023 Sep 19;11:1251299. doi: 10.3389/fchem.2023.1251299 (PMC10546187; doi:10.3389/fchem.2023.1251299)
Supplement: Supplementary file 2 [file DataSheet1.pdf]

Supplementary Material

Azetidine Synthesis by La(OTf)3-catalyzed Intramolecular Regioselective Aminolysis of *Cis*-3,4-epoxy Amines

Yuse Kuriyama, Yusuke Sasano, Yoshiharu Iwabuchi*

*** Correspondence:** Yoshiharu Iwabuchi: y-iwabuchi@tohoku.ac.jp

1 Supplementary Data

1-1. General Information

All reactions were carried out under argon atmosphere with dehydrated solvents under anhydrous conditions unless otherwise noted. Dehydrated THF and CH_2Cl_2 were purchased from Kanto Chemical Co., Inc., and the other solvents were dehydrated and distilled according to standard protocols. Reagents were obtained from commercial suppliers and used without further purification, unless otherwise noted.

Reactions were monitored by thin-layer chromatography (TLC) on 0.25 mm Merck silica gel plates (60F-254). Column chromatography was performed using Silica Gel 60N (Kanto Chemical Co., Inc., spherical, neutral, particle size 63–210 μm) and NH-DM1020 (Fuji Silysia Chemical Ltd., spherical, particle size 100 μm); flash column chromatography was performed using Silica Gel 60N (Kanto Chemical Co., Inc., spherical, neutral, particle size 40–50 μm), unless otherwise noted.

Melting points were measured using a Yazawa BY-2 and Buchi M-565 and were uncorrected. Infrared (IR) spectra were obtained using a JASCO FT-IR-4600 instrument and are reported as wavenumbers. Proton nuclear magnetic resonance (^{1}H-NMR) spectra were recorded using a JEOL JMN-AL400 (400 MHz) and a JEOL ECA-600 (600 MHz) spectrometer. Chemical shift (δ) is reported in parts per million (ppm) downfield relative to tetramethyl silane (TMS; 0.0 ppm) in $CDCl_3$ and benzene (7.16 ppm) in C_6D_6. The coupling constants (*J*) are reported in Hz. Carbon-13 nuclear magnetic resonance (^{13}C-NMR) spectra were recorded on a JEOL JMN-AL400 (100 MHz) spectrometer. Chemical shifts are reported in ppm relative to the center line of the triplet of $^{13}CDCl_3$ (77.0 ppm) and $^{13}C_6D_6$ (128.0 ppm). Low-resolution mass spectra (MS) were recorded on JEOL JMS-DX303, JMS-T100GC, and JEOL JMS-700 instruments. High-resolution mass spectra (HRMS) were recorded on JEOL JMS-T100GC and JEOL JMS-700 mass spectrometers using electron impact (EI) and on a Thermo Scientific Exactive Mass Spectrometer using electrospray ionization (ESI).

1-2. Preparation of *cis*-epoxy amines

General procedure of epoxy alkyl amines synthesis (**1aa**–**1ka**, **1ab** and **1ac**)

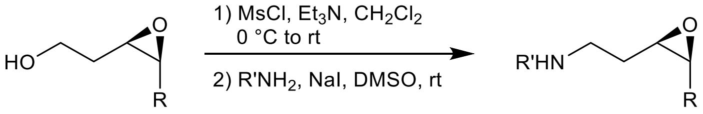

Et_3N (2.5 eq) and MsCl (1.5 eq) were added to a solution of epoxy alcohol (1 eq) in CH_2Cl_2 (0.5 M) at 0 °C, and the mixture was stirred for 10 min at room temperature. Then, saturated aqueous $NaHCO_3$ was added to the mixture at 0 °C, and the mixture was extracted thrice with CH_2Cl_2. The combined organic layers were dried over anhydrous $MgSO_4$, filtered, and concentrated under reduced pressure. The resulting crude product was used immediately in the subsequent reaction without further purification.

To a solution of the crude product in DMSO (0.5 M) were added alkyl amine (3.0 eq) and NaI (10 mol%) at room temperature (ca. 25 °C), and the mixture was stirred for 2 d at ambient temperature. The mixture was diluted with H_2O and extracted with Et_2O. The combined organic layers were washed thrice with brine, dried over anhydrous Na_2SO_4, filtered, and concentrated under reduced pressure. The resulting residue was purified by column chromatography to yield the corresponding epoxy amines.

Synthesis of epoxy alcohol **S2**

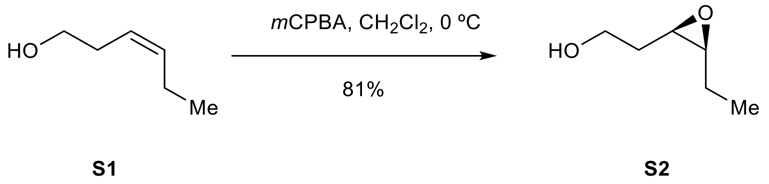

To a solution of *cis*-3-hexen-1-ol **S1** (3.07 g, 30.7 mmol) in CH_2Cl_2 (100 mL) was added *m*CPBA (contains 25-31% H_2O, 8.64 g, 36.0 mmol) at 0 °C, and stirred for 1 h at 0 °C. Then, saturated aqueous $NaHCO_3$ and saturated aqueous $Na_2S_2O_3$ were added at 0 °C, and the resulting solution was extracted with CH_2Cl_2 (25 mL×3). The combined organic layer was dried over $MgSO_4$, filtered, concentrated under reduced pressure. The resulting residue was purified column chromatography (silica gel, hexane/Et_2O = 2→1) to give epoxy alcohol **S2** (2.89g, 24.9 mmol, 81%) as a colorless oil.

S2 : IR (neat) ν 668, 738, 780, 814, 869, 905, 995, 1059, 1147, 1199, 1274, 1392, 1469, 1650, 2879, 2972, 3399 cm^{-1}; ^{1}H NMR (400 MHz, $CDCl_3$) δ 1.06 (3H, t, *J* = 7.6 Hz), 1.48-1.65 (3H, m), 1.65-1.80 (2H, m), 1.88 (1H, dddd, *J* = 4.7, 5.4, 6.8, 14.3 Hz), 2.92 (1H, dt, *J* = 4.4, 6.3 Hz), 3.11 (1H, td, *J* = 4.4, 7.9 Hz), 3.75-3.95 (2H, m); ^{13}C NMR (100 MHz, $CDCl_3$) δ 10.3, 21.0, 30.4, 55.0, 57.9, 60.2; MS (ESI) m/z: $[M+Na]^+$ calcd for $C_6H_{12}O_2Na$ 139.0730; Found 139.0728

N-benzyl-2-((2*R**,3*S**)-3-ethyloxiran-2-yl)ethan-1-amine (**1aa**)

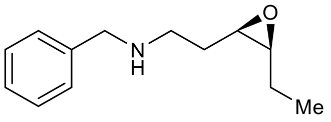

Yield: 84%, column chromatography (silica gel, $CHCl_3$/MeOH = 100→50), pale yellow oil, IR (neat) ν 699, 737, 817, 907, 1120, 1454, 1495, 2820, 2876, 2934, 2970, 3027, 3061, 3313 cm^{-1} ; ^{1}H NMR (400 MHz, $CDCl_3$) δ 1.03 (3H, t, *J* = 7.5 Hz), 1.40-1.63 (3H, m), 1.64-1.73 (1H, m), 1.73-1.86 (1H, m), 2.75-2.93 (3H, m), 3.00 (1H, td, *J* = 4.6, 7.4 Hz), 3.81 (2H, s), 7.10-7.44 (5H, m); ^{13}C NMR (100 MHz, $CDCl_3$) δ 10.5, 21.2, 28.3, 46.7, 54.01, 55.8, 58.0, 126.9, 128.0 (CH×2), 128.4 (CH×2), 140.3; MS (EI) m/z: $[M]^+$ calcd for $C_{13}H_{19}NO$ 205.1467; Found 205.1470

2-((2*R**,3*S**)-3-ethyloxiran-2-yl)-*N*-(4-methoxybenzyl)ethan-1-amine (**1ba**)

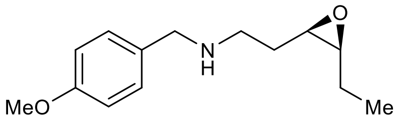

Yield: 75%, column chromatography (silica gel, hexane/EtOAc = 1 → $CHCl_3$/MeOH = 30), yellow oil, IR (neat) ν 816, 1036, 1106, 1176, 1247, 1301, 1464, 1512, 1611, 2834, 2876, 2935, 2969, 3317 cm^{-1}; ^1H NMR (400 MHz, $CDCl_3$) δ 1.03 (3H, t, *J* = 7.5 Hz), 1.39 (1H, br-s), 1.44–1.60 (2H, m), 1.61–1.72 (1H, m), 1.72–1.84 (1H, m), 2.74–2.85 (2H, m), 2.87 (1H, dt, *J* = 4.4, 6.3 Hz), 2.99 (1H, ddd, *J* = 4.4, 4.8, 9.0 Hz), 3.75 (2H, s), 3.79 (3H, s), 6.86 (2H, d, *J* = 8.5 Hz), 7.24 (2H, d, *J* = 8.5 Hz); ^{13}C NMR (100 MHz, $CDCl_3$) δ 10.5, 21.2, 28.3, 46.7, 53.4, 55.2, 55.8, 58.0, 113.7 (CH×2), 129.2 (CH×2), 132.4, 158.6; MS (EI) m/z: $[M]^+$ calcd for $C_{14}H_{21}NO_2$ 235.1572; Found 235.1580

2-((2*R**,3*S**)-3-ethyloxiran-2-yl)-*N*-(4-(trifluoromethyl)benzyl)ethan-1-amine (**1ca**)

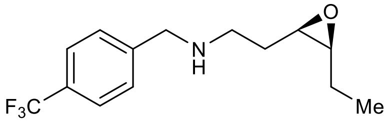

Yield: 55%, column chromatography (silica gel, hexane/EtOAc = 1 → $CHCl_3$/MeOH = 100), yellow oil, IR (neat) ν 821, 907, 1018, 1066, 1123, 1163, 1327, 1417, 1460, 1619, 2826, 2879, 2938, 2972, 3327 cm^{-1}; ^1H NMR (400 MHz, $CDCl_3$) δ 1.04 (3H, t, *J* = 7.6 Hz), 1.38–1.62 (2H, m), 1.48 (1H, br–s), 1.62–1.72 (1H, m), 1.74–1.86 (1H, m), 2.74–2.85 (2H, m), 2.89 (1H, dt, *J* = 4.4, 6.3 Hz), 3.01 (1H, td, *J* = 4.4, 7.7 Hz), 3.88 (3H, s), 7.45 (2H, d, *J* = 8.1 Hz), 7.58 (2H, d, *J* = 8.1 Hz); ^{13}C NMR (100 MHz, $CDCl_3$) δ 10.5, 21.2, 28.3, 46.9, 53.5, 55.8, 57.9, 124.2 (q, *J* = 272 Hz), 125.3 (q, *J* = 3.8 Hz), 128.2 (CH×2), 129.2 (q, *J* = 32.2 Hz), 144.5 (q, *J* = 1.2 Hz); MS (EI) m/z: $[M]^+$ calcd for $C_{14}H_{18}NOF_3$ 273.1340; Found 273.1355

N-(2-((2*R**,3*S**)-3-ethyloxiran-2-yl)ethyl)butan-1-amine (**1da**)

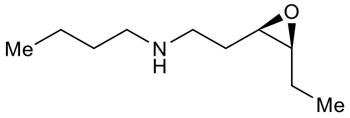

Yield: 79%, column chromatography (silica gel, $CHCl_3$/MeOH = 50→10), pale yellow oil, IR (neat) ν 737, 815, 908, 1129, 1271, 1307, 1378, 1466, 2815, 2874, 2930, 2961, 3311 cm^{-1}; ^1H NMR (400 MHz, $CDCl_3$) δ 0.92 (3H, t, *J* = 7.3 Hz), 1.04 (3H, t, *J* = 7.5 Hz), 1.12 (1H, br-s), 1.28–1.42 (2H, m), 1.43–1.71 (5H, m), 1.77 (1H, dddd, *J* = 4.6, 6.7, 7.8, 14.3 Hz), 2.63 (2H, dd, *J* = 7.1, 7.4 Hz), 2.73–2.85 (2H, m), 2.89 (1H, dt, *J* = 4.3, 6.3 Hz), 2.99 (1H, ddd, *J* = 4.3, 4.9, 7.5 Hz); ^{13}C NMR (100 MHz, $CDCl_3$) δ 10.5, 13.4, 20.5, 21.2, 28.4, 32.3, 47.4, 49.8, 55.8, 58.0; MS (EI) m/z: $[M]^+$ calcd for $C_{10}H_{21}NO$ 171.1623; Found 171.1621

N-(2-((2*R**,3*S**)-3-ethyloxiran-2-yl)ethyl)-2-methylpropan-2-amine (**1ea**)

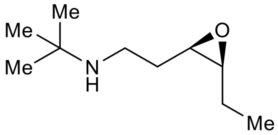

Yield: 51%, column chromatography (silica gel, $CHCl_3$/MeOH = 8), pale yellow oil, IR (neat) ν 704, 785, 817, 1114, 1146, 1230, 1361, 1467, 2876, 2968, 3313 cm^{-1}; ^1H NMR (400 MHz, $CDCl_3$) δ 1.04 (3H, t, *J* = 7.5 Hz), 1.12 (9H, s), 1.45–1.68 (3H, m), 1.70–1.84 (1H, m), 2.66–2.84 (2H, m), 2.88 (1H, dt, *J* = 4.4, 6.2 Hz), 2.99 (1H, td, *J* = 4.4, 7.7 Hz); ^{13}C NMR (100 MHz, $CDCl_3$) δ 10.5, 21.2, 29.0 (CH_3×3), 29.3, 40.1, 50.3, 55.9, 57.9; MS (EI) m/z: $[M]^+$ calcd for $C_{10}H_{21}NO$ 171.1623; Found 171.1629

N-(2-((2*R**,3*S**)-3-ethyloxiran-2-yl)ethyl)prop-2-en-1-amine (**1fa**)

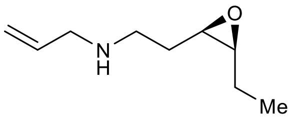

Yield: 65%, column chromatography (silica gel, $CHCl_3$/MeOH = 50→20), yellow oil, IR (neat) ν 816, 914, 994, 1120, 1459, 1641, 2816, 2877, 2919, 2936, 2971, 3077, 3313 cm^{-1}; ^{1}H NMR (400 MHz, $CDCl_3$) δ 1.05 (3H, t, *J* = 7.5 Hz), 1.40 (1H, br–s), 1.47–1.62 (2H, m), 1.63–1.72 (1H, m), 1.73–1.85 (1H, m), 2.73–2.86 (2H, m), 2.89 (1H, ddd, *J* = 4.3, 6.1, 6.6 Hz), 3.00 (1H, ddd, *J* = 4.3, 4.9, 7.5 Hz), 3.28 (2H, ddd, *J* = 1.3, 1.6, 6.0 Hz), 5.10 (1H, td, *J* = 1.3, 10.3 Hz), 5.18 (1H, td, *J* = 1.6, 17.2 Hz), 5.91 (1H, tdd, *J* = 6.0, 10.3, 17.2 Hz); ^{13}C NMR (100 MHz, $CDCl_3$) δ 10.5, 21.2, 28.4, 46.8, 52.5, 55.8, 58.0, 115.9, 136.8; MS (EI) m/z: $[M]^+$ calcd for $C_9H_{17}NO$ 155.1310 ; Found 155.1326

tert-butyl (5-((2-((2*R**,3*S**)-3-ethyloxiran-2-yl)ethyl)amino)pentyl)carbamate (**1ga**)

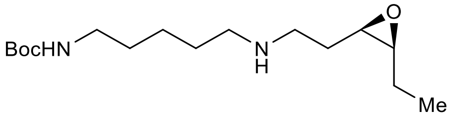

Yield: 78%, column chromatography (silica gel, $CHCl_3$/MeOH = 30→10), pale yellow oil, IR (neat) ν 738, 780, 816, 870, 908, 955, 1010, 1040, 1173, 1251, 1365, 1390, 1455, 1530, 1694, 1713, 2819, 2932, 2972, 3346 cm^{-1}; ^{1}H NMR (400 MHz, $CDCl_3$) δ 1.04 (3H, t, *J* = 7.6 Hz), 1.22–1.41 (3H, m), 1.44 (9H, s), 1.46–1.71 (7H, m), 1.72–1.88 (1H, m), 2.63 (2H, t, *J* = 7.1 Hz), 2.70–2.84 (2H, m), 2.89 (1H, dt, *J* = 4.3, 6.3 Hz), 2.99 (1H, td, *J* = 4.5, 7.6 Hz), 3.02–3.26 (2H, br-q, *J* = 6.3 Hz), 4.32–4.84 (1H, br-s); ^{13}C NMR (100 MHz, $CDCl_3$) δ 10.5, 21.1, 24.5, 28.2, 28.3 (CH_3×3), 29.6, 29.9, 40.4, 47.3, 49.8, 55.7, 57.9, 78.9, 155.9; MS (EI) m/z: $[M]^+$ calcd for $C_{16}H_{32}N_2O_3$ 300.2413; Found 300.2406

N-(2-((2*R**,3*S**)-3-ethyloxiran-2-yl)ethyl)-5-((4-methoxybenzyl)oxy)pentan-1-amine (**1ha**)

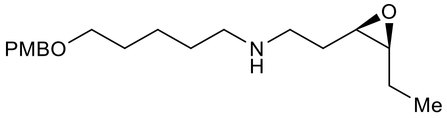

Yield: 87%, column chromatography (silica gel, $CHCl_3$/MeOH = 50→15), pale yellow oil, IR (neat) ν 755, 820, 907, 1036, 1098, 1173, 1248, 1302, 1362, 1464, 1513, 1586, 1613, 2856, 2934, 3317 cm^{-1}; ^{1}H NMR (400 MHz, $CDCl_3$) δ 1.04 (3H, t, *J* = 7.5 Hz), 1.08–1.29 (1H, br-s), 1.32–1.44 (2H, m), 1.45–1.70 (7H, m), 1.76 (1H, dddd, *J* = 5.0, 6.6, 7.7, 12.8 Hz), 2.62 (2H, t, *J* = 7.2 Hz), 2.79, (2H, dddd, *J* = 6.6, 7.7, 11.6, 18.0 Hz), 2.88 (1H, dt, *J* = 4.3, 6.3), 2.98 (1H, td, *J* = 4.5, 7.5), 3.44 (2H, t, *J* = 6.5 Hz), 3.80 (3H, s), 4.42 (2H, s), 6.88 (2H, d, *J* = 8.6 Hz), 7.25 (2H, d, *J* = 8.6 Hz); ^{13}C NMR (100 MHz, $CDCl_3$) δ 10.5, 21.1, 23.9, 28.4, 29.6, 29.9, 47.4, 50.0, 55.2, 55.8, 57.9, 70.0, 72.5, 113.7 (CH×2), 129.2 (CH×2), 130.7, 159.0; MS (EI) m/z: $[M–H]^+$ calcd for $C_{19}H_{30}NO_3$ 320.2226; Found 320.2227

3-((*tert*-butyldimethylsilyl)oxy)-*N*-(2-((2*R**,3*S**)-3-ethyloxiran-2-yl)ethyl)propan-1-amine (**1ia**)

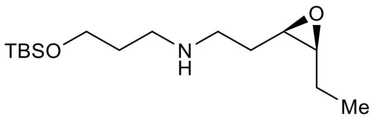

Yield: 78%, column chromatography (silica gel, $CHCl_3$/MeOH = 50→30), pale yellow oil, IR (neat) ν 662, 681, 724, 776, 837, 909, 939, 967, 1006, 1098, 1188, 1254, 1361, 1389, 1471, 2857, 2930, 2955, 3335 cm^{-1}; ^{1}H NMR (400 MHz, $CDCl_3$) δ 0.05 (6H, s), 0.89 (9H, s), 1.04 (3H, t, *J* = 7.6 Hz), 1.18–

1.39 (1H, br-s), 1.45–1.82 (6H, m), 2.72 (2H, t, *J* = 6.9 Hz), 2.75–2.85 (2H, m), 2.89 (1H, dt, *J* = 4.3, 6.3 Hz), 2.99 (1H, td, J = 4.6, 7.3 Hz), 3.69 (2H, t, *J* = 6.1 Hz); ^{13}C NMR (100 MHz, $CDCl_3$) δ -5.42 (CH_3×2), 10.5, 18.2, 21.1, 25.9 (CH_3×3), 28.4, 32.9, 47.3, 47.4, 55.7, 58.0, 61.7; MS (EI) m/z: $[M]^+$ calcd for $C_{15}H_{33}NO_2Si$ 287.2281; Found 287.2277

3-((2-((2*R**,3*S**)-3-ethyloxiran-2-yl)ethyl)amino)propanenitrile (**1ja**)

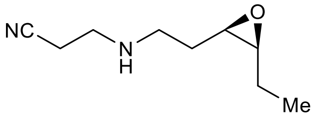

Yield: 18%, column chromatography (silica gel, $CHCl_3$/MeOH = 100→50), pale yellow oil, IR (neat) ν 740, 774, 815, 907, 1131, 1358, 1389, 1422, 1469, 2247, 2844, 2877, 2935, 2970, 3315 cm^{-1}; ^{1}H NMR (400 MHz, $CDCl_3$) δ 1.05 (3H, t, *J* = 7.5 Hz), 1.39 (1H, br–s), 1.45–1.72 (3H, m), 1.73–1.86 (1H, m), 2.53 (2H, t, *J* = 6.6 Hz), 2.78–2.93 (3H, m), 2.96 (2H, t, *J* = 6.6 Hz), 3.01 (1H, td, *J* = 4.4, 7.8 Hz); ^{13}C NMR (100 MHz, $CDCl_3$) δ 10.5, 18.7, 21.2, 28.2, 45.1, 46.7, 55.6, 57.9, 118.6; MS (EI) m/z: $[M]^+$ calcd for $C_9H_{16}N_2O$ 168.1263; Found 168.1258

N-(2-((2*R**,3*S**)-3-ethyloxiran-2-yl)ethyl)-3-(methylthio)propan-1-amine (**1ka**)

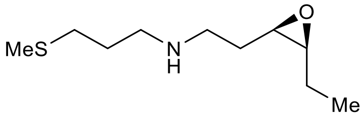

Yield: 75%, column chromatography (silica gel, $CHCl_3$/MeOH = 100→20), pale yellow oil, IR (neat) ν 815, 907, 1127, 1467, 2820, 2876, 2916, 2969, 3307 cm^{-1}; ^{1}H NMR (400 MHz, $CDCl_3$) δ 1.04 (3H, t, *J* = 7.5 Hz), 1.36 (1H, br-s), 1.46–1.71 (3H, m), 1.72–1.86 (3H, m), 2.11 (3H, s), 2.56 (3H, t, *J* = 7.2 Hz), 2.73 (2H, t, *J* = 7.0 Hz), 2.73–2.86 (2H, m), 2.89 (1H, dt, *J* = 4.4, 6.3 Hz), 2.99 (1H, td, *J* = 4.4, 7.6 Hz); ^{13}C NMR (100 MHz, $CDCl_3$) δ 10.5, 15.6, 21.2, 28.4, 29.4, 32.1, 47.4, 48.9, 55.8, 58.0; MS (EI) m/z: $[M]^+$ calcd for $C_{10}H_{21}NOS$ 203.1344; Found 203.1339

General procedure of epoxy anilines synthesis (1la–1na) [1]

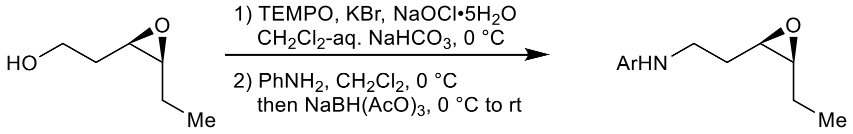

S2 **1la–1na**

A pre-mixed solution of $NaOCl·5H_2O$ (1.5 eq) in saturated aqueous $NaHCO_3$ was added dropwise to a cooled and well stirred mixture of epoxy alcohol (1.0 eq) and TEMPO (1 mol%) in CH_2Cl_2 (0.2 M) and saturated aqueous $NaHCO_3$ containing KBr (10 mol%), and the resulting mixture was stirred for 10 min at 0 °C. Then, saturated aqueous $Na_2S_2O_3$ was added at 0 °C, and the mixture was extracted with CH_2Cl_2. The combined organic layers were washed with brine, dried over $MgSO_4$, filtered, and concentrated under reduced pressure. The resulting crude product was used immediately in the subsequent reaction without purification.

$ArNH_2$ (1.0 eq) was added to a solution of the above crude product in CH_2Cl_2. After the mixture was stirred for 10 min at 0 °C, $NaBH(OAc)_3$ (1.2 eq) was added at 0 °C and stirred at room temperature. Then saturated aqueous $NaHCO_3$ was added, and the resulting mixture extracted thrice with CH_2Cl_2. The combined organic layers were dried over anhydrous Na_2SO_4, filtered, and concentrated under reduced pressure. The resulting residue was purified using column chromatography to yield the corresponding epoxy anilines.

N-(2-((2*R**,3*S**)-3-ethyloxiran-2-yl)ethyl)aniline (**1la**)

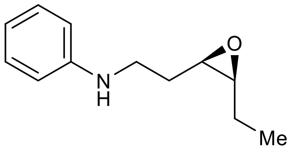

Yield: 25%, column chromatography (silica gel, hexane/EtOAc = 15), pale yellow oil, IR (neat) ν 693, 750, 815, 875, 907, 991, 1027, 1073, 1121, 1152, 1179, 1210, 1265, 1320, 1390, 1434, 1475, 1510, 1604, 1768, 1825, 1920, 2583, 2875, 2970, 3022, 3052, 3390 cm^{-1}; ^{1}H NMR (400 MHz, $CDCl_3$) δ 1.06 (3H, t, *J* = 7.6 Hz), 1.46–1.66 (2H, m), 1.73 (1H, tdd, *J* = 6.9, 8.0, 14.0 Hz), 1.97 (1H, dddd, *J* = 4.3, 6.6, 7.5, 14.0 Hz), 2.92 (1H, dt, *J* = 4.3, 6.3 Hz), 3.05 (1H, td, J = 4.3, 8.0 Hz), 3.35 (2H, t, *J* = 6.8 Hz), 3.86 (1H, br-s), 6.63 (2H, d, *J* = 8.5 Hz), 6.71 (1H, t, *J* = 7.4 Hz), 7.18 (2H, dd, *J* = 7.4, 8.5 Hz); ^{13}C NMR (100 MHz, $CDCl_3$) δ 10.5, 21.2, 27.7, 41.8, 55.7, 57.9, 112.8 (CH×2), 117.4, 129.3 (CH×2), 148.1; MS (EI) m/z: $[M]^+$ calcd for $C_{12}H_{17}NO$ 191.1310; Found 191.1304

N-(2-((2*R**,3*S**)-3-ethyloxiran-2-yl)ethyl)-4-mehtoxyaniline (**1ma**)

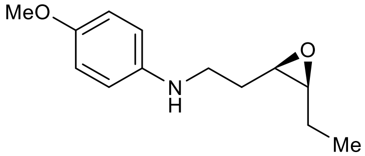

Yield: 33%, column chromatography (silica gel, hexane/EtOAc = 10), yellow oil, IR (neat) ν 754, 820, 907, 1038, 1123, 1149, 1179, 1235, 1295, 1390, 1409, 1466, 1515, 1619, 1737, 1847, 2062, 2832, 2876, 2970, 3381 cm^{-1}; ^{1}H NMR (400 MHz, $CDCl_3$) δ 1.04 (3H, t, *J* = 7.5 Hz), 1.42–1.65 (2H, m), 1.65–1.78 (1H, m), 1.92 (1H, dtd, *J* = 4.3, 6.8, 14.0 Hz), 2.90 (1H, ddd, *J* = 4.3, 6.2, 6.5 Hz), 3.04, (1H, td, *J* = 4.3, 8.0 Hz), 3.28 (2H, t, *J* = 6.8 Hz), 3.50–3.66 (1H, br-s), 3.74 (3H, s), 6.59 (2H, d, *J* = 9.0 Hz), 6.78 (2H, d, *J* = 9.0 Hz); ^{13}C NMR (100 MHz, $CDCl_3$) δ 10.4, 21.2, 27.7, 42.6, 55.6, 55.7, 57.8, 114.0 (CH×2), 114.9 (CH×2), 142.3, 152.1; MS (EI) m/z: $[M]^+$ calcd for $C_{13}H_{19}NO_2$ 221.1416; Found 221.1406

N-(2-((2*R**,3*S**)-3-ethyloxiran-2-yl)ethyl)-4-nitroaniline (**1na**)

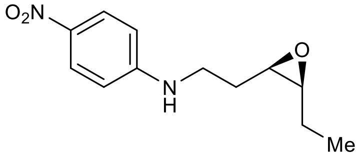

Yield: 17% from 4-nitroaniline (0.25 eq), column chromatography (silica gel, hexane/EtOAc = 10 then hexane/CH_2Cl_2 = 1/2 → CH_2Cl_2 only), yellow oil, IR (neat) ν 695, 753, 833, 1111, 1185, 1307, 1472, 1504, 1534, 1603, 2971, 3369 cm^{-1}; ^{1}H NMR (400 MHz, $CDCl_3$) δ 1.06 (3H, t, *J* = 7.5 Hz), 1.46–1.81 (3H, m), 2.06 (1H, dtd, *J* = 3.6, 6.2, 14.4 Hz), 2.94 (1H, ddd, *J* = 4.3, 6.0, 6.7 Hz), 3.06 (1H, ddd, *J* = 3.7, 4.2, 8.7 Hz), 3.36–3.54 (2H, m), 4.86–5.11 (1H, br-s), 6.55 (2H, d, *J* = 9.2 Hz), 8.08 (2H, d, *J* = 9.2 Hz); ^{13}C NMR (100 MHz, $CDCl_3$) δ 10.4, 21.2, 27.0, 41.5, 55.4, 57.8, 111.0 (CH×2), 126.4 (CH×2), 138.0, 153.2; MS (EI) m/z: $[M]^+$ calcd for $C_{12}H_{16}N_2O_3$ 236.1161; Found 236.1161

Synthesis of epoxy alcohol **S6a**, **S6b**

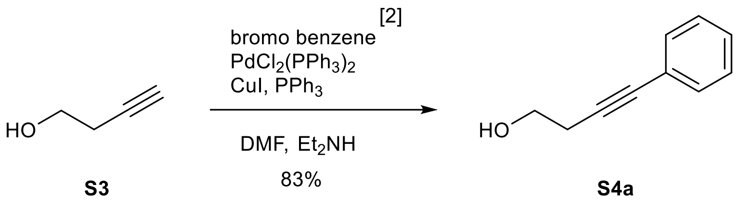

Bromobenzene (0.70 mL, 6.68 mmol), bis(triphenylphosphine) palladium (II) chloride (241 mg, 0.343 mmol), triphenylphosphine (123 mg, 0.469 mmol), and copper (I) iodide (14.1 mg, 74.0 μmol) were added to a dry round-bottomed flask, which was then sparged with argon and charged with diethylamine (1.3 mL) and DMF (13 mL). 3-Butyn-1-ol (**S3**) (0.5 mL, 6.61 mmol) was added via syringe. The stirred reaction mixture was heated at 80 °C for 12 h. After it was cooled to room temperature, the reaction mixture was diluted with diethyl ether (15 mL), and filtered. The filtrate was poured into water and the aqueous layer was extracted with diethyl ether (3×20 mL). The combined organic layer was washed with brine, dried over anhydrous $MgSO_4$ and filtered, and concentrated under reduced pressure. The resulting residue was purified by column chromatography (silica gel, hexane/Et_2O = 3→1.5) to give a homopropargyl alcohol **S4a** (802 mg, 83%) as a dark brown liquid.

S4a : IR (neat) ν 691, 756, 846, 915, 1044, 1179, 1334, 1442, 1490, 1572, 1600, 2234, 2886, 3056, 3349 cm^{-1}; ^1H NMR (400 MHz, $CDCl_3$) δ 2.06 (1H, br-s), 2.69 (2H, t, *J* = 6.3 Hz), 3.81 (2H, dt, *J* = 4.0, 6.3 Hz), 7.29 (3H, m), 7.41 (2H, m); ^{13}C NMR (100 MHz, $CDCl_3$) δ 23.8, 61.1, 82.4, 86.3, 123.3, 127.9, 128.2 (CH×2), 131.6 (CH×2); MS (EI) m/z: $[M]^+$ calcd for $C_{10}H_{10}O$ 146.0732 ; Found 146.0717

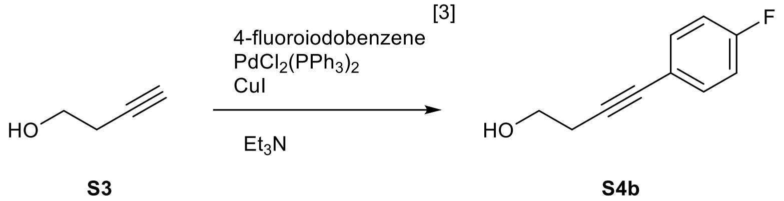

A mixture of 4-fluoroiodobenzene (1.50 g, 6.76 mmol), $PdCl_2(PPh_3)_2$ (235 mg, 0.335 mmol), and CuI (102 mg, 0.538 mmol) under an argon atmosphere was dissolved in triethylamine (100 mL). 3-Butyn-1-ol (**S3**) (0.61 mL, 8.10 mmol) was then added dropwise. After being stirred at room temperature for 5 h, the solution was quenched with a saturated solution of NH_4Cl, and the resulting mixture was filtered through a celite pad. The filtrate was extracted with Et_2O (3×15 mL). The organic layers were then washed with brine, dried with Na_2SO_4, and evaporated in vacuo to give the crude product which was purified by column chromatography (silica gel, hexane/Et_2O = 1) to give a homopropargyl alcohol **S4b** (1.04 g, 94%) as a pale-yellow solid.

S4b: m.p. 33.6–35.1 °C; IR (neat) ν 811, 836, 1014, 1045, 1092, 1156, 1231, 1421, 1471, 1507, 1602, 1893, 2239, 2888, 2945, 3349 cm^{-1}; ^1H NMR (400 MHz, $CDCl_3$) δ 2.05–2.27 (1H, br-s), 2.66 (2H, t, *J* = 6.3 Hz), 3.80 (2H, q, *J* = 6.1 Hz), 6.97 (2H, t, *J* = 8.8 Hz), 7.38 (2H, dd, *J* = 5.4, 8.8 Hz); ^{13}C NMR (100 MHz, $CDCl_3$) δ 23.7, 61.1, 81.3, 86.0 (d, *J* = 1.4 Hz), 115.4 (CH×2, d, *J* = 22.1 Hz), 119.4 (d, *J* = 3.7 Hz), 133.4 (CH×2, d, *J* = 8.4 Hz), 162.2 (d, *J* = 248.9 Hz); MS (EI) m/z: $[M]^+$ calcd for $C_{10}H_9FO$ 164.0637; Found 164.0645

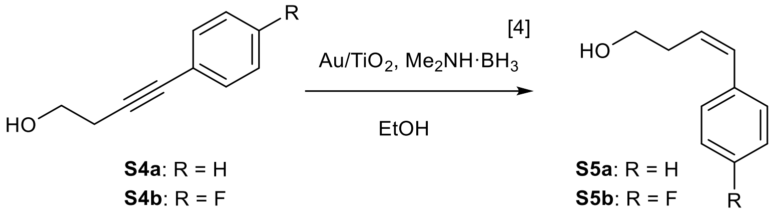

To a solution of **S4** (1.0 eq) in EtOH (0.4 M) was added gold nano particles (1% on TiO_2, 50 w/w%) and $Me_2NH·BH_3$ (10 eq) at room temperature. Upon completion of the reaction, the reaction mixture was filtered through a Celite pad, and the filtrate was concentrated. Then, the residue was diluted with H_2O and extracted thrice with Et_2O. The combined organic layers were washed with water and brine, dried over anhydrous $MgSO_4$, filtered, and concentrated under reduced pressure. The residue was purified by column chromatography to yield a *cis*-styrene **S5**.

S5a: yield 63%, column chromatography (silica gel, hexane/Et_2O = 5), colorless oil; IR (neat) ν 699, 768, 795, 1051, 1446, 1494, 2881, 3022, 3056, 3336 cm^{-1}; ^1H NMR (400 MHz, $CDCl_3$) δ 1.49 (1H, br-d, *J* = 8.7 Hz), 2.62 (2H, td, *J* = 6.3, 7.3 Hz), 3.74 (2H, t, *J* = 6.3 Hz), 5.69 (1H, td, *J* = 7.3, 11.7 Hz), 6.59 (1H, d, *J* = 11.7 Hz), 7.20–7.39 (5H, m); ^{13}C NMR (100 MHz, $CDCl_3$) δ 32.0, 62.5, 126.8, 128.17 (CH×2), 128.22, 128.7 (CH×2), 131.6, 137.2; MS (EI) m/z: $[M]^+$ calcd for $C_{10}H_{12}O$ 148.0888 ; Found 148.0870

S5b: yield 75%, column chromatography (silica gel, hexane/EtOAc = 15), colorless oil; IR (neat) ν 737, 843, 1050, 1096, 1158, 1224, 1398, 1508, 1603, 1893, 2882, 3014, 3349 cm^{-1}; ^1H NMR (400 MHz, $CDCl_3$) δ 1.71–1.88 (1H, br-s), 2.56 (2H, dtd, J = 1.8, 6.5, 7.3 Hz), 3.72 (2H, t, *J* = 6.5 Hz), 5.66 (1H, td, *J* = 7.3, 11.7 Hz), 6.52 (1H, d, *J* = 11.7 Hz), 7.01 (2H, t, *J* = 8.8 Hz), 7.25 (2H, dd, *J* = 5.6, 8.6 Hz); ^{13}C NMR (100 MHz, $CDCl_3$) δ 31.8, 62.3, 115.0 (CH×2, d, *J* = 21.3 Hz), 128.2 (d, *J* = 1.4 Hz), 130.2 (CH×2, d, *J* = 8.0 Hz), 130.4, 133.2 (d, *J* =3.3 Hz), 161.3 (d, *J* = 246.2 Hz); MS (EI) m/z: $[M]^+$ calcd for $C_{10}H_{11}FO$ 166.0794; Found 166.0794

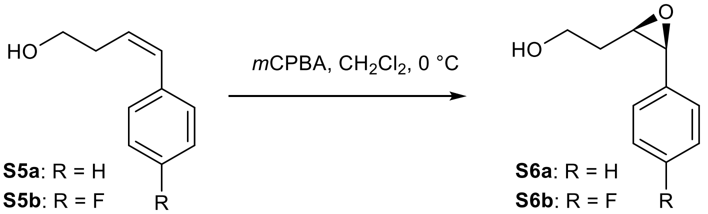

To a solution of **S5** (1.0 eq) in CH_2Cl_2 was added *m*CPBA (contains 27-31% H_2O, 1.5 eq) at 0 °C, and stirred at 0 °C. Then, saturated aqueous $NaHCO_3$ and saturated aqueous $Na_2S_2O_3$ were added at 0 °C, and the resulting solution was extracted fifth times with CH_2Cl_2. The combined organic layer was dried over $MgSO_4$, filtered, concentrated under reduced pressure. The resulting residue was purified by column chromatography to yield epoxy alcohol **S6**.

S6a: yield 84%, column chromatography (silica gel, hexane/Et_2O = 3), yellow oil; IR (neat) ν 701, 743, 884, 913, 1050, 1103, 1132, 1200, 1256, 1314, 1374, 1453, 1496, 1605, 2962, 3031, 3062, 3399 cm^{-1}; ^1H NMR (400 MHz, $CDCl_3$) δ 1.52–1.66 (2H, m), 3.40 (1H, ddd, *J* = 4.4, 5.4, 6.8 Hz), 3.69–3.84 (2H, m), 4.13 (1H, d, *J* = 4.2 Hz), 7.27–7.42 (5H, m); ^{13}C NMR (100 MHz, $CDCl_3$) δ 29.9, 57.0, 57.3, 60.4, 126.5 (CH×2), 127.7, 128.2 (CH×2), 135.3; MS (EI) m/z: $[M]^+$ calcd for $C_{10}H_{12}O_2$ 164.0837; Found 164.0835

S6b: yield 98%, column chromatography (silica gel, hexane/EtOAc = 2), colorless oil; IR (neat) ν 704, 759, 815, 841, 887, 1013, 1050, 1095, 1156, 1222, 1295, 1372, 1437, 1512, 1607, 1901, 2885, 2963, 3399 cm^{-1}; ^{1}H NMR (400 MHz, $CDCl_3$) δ 1.41–1.64 (2H, m), 1.64–1.80 (1H, br-s), 3.38 (1H, ddd, *J* = 1.5, 4.1, 5.3 Hz), 3.68–3.83 (2H, m), 4.10 (1H, d, *J* = 4.1 Hz), 7.04 (2H, t, *J* = 8.7 Hz), 7.28 (2H, dd, *J* = 5.3, 8.7 Hz); ^{13}C NMR (100 MHz, $CDCl_3$) δ 29.8, 56.5, 57.2, 60.2, 115.1 (CH×2, d, *J* = 21.5 Hz), 128.1 (CH×2, d, *J* = 8.0 Hz), 131.0 (d, *J* = 3.0 Hz), 162.3 (d, *J* = 246.0 Hz); MS (EI) m/z: $[M]^+$ calcd for $C_{10}H_{11}FO_2$ 182.0743; Found 182.0748

The epoxy amine substrate with an electron-donating, p-methoxy group on the phenyl group could not be examined because the corresponding epoxy alcohol intermediate ***S6c*** *couldn't be prepared likely due to the instability of the methoxy styrene oxide moiety via electron donation of the methoxy group.*

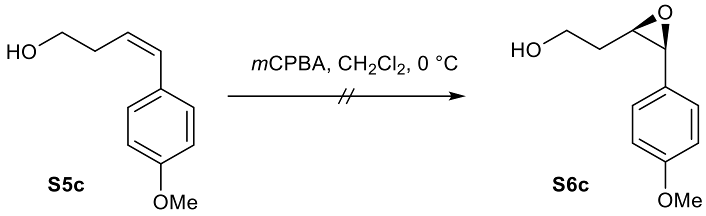

N-benzyl-2-((2*R**,3*S**)-3-phenyloxiran-2-yl)ethan-1-amine (**1ab**)

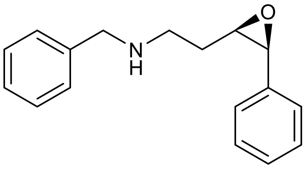

Yield: 79%, column chromatography (silica gel, hexane/EtOAc = 3→1), pale yellow oil, IR (neat) ν 699, 742, 1027, 1119, 1372, 1453, 1496, 2818, 2921, 3028, 3061 cm^{-1}; ^{1}H NMR (400 MHz, $CDCl_3$) δ 1.36 (1H, br-s), 1.43–1.64 (2H, m), 2.64–2.80 (2H, m), 3.26–3.34 (1H, m), 3.67 (1H, d, *J* = 13.3 Hz), 3.72 (1H, d, *J* = 13.3 Hz), 4.08 (1H, d, *J* = 4.2 Hz), 7.19–7.37 (10H, m); ^{13}C NMR (100 MHz, $CDCl_3$) δ 27.3, 46.1, 53.7, 57.1, 58.1, 126.4 (CH×2), 126.8, 127.5, 127.95 (CH×2), 128.02 (CH×2), 128.3 (CH×2), 135.5, 140.2; MS (EI) m/z: $[M]^+$ calcd for $C_{17}H_{19}NO$ 253.1467; Found 253.1486

N-benzyl-2-((2*R**,3*S**)-3-(4-fluorophenyl)oxiran-2-yl)ethan-1-amine (**1ac**)

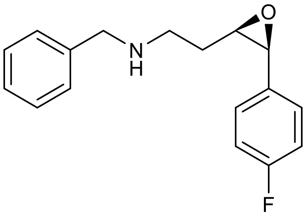

Yield: 88%, column chromatography (silica gel, $CHCl_3$/MeOH = 30), pale yellow oil, IR (neat) ν 699, 738, 815, 875, 969, 1028, 1119, 1155, 1221, 1295, 1369, 1408, 1454, 1512, 1607, 1898, 1954, 2820, 2920, 2964, 3028, 3061, 3324 cm^{-1}; ^{1}H NMR (400 MHz, $CDCl_3$) δ 1.09–1.40 (1H, br-s), 1.40–1.65 (2H, m), 2.59–2.82 (2H, m), 3.28 (1H, ddd, *J* = 4.2, 5.7, 6.7 Hz), 3.69 (1H, *J* = 13.3 Hz), 3.74 (1H, *J* = 13.3 Hz), 4.04 (1H, d, *J* = 4.2 Hz), 7.01 (2H, t, J = 8.7 Hz), 7.20–7.34 (7H, m); ^{13}C NMR (100 MHz, $CDCl_3$) δ 27.4, 46.1, 53.8, 56.6, 58.1, 115.1 (CH×2, d, *J* = 21.7 Hz), 126.9, 127.97 (CH×2), 128.04 (CH×2, d, *J* = 8.2 Hz), 128.4 (CH×2), 131.2 (d, *J* = 3.1 Hz), 140.2, 162.3 (d, *J* = 245.8 Hz); MS (EI) m/z: $[M]^+$ calcd for $C_{17}H_{18}FNO$ 271.1372; Found 271.1367

1-3. Cyclization of epoxy amines

Optimized condition of intramolecular aminolysis of *cis*-3,4-epoxy amines

To a solution of *cis*-3,4-epoxy amine (1 eq) in $(CH_2Cl)_2$ (0.2 M) was added $La(OTf)_3$ (5 mol%) at room temperature, and the mixture was stirred under reflux. Upon completion of the reaction, the mixture was cooled to 0 °C and saturated aqueous $NaHCO_3$ was added. The mixture was extracted thrice with CH_2Cl_2. The combined organic layers were dried over Na_2SO_4, filtered, and then concentrated under reduced pressure. The resulting residue was purified using column chromatography to yield the corresponding azetidine.

(*S**)-1-((*S**)-1-benzylazetidin-2-yl)propan-1-ol (**2aa**)

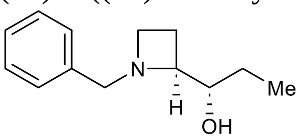

Yield : 87%, column chromatography (silica gel, $CHCl_3$/MeOH = 80), pale yellow oil, IR (neat) ν 699, 735, 971, 1028, 1171, 1362, 1454, 1496, 2873, 2931, 2960, 3028, 3062, 3410 cm^{-1}; ^{1}H NMR (400 MHz, $CDCl_3$) δ 7.06-7.38 (5H, m), 3.86 (1H, d, *J* = 13.1 Hz), 3.52 (1H, d, *J* = 13.1 Hz), 3.17-3.44 (3H, m), 2.84-3.14 (1H, br-s), 2.85 (1H, ddd, *J* = 7.2, 8.9, 9.4 Hz), 1.83-2.24 (2H, m), 1.22-1.47 (2H, m), 0.95 (3H, t, *J* = 7.4 Hz); ^{13}C NMR (100 MHz, $CDCl_3$) δ 10.4, 19.6, 27.9, 51.0, 63.5, 69.1, 75.0, 127.1, 128.3 (CH×2), 128.5 (CH×2), 138.3; HRMS (EI) m/z: $[M]^+$ calcd for $C_{13}H_{19}NO_2$ 205.1467; Found 205.1483

Acetylation for structure determination

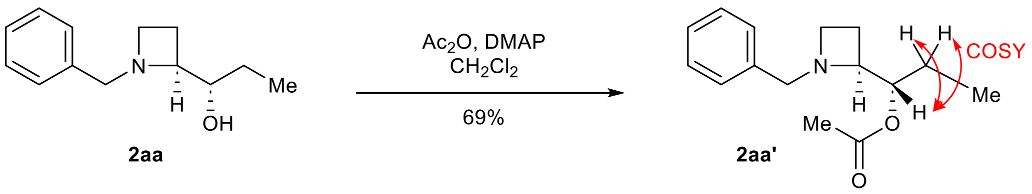

To a solution of **2aa** (34.4 mg, 0.168 mmol) in CH_2Cl_2 (0.1 M) were added DMAP (51.6 mg, 0.422 mmol) and Ac_2O (19 μL, 0.201 mmol) at 0 °C, and the mixture was stirred for 1.5 h at room temperature. the mixture was cooled to 0 °C and saturated aqueous $NaHCO_3$ was added at 0 °C. The mixture was extracted thrice with CH_2Cl_2. The combined organic layers were dried over Na_2SO_4, filtered, and then concentrated under reduced pressure. The resulting residue was purified using column chromatography (silica gel, $CHCl_3$/MeOH = 50) to yield *O*-acetylated azetidine **2aa’** (28.4 mg, 0.115 mmol, 69%) as a pale-yellow oil.

2aa’: IR (neat) ν 701, 738, 795, 905, 963, 1028, 1087, 1154, 1239, 1370, 1454, 1496, 1604, 1732, 2829, 2968, 3028, 3062 cm^{-1}; ^{1}H NMR (400 MHz, $CDCl_3$) δ 0.88 (3H, t, *J* = 7.5 Hz), 1.46 (1H, m), 1.62 (1H, dqd, *J* = 4.2, 7.5, 14.3 Hz), 1.85–1.98 (2H, m), 1.99 (3H, s), 2.75 (1H, dt, *J* = 7.1, 8.6 Hz), 3.22 (1H, dt, *J* = 3.4, 7.2 Hz), 3.31 (1H, q, *J* = 8.0 Hz), 3.38 (1H, d, *J* = 13.0 Hz), 3.84 (1H, d, *J* = 13.0 Hz), 4.93 (1H, dt, *J* = 4.2, 7.9 Hz), 7.19–7.33 (5H, m); ^{13}C NMR (100 MHz, $CDCl_3$) δ 9.48, 20.3, 21.1, 23.3, 51.0, 63.3, 67.1, 79.0, 126.9, 128.2 (CH×2), 128.7 (CH×2), 138.4, 170.7; MS (EI) m/z: $[M]^+$ calcd for $C_{15}H_{21}NO_2$ 247.1572; Found 247.1580

(*S**)-1-((*S**)-1-(4-methoxybenzyl)azetidin-2-yl)propan-1-ol (**2ba**)

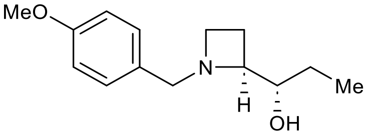

Yield: 83%, column chromatography (silica gel, $CHCl_3$/MeOH = 100→50), pale yellow oil, IR (neat) ν 826, 972, 1037, 1108, 1176, 1248, 1301, 1361, 1464, 1513, 1612, 2835, 2959, 3410 cm^{-1}; ^1H NMR (400 MHz, $CDCl_3$) δ 0.95 (3H, t, *J* = 7.4 Hz), 1.28–1.43 (2H, m), 1.89–2.12 (2H, m), 2.85 (1H, dt, *J* = 7.2, 8.9 Hz), 3.17–3.37 (3H, m), 3.47 (1H, d, *J* = 12.9 Hz), 3.76 (1H, d, *J* = 12.9 Hz), 3.79 (3H, s), 6.86 (2H, d, *J* = 8.7 Hz), 7.20 (2H, d, *J* = 8.7 Hz); ^{13}C NMR (100 MHz, $CDCl_3$) δ 10.4, 19.6, 27.9, 50.7, 55.2, 62.8, 69.1, 75.0, 113.8, 129.7, 130.3, 158.7; MS (EI) m/z: $[M]^+$ calcd for $C_{14}H_{21}NO_2$ 235.1572; Found 235.1573

(*S**)-1-((*S**)-1-(4-(trifluoromethyl)benzyl)azetidin-2-yl)propan-1-ol (**2ca**)

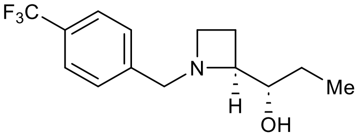

Yield: 92%, column chromatography (silica gel, $CHCl_3$/MeOH = 100), pale yellow oil, IR (neat) ν 826, 972, 1019, 1066, 1125, 1164, 1327, 1417, 1620, 2876, 2965, 3399 cm^{-1}; ^1H NMR (400 MHz, $CDCl_3$) δ 0.97 (3H, t, *J* = 7.4 Hz), 1.28–1.44 (2H, m), 1.95–2.20 (2H, m), 2.72–2.95 (1H, br-s), 2.83 (1H, dt, *J* = 7.3, 8.8 Hz), 3.24–3.44 (3H, m), 3.57 (1H, d, *J* = 13.6 Hz), 3.95 (1H, J = 13.6 Hz), 7.40 (2H, d, *J* = 8. 0 Hz), 7.57 (2H, d, *J* = 8.0 Hz); ^{13}C NMR (100 MHz, $CDCl_3$) δ 10.3, 19.7, 27.9, 51.2, 63.0, 69.3, 75.1, 124.2 (q, *J* = 272 Hz), 125.3 (CH×2, q, *J* = 3.7 Hz), 128.6 (CH×2), 129.4 (q, *J* = 32.3 Hz), 142.4 (q, *J* = 1.0 Hz); MS (EI) m/z: $[M]^+$ calcd for $C_{14}H_{18}NOF_3$ 273.1340; Found 273.1346

(*S**)-1-((*S**)-1-butylazetidin-2-yl)propan-1-ol (**2da**)

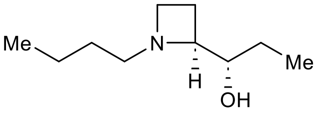

Yield: 80%, column chromatography (NH-silica gel, hexane/$CHCl_3$ = 20→7), pale yellow oil, IR (neat) ν 975, 1181, 1376, 1463, 2872, 2930, 2958, 3400 cm^{-1}; ^1H NMR (400 MHz, $CDCl_3$) δ 0.86 (3H, t, *J* = 7.1 Hz), 0.96 (3H, t, *J* = 7.4 Hz), 1.22–1.45 (6H, m), 1.90–2.12 (2H, m), 2.27 (1H, m), 2.58–2.73 (1H, m), 2.76 (1H, dt, *J* = 7.1, 8.9 Hz), 3.14 (1H, dt, *J* = 3.1, 8.1 Hz), 3.26 (1H, ddd, *J* = 3.4, 5.4, 7.8 Hz), 3.36 (1H, ddd, *J* = 3.0, 7.2, 8.1 Hz); ^{13}C NMR (100 MHz, $CDCl_3$) δ 10.4, 14.0, 19.7, 20.4, 28.2, 29.6, 51.0, 59.5, 69.2, 74.7; MS (EI) m/z: $[M]^+$ calcd for $C_{10}H_{21}NO$ 171.1623; Found 171.1629

(*S**)-1-((*S**)-1-(*tert*-butyl)azetidin-2-yl)propan-1-ol (**2ea**)

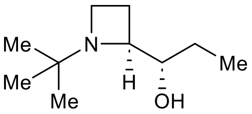

Yield: 86%, column chromatography (NH-silica gel, hexane/$CHCl_3$ = 20→5), colorless oil, IR (neat) ν 973, 1038, 1073, 1234, 1362, 1389, 1463, 2873, 2966, 3410 cm^{-1}; ^1H NMR (400 MHz, $CDCl_3$) δ 0.96 (3H, t, *J* = 7.4 Hz), 0.99 (9H, s), 1.42 (2H, quint, *J* = 7.4), 1.83 (1H, ddd, *J* = 6.9, 8.0, 11.0 Hz), 2.05 (1H, ddd, *J* = 5.7, 8.5, 11.0 Hz), 3.12–3.22 (2H, m), 3.30 (1H, dt, *J* = 4.0, 6.6 Hz), 3.41 (1H, ddd, *J* = 4.0, 6.9, 8.8 Hz), 3.53 (1H, br-s); ^{13}C NMR (100 MHz, $CDCl_3$) δ 10.5, 20.0, 25.6 (CH_3×3), 27.6, 43.7, 52.0, 61.7, 75.5; MS (EI) m/z: $[M]^+$ calcd for $C_{10}H_{21}NO$ 171.1623; Found 171.1635

(*S**)-1-((*S**)-1-allylazetidin-2-yl)propan-1-ol (**2fa**)

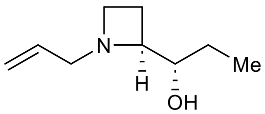

Yield: <63%, column chromatography (NH-silica gel, hexane/$CHCl_3$ = 10→5), pale yellow oil, IR (neat) ν 798, 920, 976, 1036, 1097, 1176, 1246, 1305, 1340, 1419, 1463, 1643, 2874, 2933, 2960, 3078, 3400 cm^{-1}; ^{1}H NMR (400 MHz, $CDCl_3$) δ 0.96 (3H, t, *J* = 7.4 Hz), 1.28–1.46 (2H, m), 1.91–2.12 (2H, m), 2.82 (1H, dt, *J* = 7.4, 8.9 Hz), 2.97 (1H, dd, J = 6.9, 13.5 Hz), 3.08–3.58 (5H, m), 5.10 (1H, d, *J* = 10.1 Hz), 5.17 (1H, ddd, *J* = 1.7, 3.2, 14.0 Hz), 5.78 (1H, dddd, *J* = 5.4, 6.9, 10.3, 17.1 Hz); ^{13}C NMR (100 MHz, $CDCl_3$) δ 10.3, 19.8, 27.7, 50.7, 62.1, 69.1, 75.2, 117.0, 134.7; MS (ESI) m/z: $[M+H]^+$ calcd for $C_9H_{18}NO$ 156.1383; Found 156.1381

tert-butyl (5-((*S**)-2-((*S**)-1-hydroxypropyl)azetidin-1-yl)pentyl)carbamate (**2ga**)

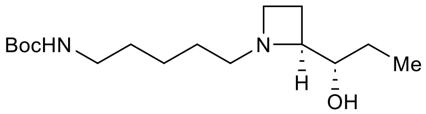

Yield: <87%, column chromatography (NH-silica gel, hexane/CH_3Cl = 8→2), colorless oil, IR (neat) n 666, 755, 871, 976, 1040, 1174, 1250, 1365, 1391, 1455, 1531, 1694, 2860, 2932, 2972, 3346 cm^{-1}; ^{1}H NMR (400 MHz, $CDCl_3$) d 0.95 (3H, dd, *J* = 1.5, 7.3 Hz), 1.19–1.39 (7H, m), 1.41–1.49 (1H, m), 1.44 (9H, s), 1.89–2.10 (2H, m), 2.24–2.40 (1H, m), 2.57–2.67 (1H, m), 2.74 (1H, dt, *J* = 7.3, 8.8 Hz), 3.04–3.16 (3H, m), 3.18–3.32 (2H, m), 3.34 (1H, ddd, *J* = 3.0, 7.1, 8.1 Hz), 4.42–4.74 (1H, br-s); ^{13}C NMR (100 MHz, $CDCl_3$) d 10.4, 19.7, 24.5, 27.2, 28.2, 28.4 (CH_3×3), 29.9, 40.5, 51.0, 59.6, 69.1, 74.8, 79.0, 156.0; MS (EI) m/z: $[M]^+$ calcd for $C_{16}H_{32}N_2O_3$ 300.2413; Found 300.2418

(*S**)-1-((*S**)-1-(5-((4-methoxybenzyl)oxy)pentyl)azetidin-2-yl)propan-1-ol (**2ha**)

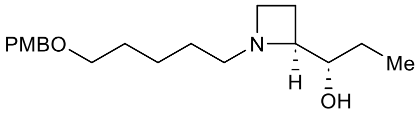

Yield: 83%, column chromatography (silica gel, $CHCl_3$/MeOH = 20→8), pale yellow oil, IR (neat) ν 688, 756, 821, 891, 975, 1036, 1097, 1173, 1247, 1302, 1361, 1464, 1514, 1586, 1613, 1881, 2063, 2857, 2934, 3418 cm^{-1}; ^{1}H NMR (400 MHz, C_6D_6) δ 1.01 (3H, t, *J* = 7.4 Hz), 1.14–1.48 (6H, m), 1.50–1.68 (3H, m), 1.97 (1H, ddd, *J* = 8.5, 10.4, 17.5 Hz), 2.12 (1H, ddd, *J* = 4.8, 7.5, 12.4 Hz), 2.43 (1H, dt, *J* = 6.9, 8.9 Hz), 2.55 (1H, ddd, *J* = 6.9, 8.5, 11.4 Hz), 2.86 (1H, dt, *J* = 3.3, 8.0 Hz), 3.17 (1H, ddd, *J* = 2.7, 7.0, 8.5 Hz), 3.24 (1H, td, *J* = 4.1, 8.5 Hz), 3.31 (3H, s), 3.32–3.40 (2H, m), 4.35 (2H, s), 6.83 (2H, d, *J* = 8.6 Hz), 7.26 (2H, d, *J* = 8.6 Hz); ^{13}C NMR (100 MHz, C_6D_6) δ 10.8, 19.7, 24.3, 27.6, 28.8, 30.1, 51.1, 54.7, 59.8, 69.7, 70.0, 72.7, 74.5, 114.0 (CH×2), 129.3 (CH×2), 131.5, 159.7; MS (EI) m/z: $[M]^+$ calcd for $C_{19}H_{31}NO_3$ 321.2304; Found 321.2317

(*S**)-1-((*S**)-1-(3-((*tert*-butyldimethylsilyl)oxy)propyl)azetidin-2-yl)propan-1-ol (**2ia**)

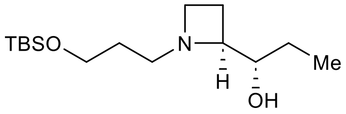

Yield: 80%, column chromatography (silica gel, $CHCl_3$/MeOH = 50→30→10), pale yellow oil, IR (neat) ν 663, 718, 776, 837, 938, 974, 1006, 1099, 1178, 1254, 1362, 1388, 1471, 2857, 2955, 3428 cm^{-1}; ^{1}H NMR (400 MHz, $CDCl_3$) δ 0.04 (6H, s), 0.89 (9H, s), 0.95 (3H, t, *J* = 7.4 Hz), 1.27–1.46 (2H, m), 1.55 (2H, ddd, *J* = 6.3, 7.7, 12.9 Hz), 2.40 (1H, td, *J* = 6.4, 11.5 Hz), 2.67–2.86 (2H, m), 3.16 (2H, dt, *J* = 3.3, 8.0 Hz), 3.26 (1H, ddd, J = 3.6, 5.0, 8.0 Hz), 3.37 (1H, dt, *J* = 3.0, 7.7 Hz), 3.58–3.71 (2H, m); ^{13}C NMR (100 MHz, $CDCl_3$) δ –5.36, –5.35, 10.4, 18.3, 19.5, 25.9 (CH_3×3), 28.1, 30.8, 51.1, 56.1, 60.9, 69.1, 74.7; MS (EI) m/z: $[M]^+$ calcd for $C_{15}H_{33}NO_2Si$ 287.2281; Found 287.2273

3-((*S**)-2-((*S**)-1-hydroxypropyl)azetidin-1-yl)propanenitrile (**2ja**)

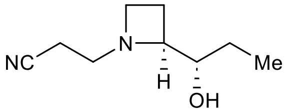

Yield: 92%, column chromatography (silica gel, $CHCl_3$/MeOH = 20), pale yellow oil, IR (neat) ν 974, 1097, 1177, 1227, 1370, 1421, 1460, 2249, 2844, 2963, 3409 cm^{-1}; ^{1}H NMR (400 MHz, $CDCl_3$) δ 0.97 (3H, t, *J* = 7.4 Hz), 1.28–1.42 (2H, m), 1.95–2.15 (2H, m), 2.41 (2H, dd, *J* = 6.5, 7.2 Hz), 2.51 (1H, br-s), 2.69 (1H, td, *J* = 6.4, 12.0 Hz), 2.87 (1H, dt, *J* = 6.8, 8.9 Hz), 2.93 (1H, td, *J* = 7.3, 12.0 Hz), 3.23 (1H, dt, *J* = 4.6, 8.1 Hz), 3.30–3.41 (1H, m), 3.49 (1H, ddd, *J* = 3.2, 7.2, 8.0 Hz); ^{13}C NMR (100 MHz, $CDCl_3$) δ 10.2, 17.0, 19.6, 27.3, 51.5, 54.5, 69.5, 75.5, 118.7; MS (EI) m/z: $[M]^+$ calcd for $C_9H_{16}N_2O$ 168.1263; Found 168.1261

(*S**)-1-((*S**)-1-(3-(methylthio)propyl)azetidin-2-yl)propan-1-ol (**2ka**)

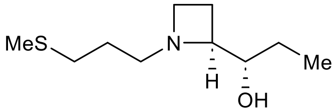

Yield: 88%, column chromatography (silica gel, $CHCl_3$/MeOH = 50→20→10), pale yellow oil, IR (neat) ν 974, 1038, 1172, 1227, 1372, 1442, 1727, 2836, 2918, 3417 cm^{-1}; ^{1}H NMR (400 MHz, $CDCl_3$) δ 0.96 (3H, t, *J* = 7.4 Hz), 1.29–1.46 (2H, m), 1.55–1.73 (2H, m), 1.92–2.12 (2H, m), 2.09 (3H, s), 2.45 (1H, ddd, *J* = 4.8, 7.3, 11.2 Hz), 2.48–2.60 (2H, m), 2.71–2.84 (2H, m), 3.16 (1H, dt, *J* = 3.6, 8.1 Hz), 3.28 (1H, ddd, *J* = 3.8, 5.2, 8.0 Hz), 3.38 (1H, ddd, *J* = 3.1, 6.9, 8.1 Hz); ^{13}C NMR (100 MHz, $CDCl_3$) δ 10.4, 15.5, 19.6, 26.8, 28.1, 31.9, 51.1, 58.1, 69.0, 75.0; MS (EI) m/z: $[M]^+$ calcd for $C_{10}H_{21}NOS$ 203.1344 ; Found 203.1343

(*S**)-1-((*S**)-1-phenylazetidin-2-yl)propan-1-ol (**2la**)

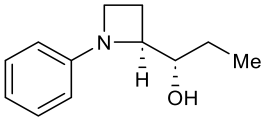

Yield: 39%, column chromatography (NH-silica gel, hexane/Et_2O = 10), white solid, m.p. 49.8–53.0 °C, IR (neat) ν 695, 753, 876, 969, 984, 1036, 1099, 1140, 1179, 1232, 1320, 1374, 1475, 1499, 1600, 1923, 2872, 2925, 2961, 3026, 3062, 3419 cm^{-1}; ^{1}H NMR (400 MHz, $CDCl_3$) δ 1.05 (3H, t, *J* = 7.4 Hz), 1.04–1.51 (1H, m), 1.56–1.67 (1H, m), 2.11–2.28 (1H, m), 2.21 (1H, d, *J* = 5.1 Hz), 2.35 (1H, ddd, *J* = 4.5, 8.7, 9.6 Hz), 3.54–3.75 (2H, m), 3.94–4.13 (2H, m), 6.71 (2H, d, *J* = 8.5 Hz), 6.78 (1H, t, *J* = 7.4 Hz), 7.22 (2H, dd, *J* = 7.4, 8.5 Hz); ^{13}C NMR (100 MHz, $CDCl_3$) δ 10.3, 20.3, 26.3, 51.4, 68.5, 77.7, 112.6 (CH×2), 118.2, 128.8 (CH×2), 152.9; MS (EI) m/z: $[M]^+$ calcd for $C_{12}H_{17}NO$ 191.1310; Found 191.1303

(2*R**,3*R**)-2-ethyl-1-phenylpyrrolidin-3-ol (**3la**)

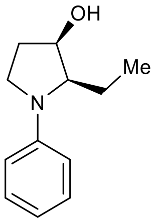

Yield: 19%, column chromatography (NH-silica gel, hexane/Et_2O = 10→5→2 then silica gel, hexane/EtOAc = 10), white amorphas, IR (neat) ν 692, 747, 826, 858, 907, 990, 1038, 1089, 1158, 1187, 1363, 1484, 1505, 1597, 2873, 2932, 2965, 3025, 3398 cm^{-1}; ^1H NMR (400 MHz, $CDCl_3$) δ 1.02 (3H, t, *J* = 7.6 Hz), 1.58–1.80 (3H, m), 2.03 (1H, qd, *J* = 7.6, 12.4 Hz), 2.17 (1H, dddd, J = 4.7, 6.0, 7.7, 12.3 Hz), 3.21 (1H, td, *J* = 7.6, 9.4 Hz), 3.58 (1H, dddd, *J* = 4.7, 8.3, 9.4, 14.5 Hz), 4.48 (1H, br-q, *J* = 6.7 Hz), 6.58 (2H, d, *J* = 8.5 Hz), 6.68 (1H, t, *J* = 7.4 Hz), 7.22 (2H, dd, *J* = 7.4, 8.5 Hz); ^{13}C NMR (100 MHz, $CDCl_3$) δ 11.2, 20.8, 32.1, 46.5, 62.1, 72.4, 112.4 (CH×2), 116.0, 129.1 (CH×2), 147.7; MS (EI) m/z: $[M]^+$ calcd for $C_{12}H_{17}NO$ 191.1310; Found 191.1304

(*S**)-1-((*R**)-1,2,3,4-tetrahydroquinolin-4-yl)propan-1-ol (**4a**)

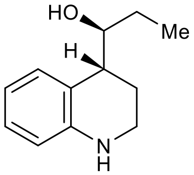

Yield: 21%, column chromatography (NH-silica gel, hexane/Et_2O = 10→5→2 then silica gel, hexane/EtOAc = 10→1), pale yellow oil, IR (neat) ν 747, 830, 894, 969, 1037, 1099, 1155, 1191, 1275, 1313, 1360, 1504, 1583, 1605, 1892, 1876, 2929, 2961, 3020, 3052, 3408, 3540 cm^{-1}; ^1H NMR (400 MHz, $CDCl_3$) δ 1.03 (3H, t, *J* = 7.4 Hz), 1.40–1.64 (2H, m), 1.71 (1H, dqd, J = 3.2, 7.5, 14.0 Hz), 1.87–2.20 (2H, m), 2.78 (1H, ddd, *J* = 3.7, 4.2, 7.2 Hz), 3.30 (1H, td, *J* = 4.5, 11.3 Hz), 3.38 (1H, ddd, J = 5.6, 9.8, 11.3 Hz), 3.64 (1H, ddd, J = 3.2, 6.9, 8.6 Hz), 3.72–4.14 (1H, br-s), 6.50 (1H, dd, J = 1.2, 8.3 Hz), 6.61 (1H, dt, J = 1.2, 7.4 Hz), 6.93–7.10 (2H, m); ^{13}C NMR (100 MHz, $CDCl_3$) δ 10.2, 24.0, 26.9, 38.6, 41.4, 75.7, 114.2, 116.3, 120.2, 127.7, 130.3, 144.7; MS (EI) m/z: $[M]^+$ calcd for $C_{12}H_{17}NO$ 191.1310; Found 191.1302

(*S**)-1-((*S**)-1-(4-methoxyphenyl)azetidin-2-yl)propan-1-ol (**2ma**)

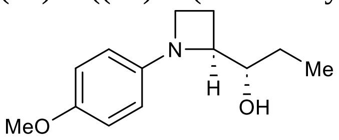

Yield: 38%, column chromatography (silica gel, hexane/EtOAc = 10→5), pale yellow oil, IR (neat) ν 798, 824, 970, 1038, 1116, 1179, 1239, 1464, 1513, 1580, 1616, 2875, 2959, 3433 cm^{-1}; ^1H NMR (400 MHz, $CDCl_3$) δ 1.03 (3H, t, *J* = 7.4 Hz), 1.37–1.50 (1H, m), 1.50–1.61 (1H, m), 2.10–2.22 (1H, m), 2.30 (1H, dddd, *J* = 4.2, 8.6, 9.6, 11.1 Hz), 2.38–2.54 (1H, br-s), 3.54 (1H, td, *J* = 7.5, 9.6 Hz), 3.58–3.67 (1H, br-s), 3.75 (3H, s), 3.92 (1H, td, *J* = 6.6, 8.5 Hz), 3.99 (1H, ddd, *J* = 4.2, 7.3, 8.7 Hz), 6.68 (2H, d, *J* = 9.0 Hz), 6.80 (2H, d, *J* = 9.0 Hz); ^{13}C NMR (100 MHz, $CDCl_3$) δ 10.2, 20.3, 26.3, 51.7, 55.8, 68.7, 77.7, 114.0 (CH×2), 114.4 (CH×2), 147.4, 152.7; MS (EI) m/z: $[M]^+$ calcd for $C_{13}H_{19}NO_2$ 221.1416; Found 221.1419

(2*R**,3*R**)-2-ethyl-1-(4-methoxyphenyl)pyrrolidin-3-ol (**3ma**)

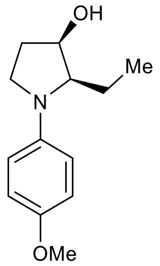

Yield: <12%, column chromatography (silica gel, hexane/EtOAc = 10→5→2), yellow oil, IR (neat) ν 812, 1040, 1082, 1179, 1239, 1362, 1464, 1509, 1618, 2869, 2961, 3419 cm^{-1}; ^{1}H NMR (400 MHz, $CDCl_3$) δ 1.01 (3H, t, *J* = 7.5 Hz), 1.51–1.88 (3H, m), 1.93–2.07 (1H, m), 2.09–2.26 (1H, m), 3.14 (1H, ddd, *J* = 6.7, 8.2, 9.1 Hz), 3.46 (1H, ddd, *J* = 3.9, 5.8, 8.4 Hz), 3.58 (1H, ddd, *J* = 5.2, 8.0, 9.1 Hz), 3.76 (3H, s), 4.46 (1H, q, *J* = 6.1), 6.58 (2H, d, *J* = 8.9 Hz), 6.85 (2H, d, *J* = 8.9 Hz); ^{13}C NMR (100 MHz, $CDCl_3$) δ 11.1, 20.8, 32.4, 48.0, 55.9, 63.3, 72.4, 112.8, 114.2, 114.9 (CH×2), 142.7, 151.5; MS (EI) m/z: $[M]^+$ calcd for $C_{13}H_{19}NO_2$ 221.1416; Found 221.1423

(*S**) -((*S**)-1-benzylazetidin-2-yl)(phenyl)methanol (**2ab**)

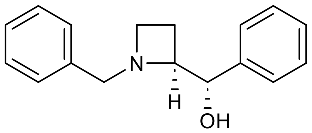

Yield: 82%, column chromatography (silica gel, hexane/EtOAc = 2), white solid, m.p. 108.7–109.1 °C, IR (neat) ν 699, 727, 760, 1020, 1036, 1166, 1275, 1449, 1495, 2854, 2962, 3029, 3059, 3165 cm^{-1}; ^{1}H NMR (400 MHz, $CDCl_3$) δ 2.01 (1H, m), 2.25 (1H, m), 2.84 (1H, dt, *J* = 7.2, 8.9 Hz), 3.24 (1H, d, *J* = 13.2 Hz), 3.22–3.30 (1H, m), 3.39 (1H, d, *J* = 13.2 Hz), 3.61 (1H, dt, *J* = 2.7, 8.0 Hz), 3.98 (1H, br-s), 4.56 (1H, d, *J* = 2.7 Hz), 7.08–7.45 (10H, m); ^{13}C NMR (100 MHz, $CDCl_3$) δ 20.1, 50.7, 62.3, 70.8, 74.0, 125.43 (CH×2), 127.00, 127.04, 128.26 (CH×2), 128.27 (CH×2), 128.5 (CH×2), 138.0, 143.2; MS (EI) m/z: $[M]^+$ calcd for $C_{17}H_{19}NO$ 253.1467; Found 253.1467

Acetylation for structure determination

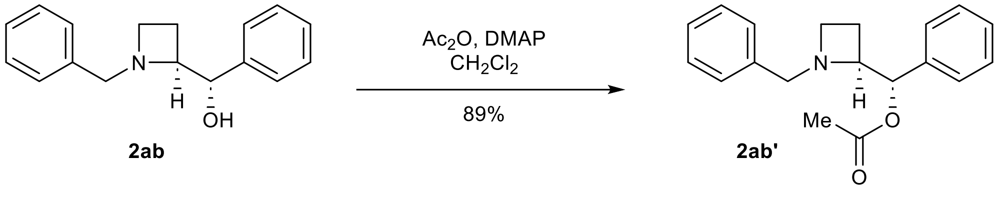

To a solution of **2ab** (19.3 mg, 0.076 mmol) in CH_2Cl_2 (0.05 M) were added DMAP (28.2 mg, 0.231 mmol) and Ac_2O (11 μL, 0.12 mmol) at 0 °C, and the mixture was stirred for 3 h at room temperature. the mixture was cooled to 0 °C and saturated aqueous $NaHCO_3$ was added at 0 °C. The mixture was extracted thrice with CH_2Cl_2. The combined organic layers were dried over Na_2SO_4, filtered, and then concentrated under reduced pressure. The resulting residue was purified using column chromatography (silica gel, $CHCl_3$/MeOH = 100) to yield *O*-acetylated azetidine **2ab'** (20.1 mg, 0.68 mmol, 89%) as white solid.

2ab': m.p. 55.2–58.7 °C; IR (neat) ν 700, 740, 793, 845, 908, 975, 1025, 1074, 1179, 1232, 1371, 1454, 1495, 1585, 1604, 1740, 2833, 2955, 3030, 3062 cm^{-1}; ^{1}H NMR (400 MHz, $CDCl_3$) δ 1.69–1.77 (1H, m), 1.85–1.96 (1H, m), 1.98 (3H, s), 2.75 (1H, ddd, *J* = 7.1, 8.2, 9.2 Hz), 3.23 (1H, ddd, *J* = 2.1, 6.5, 8.6 Hz), 3.42 (1H, d, *J* = 13.1 Hz), 3.55 (1H, td, *J* = 8.0, 8.2 Hz), 3.87 (1H, d, *J* = 13.1 Hz), 5.79 (1H,

d, J = 8.0 Hz), 7.17–7.40 (10H, m); ^{13}C NMR (100 MHz, $CDCl_3$) δ 20.3, 21.1, 50.6, 63.1, 68.2, 80.3, 126.9, 127.0 (CH×2), 128.1, 128.3 (CH×2), 128.4 (CH×2), 128.6 (CH×2), 137.6, 138.5, 170.0; MS (EI) m/z: $[M]^+$ calcd for $C_{19}H_{21}NO_2$ 295.1572; Found 295.1560

A doublet peak (4.56 ppm) of ***2ab*** *was shifted to 5.79 ppm of* ***2ab'*** *via acetylation. Therefore, the* ***2ab*** *and* ***2ab'*** *were assigned as azetidines.*

(*S**) -((*S**)-1-benzylazetidin-2-yl)(4-fluorophenyl)methanol (**2ac**)

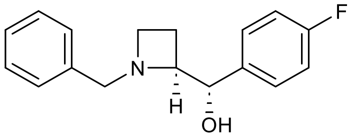

Yield: 62%, column chromatography (silica gel, $CHCl_3$/MeOH = 50), white solid, m.p. 124.8–127.1 °C, IR (neat) ν 694, 729, 831, 856, 936, 1037, 1092, 1165, 1222, 1451, 1506, 1601, 2851, 2963, 3170 cm^{-1}; ^{1}H NMR (400 MHz, $CDCl_3$) δ 1.99 (1H, dtd, J = 2.7, 8.2, 10.8 Hz), 2.19 (1H, tdd, J = 8.6, 8.9, 10.8), 2.85 (1H, dt, J = 7.2, 8.9 Hz), 3.19–3.34 (2H, m), 3.41 (1H, d, J = 13.2 Hz), 3.54 (1H, dt, J = 3.1, 8.0 Hz), 3.66–4.18 (1H, br-s), 4.53 (1H, d, J = 3.1 Hz), 7.00 (2H, t, J = 8.8 Hz), 7.14 (2H, d, J = 7.5 Hz), 7.17–7.40 (5H, m); ^{13}C NMR (100 MHz, $CDCl_3$) δ 20.0, 50.7, 62.4, 70.9, 73.6, 115.1 (CH×2, d, J = 21.3 Hz), 127.0 (CH×2, d, J = 8.0 Hz), 127.1, 128.3 (CH×2), 128.4 (CH×2), 137.9, 138.9 (d, J = 3.1 Hz), 162.0 (d, J = 244.8 Hz); MS (EI) m/z: $[M]^+$ calcd for $C_{17}H_{18}FNO$ 271.1372; Found 271.1384

2 Computational details

2.1 General computational information

All calculations were conducted using the Gaussian 09 Rev. E.01 suite of programs. Structure optimization and frequency calculation were carried out with the ωB97XD functional and LanL2DZ basis set for La atom and the 6-31G** basis set for all other atoms. Single point energy was obtained via calculation of the ωB97XD/6-31G**, SDD (La) geometries with the ωB97XD functional, the SDD basis set and the 6-311++G** basis set. Gibbs free energy (kJ/mol) was calculated based on ωB97XD/6-311++G**, SDD (La) single point energy and ωB97XD/6-31G**, LanL2DZ (La) frequency according to literature methods. Solvent effects were corrected by using Self-Consistent Reaction Field (SCRF) method using the Polarizable Continuum Model (PCM) together with dichloromethane and 1,2-dichloroethane as a solvent. All stationary points were characterized by frequency calculations to confirm their identity as either local minima (zero imaginary frequencies) or first-order saddle points (one imaginary frequency). For transition structures, an intrinsic reaction coordinate calculation (IRC) was conducted to ensure transit between desired reactant and product minima.

2.1.1 Naked lanthanum(III) coordination to epoxy amine

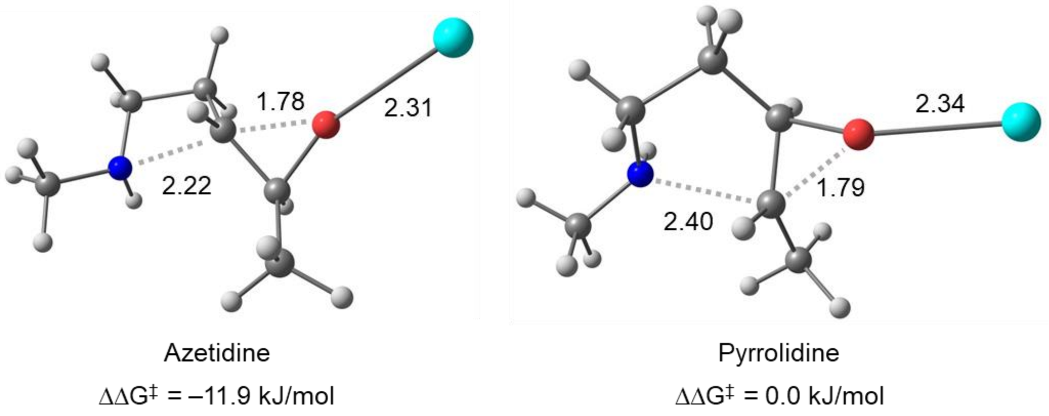

Supplementary Figure 1 Transition states of *trans*-epoxy amines with naked lanthanum(III) at 40 °C (PCM (dichloromethane)/ωB97XD/6-311++G**, SDD (La)//PCM (dichloromethane)/ωB97XD/6-31G**, LanL2DZ (La)); lanthanum (light blue), Oxygen (red), nitrogen (blue), carbon (gray), hydrogen (white)

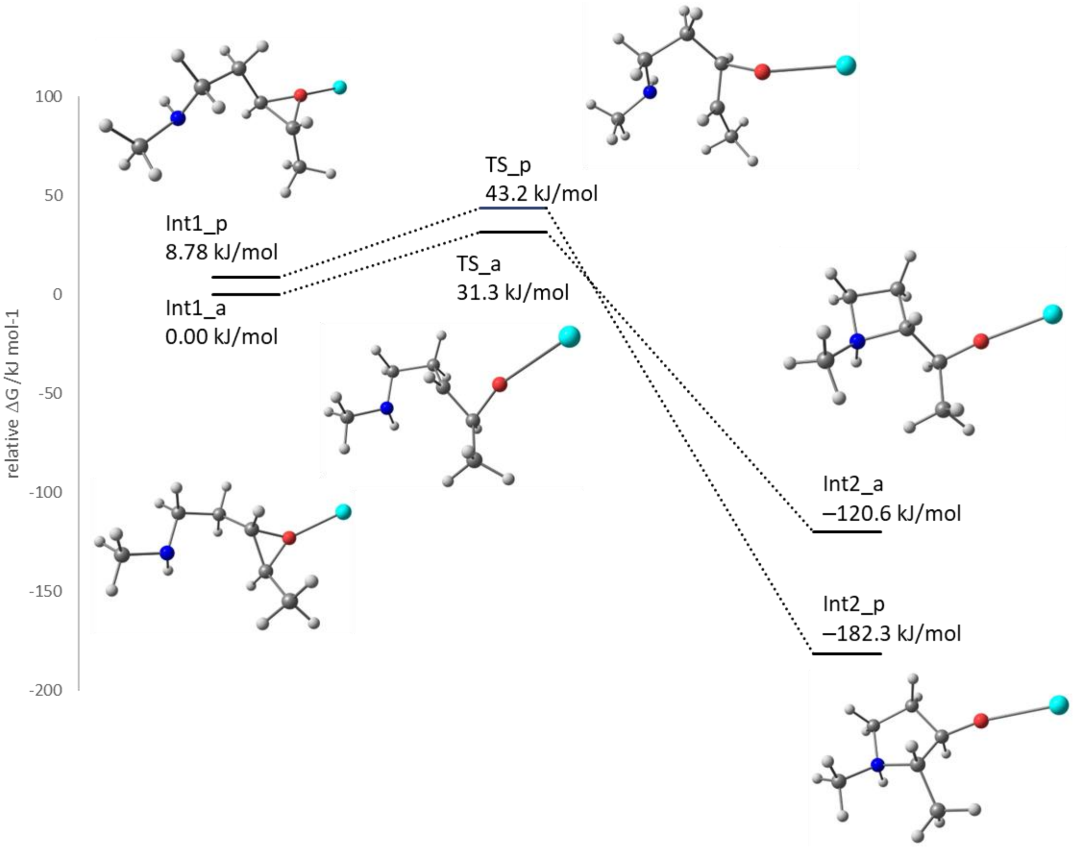

Supplementary Figure 2 Energy diagram of *trans*-epoxy amine intramolecular aminolysis with naked lanthanum(III) at 40 °C (PCM (dichloromethane)/ωB97XD/6-311++G**, SDD (La)//PCM (dichloromethane)/ωB97XD/6-31G**, LanL2DZ (La)); lanthanum (light blue), Oxygen (red), nitrogen (blue), carbon (gray), hydrogen (white)

Supplementary Table 1 The calculated energy values of *trans*-epoxy amine with naked lanthanum (III) (ZPE: zero point energy, kJ/mol)

	Azetidine formation			Pyrrolidine formation		
	Int1_a	TS_a	Int2_a	Int1_p	TS_p	Int2_p
relative ΔE	0.00	32.6	–131.1	8.24	42.4	–198.6
relative ΔE + ZPE	0.00	29.6	–123.1	8.29	40.5	–186.3
relative ΔH	0.00	28.8	–125.6	8.16	39.2	–189.1
relative ΔG	0.00	31.3	–120.6	8.78	43.2	–182.3

Supplementary Table 2 Cartesian coordinates (Angstroms) for Int1_a of *trans*-epoxy amines with naked lanthanum(III)

Optimized energy: E(RwB97XD) = –801.707026513
Number of imaginary frequencies: 0
Zero-point correction = 0.192733 (Hartree/Particle)
Thermal correction to Energy = 0.204675
Thermal correction to Enthalpy = 0.205666
Thermal correction to Gibbs Free Energy = 0.151556

3 1

C	-3.286549000	-1.309561000	-0.145173000
C	-1.191233000	-0.008489000	-0.328840000
C	-1.808445000	-1.250467000	0.247612000
C	-5.351488000	-0.044554000	-0.212403000
C	-0.967589000	1.202038000	0.453377000
H	-1.289877000	0.118931000	-1.405231000
H	-1.202629000	1.143462000	1.513457000
O	0.168928000	0.325827000	0.124335000
H	-3.364615000	-1.414466000	-1.234520000
H	-3.739643000	-2.215389000	0.290486000
H	-1.697614000	-1.245971000	1.337650000
H	-1.297017000	-2.138489000	-0.135949000
H	-5.382420000	-0.026997000	-1.306721000
H	-5.829657000	0.867796000	0.151561000
H	-5.947188000	-0.907506000	0.126582000
La	2.533135000	-0.208993000	-0.025318000
H	-3.944244000	0.007512000	1.244675000
N	-3.963982000	-0.078384000	0.232415000
C	-0.956988000	2.565628000	-0.156220000
H	-0.269819000	3.229420000	0.372939000
H	-0.689405000	2.527475000	-1.214461000
H	-1.964344000	2.981340000	-0.069068000

Supplementary Table 3 Cartesian coordinates (Angstroms) for TS_a of *trans*-epoxy amines with naked lanthanum(III)

Optimized energy: E(RwB97XD) = –801.694605939
Number of imaginary frequencies: 1
Imaginary frequencies = –514.1541 cm^{-1}
Zero-point correction = 0.191594 (Hartree/Particle)
Thermal correction to Energy = 0.203223
Thermal correction to Enthalpy = 0.204215
Thermal correction to Gibbs Free Energy = 0.151053

3 1

C	-3.333893000	-1.440160000	0.154274000
C	-1.433144000	-0.112195000	0.087409000
C	-1.913862000	-1.389588000	0.721815000
C	-4.582095000	0.313694000	-1.083681000
C	-0.907679000	1.025319000	0.828979000
H	-1.381835000	-0.052974000	-0.993762000
H	-1.147441000	1.028659000	1.893937000
O	0.237930000	0.209372000	0.563322000
H	-3.322722000	-1.899160000	-0.838205000
H	-4.047566000	-1.992930000	0.773429000
H	-1.909338000	-1.291568000	1.810336000
H	-1.304455000	-2.251571000	0.443360000
H	-4.150241000	0.013209000	-2.042388000
H	-4.744939000	1.392750000	-1.103774000
H	-5.551690000	-0.187942000	-0.968277000
La	2.392817000	-0.169983000	-0.174150000
H	-3.962899000	0.383568000	0.865459000
N	-3.646209000	-0.013544000	-0.013897000
C	-0.909851000	2.393284000	0.208290000
H	-0.184735000	3.040544000	0.707038000
H	-0.678654000	2.345596000	-0.859407000
H	-1.900910000	2.838540000	0.326989000

Supplementary Table 4 Cartesian coordinates (Angstroms) for Int2_a of *trans*-epoxy amines with naked lanthanum(III)

Optimized energy: E(RwB97XD) = –801.756946247
Number of imaginary frequencies: 0
Zero-point correction = 0.195750 (Hartree/Particle)
Thermal correction to Energy = 0.206928
Thermal correction to Enthalpy = 0.207920
Thermal correction to Gibbs Free Energy = 0.155555

3 1

C	-3.485530000	-1.552356000	0.179993000
C	-1.889147000	-0.176015000	-0.159608000
C	-1.955368000	-1.659663000	0.237373000
C	-4.248945000	0.700677000	-0.802576000
C	-0.959728000	0.748480000	0.623267000
H	-1.761690000	-0.025530000	-1.234559000
H	-1.168656000	0.608296000	1.696498000
O	0.332906000	0.323411000	0.343460000
H	-3.921350000	-1.894961000	-0.757958000
H	-4.072086000	-1.903629000	1.025953000
H	-1.578360000	-1.835624000	1.246523000
H	-1.504020000	-2.363909000	-0.457863000
H	-4.114256000	0.282407000	-1.799235000
H	-3.967331000	1.752830000	-0.795426000
H	-5.284913000	0.586369000	-0.485264000
La	2.414531000	-0.128142000	-0.103086000
H	-3.515983000	0.335677000	1.082464000
N	-3.380964000	-0.039300000	0.141002000
C	-1.153808000	2.217823000	0.266683000
H	-0.419718000	2.820314000	0.806456000
H	-1.014798000	2.374445000	-0.807528000
H	-2.146448000	2.581542000	0.550359000

Supplementary Table 5 Cartesian coordinates (Angstroms) for Int1_p of *trans*-epoxy amines with naked lanthanum(III)

Optimized energy: E(RwB97XD) = –801.703886390
Number of imaginary frequencies: 0
Zero-point correction = 0.192749 (Hartree/Particle)
Thermal correction to Energy = 0.204643
Thermal correction to Enthalpy = 0.205634
Thermal correction to Gibbs Free Energy = 0.151759

3 1

C	-1.069244000	0.707426000	-0.495834000
C	-1.613620000	-0.226692000	0.486494000
C	-2.401315000	-1.439135000	0.069938000
C	-3.799258000	-1.017485000	-0.388005000
H	-1.199424000	0.450215000	-1.544561000
H	-1.796654000	0.168017000	1.481114000
H	-1.883137000	-1.945786000	-0.749709000
H	-2.465151000	-2.140624000	0.907994000
H	-3.705711000	-0.417308000	-1.301580000
H	-4.380622000	-1.914049000	-0.660085000
O	-0.177609000	-0.252769000	0.170382000
La	2.170561000	-0.250602000	0.820784000
C	-0.821140000	2.148920000	-0.180339000
H	0.060223000	2.520595000	-0.709122000
H	-1.679978000	2.737705000	-0.511768000
H	-0.698455000	2.299396000	0.893720000
N	-4.445721000	-0.189314000	0.619941000
H	-4.626207000	-0.757103000	1.442996000
C	-5.708361000	0.369091000	0.151977000
H	-5.514500000	1.070675000	-0.666186000
H	-6.418625000	-0.388341000	-0.217332000
H	-6.187562000	0.923045000	0.962625000

Supplementary Table 6 Cartesian coordinates (Angstroms) for TS_p of *trans*-epoxy amines with naked lanthanum(III)

Optimized energy: E(RwB97XD) = – 801.690885605
Number of imaginary frequencies: 1
Imaginary frequencies = –464.3240 cm^{-1}
Zero-point correction = 0.192022 (Hartree/Particle)
Thermal correction to Energy = 0.203453
Thermal correction to Enthalpy = 0.204445
Thermal correction to Gibbs Free Energy = 0.151858

3 1

C	-1.333553000	0.713521000	-0.254390000
C	-1.022740000	-0.490768000	0.488459000
C	-1.898972000	-1.705308000	0.220935000
C	-3.286651000	-1.234006000	-0.262535000
H	-1.697650000	0.574532000	-1.264857000
H	-0.816110000	-0.326220000	1.547416000
H	-1.431338000	-2.343976000	-0.532380000
H	-1.992282000	-2.291617000	1.137690000
H	-3.287454000	-1.127490000	-1.353113000
H	-4.050560000	-1.982889000	-0.018140000
O	0.144722000	-0.296473000	-0.318141000
La	2.450651000	-0.021911000	-0.020791000
C	-1.073672000	2.096859000	0.217051000
H	-0.476677000	2.652598000	-0.508448000
H	-2.043486000	2.598063000	0.286797000
H	-0.602888000	2.116106000	1.200018000
N	-3.582044000	0.084876000	0.298008000
H	-3.665187000	0.016454000	1.309160000
C	-4.787354000	0.704311000	-0.241599000
H	-4.656050000	0.881530000	-1.313033000
H	-5.684655000	0.085321000	-0.103448000
H	-4.948595000	1.666494000	0.248991000

Supplementary Table 7 Cartesian coordinates (Angstroms) for Int2_p of *trans*-epoxy amines with naked lanthanum(III)

Optimized energy: E(RwB97XD) = –801.782682547
Number of imaginary frequencies: 0
Zero-point correction = 0.197450 (Hartree/Particle)
Thermal correction to Energy = 0.208290
Thermal correction to Enthalpy = 0.209281
Thermal correction to Gibbs Free Energy = 0.157786

3 1

C	-1.953206000	0.632178000	-0.152826000
C	-0.994161000	-0.410539000	0.443410000
C	-1.657867000	-1.740552000	0.053760000
C	-3.164872000	-1.458286000	0.067357000
H	-1.882108000	0.582710000	-1.244273000
H	-1.015703000	-0.298915000	1.540745000
H	-1.328075000	-2.002813000	-0.954376000
H	-1.390529000	-2.553255000	0.729368000
H	-3.655944000	-1.742453000	-0.862634000
H	-3.698459000	-1.913569000	0.900223000
O	0.290424000	-0.258308000	-0.027962000
La	2.463290000	0.031887000	-0.052727000
C	-1.767329000	2.047691000	0.346450000
H	-0.749274000	2.357961000	0.101095000
H	-2.451688000	2.754426000	-0.126911000
H	-1.886355000	2.105689000	1.432885000
N	-3.298880000	0.051979000	0.204371000
H	-3.460918000	0.259032000	1.192848000
C	-4.452828000	0.613833000	-0.548068000
H	-4.286300000	0.449481000	-1.612019000
H	-5.357898000	0.102348000	-0.223367000
H	-4.533966000	1.678487000	-0.335552000

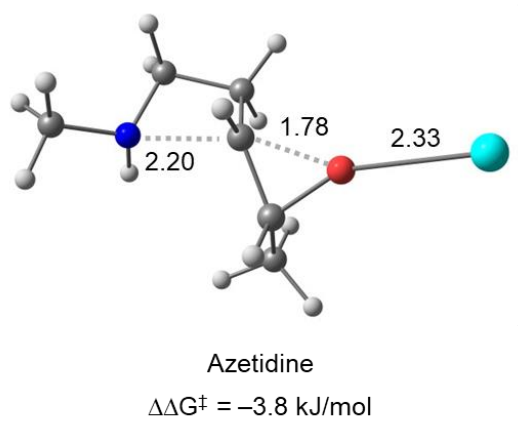

Supplementary Figure 3 Transition states of *cis*-epoxy amines with naked lanthanum(III) at 84 °C (PCM (dichloroethane)/ωB97XD/6-311++G**, SDD (La)//PCM (dichloroethane)/ωB97XD/6-31G**, LanL2DZ (La)); lanthanum (light blue), Oxygen (red), nitrogen (blue), carbon (gray), hydrogen (white)

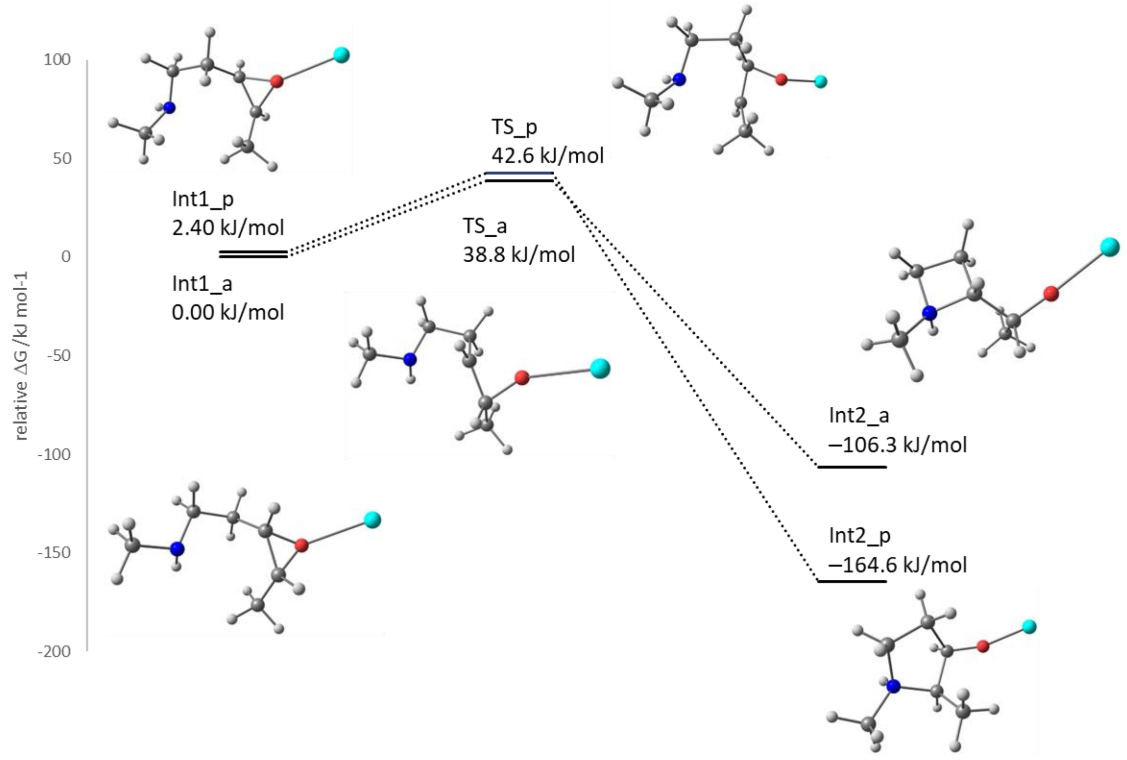

Supplementary Figure 4 Energy diagram of *cis*-epoxy amine intramolecular aminolysis with naked lanthanum(III) at 84 °C (PCM (dichloromethane)/ωB97XD/6-311++G**, SDD (La)//PCM (dichloromethane)/ωB97XD/6-31G**, LanL2DZ (La)); lanthanum (light blue), Oxygen (red), nitrogen (blue), carbon (gray), hydrogen (white)

Supplementary Table 8 The calculated energy values of *cis*-epoxy amine with naked lanthanum(III) (ZPE: zero point energy, kJ/mol)

	Azetidine formation			Pyrrolidine formation		
	Int1_a	TS_a	Int2_a	Int1_p	TS_p	Int2_p
relative ΔE	0.00	35.7	–122.1	–2.97	41.4	–189.9
relative ΔE + ZPE	0.00	34.7	–111.2	–1.06	38.9	–174.8
relative ΔH	0.00	33.0	–114.5	–2.24	37.3	–179.7
relative ΔG	0.00	38.8	–106.3	2.40	42.6	–164.6

Supplementary Table 9 Cartesian coordinates (Angstroms) for Int1_a of *cis*-epoxy amines with naked lanthanum(III)

Optimized energy: E(RwB97XD) = –801.719684656
Number of imaginary frequencies: 0
Zero-point correction = 0.191867 (Hartree/Particle)
Thermal correction to Energy = 0.207166
Thermal correction to Enthalpy = 0.208297
Thermal correction to Gibbs Free Energy = 0.141888

3 1

C	-3.295876000	-1.210222000	0.346569000
C	-1.083077000	-0.245311000	-0.235503000
C	-1.892041000	-0.882643000	0.858171000
C	-5.282172000	-0.291217000	-0.686137000
C	-0.902952000	1.195673000	-0.416714000
H	-0.968497000	-0.856019000	-1.129590000
C	-1.496316000	2.242781000	0.471851000
O	0.181357000	0.388343000	0.158738000
H	-3.219278000	-1.887184000	-0.513826000
H	-3.841764000	-1.762548000	1.129346000
H	-1.946046000	-0.229478000	1.733322000
H	-1.400803000	-1.807480000	1.174573000
H	-5.142719000	-0.847868000	-1.618862000
H	-5.789978000	0.645665000	-0.926794000
H	-5.943889000	-0.886926000	-0.036408000
H	-0.666399000	1.523078000	-1.425961000
H	-2.481780000	2.501618000	0.075968000
H	-1.614156000	1.893663000	1.498061000
La	2.599013000	-0.174743000	-0.066442000
H	-0.875084000	3.141037000	0.472430000
H	-4.118094000	0.600870000	0.711187000
N	-3.981647000	-0.004166000	-0.093658000

Supplementary Table 10 Cartesian coordinates (Angstroms) for TS_a of *cis*-epoxy amines with naked lanthanum(III)

Optimized energy: E(RwB97XD) = –801.706095589
Number of imaginary frequencies: 1
Imaginary frequencies = –533.5756 cm^{-1}
Zero-point correction = 0.191493 (Hartree/Particle)
Thermal correction to Energy = 0.206133
Thermal correction to Enthalpy = 0.207264
Thermal correction to Gibbs Free Energy = 0.143082

3 1

C	-3.407004000	-1.024075000	0.859701000
C	-1.494209000	-0.155648000	-0.110949000
C	-1.968133000	-0.660703000	1.227527000
C	-4.673480000	-0.457871000	-1.203323000
C	-0.877551000	1.143215000	-0.379055000
H	-1.471141000	-0.865980000	-0.929476000
C	-1.134716000	2.350944000	0.480199000
O	0.212147000	0.317750000	0.033968000
H	-3.442603000	-2.024391000	0.419254000
H	-4.111190000	-0.996128000	1.697238000
H	-1.926806000	0.124629000	1.984209000
H	-1.375165000	-1.509974000	1.572904000
H	-4.310067000	-1.344971000	-1.728939000
H	-4.803133000	0.345333000	-1.930932000
H	-5.649407000	-0.692715000	-0.759149000
H	-0.823498000	1.392800000	-1.440660000
H	-2.090440000	2.803628000	0.203253000
H	-1.152615000	2.102358000	1.542607000
La	2.472055000	-0.221464000	-0.091303000
H	-0.351270000	3.092653000	0.309177000
H	-3.950798000	0.841758000	0.198248000
N	-3.687949000	-0.051816000	-0.207116000

Supplementary Table 11 Cartesian coordinates (Angstroms) for Int2_a of *cis*-epoxy amines with naked lanthanum(III)

Optimized energy: E(RwB97XD) = –801.766207677
Number of imaginary frequencies: 0
Zero-point correction = 0.196022 (Hartree/Particle)
Thermal correction to Energy = 0.210069
Thermal correction to Enthalpy = 0.211200
Thermal correction to Gibbs Free Energy = 0.147922

3 1

C	-3.384158000	-1.279857000	0.722114000
C	-1.857621000	-0.181242000	-0.271472000
C	-1.854990000	-1.230595000	0.852531000
C	-4.235896000	0.133578000	-1.260364000
C	-0.966305000	1.062170000	-0.187070000
H	-1.734063000	-0.649049000	-1.250087000
C	-1.180580000	1.912994000	1.060536000
O	0.338168000	0.586587000	-0.247857000
H	-3.748393000	-2.078149000	0.076669000
H	-3.995244000	-1.202931000	1.618630000
H	-1.526849000	-0.833279000	1.813868000
H	-1.330778000	-2.158419000	0.634860000
H	-4.015369000	-0.685976000	-1.943819000
H	-4.044580000	1.089994000	-1.745324000
H	-5.273180000	0.079965000	-0.931375000
H	-1.178548000	1.683859000	-1.069944000
H	-2.173732000	2.373604000	1.092129000
H	-1.029274000	1.332290000	1.974830000
La	2.381065000	-0.170839000	-0.085301000
H	-0.455894000	2.729878000	1.051428000
H	-3.532823000	0.796951000	0.551945000
N	-3.358575000	0.009157000	-0.075211000

Supplementary Table 12 Cartesian coordinates (Angstroms) for Int1_p of *cis*-epoxy amines with naked lanthanum(III)

Optimized energy: E(RwB97XD) = –801.720817031
Number of imaginary frequencies: 0
Zero-point correction = 0.192596 (Hartree/Particle)
Thermal correction to Energy = 0.207447
Thermal correction to Enthalpy = 0.208578
Thermal correction to Gibbs Free Energy = 0.143933

3 1

C	0.901421000	0.938892000	-0.650339000
C	1.105620000	-0.497958000	-0.465732000
C	2.016413000	-1.108182000	0.559859000
C	3.381546000	-1.403046000	-0.078664000
H	0.918099000	-1.123999000	-1.336018000
H	2.124814000	-0.451261000	1.426236000
H	1.577580000	-2.045670000	0.916353000
H	3.995589000	-1.952267000	0.653353000
H	3.234153000	-2.072664000	-0.932693000
O	-0.131097000	0.127492000	0.019563000
La	-2.573916000	-0.079465000	0.042885000
H	0.580093000	1.251089000	-1.640743000
N	4.026541000	-0.186085000	-0.555802000
H	4.656539000	-0.423934000	-1.312779000
C	1.548015000	2.001429000	0.179458000
H	0.924899000	2.897812000	0.208170000
H	1.733722000	1.667242000	1.200650000
H	2.505280000	2.253796000	-0.283279000
C	4.780579000	0.512982000	0.476961000
H	4.104430000	0.881314000	1.254854000
H	5.541388000	-0.116046000	0.966943000
H	5.278041000	1.382070000	0.039383000

Supplementary Table 13 Cartesian coordinates (Angstroms) for TS_p of transition states of *cis*-epoxy amines with naked lanthanum(III)

Optimized energy: E(RwB97XD) = –801.703914187
Number of imaginary frequencies: 1
Imaginary frequencies = –507.4485 cm^{-1}
Zero-point correction = 0.190930 (Hartree/Particle)
Thermal correction to Energy = 0.205609
Thermal correction to Enthalpy = 0.206740
Thermal correction to Gibbs Free Energy = 0.142340

3 1

C	1.279267000	0.788287000	-0.454162000
C	1.014652000	-0.615288000	-0.179610000
C	1.955042000	-1.297830000	0.776983000
C	3.276232000	-1.387064000	-0.014423000
C	0.872123000	-1.216755000	-1.082290000
C	2.075845000	-0.720545000	1.697528000
C	1.604851000	-2.296206000	1.049439000
C	4.094693000	-1.697866000	0.643778000
C	3.173368000	-2.156605000	-0.785136000
O	-0.201976000	-0.017306000	0.247113000
La	-2.506197000	-0.015940000	-0.040999000
H	1.026954000	1.140891000	-1.450744000
N	3.570697000	-0.101305000	-0.669307000
H	3.820367000	-0.256649000	-1.638437000
C	1.662198000	1.827030000	0.533665000
H	0.849062000	2.550474000	0.635012000
H	1.912055000	1.421755000	1.512740000
H	2.520886000	2.374409000	0.137071000
C	4.621165000	0.675645000	-0.019336000
H	4.335370000	0.892445000	1.013428000
H	5.585467000	0.150552000	-0.002383000
H	4.751784000	1.625589000	-0.543508000

Supplementary Table 14 Cartesian coordinates (Angstroms) for Int2_p of *cis*-epoxy amines with naked lanthanum(III)

Optimized energy: E(RwB97XD) = –801.791999797
Number of imaginary frequencies: 0
Zero-point correction = 0.197617 (Hartree/Particle)
Thermal correction to Energy = 0.211027
Thermal correction to Enthalpy = 0.212158
Thermal correction to Gibbs Free Energy = 0.151508

3 1

C	2.346389000	0.739876000	-0.482982000
C	1.350494000	-0.440730000	-0.401156000
C	2.036263000	-1.410841000	0.584839000
C	3.541385000	-1.169825000	0.421531000
H	1.320272000	-0.910834000	-1.396569000
H	1.716877000	-1.158409000	1.598205000
H	1.765362000	-2.448051000	0.386446000
H	4.024011000	-0.858216000	1.347516000
H	4.093179000	-2.012045000	0.006595000
O	0.086183000	-0.060233000	-0.020760000
La	-2.088511000	-0.046287000	0.287642000
H	2.246803000	1.299570000	-1.414978000
N	3.656469000	-0.015369000	-0.557282000
H	3.692164000	-0.423709000	-1.493904000
C	2.281762000	1.674003000	0.709216000
H	1.265389000	2.066579000	0.759348000
H	2.488869000	1.162551000	1.653316000
H	2.965223000	2.517975000	0.604068000
C	4.898778000	0.786092000	-0.398307000
H	4.922458000	1.213214000	0.602114000
H	5.751507000	0.123015000	-0.537845000
H	4.908650000	1.574678000	-1.149589000

2.1.2 Dimethylamine-coordinated lanthanum(III) coordination to epoxy amine

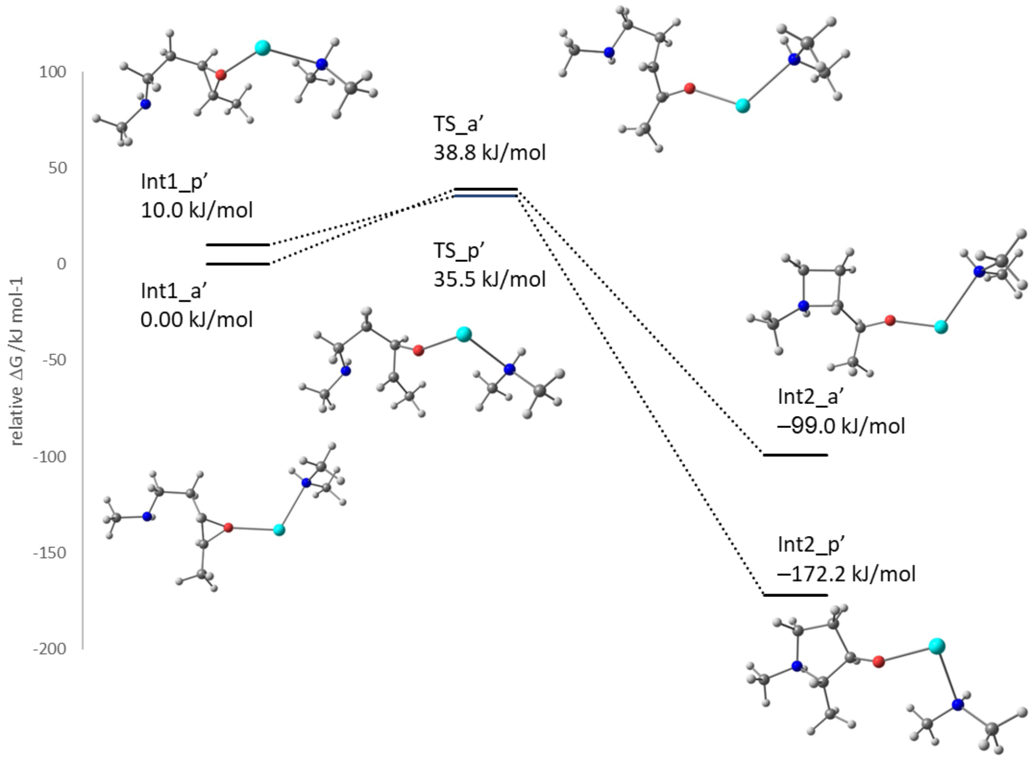

Supplementary Figure 5 Energy diagram of *trans*-epoxy amine intramolecular aminolysis with dimethylamine-coordinated lanthanum(III) at 40 °C (PCM (dichloromethane)/ωB97XD/6-311++G**, SDD (La)//PCM (dichloromethane)/ωB97XD/6-31G**, LanL2DZ (La)); lanthanum (light blue), Oxygen (red), nitrogen (blue), carbon (gray), hydrogen (white)

Supplementary Table 15 The calculated energy values of *trans*-epoxy amine with dimethylamine-coordinated lanthanum(III) (ZPE: zero point energy, kJ/mol)

	Azetidine formation			Pyrrolidine formation		
	Int1_a’	TS_a’	Int2_a’	Int1_p’	TS_p’	Int2_p’
relative ΔE	0.00	33.3	–117.1	0.42	29.9	–196.6
relative ΔE + ZPE	0.00	32.2	–107.2	4.83	30.7	–182.2
relative ΔH	0.00	30.4	–110.3	3.02	28.7	–186.4
relative ΔG	0.00	38.8	–99.0	10.0	35.5	–172.2

Supplementary Table 16 Cartesian coordinates (Angstroms) for Int1_a' of *trans*-epoxy amines with dimethylamine-coordinated lanthanum(III)

Optimized energy: E(RwB97XD) = –936.896279181
Number of imaginary frequencies: 0
Zero-point correction = 0.289750 (Hartree/Particle)
Thermal correction to Energy = 0.307445
Thermal correction to Enthalpy = 0.308437
Thermal correction to Gibbs Free Energy = 0.241363

3 1

C	-3.937839000	2.063615000	0.293495000
C	-2.093509000	0.419647000	0.396680000
C	-2.490664000	1.771644000	-0.117325000
C	-6.200205000	1.192666000	0.266308000
C	-2.287242000	-0.793725000	-0.384305000
H	-2.073809000	0.304237000	1.479059000
H	-2.647157000	-0.662568000	-1.401000000
O	-0.935209000	-0.223927000	-0.236728000
H	-4.012334000	2.055614000	1.388236000
H	-4.203483000	3.083608000	-0.028998000
H	-2.391067000	1.792562000	-1.208433000
H	-1.830744000	2.540876000	0.294280000
H	-6.221683000	1.019227000	1.347165000
H	-6.842631000	0.445577000	-0.205849000
H	-6.631228000	2.189046000	0.077315000
La	1.449067000	-0.778979000	-0.053833000
H	-4.845425000	1.099782000	-1.236253000
N	-4.835425000	1.039350000	-0.222090000
C	-2.533292000	-2.126274000	0.245138000
H	-2.142662000	-2.936132000	-0.375059000
H	-2.094541000	-2.184114000	1.244274000
H	-3.614017000	-2.262080000	0.336122000
N	3.223761000	1.225967000	-0.062833000
H	2.768500000	2.036844000	-0.480029000
C	4.411318000	0.917156000	-0.891347000
H	4.108275000	0.750989000	-1.926025000
H	5.138226000	1.734296000	-0.863014000
H	4.887968000	0.010649000	-0.507644000
C	3.625174000	1.614258000	1.308654000
H	4.324822000	2.455227000	1.294670000
H	2.743289000	1.898607000	1.886000000
H	4.112591000	0.766226000	1.794677000

Supplementary Table 17 Cartesian coordinates (Angstroms) for TS_a’ of *trans*-epoxy amines with dimethylamine-coordinated lanthanum(III)

Optimized energy: E(RwB97XD) = –936.883597432
Number of imaginary frequencies: 1
Imaginary frequencies = –531.2955 cm^{-1}
Zero-point correction = 0.289321 (Hartree/Particle)
Thermal correction to Energy = 0.306341
Thermal correction to Enthalpy = 0.307333
Thermal correction to Gibbs Free Energy = 0.243054

3 1

C	-3.861017000	1.872881000	-0.339711000
C	-2.290637000	0.217286000	0.038537000
C	-2.433724000	1.497821000	-0.739245000
C	-5.578827000	0.489741000	0.809960000
C	-1.932696000	-1.067812000	-0.546892000
H	-2.325505000	0.260588000	1.121655000
H	-2.102252000	-1.147360000	-1.623318000
O	-0.673915000	-0.457529000	-0.254487000
H	-3.854752000	2.397848000	0.619856000
H	-4.389465000	2.497872000	-1.066720000
H	-2.342374000	1.300094000	-1.810507000
H	-1.688517000	2.241863000	-0.452361000
H	-5.241322000	0.805475000	1.800488000
H	-5.937421000	-0.538813000	0.878968000
H	-6.412905000	1.134760000	0.504704000
La	1.613912000	-0.754131000	0.064956000
H	-4.723881000	0.134426000	-1.010901000
N	-4.457555000	0.546867000	-0.121524000
C	-2.240394000	-2.334427000	0.204346000
H	-1.640885000	-3.163750000	-0.179011000
H	-2.045386000	-2.210943000	1.272600000
H	-3.294634000	-2.589974000	0.070170000
N	3.312274000	1.325330000	-0.117887000
H	2.856921000	2.038224000	-0.685879000
C	4.568492000	0.965725000	-0.808342000
H	4.348582000	0.628562000	-1.822485000
H	5.252577000	1.818543000	-0.860646000
H	5.063333000	0.158704000	-0.263170000
C	3.588593000	1.941795000	1.198822000
H	4.279624000	2.785404000	1.107513000
H	2.657470000	2.296039000	1.645285000
H	4.037334000	1.194719000	1.858191000

Supplementary Table 18 Cartesian coordinates (Angstroms) for Int2_a’ of *trans*-epoxy amines with dimethylamine-coordinated lanthanum(III)

Optimized energy: E(RwB97XD) = –936.940881602
Number of imaginary frequencies: 0
Zero-point correction = 0.293529 (Hartree/Particle)
Thermal correction to Energy = 0.310049
Thermal correction to Enthalpy = 0.311041
Thermal correction to Gibbs Free Energy = 0.247733

3 1

C	-4.044210000	2.351166000	-0.356097000
C	-2.778526000	0.701352000	0.124194000
C	-2.533790000	2.099006000	-0.464858000
C	-5.238685000	0.464756000	0.926038000
C	-2.115897000	-0.500340000	-0.544193000
H	-2.632288000	0.664834000	1.206921000
H	-2.349951000	-0.460633000	-1.621439000
O	-0.748769000	-0.337530000	-0.358313000
H	-4.342025000	2.893210000	0.540918000
H	-4.581821000	2.724365000	-1.225023000
H	-2.182064000	2.056937000	-1.497201000
H	-1.897736000	2.761410000	0.117735000
H	-4.955758000	0.948534000	1.859972000
H	-5.208213000	-0.618098000	1.039823000
H	-6.236079000	0.783519000	0.625325000
La	1.335574000	-0.836373000	0.088458000
H	-4.537703000	0.421792000	-1.006125000
N	-4.276884000	0.869183000	-0.124230000
C	-2.603913000	-1.831050000	0.021787000
H	-2.040003000	-2.643944000	-0.441557000
H	-2.447438000	-1.867447000	1.104777000
H	-3.663304000	-2.010415000	-0.186108000
N	3.120309000	1.204740000	0.066654000
H	2.679858000	2.025455000	-0.344503000
C	4.300245000	0.876740000	-0.757383000
H	3.994939000	0.718605000	-1.793186000
H	5.049192000	1.674974000	-0.727376000
H	4.758148000	-0.041891000	-0.379337000
C	3.515600000	1.562038000	1.444408000
H	4.250977000	2.373313000	1.457765000
H	2.635433000	1.873296000	2.010825000
H	3.957927000	0.688109000	1.930308000

Supplementary Table 19 Cartesian coordinates (Angstroms) for Int1_p' of *trans*-epoxy amines with dimethylamine-coordinated lanthanum(III)

Optimized energy: E(RwB97XD) = –936.896119802
Number of imaginary frequencies: 0
Zero-point correction = 0.291429 (Hartree/Particle)
Thermal correction to Energy = 0.308437
Thermal correction to Enthalpy = 0.309428
Thermal correction to Gibbs Free Energy = 0.244689

3 1

C	-1.823399000	1.638446000	0.244849000
C	-1.968689000	0.648599000	1.311949000
C	-3.086116000	-0.354431000	1.416272000
C	-3.925305000	-0.573017000	0.160092000
H	-2.576806000	1.601845000	-0.536757000
H	-1.468827000	0.876113000	2.251144000
H	-2.656807000	-1.307787000	1.742577000
H	-3.732614000	-0.023580000	2.238039000
H	-3.269012000	-0.796907000	-0.691746000
H	-4.547530000	-1.470703000	0.314960000
O	-0.990548000	0.426612000	0.236618000
La	1.084768000	-0.848132000	-0.186656000
C	-1.105157000	2.933808000	0.452050000
H	-0.566212000	3.231246000	-0.450838000
H	-1.842729000	3.710575000	0.669301000
H	-0.409724000	2.875079000	1.291343000
N	-4.710673000	0.605167000	-0.173570000
H	-5.286346000	0.852103000	0.626065000
C	-5.572062000	0.391876000	-1.328473000
H	-4.956083000	0.240727000	-2.221183000
H	-6.236950000	-0.481434000	-1.227975000
H	-6.189405000	1.277788000	-1.493041000
N	3.032682000	0.972972000	-0.072781000
C	3.739621000	1.142854000	-1.361126000
H	3.025559000	1.483782000	-2.115653000
H	4.540761000	1.883373000	-1.279920000
H	4.167333000	0.190831000	-1.679333000
H	3.717212000	0.623578000	0.597318000
C	2.561355000	2.279409000	0.436539000
H	3.386449000	2.991668000	0.529177000
H	1.824350000	2.687802000	-0.257633000
H	2.095003000	2.148360000	1.414194000

Supplementary Table 20 Cartesian coordinates (Angstroms) for TS_p’ of *trans*-epoxy amines with dimethylamine-coordinated lanthanum(III)

Optimized energy: E(RwB97XD) = –936.884888243
Number of imaginary frequencies: 1
Imaginary frequencies = –470.6775 cm^{-1}
Zero-point correction = 0.290053 (Hartree/Particle)
Thermal correction to Energy = 0.306984
Thermal correction to Enthalpy = 0.307975
Thermal correction to Gibbs Free Energy = 0.243163

3 1

C	-1.849980000	0.862875000	-0.080050000
C	-1.738242000	-0.162654000	0.942068000
C	-2.675591000	-1.357914000	0.824104000
C	-3.833471000	-1.021271000	-0.131682000
H	-2.047523000	0.523421000	-1.088090000
H	-1.647855000	0.221507000	1.959087000
H	-2.136743000	-2.243874000	0.478876000
H	-3.071266000	-1.589090000	1.816237000
H	-3.524043000	-1.171819000	-1.172506000
H	-4.681040000	-1.695888000	0.043408000
O	-0.460457000	-0.195082000	0.304998000
La	1.641842000	-1.051381000	-0.143700000
C	-1.569145000	2.299262000	0.144708000
H	-0.892308000	2.694699000	-0.614218000
H	-2.521474000	2.828262000	0.038890000
H	-1.173882000	2.487581000	1.143557000
N	-4.185969000	0.390087000	0.013536000
H	-4.564674000	0.559529000	0.941133000
C	-5.118063000	0.877752000	-0.996660000
H	-4.650480000	0.804309000	-1.983328000
H	-6.059156000	0.311830000	-1.021997000
H	-5.345538000	1.927751000	-0.802674000
N	3.071936000	1.160617000	0.335787000
C	4.103518000	1.397883000	-0.696622000
H	3.616929000	1.595874000	-1.653850000
H	4.729301000	2.259158000	-0.442719000
H	4.741835000	0.518211000	-0.795706000
H	3.565728000	0.972415000	1.208292000
C	2.254023000	2.371630000	0.562411000
H	2.876623000	3.228420000	0.837551000
H	1.712787000	2.615032000	-0.354740000
H	1.531372000	2.181752000	1.358791000

Supplementary Table 21 Cartesian coordinates (Angstroms) for Int2_p' of *trans*-epoxy amines with dimethylamine-coordinated lanthanum(III)

Optimized energy: E(RwB97XD) = – 936.971161310
Number of imaginary frequencies: 0
Zero-point correction = 0.295228 (Hartree/Particle)
Thermal correction to Energy = 0.311348
Thermal correction to Enthalpy = 0.312340
Thermal correction to Gibbs Free Energy = 0.249987

3 1

C	-2.504712000	1.139919000	-0.287471000
C	-1.685419000	0.191356000	0.601994000
C	-2.584990000	-1.056928000	0.713822000
C	-4.021135000	-0.558471000	0.507164000
H	-2.510787000	0.741656000	-1.308067000
H	-1.591787000	0.662815000	1.594297000
H	-2.312852000	-1.750670000	-0.085446000
H	-2.463349000	-1.565754000	1.670579000
H	-4.490121000	-0.995989000	-0.373820000
H	-4.681105000	-0.696873000	1.361870000
O	-0.437360000	-0.058415000	0.077201000
La	1.473791000	-1.121221000	-0.162020000
C	-2.067948000	2.587617000	-0.264343000
H	-1.024710000	2.624822000	-0.584415000
H	-2.650387000	3.212077000	-0.944417000
H	-2.124519000	3.006078000	0.745447000
N	-3.900664000	0.929470000	0.244031000
H	-3.958563000	1.408042000	1.147375000
C	-4.997537000	1.466803000	-0.605018000
H	-4.928345000	1.009678000	-1.591698000
H	-5.948810000	1.214276000	-0.137773000
H	-4.897183000	2.548075000	-0.678584000
N	2.860652000	1.126611000	0.287940000
C	3.955818000	1.305238000	-0.686340000
H	3.531525000	1.386774000	-1.689970000
H	4.534969000	2.211645000	-0.481570000
H	4.623988000	0.441523000	-0.652580000
H	3.284665000	1.044601000	1.211049000
C	1.970869000	2.306181000	0.317104000
H	2.527622000	3.224299000	0.532121000
H	1.487568000	2.410810000	-0.657028000
H	1.197797000	2.163513000	1.073947000

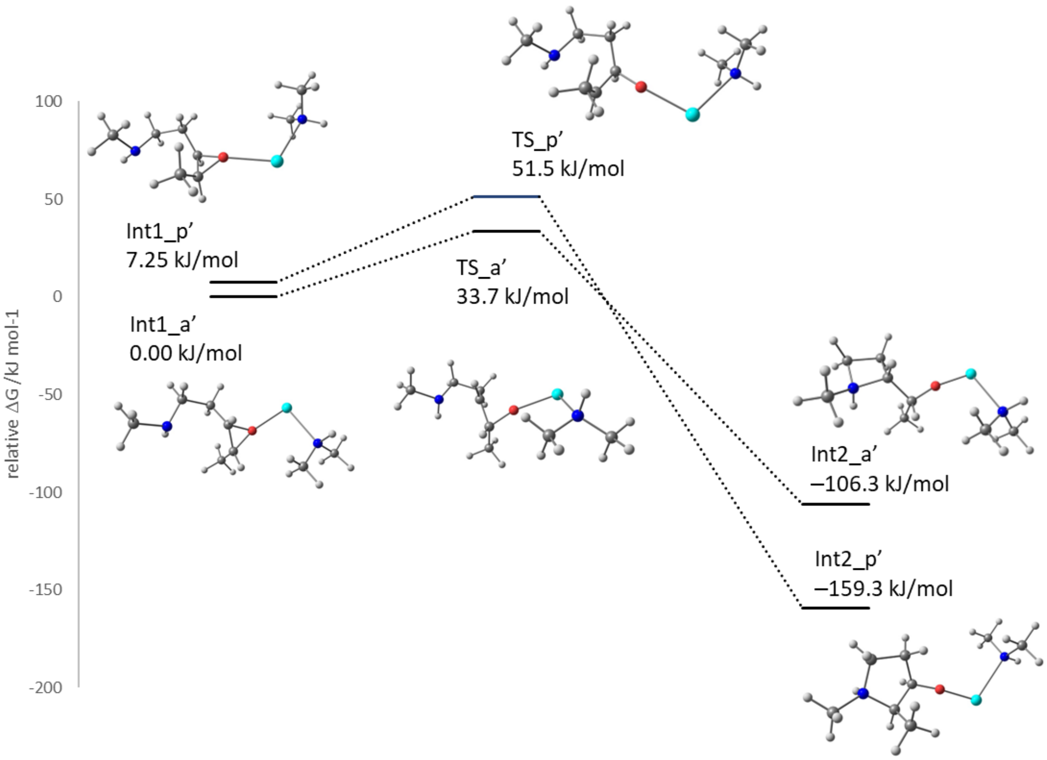

Supplementary Figure 6 Energy diagram of *cis*-epoxy amine intramolecular aminolysis with dimethylamine-coordinated lanthanum(III) at 84 °C (PCM (dichloroethane)/ωB97XD/6-311++G**, SDD (La)//PCM (dichloroethane)/ωB97XD/6-31G**, LanL2DZ (La)); lanthanum (light blue), Oxygen (red), nitrogen (blue), carbon (gray), hydrogen (white).

Supplementary Table 22 The calculated energy values of *cis*-epoxy amine with dimethylamine-coordinated lanthanum(III) (ZPE: zero point energy, kJ/mol)

	Azetidine formation			Pyrrolidine formation		
	Int1_a’	TS_a’	Int2_a’	Int1_p’	TS_p’	Int2_p’
relative ΔE	0.00	33.8	–118.7	1.12	44.7	–182.9
relative ΔE + ZPE	0.00	30.9	–109.8	3.64	44.5	–168.1
relative ΔH	0.00	29.7	–112.1	2.38	41.9	–172.6
relative ΔG	0.00	33.7	–106.3	7.25	51.5	–159.3

Supplementary Table 23 Cartesian coordinates (Angstroms) for Int1_a' of *cis*-epoxy amines with dimethylamine-coordinated lanthanum(III)

Optimized energy: E(RwB97XD) = –936.910485426
Number of imaginary frequencies: 0
Zero-point correction = 0.290465 (Hartree/Particle)
Thermal correction to Energy = 0.312290
Thermal correction to Enthalpy = 0.313421
Thermal correction to Gibbs Free Energy = 0.233128

3 1

C	-3.991010000	-0.861122000	-0.672666000
C	-1.621799000	-0.159172000	-0.915427000
C	-2.770748000	-0.286799000	0.046629000
C	-5.393373000	-0.655731000	-2.630992000
C	-1.100345000	1.098550000	-1.449688000
H	-1.413937000	-1.049028000	-1.506156000
C	-1.663210000	2.451075000	-1.148152000
O	-0.380683000	0.416107000	-0.368328000
H	-3.757636000	-1.876561000	-1.016648000
H	-4.821935000	-0.958317000	0.045717000
H	-3.008422000	0.678062000	0.503296000
H	-2.492663000	-0.968274000	0.856552000
H	-5.047681000	-1.603149000	-3.057787000
H	-5.653548000	0.009690000	-3.457219000
H	-6.307364000	-0.863419000	-2.051225000
H	-0.553645000	1.024241000	-2.386736000
H	-2.432228000	2.670239000	-1.894033000
H	-2.114403000	2.505109000	-0.156917000
La	1.246077000	-0.257200000	1.333474000
H	-0.886026000	3.213643000	-1.229724000
H	-4.614369000	0.861045000	-1.534085000
N	-4.321179000	-0.061761000	-1.841772000
N	2.935381000	-0.085852000	-0.713670000
H	3.215861000	-1.049702000	-0.892727000
C	4.175999000	0.655289000	-0.390881000
H	4.622970000	0.245416000	0.517293000
H	4.901047000	0.589060000	-1.207162000
H	3.933015000	1.706764000	-0.223920000
C	2.328616000	0.416336000	-1.964783000
H	3.060276000	0.438468000	-2.778236000
H	1.497707000	-0.230643000	-2.249677000
H	1.952372000	1.429205000	-1.804877000

Supplementary Table 24 Cartesian coordinates (Angstroms) for TS_a’ of *cis*-epoxy amines with dimethylamine-coordinated lanthanum(III)

Optimized energy: E(RwB97XD) = –936.897604291
Number of imaginary frequencies: 1
Imaginary frequencies = –502.3615 cm^{-1}
Zero-point correction = 0.289340 (Hartree/Particle)
Thermal correction to Energy = 0.310708
Thermal correction to Enthalpy = 0.311839
Thermal correction to Gibbs Free Energy = 0.233090

3 1

C	-9.037491000	-0.320608000	-1.861602000
C	-6.849888000	0.251122000	-2.358443000
C	-7.764912000	0.156377000	-1.164817000
C	-9.566354000	-0.362644000	-4.285475000
C	-6.044596000	1.409878000	-2.744031000
H	-6.643671000	-0.664542000	-2.900965000
C	-6.425420000	2.817822000	-2.376849000
O	-5.230472000	0.726724000	-1.794952000
H	-9.002399000	-1.404198000	-2.006846000
H	-9.964442000	-0.077969000	-1.332381000
H	-7.903578000	1.130606000	-0.693205000
H	-7.386812000	-0.542608000	-0.417155000
H	-9.130872000	-1.356985000	-4.414011000
H	-9.410608000	0.205028000	-5.204251000
H	-10.645183000	-0.475001000	-4.117178000
H	-5.629140000	1.341007000	-3.751366000
H	-7.207943000	3.174838000	-3.051134000
H	-6.782348000	2.891573000	-1.347967000
La	-3.650696000	0.111159000	-0.247335000
H	-5.556868000	3.470491000	-2.493659000
H	-9.196224000	1.284351000	-3.132913000
N	-8.897571000	0.314199000	-3.179194000
N	-1.980774000	0.288816000	-2.335169000
H	-1.697490000	-0.672735000	-2.520183000
C	-0.742784000	1.038042000	-2.030172000
H	-0.272763000	0.618808000	-1.137342000
H	-0.032666000	0.994116000	-2.861677000
H	-0.992926000	2.084181000	-1.840947000
C	-2.621122000	0.786047000	-3.569843000
H	-1.911361000	0.814866000	-4.402991000
H	-3.456145000	0.135451000	-3.832247000
H	-3.003277000	1.794927000	-3.400339000

Supplementary Table 25 Cartesian coordinates (Angstroms) for Int2_a' of *cis*-epoxy amines with dimethylamine-coordinated lanthanum(III)

Optimized energy: E(RwB97XD) = –936.955690272
Number of imaginary frequencies: 0
Zero-point correction = 0.293840 (Hartree/Particle)
Thermal correction to Energy = 0.314782
Thermal correction to Enthalpy = 0.315913
Thermal correction to Gibbs Free Energy = 0.237851

3 1

C	-4.492819000	-0.427810000	-0.384074000
C	-2.418757000	-0.361521000	-0.849521000
C	-3.212733000	-0.453844000	0.463928000
C	-3.995655000	-0.459734000	-2.917022000
C	-1.194087000	0.551638000	-0.962539000
H	-2.167343000	-1.353195000	-1.231343000
C	-1.471658000	2.029013000	-0.699539000
O	-0.272722000	0.047444000	-0.055139000
H	-4.882917000	-1.416924000	-0.622930000
H	-5.299744000	0.248864000	-0.111697000
H	-3.107571000	0.430294000	1.093769000
H	-3.051305000	-1.352416000	1.054639000
H	-3.935460000	-1.547000000	-2.885420000
H	-3.270432000	-0.061981000	-3.625955000
H	-5.002276000	-0.147734000	-3.194099000
H	-0.804251000	0.455162000	-1.988201000
H	-2.115980000	2.482033000	-1.460844000
H	-1.918285000	2.187043000	0.286699000
La	1.405114000	-0.249514000	1.325925000
H	-0.521239000	2.568263000	-0.731275000
H	-3.742242000	1.091756000	-1.598739000
N	-3.692926000	0.071380000	-1.570077000
N	2.912268000	-0.055807000	-0.923602000
H	3.431066000	-0.928789000	-1.002328000
C	3.903811000	1.032396000	-0.827492000
H	4.550939000	0.864733000	0.035522000
H	4.524689000	1.098925000	-1.727280000
H	3.379756000	1.982877000	-0.697933000
C	2.095687000	0.073644000	-2.144487000
H	2.717005000	0.057552000	-3.046511000
H	1.369541000	-0.739327000	-2.186476000
H	1.553031000	1.020629000	-2.111049000

Supplementary Table 26 Cartesian coordinates (Angstroms) for Int1_p' of *cis*-epoxy amines with dimethylamine-coordinated lanthanum(III)

Optimized energy: E(RwB97XD) = –936.910057170
Number of imaginary frequencies: 0
Zero-point correction = 0.291425 (Hartree/Particle)
Thermal correction to Energy = 0.312769
Thermal correction to Enthalpy = 0.313900
Thermal correction to Gibbs Free Energy = 0.235462

3 1

C	-1.822386000	-1.182089000	-0.415656000
C	-1.815450000	0.218828000	-0.840292000
C	-2.458987000	1.344593000	-0.083534000
C	-3.889150000	1.565711000	-0.596660000
H	-1.702367000	0.392836000	-1.908848000
H	-2.460403000	1.141056000	0.990180000
H	-1.876683000	2.257815000	-0.241316000
H	-4.282286000	2.487227000	-0.137157000
H	-3.854251000	1.741449000	-1.677156000
O	-0.601504000	-0.364789000	-0.267801000
La	1.784443000	-0.902893000	-0.098969000
H	-1.707148000	-1.916853000	-1.209534000
N	-4.736843000	0.406733000	-0.346558000
H	-5.489576000	0.399199000	-1.024598000
C	-2.441457000	-1.689693000	0.847887000
H	-1.942551000	-2.603942000	1.177622000
H	-2.395930000	-0.951589000	1.649402000
H	-3.491002000	-1.914104000	0.644221000
C	-5.314007000	0.391111000	0.991556000
H	-4.525679000	0.278277000	1.742557000
H	-5.879458000	1.304694000	1.237116000
H	-5.982411000	-0.467745000	1.090237000
N	3.459053000	1.146202000	0.254079000
C	3.411015000	2.120528000	-0.857083000
H	2.403486000	2.539311000	-0.920858000
H	4.120591000	2.938524000	-0.701335000
H	3.648800000	1.620957000	-1.797375000
H	4.402546000	0.762135000	0.269886000
C	3.247003000	1.820531000	1.553745000
H	3.960143000	2.637269000	1.700056000
H	2.234142000	2.229940000	1.583822000
H	3.363861000	1.099123000	2.364267000

Supplementary Table 27 Cartesian coordinates (Angstroms) for TS_p’ of *cis*-epoxy amines with dimethylamine-coordinated lanthanum(III)

Optimized energy: E(RwB97XD) = –936.893445612
Number of imaginary frequencies: 1
Imaginary frequencies = –495.4038 cm^{-1}
Zero-point correction = 0.290382 (Hartree/Particle)
Thermal correction to Energy = 0.311215
Thermal correction to Enthalpy = 0.312346
Thermal correction to Gibbs Free Energy = 0.235696

3 1

C	-2.244760000	-0.991925000	-0.315806000
C	-1.659764000	0.304378000	-0.629286000
C	-2.213662000	1.489299000	0.115685000
C	-3.638262000	1.647267000	-0.451123000
H	-1.609086000	0.506199000	-1.703242000
H	-2.228890000	1.309131000	1.193948000
H	-1.627159000	2.392891000	-0.067743000
H	-4.204925000	2.376731000	0.138030000
H	-3.570202000	2.038994000	-1.470270000
O	-0.513475000	-0.397132000	-0.183481000
La	1.692768000	-1.111060000	-0.176904000
H	-2.287403000	-1.707767000	-1.133084000
N	-4.318603000	0.342076000	-0.494487000
H	-4.774432000	0.220389000	-1.390694000
C	-2.579021000	-1.511463000	1.033116000
H	-1.928367000	-2.356887000	1.271090000
H	-2.503359000	-0.760063000	1.817563000
H	-3.597727000	-1.905600000	1.004127000
C	-5.293791000	0.141465000	0.573098000
H	-4.799095000	0.212257000	1.545591000
H	-6.102969000	0.883443000	0.549808000
H	-5.734136000	-0.854868000	0.485907000
N	3.315806000	0.995008000	0.185494000
C	3.143791000	2.006522000	-0.879061000
H	2.120057000	2.388192000	-0.851521000
H	3.830869000	2.848068000	-0.746623000
H	3.328234000	1.550549000	-1.853940000
H	4.273677000	0.653517000	0.124631000
C	3.155369000	1.608685000	1.517878000
H	3.840664000	2.450848000	1.656798000
H	2.130599000	1.973489000	1.624609000
H	3.347956000	0.865647000	2.293307000

Supplementary Table 28 Cartesian coordinates (Angstroms) for Int2_p’ of *cis*-epoxy amines with dimethylamine-coordinated lanthanum(III)

Optimized energy: E(RwB97XD) = –936.980141366
Number of imaginary frequencies: 0
Zero-point correction = 0.296080 (Hartree/Particle)
Thermal correction to Energy = 0.316208
Thermal correction to Enthalpy = 0.317339
Thermal correction to Gibbs Free Energy = 0.242094

3 1

C	-2.714443000	-0.640517000	-0.100380000
C	-1.583476000	0.237209000	-0.687996000
C	-1.972432000	1.662471000	-0.237815000
C	-3.499991000	1.651624000	-0.101830000
H	-1.660180000	0.179058000	-1.785216000
H	-1.502340000	1.863268000	0.727321000
H	-1.630782000	2.415833000	-0.947859000
H	-3.837116000	1.876745000	0.909809000
H	-4.023668000	2.306057000	-0.797232000
O	-0.330658000	-0.153581000	-0.282877000
La	1.678779000	-1.027641000	-0.141238000
H	-2.842813000	-1.573855000	-0.652694000
N	-3.917265000	0.224226000	-0.405458000
H	-4.056656000	0.164156000	-1.416494000
C	-2.560819000	-0.915111000	1.383019000
H	-1.600562000	-1.412474000	1.526460000
H	-2.551615000	0.001193000	1.979208000
H	-3.342624000	-1.576754000	1.758957000
C	-5.204818000	-0.177546000	0.221544000
H	-5.124339000	-0.072116000	1.301645000
H	-5.987930000	0.477982000	-0.156995000
H	-5.419497000	-1.211426000	-0.046197000
N	3.292741000	1.094174000	0.212518000
C	3.129054000	2.089691000	-0.864595000
H	2.104512000	2.469758000	-0.848882000
H	3.815319000	2.934189000	-0.742282000
H	3.317213000	1.619302000	-1.832016000
H	4.251770000	0.754119000	0.166034000
C	3.113920000	1.715918000	1.536780000
H	3.790781000	2.564713000	1.680916000
H	2.084941000	2.074029000	1.627126000
H	3.300169000	0.979146000	2.320163000

3 Reference

[1] P. L. Anelli, C. Biffi, F. Montanari, S. Quici, *J. Org. Chem.* **1987**, *52*, 2559-2562.
[2] X. H. Zeng, C. X. Miao, S. F. Wang, C. G. Xia, W. Sun, *Chem. Commun.* **2013**, *49*, 2418-2420.
[3] E. Rasolofonjatovo, B. Tréguier, O. Provot, A. Hamze, J.-D. Brion, M. Alami, *Eur. J. Org. Chem.* **2012**, 1603-1615
[4] E. Vasilikogiannaki, I. Titilas, G. Vassilikogiannakis, M. Stratakis, *Chem. Commun.* **2015**, *51*, 2384-2387.
